# How many mosquito nets are needed to maintain universal coverage: an update

**DOI:** 10.1186/s12936-023-04609-z

**Published:** 2023-06-30

**Authors:** Hannah Koenker, Josh Yukich, Marcy Erskine, Robert Opoku, Eleanore Sternberg, Albert Kilian

**Affiliations:** 1 Tropical Health LLP, Baltimore, USA; 2 Center for Applied Malaria Research and Evaluation, Tulane University, New Orleans, USA; 3 International Federation of Red Cross and Red Crescent Societies, Geneva, Switzerland; 4 International Federation of Red Cross and Red Crescent Societies, Nairobi, Kenya; 5 Tropical Health LLP, Liverpool, UK; 6 Tropical Health LLP, Montagut, Spain

## Abstract

**Background:**

Insecticide-treated nets (ITNs) have served as the cornerstone of malaria vector control in sub-Saharan Africa for the past two decades. Over 2.5 billion ITNs have been delivered since 2004 primarily through periodic mass distribution campaigns scheduled at approximately three-year intervals, aligning with the expected lifespan of nets. Recent work indicates that ITN retention times are less than two years in most countries, raising key questions for quantification approaches and delivery frequency for ITN distribution. This paper models several quantification approaches for five typical ITN distribution strategies, estimates the proportion of the population with access to an ITN, and presents recommended quantification approaches to meet global targets for ITN access and use.

**Methods:**

A stock and flow model with annual timesteps was used to model ITN distribution and resulting ITN access for 2020–2035 under five scenarios in 40 countries: (1) three-year mass campaigns, (2) full-scale annual continuous distribution, (3) three-year mass campaigns plus continuous distribution in the years between campaigns, (4) three-year mass campaigns at different quantification approaches, (5) two-year mass campaigns at different quantification approaches. All scenarios included ITN distribution to pregnant women at antenatal clinics and infants at immunization visits.

**Results:**

The current status quo of conducting mass campaigns every three years using a population/1.8 quantifier is insufficient to achieve or maintain targets of 80% population access to ITNs in most malaria-endemic countries, given most estimated retention times are less than three years. Tailored three- or two-year mass campaigns were less efficient than annual continuous distribution strategies in nearly all settings. For countries with at least 2.5 year median ITN retention times, full scale continuous distribution provided better ITN access while needing 20-23% fewer ITNs compared to current mass campaigns.

**Conclusion:**

Given variation in ITN retention times across countries, tailored quantification approaches for mass campaigns and continuous distribution strategies are warranted. Continuous distribution strategies are likely to offer more efficient ways to maintain ITN coverage, with fewer nets, where ITN retention times are at least two and a half years. National malaria programmes and their funding partners should work to increase the number of ITNs available to those vulnerable to malaria, while at the same time working to extend the useful life of these critical commodities.

**Supplementary Information:**

The online version contains supplementary material available at 10.1186/s12936-023-04609-z.

## Background

Insecticide-treated nets (ITNs) have served as the cornerstone of malaria vector control in sub-Saharan Africa for the past two decades. Over 2.5 billion ITNs have been delivered to countries since 2004 [1], primarily through periodic mass distribution campaigns scheduled at approximately three-year intervals, aligning with the expected lifespan of nets. Recent work has shown significant variation in ITN durability across geographic zones, and while some studies support a three-year median lifespan, multi-country analyses of ITN retention times indicate half of countries can expect two years or less of useful life for the majority of nets they distribute [2]. The shorter-than-expected retention times have important implications for the way countries quantify ITN commodity needs for mass campaigns, and raise several key questions. First, what is the projected impact of the mismatch in campaign cycle and ITN retention in terms of overall ITN coverage? Second, if mass campaigns every three years are insufficient due to ITNs lasting only one to two years, is switching to a two-year campaign cycle indicated, or are there alternative or supplemental ways to distribute ITNs to ensure high rates of ITN access are maintained over time? Third, with what is now known about ITN retention and ITN distribution modalities, is “population divided by 1.8” (as recommended since 2010 [3, 4]) the correct quantification approach for mass campaigns for all countries? Finally, what would optimum ITN quantification look like for countries given their particular ITN retention times, aiming to sustain high levels of ITN access (the necessary, but not sufficient, precursor to ITN use)?

In practical terms, quantification for ITN distribution has referred to a process whereby national malaria programmes estimate the number of ITNs required for mass campaigns by dividing the total population (or population living within areas targeted for the ITN campaign) by 1.8, which is intended to achieve an end result of households owning one ITN for every two people. The quantifier 1.8 was selected to account for the 47–59% of households who have an odd number of household members, reflecting the need to round up the number of ITNs allocated to households in these cases [3]. Kilian et al*.* originally recommended dividing by 1.6, in order to accommodate distribution challenges including outdated census information and the need to preposition full bales of nets rather than precisely subdividing them. In practice, the World Health Organization (WHO) recommends “population divided by 1.8”, and allows a buffer of up to 10% when the previous census is over five years old.

Continuous distribution (CD) is the delivery of ITNs on an annual or continuous basis, typically through school-based distribution or through community volunteers who deliver coupons to eligible families on demand, that can then be redeemed for ITNs at health facilities or other community storage points. Continuous distribution can occur at a limited scale between mass campaigns to offset losses of nets, as in Ghana, or at full-scale as a replacement for mass campaigns, as in Tanzania. The goal of continuous distribution strategies is to maintain high rates of population ITN access more consistently throughout a given three-year period, in contrast to campaigns whose cohorts of nets wear out en masse in a given geographic area [5]. Regardless of whether mass campaigns and/or continuous distribution channels are used, the WHO recommends implementing ITN distribution to pregnant women and infants as a routine intervention [4].

Quantification for continuous ITN distribution lacks a simple “population divided by X” quantifier. It is most straightforward for distribution to pregnant women and infants, as a) these populations are relatively consistent at around 4–5% and 4% of the population in sub-Saharan Africa at any given time, respectively [6], and b) attendance rates at antenatal care visits (ANC) and immunization visits (EPI) are generally well-monitored through the national health management information system (HMIS). Annual procurements of ITNs for these channels are thus quite predictable. However, for ITN distribution through schools or community channels, quantification has been particularly challenging. The first large-scale pilots of these channels relied on modelling with NetCALC [5], running individual scenarios to fine-tune the number of nets that would produce desired levels of ITN access. No rule of thumb quantifier was produced. It is likely that this gap has contributed in part to the limited scale-up of continuous distribution channels across malaria-endemic countries.

This paper explores the above questions using an existing stock and flow model [7] to project population ITN access for countries in sub-Saharan Africa over five different distribution scenarios, using estimated ITN retention times from Bertozzi-Villa et al*.* [2] and varying quantification approaches within each distribution scenario. Recommended quantifiers for each country are presented for each scenario and at different target levels of population ITN access.

## Methods

### Projections of future coverage

**Fig. 1:**
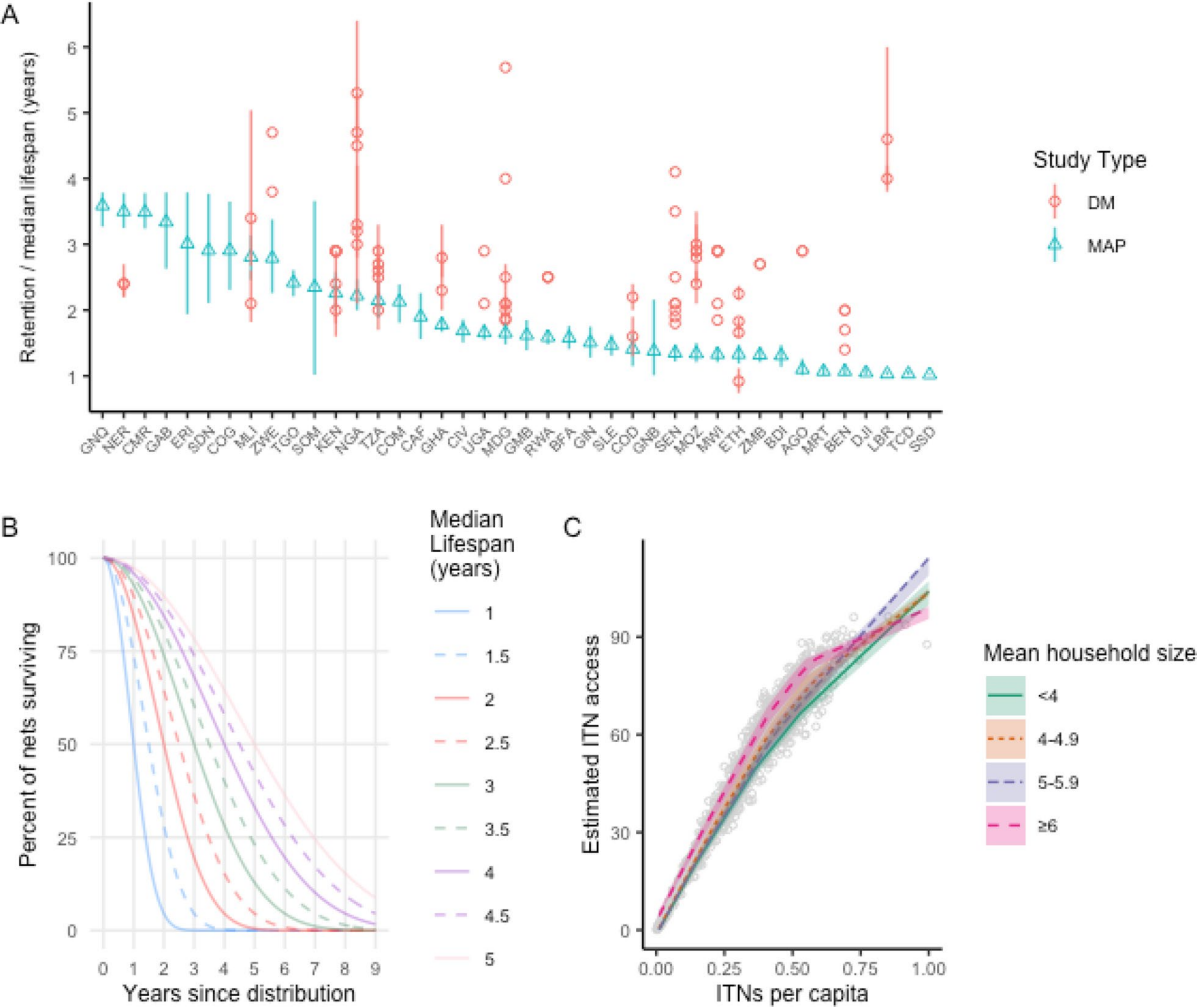
**A** ITN retention times estimated by Malaria Atlas Project (MAP) and median lifespans estimated from durability monitoring (DM) studies; countries are labeled by IS03 code. **B** Smooth-compact loss function for net decay **C** Nonparametric conditional quantile function for ITN access as a function of nets-per-capita (NPC)

ITNs were distributed in the model for each scenario as shown in Table 1. For each year, the stock and flow model used a country-specific estimated median lifespan from Malaria Atlas Project (MAP) estimates published in Bertozzi-Villa et al*.* [2], shown in blue in Fig. 1A, to decay each crop of distributed nets in one-year time steps. Figure 1A also presents estimated median lifespans from ITN durability monitoring (DM) activities gathered from U.S. President’s Malaria Initiative reports (www.pmi.gov) and published literature [8–15]. The net decay functions rely on smooth-compact loss function developed by Nakul Chitnis and described in Koenker et al*.* and Bhatt et al*.* [5, 7, 16], and are shown in Fig. 1B. Each country was assigned an indicative population of 10 million people in the database, starting in 2020, and an annual population growth rate of 3%, as the model outputs are adjusted for population and thus do not require specific population estimates. The model was built and run in Stata (17, StataCorp LLC, College Station, TX).

**Table 1:** Distribution Scenarios and their ITN inputs through mass campaign, routine (ANC/EPI), and continuous (school or community) channels

Scenario	Mass campaign	ANC/EPI (routine)	Annual school/ community	Number of different models per scenario
1. “Status quo”	In 2022, 2025, 2028, 2031, 2034 at population/1.8	2020–2035, varying from population × 5% to population × 7%	None	3
2. “Full-scale continuous”	In 2020, to establish high coverage at population/1.8	2020–2035 using population × 6%	2022–2035 varying the CD quantifier from population × 0% to population × 50%	51
3. “Mass plus continuous”	In 2022, 2025, 2028, 2031, 2034 at population/1.8	2020–2035 using population × 6%	Only in years between campaigns, varying the CD quantifier from population × 0% to population × 40%	41
4. “Varying 3-year mass”	In 2022, 2025, 2028, 2031, 2034, varying from population / 0.1–2.0	2020–2035 using population × 6%	None	20
5. “Varying 2-year mass”	In 2022, 2024, 2026, 2028, 2030, 2032, 2034 varying from population/0.5–2.0	2020–2035 using population × 6%	None	16
			Total models	131

Population refers to the total population living in areas targeted for ITNs

*ANC* antenatal care; *EPI* expanded program on immunization; *CD* continuous distribution

The total net crop (consisting of all surviving nets from various channels to date) was summed for each year and country. This was then divided by the projected population to calculate nets-per-capita (NPC). To estimate ITN access from NPC, a nonparametric conditional quantile function for ITN access as a function of NPC was estimated from 155 Demographic and Health Surveys (DHS) and Malaria Indicator Surveys (MIS), stratifying by average household size, using the “quantreg” R package (v5.93) [7, 17–19]. A grid of 100 points was produced and used to predict ITN access from NPC (Fig. 1C). Confidence intervals for both estimated median lifespan and the function of ITN access vs NPC were used to generate an overall confidence interval around the estimate of ITN access.

Recommended quantifiers were obtained by identifying the scenario that provided the greatest number of years of ITN access at or above the target level; if multiple scenarios provided the same number of years, the scenario requiring the fewest nets was selected.

### Scenarios

To inform recommendations for quantification of ITNs, the above process was used to model ITN distributions under five typical ITN distribution scenarios, varying quantification approaches within each scenario. The majority of malaria-endemic countries currently implement Scenario 1; Tanzania has implemented Scenario 2 since 2013 in a subset of regions, while Ghana has implemented Scenario 3 since 2012. While countries aim to deliver mass campaigns every three years, some have recently argued for campaign every two years to offset shorter median net lifespans.

“Status quo”: Mass campaigns every three years with routine distribution of ITNs to pregnant women and infants through antenatal clinics (ANC) and immunization visits (EPI). Quantification of the mass campaigns was fixed at population/1.8 while quantification of routine distribution varied from population × 5–7%.

“Full-scale continuous”: Full-scale annual school distribution of ITNs with routine distribution of ANC and EPI ITNs, fixing the routine distribution at population × 6% and varying the quantification of school distributions from population × 0–50%.

“Mass plus continuous”: Mass campaign every three years with ongoing routine distribution of ANC and EPI ITNs and with annual school distribution in a limited number of classes (or limited community distribution) in the years between campaigns. Quantification of the mass campaigns was fixed at population/1.8 and routine distribution at population × 6%, varying the annual school/community distribution between population × 0–40%.

“Varying three-year mass”: Mass campaigns every three years with routine distribution of ITNs to pregnant women and infants through ANC and EPI. Quantification of routine distribution was fixed at 6%, and quantification of the mass campaigns was varied from population/0.1 (ten nets per person) to population/2.0 (one net for two people) in increments of 0.1.

“Varying two-year mass”: Mass campaigns every two years with routine distribution of ITNs to pregnant women and infants through ANC and EPI. Quantification of routine distribution was fixed at 6%, and quantification of the mass campaigns was varied from population/0.5 (two nets per person) to population/2.0 (one net for two people) in increments of 0.1.

All scenarios with mass campaigns began with a mass campaign in 2022 and ended in 2035. The “full scale continuous” scenario assumed a mass campaign in 2020, quantified with population/1.8, to scale up coverage prior to switching over to a fully continuous ITN strategy in 2022. Plots for all scenarios are available as Additional file 1.

To assess feasibility of large-scale school distribution in relation to optimal quantifiers, the proportion of the population that are primary school students currently attending school was calculated from the most recent DHS for each country, obtained with permission from dhsprogram.com.

## Results

Given a target of 80% ITN access, the recommended quantification approaches for each scenario varied considerably across countries. Adjustments in quantification for ANC-EPI distribution did not lead to large differences in ITN access in Scenario 1. The key factors driving variation across countries within a given scenario were the estimated retention times for each country and the mean household size. Recommended quantification approaches are summarized for the scenarios that include continuous distribution in Table 2, for three-year mass campaigns in Table 3, and for two-year mass campaigns in Table 4. Six countries are included in these tables representing a range of ITN retention times from one year to three and a half years at approximately half-year intervals. The complete tables including all countries are available in Additional file 2, Tables S1, S2, and S3.

**Table 2:** Recommended annual quantifiers for continuous distribution channels for six countries with a range of estimated ITN retention times; i.e. Togo should quantify the nets needed for full-scale continuous distribution by multiplying their population by 17%, each year, to maintain 80% ITN access

Minimum quantifier (population x quantifier, annually) to sustain ITN access at or above specified target level							
		Scenario 2 (full continuous distribution strategy)			Scenario 3 (continuous distribution between mass campaigns)		
		Targeted ITN access level					
Country (ISO3 code)	Retention time (years)	70%	80%	90%	70%	80%	90%
LBR	1.0	28	36	46	27	35	
GIN	1.5	18	28	36	11	16	29
TZA	2.1	14	21	28	4	11	20
TGO	2.4	12	17	24	1	8	16
COG	2.9	10	13	20	0	4	15
CMR	3.5	8	11	14	0	1	9

Liberia has a blank value for Scenario 3 at the 90% ITN access target because the target level is not achievable in the model; it would require a quantifier greater than 40%. All scenarios assume that ANC and EPI delivery of ITNs is ongoing and provides nets to 6% of the population, in addition to any CD nets. The six countries shown here (labeled by IS03 code) represent the range of ITN retention times from 1 year to 3.5 years at approximately 6-month intervals. Results for all countries are available in Additional file 2, Table S1.

**Table 3:**
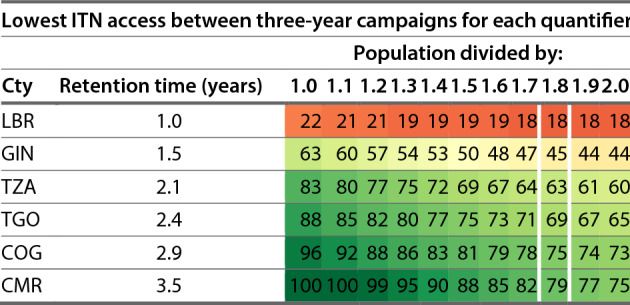
Lowest level of ITN access between three-year campaigns at different population quantifiers

Values of 100 indicate excess nets in the system (for example Cameroon). Routine ITN delivery to pregnant women and infants is assumed. The six countries shown here represent the range of ITN retention times from 1 year to 3.5 years at approximately 6-month intervals. Results for all countries are available in Additional file 2, Table S2.

**Table 4:**
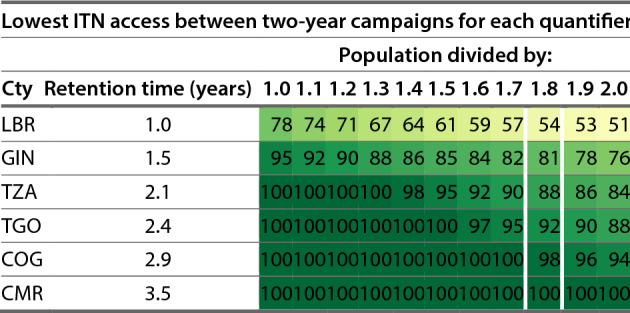
Lowest level of ITN access between two-year campaigns at different population quantifiers

Values of 100 indicate excess nets in the system (for example Cameroon). Routine ITN delivery to pregnant women and infants is assumed. The six countries shown here represent the range of ITN retention times from 1 year to 3.5 years at approximately 6-month intervals. Results for all countries are available in Additional file 2, Table S3.

For Scenario 2, which relies on full-scale annual continuous distribution in combination with routine ANC/EPI ITN delivery to maintain access, the annual quantifier needed to maintain ITN access at 70% ranged from 7% of the population in Eritrea (estimated net retention time of 3.01 years), to 30% of the population in South Sudan and Chad (retention times of 1.02 and 1.03 years, respectively). Similarly, to maintain ITN access at 80%, the quantifier ranged from 10% of the population in Eritrea, to 37% of the population in Djibouti. For ITN access to be maintained at 90% required a quantifier of between 14% of the population in Cameroon (retention time of 3.49 years) and 46% of the population in Liberia (1.03 years retention time; Table 2).

For Scenario 3, where mass campaigns are conducted every three years, routine distribution of ITNs through ANC/EPI is conducted consistently, and continuous distribution supplements ITN access in the years between campaigns, there was also a range of quantifiers for the annual continuous distribution channels. At the 70% target, ten countries required ITNs equivalent to 0% of the population, essentially indicating that three-year campaigns with ANC and EPI distribution would maintain ITN access at 70%. For countries with the shortest retention times like South Sudan, continuous distribution quantified using population × 28% was needed to maintain ITN access between campaigns. Two countries were able to maintain the 80% access target with no continuous distribution ITNs between campaigns (Eritrea and Mali), while South Sudan was estimated to need 36% of the population in ITNs in each year between campaigns. Equatorial Guinea, with an estimated net retention time of 3.59 years, was the country with the lowest quantifier that maintained ITN access at 90% between campaigns, with population times 8% between campaigns. Table 2 summarizes the recommended quantifiers for six countries, to maintain ITN access at 70%, 80%, or 90%; full results are in Additional file 2, Table S1.

For Scenario 4, the quantifier used for three-year mass campaigns (in combination with routine ITN distribution at ANC/EPI clinics) was varied from 0.1 to 2.0. The lowest level of ITN access between campaigns is shown in Table 3. Under the current recommended quantifier of 1.8, only Eritrea and Mali, both with net retention times of over three years, were estimated to maintain ITN access at or above 80% between campaigns. Even at population divided by 1.6, only six countries (15% of countries in the sample) maintained ITN access above 80%, likewise all with net retention times of at least three years. In Guinea, with the standard quantifier of population / 1.8 and an estimated retention time of 1.51 years, the country reaches a low point of 45% ITN access between campaigns. Using population / 1.1 would result in an estimated low point of 60% population ITN access between campaigns.

Table 4 provides a similar picture but for campaigns conducted every two years. Under a population / 1.8 quantifier, eighteen countries (45%) including Central African Republic, Rep. Congo, Cameroon, Eritrea, Gabon, Gambia, Guinea, Equatorial Guinea, Kenya, Comoros, Mali, Niger, Nigeria, Sudan, Somalia, Togo, Tanzania, and Zimbabwe would all maintain ITN access at or above 80% between campaigns. In other countries, two-yearly campaigns closer to one ITN per person would be needed in order to maintain ITN access at the 80% target. For example, Liberia reaches a low point of 54% ITN access in the year between campaigns using population/1.8. Using population/1.0 (one net per person, every two years) would result in an estimated ITN access of 78% between campaigns.

**Fig. 2:**
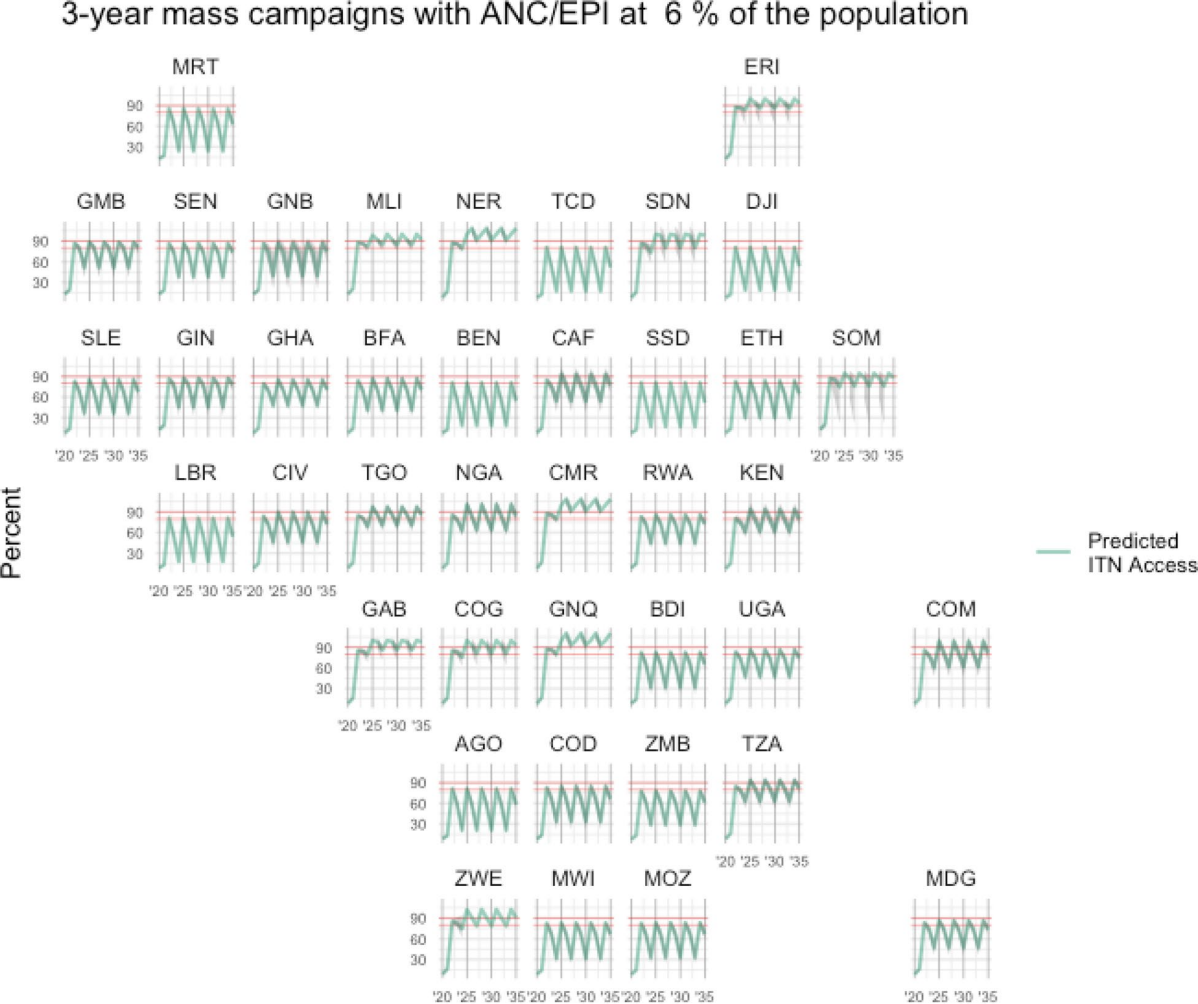
Estimated ITN access under a typical three-year mass campaign strategy, with ANC/EPI distribution at 6% of the population annually. Red lines indicate 80% and 90% ITN access targets

**Fig. 3:**
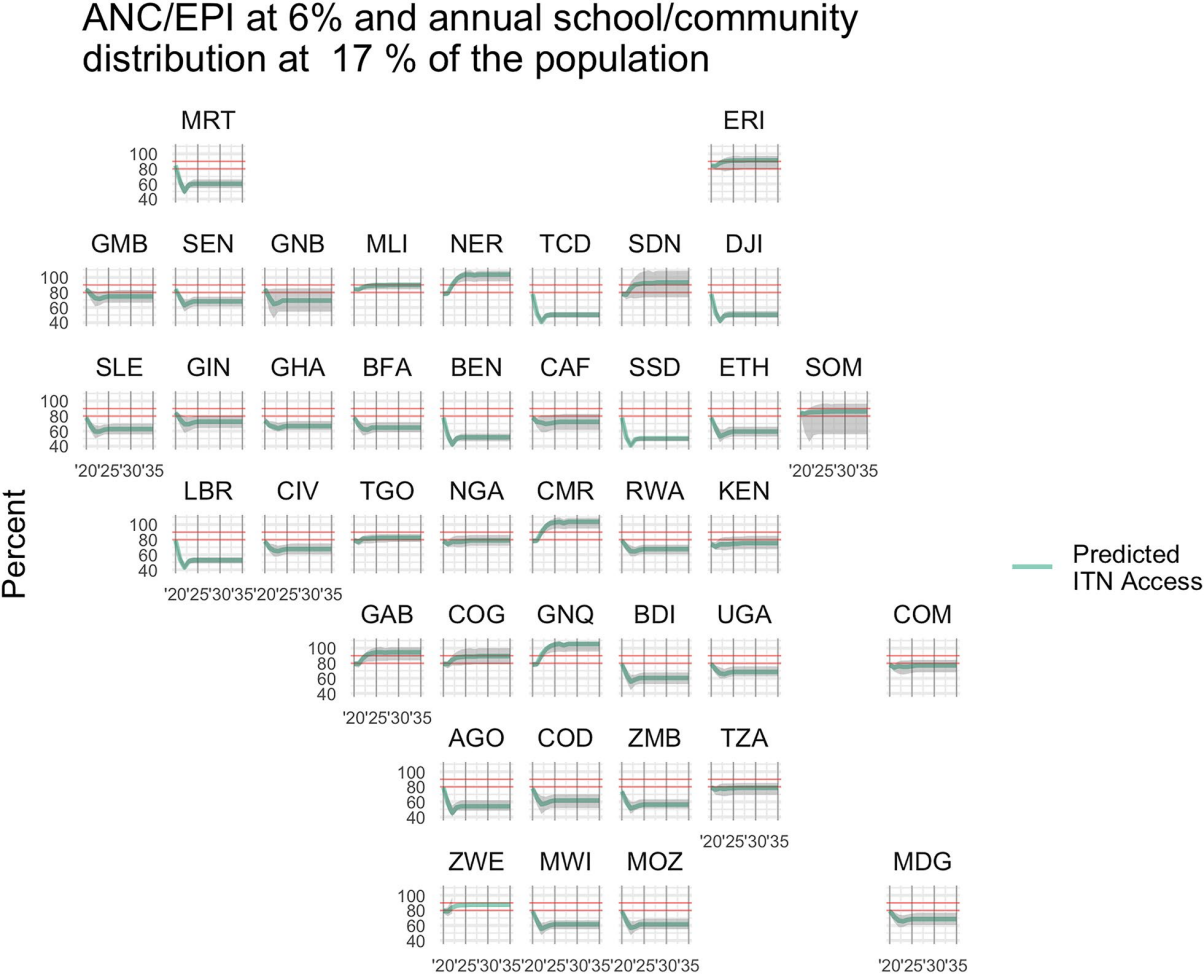
Estimated ITN access with annual ANC/EPI at 6% and full continuous distribution strategy at 17% of the population in nets each year. Shaded areas indicate 95% confidence intervals accounting for both net retention times and ITN access as a function of nets-per-capita (NPC). Red lines indicate 80% and 90% ITN access targets

**Fig. 4:**
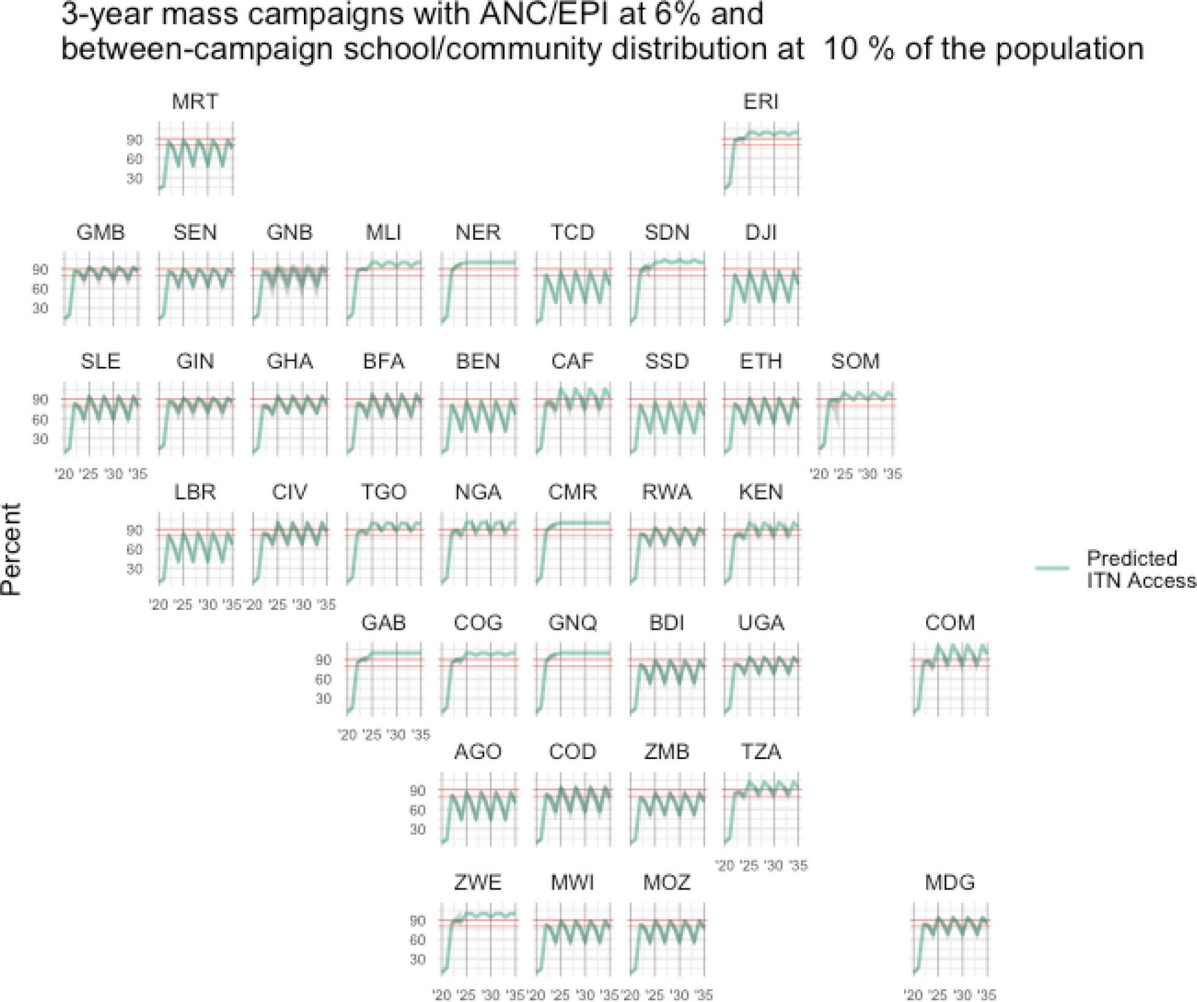
Scenario 3—Estimated ITN access under three-year mass campaigns with ANC/EPI distribution at 6%, and between-campaign continuous distribution at 10%. Red lines indicate 80% and 90% ITN access targets

The below figures illustrate scenario results for each country, for Scenario 1 (Fig. 2), Scenario 2 (Fig. 3), and Scenario 3 (Fig. 4), with predicted population ITN access estimates shown in green and target levels of ITN access highlighted in red at 80% and 90%. The typical rise and fall of ITN access is apparent in Fig. 2 and Fig. 4, while ITN access is maintained at a steady rate in Fig. 3 where distributions are annual through continuous channels.

The complete set of graphs for all 131 scenarios is included as Additional File 1.

Table 5 summarizes the recommended quantifiers under each scenario, given a target of maintaining 80% population ITN access. For Liberia, with a median retention time of just over one year, a full scale continuous distribution strategy would need to have an annual ITN need of population × 36%, and a strategy implementing mass campaigns every three years using population/1.8 and adding continuous distribution between campaigns would need to quantify the continuous distribution channel in each non-campaign year using population × 35%. Both strategies assume that ITN delivery to pregnant women and infants is ongoing, reaching about 6% of the population. Under tailored mass campaign scenarios, the models suggest that when net lifetimes are around 12 months, no three-year campaign strategy can achieve universal access; a strategy of campaigns every two years would only maintain ITN access above 80% if the quantification approach is to multiply the population by 0.9—delivering, in essence, one net per person every other year. In contrast, countries with longer retention times such as Republic of Congo or Cameroon are able to sustain 80% ITN access with full scale continuous strategies that quantify the annual ITN need using population × 13% or 11%, respectively, or could choose to implement continuous distribution between campaigns to sustain access with only population × 4% or 1%, respectively. Alternately, they could implement three-yearly campaigns with no continuous distribution, using population divided by 1.5 and 1.7, always along with ITN delivery to pregnant women and infants.

**Table 5:** Summary of recommended quantifiers for scenarios, to maintain ITN access at or above 80%. The quantifiers are for only the continuous distribution channel or mass campaign; annual ANC/EPI distribution equivalent to 6% of the population is assumed in each scenario, but is not part of the listed quantifiers. Results for all countries are available in Additional file 2, Table S4.

		Continuous distribution ITNs = population x X, annually		Mass Campaign ITNs = Population / X	
Country	Retention time (years)	Scenario 2: full-scale continuous + routine (%)	Scenario 3: campaign + routine + continuous between campaigns (%)	Scenario 4: three-yearly campaigns	Scenario 5: two-yearly campaigns
LBR	1.0	36	35		0.9
GIN	1.5	28	16	0.6	1.8
TZA	2.1	21	11	1.0	2.0
TGO	2.4	17	8	1.2	2.0
COG	2.9	13	4	1.5	2.0
CMR	3.5	11	1	1.7	2.0

**Fig. 5:**
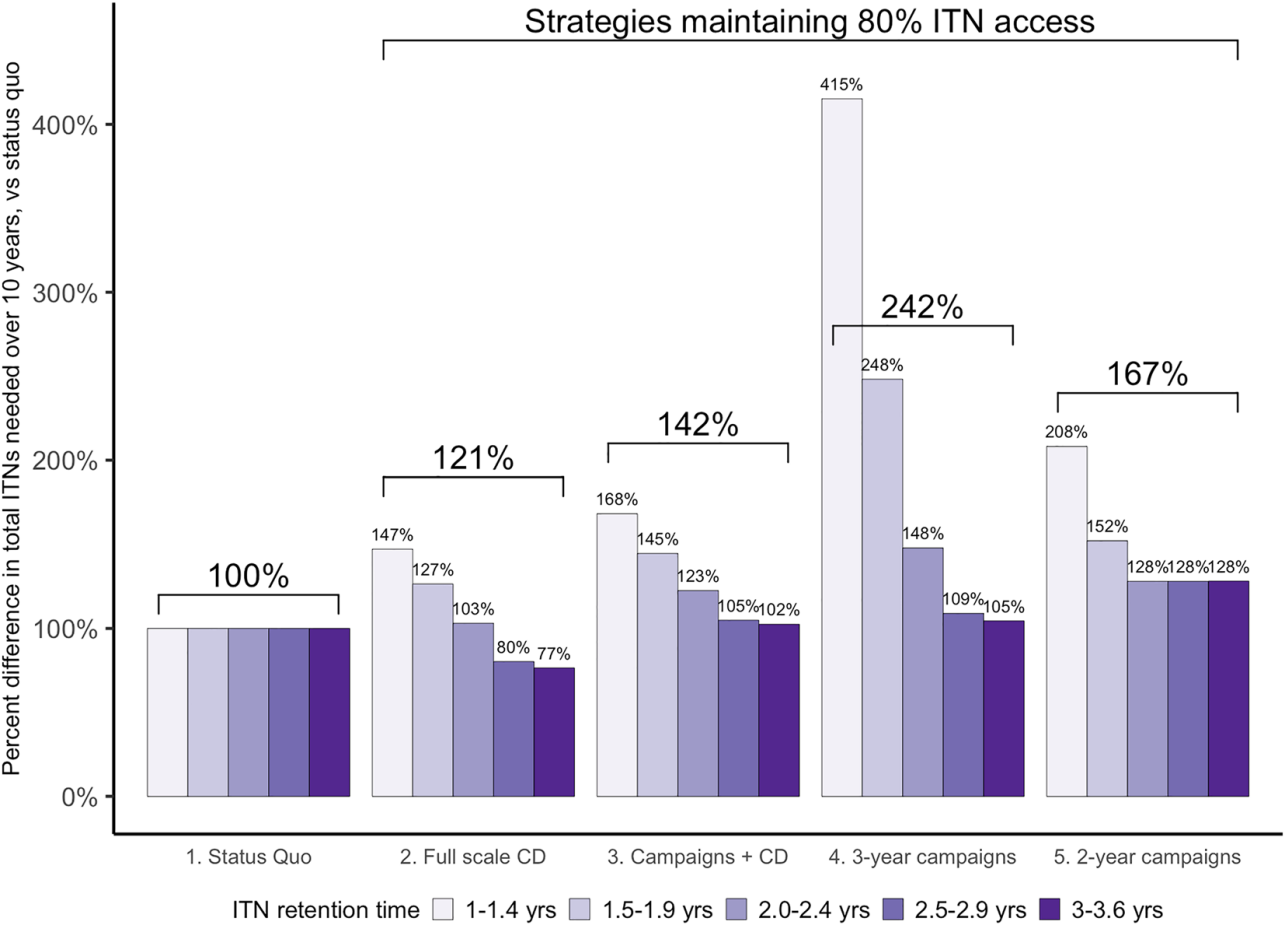
Relative proportions of total ITNs needed over 10 years, compared to status quo (three-yearly campaigns at population/1.8), by scenario and ITN retention time. Grey labels show group average for each scenario

The total number of nets needed under each recommended scenario in Table 5 to maintain 80% ITN access is presented in Fig. 5, summarized by ITN retention times grouped in half-year intervals. For comparability, results are presented as a percentage difference compared to a status quo scenario of three-yearly campaigns quantified with population/1.8 plus routine distribution of ITNs to pregnant women and infants through ANC and EPI channels. Continuous distribution required fewer nets to maintain ITN access at 80% for countries with ITN retention times of at least two and a half years (11 of the 40 study countries). The full scale CD scenario required 20% fewer nets for retention times of 2.5–2.9 years, and 23% fewer nets where median retention times were at least three years. Across all countries, full-scale continuous distribution was expected to maintain ITN access at 80% with 21% more ITNs than status quo. Strategies deploying continuous distribution between mass campaigns required 42% more ITNs than status quo across all countries. For countries with ITN retention times of less than two and a half years, 80% ITN access was maintained but required more nets than currently being procured, while for countries with retention times of 2.5–2.9 years the relative number of nets required was 5% higher, and 2% higher where median retention times were at least three years.

Three-yearly mass campaign strategies with tailored quantifiers required nearly two and a half times as many ITNs compared to status quo, although for countries with ITN retention times of at least 2.5 years, only 9% (2.5–2.9 years) and 5% (3.0–2.6 years) more nets would be needed. Overall, 67% more ITNs would be required for tailored two-yearly campaigns to maintain 80% access, compared to status quo, and 28% more nets were required under this scenario even for countries with at least two-year ITN retention times. Comparing the full-scale CD results to the tailored three- and two-year campaign results provides a direct comparison of nets required for each strategy to achieve similar ITN access and demonstrates that 80% ITN access is more efficiently achieved by continuous strategies than by tailored mass campaign strategies.

**Fig. 6:**
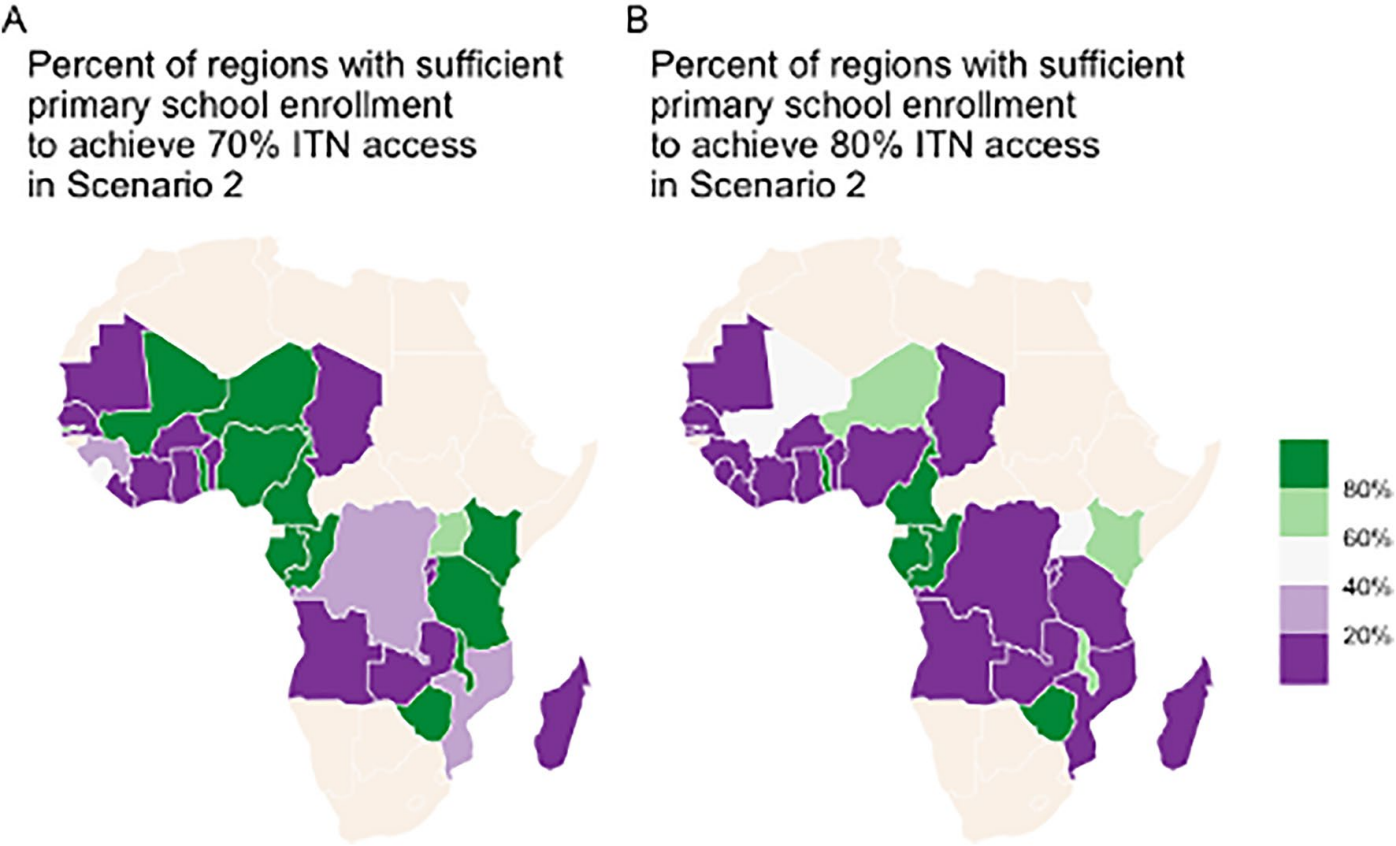
Percentage of regions/provinces per country where large-scale annual school distribution to achieve ITN access targets for **A** 70% and **B** 80% is feasible, assuming one ITN is given per child

To assess feasibility of primary school channels delivering the recommended numbers of nets to households, the proportion of the population currently attending primary school was calculated for each region or province in DHS surveys and compared to the population quantifiers needed to achieve 70% and 80% ITN access targets in Scenario 2. The proportion of regions/provinces within each country where primary school students attending school met or exceeded the population quantifier are shown in Fig. 6, as an indication of the extent within a country where annual school distribution would be feasible. This assumes that only one ITN is given per pupil; for the countries in Fig. 6 with a limited proportion of regions where the primary-school-attending population is large enough, giving more than one ITN per pupil could provide a solution. Alternatively, additional community-based channels could be designed to distribute ITNs alongside school-based distribution.

**Fig. 7:**
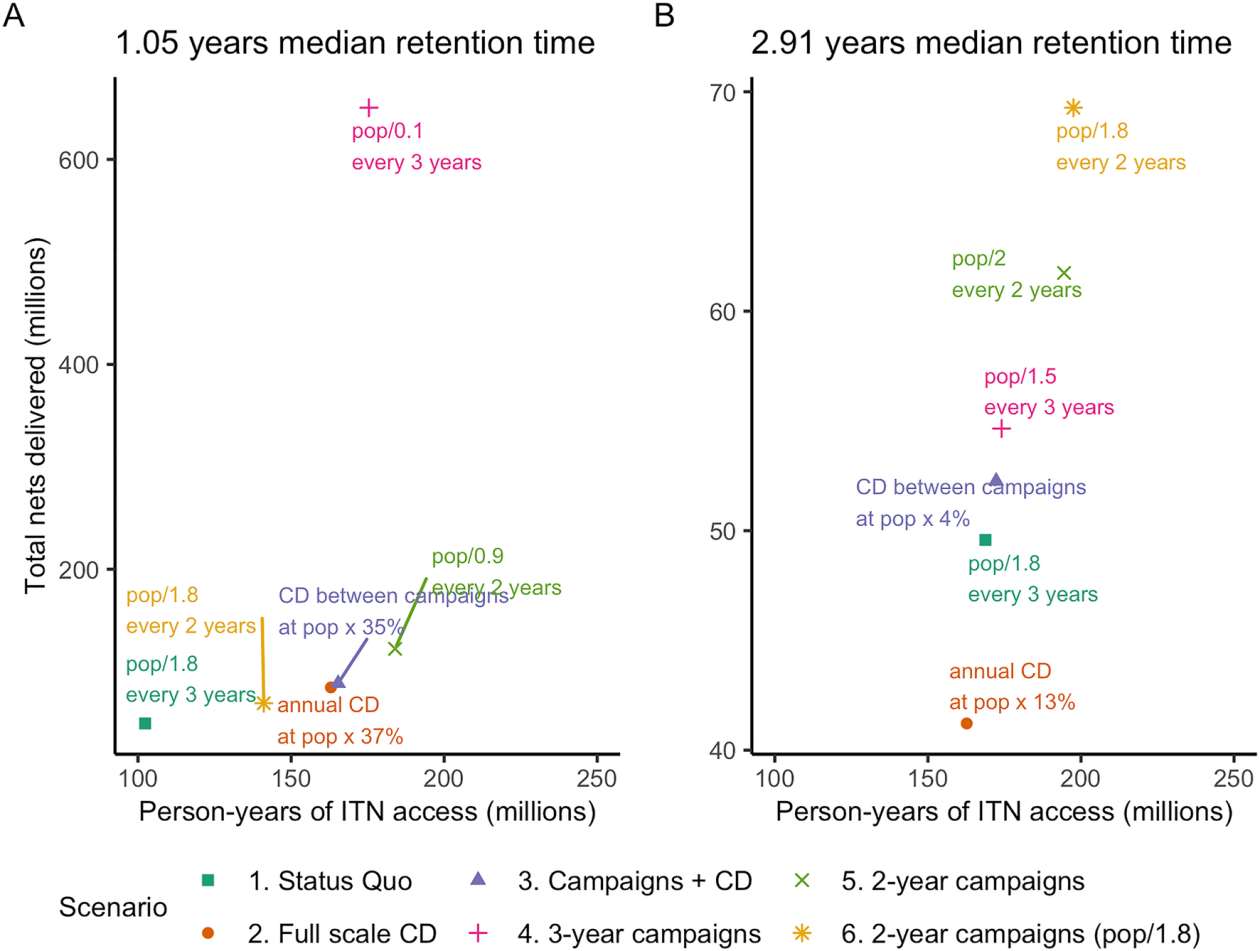
Frontier plots of person-years of ITN access vs total nets delivered, in illustrative populations of 10 million people for comparability, for **A** a country with a median retention time of 1.05 years and **B** a country with a median retention time of 2.91 years. Results for all countries are included in Additional File 3

Finally, person-years of protection, expressed as person-years of ITN access, can be compared to total nets delivered to assess value for money of different ITN distribution strategies and within them, different quantifiers. Figure 7 shows the quantification options within each scenario that provided the maximum person-years of protection, against the total nets required, for two countries representing shorter and longer retention times, over a ten year period. While status quo strategies of three-yearly campaigns quantified using population/1.8 with ANC/EPI at 7% require the fewest ITNs, they also provide the fewest person-years of ITN access. Moving rightward along the x-axis in Fig. 7A, the options provide more person-years of ITN access, each with slightly more total nets required. However, in Fig. 7B, with a nearly three-year retention time, annual CD quantified at population × 13% provides 163 million person-years of ITN access over 10 years, with other options requiring more total nets but providing only minimal, if any, additional protection. Moreover, countries with shorter retention times require far more nets overall in order to provide similar levels of protection compared to countries with longer retention times: a full-scale CD programme in country A achieves less than 163 million person-years of ITN access for around 84.7 million ITNs, compared to country B, where full scale CD provides 162 million person-years of ITN access for around 41.2 million ITNs. Frontier plots for all countries are included in Additional File 3.

## Discussion

This analysis shows that the current status quo of conducting mass campaigns every three years using a population/1.8 quantifier is insufficient to achieve targets of 80% population access to ITNs in the majority of malaria-endemic countries, given ITN retention times are estimated to be less than three years in many settings.

Second, the impulse to conduct mass campaigns every two years in places where nets have an estimated retention time or durability of two years, however intuitive, ultimately requires far more ITNs to maintain 80% population ITN access than under a strategy of continuous distribution. Compared to the current status quo, full-scale continuous distribution of nets could provide continuous ITN access at 80% or higher, with fewer nets compared to current mass campaign approaches in settings where ITN retention times are at least two and a half years. In these countries, between 20-23% fewer ITNs would result in better ITN access compared to current mass campaigns. A strategy of two-year mass campaigns using a quantifier tailored for net retention rates would require 67% more nets than status quo with similar coverage outcomes (Fig. 5).

Third, after examining whether population/1.8 is appropriate in all settings, these findings indicate that in order to maintain population ITN access at target levels of 80%, tailoring of the quantifier to the demographic profile and net durability profile of each country is necessary. As described in Bertozzi-Villa et al. and shown here, the relationship between nets-per-capita and population ITN access is sensitive to average household size. Countries with an average household size below five achieve less ITN access for the same number of nets-per-capita than countries with average household size of five or more. Net retention time is however the major driving factor behind the calculations for the optimal quantifier. Countries with retention times of less than two years could not maintain ITN access at 80% between campaigns even when one ITN per person was delivered in the model, and countries with retention time of around 2.5 years required population/1.5 or population/1.2 to offset the rapid loss of nets post-campaign and still maintain high ITN coverage. However, even these approaches would introduce what most planners and donors would consider a vast “oversupply” of ITNs at each campaign.

It is worth noting that the model assumes that all distributed nets decay at the same rates; that is, nets are not stored for later use. There are few studies reporting what households do with their nets when they are oversupplied. Should households retain extra nets for later use, this would greatly improve rates of ITN access in the model. Conversely, if all oversupplied households immediately diverted extra nets, for example to misuse, net lifetimes would be further reduced and ITN access much lower than the estimates presented here. Given that “saving for later” is a key reason why nets go unused in households that own “too many” [20], the reality may be closer to the former case, but results are likely between these two extremes.

Fourthly, optimal quantifiers are presented here for a variety of ITN distribution strategies, along with the relative number of nets that would be required, in an effort to facilitate decision making for national programmes and their partners.

The global malaria community faces significant challenges, with limited funding to implement life-saving interventions potentially the greatest immediate threat to malaria control efforts. Countries are advised to generate subnational strategies to tailor intervention packages to specific settings, but resources are inadequate to implement the desired combinations of interventions that would have the greatest impact. National programmes thus face difficult choices about which population segments to cover fully, and which to leave with suboptimal protection. These recommendations to “do more with less” are not tenable. Insufficient vector control coverage is a key driver of malaria transmission; malaria cases have been shown to decrease for only a year or two between campaigns before rising again in several observational studies [21–24] and in model outputs [25]. The challenges of delivering more ITNs are clear: limited funding for both the nets and their distribution costs; diminishing donor appetite; and the additional cost of new types of ITNs recommended for areas with insecticide resistance. The implementation of each of these types of distribution strategies also comes with unique challenges, which are beyond the scope of this paper. However, the costs of continuing to deliver insufficient quantities of ITNs and providing only intermittent protection are also clear: continued loss of life and disability, school and work absenteeism, and decreased quality of life.

There is a growing call to improve the retention time and/or durability of ITNs through improved physical specifications for ITNs and through improvements in household-level behaviours related to net care. Improvements in both areas would undoubtedly contribute towards increasing retention times, but it is unlikely that this would fully offset the general and continued challenges of keeping nets in good condition for long periods of time across settings in malaria-endemic areas. Net durability is driven by behavioural factors in part, but these relatively fragile products are subject to numerous daily stresses in most household environments [26]. This is particularly true for people living through humanitarian emergencies, and this population is projected to increase due to climate change and climate-related emergencies, among other factors.

All of these results hinge on the estimated retention times. It is important to highlight that ITN durability studies typically observe longer median lifespans compared to the MAP-estimated ITN retention times. In several cases, the divergence is striking. Most notably, Liberia’s retention time was estimated at 1.0 years, but an ITN durability study completed in 2021 in two counties observed a median survival in serviceable condition of four years [27]. Countries like Liberia with divergent data for net lifespans need to weigh these results carefully. Likewise, net retention times and durability have been demonstrated to vary subnationally; durability monitoring of ITNs in northern Nigeria showed median lifespans of over five years, while the same product monitored simultaneously in southern zones of Nigeria had a median lifespan of just over three years [12], consistent with findings from an earlier cross-sectional durability study in similar areas of Nigeria [13]. Programmes must consider potential differences in net retention behaviour and net durability as they weigh their quantification decisions, and plan for additional data collection where information is not available or insufficient.

Two countries currently implement large-scale continuous distribution: Ghana distributes ITNs to school children in grades two and six between three-year campaigns, while Tanzania has implemented full-scale school distribution in fourteen mainland regions since 2016, and expanded the programme to five additional regions in 2022. The quantities of ITNs distributed to school children in Ghana amount to 4.4% of the population (1.4 million ITNs in the 2020 school distribution)—far from the 20% estimated to be needed to maintain ITN access at levels of 80%. Tanzania’s school net programme (SNP) has delivered ITNs equivalent to 12–16% of the population in SNP zones over recent years, but they may require quantification of population × 21% to maintain ITN access at 80%, discussed in detail elsewhere [7].

School distribution, conducted annually to all or selected primary school classes, is a promising approach to delivering ITNs [5, 7, 28, 29]. Studies estimate that school-age children drive at least 60% of malaria transmission [30, 31] due in part to their lower rates of ITN use, especially when households do not own enough nets [31, 32]. Ensuring that these children—and their family members—are prioritized for protection with ITNs, along with vulnerable pregnant women and children, is a hallmark of the school distribution strategy. Annualized distributions also facilitate planning, avoiding the three-yearly overload of mass campaign planning among national malaria programmes and their implementing partners, and the platform leverages Ministry of Education personnel, providing opportunities for additional integration of other school health interventions.

This analysis uses a target of 80% population ITN access, but it must be noted that for ITN use levels to reach 80%, ITN access must be at least 90%, given the persistent yet small gap between ITN access and ITN use [2, 16, 33]. Targeting ITN access at 80% means that ITN use will rarely, if ever, exceed 70%. Donors and programmes must, therefore, evaluate what target levels of ITN use are necessary for success, and adjust ITN access targets upwards accordingly.

## Limitations

The analysis is faced with several limitations. First, the estimated retention times for certain countries are based on a limited number of surveys, described further in Bertozzi-Villa et al*.* [2]. The uncertainty of these estimates is accounted for in the calculations of ITN access. Second, localized durability monitoring studies sometimes find significantly longer median survival of ITNs than the retention time estimates, which could indicate subnational differences in ITN longevity or be evidence of analytical challenges in the ITN retention times or conversely, Hawthorne effect in the durability monitoring studies, leading households to retain their nets longer when they expect to be visited by study teams. Third, the relationship between nets-per-capita and ITN access is assumed to be consistent regardless of ITN distribution strategy, but it is likely that it would be influenced by oversaturation of ITNs in certain types of households, as occurs with school distribution of ITNs. It will be important to explore this relationship further as additional data become available. Fourth, the performance of ANC and EPI channels in the models presented here (reaching 6% rather than the estimated 8–9% of targeted populations) reflect relatively robust health systems with high ANC1 and vaccination attendance and reasonable efficiency at reaching these populations with ITNs. Many countries may not currently reach this level of performance, particularly countries with challenging supply chain and reordering systems, and the few remaining countries that deliver ITNs to pregnant women but not to infants.

Finally, retention behaviour is influenced by net availability (or lack thereof); with increasing availability of nets, households could be disincentivized to take care of their nets. Programmes must be mindful of these types of perverse incentives that may drive up the number of nets required unnecessarily.

## Conclusion

Given variation in ITN retention times across countries, tailored quantification approaches for mass campaigns and continuous distribution strategies are warranted. To reach target levels of ITN use of 80% of the population, ITN access must be maintained near 90% in most settings. The quantity of ITNs required to meet these goals is substantially larger than current plans, but these findings indicate that full-scale continuous/annual distributions are likely to provide the most efficient mechanism to maintain higher ITN access, as they require fewer ITNs than tailored campaign strategies that maintain similar levels of ITN access. Moreover, in countries with ITN retention times of at least two and a half years, full scale continuous distribution strategies are estimated to require up to 20-23% fewer ITNs to achieve better ITN access compared to current mass campaigns. These findings are especially pertinent in the context of new, more expensive ITNs, requiring careful consideration of ITN needs in light of varied products and prices. National programmes and their funding partners should work to increase the availability of ITNs to those vulnerable to malaria, while at the same time working to extend the useful life of these critical commodities.

## Supplementary Information

**Additional file 1: **Estimated ITN access under five ITN distributions scenarios and at varying quantification approaches.

**Additional file 2: Table S1.** Recommended annual quantifiers for continuous distribution channels. All scenarios assume that ANC and EPI delivery of ITNs is ongoing and provides nets to 6% of the population. However, quantifiers listed in the table represent only the continuous distribution channel, e.g. Angola would require both ANC/EPI distribution as well as continuous distribution quantified using population x 27% to maintain ITN access at levels of 70%. **Table S2.** Lowest level of ITN access between three-year campaigns at different population quantifiers for all countries. Green color reflects higher ITN access while red indicates lower ITN access reached between campaigns. Routine ITN delivery to pregnant women and infants is assumed. **Table S3.** Lowest level of ITN access between two-year campaigns at different population quantifiers for all countries. Green color reflects higher ITN access while red indicates lower ITN access reached between campaigns. Values of 100 indicate excess nets in the system. Routine ITN delivery to pregnant women and infants is assumed. **Table S4.** Summary of recommended quantifiers for all countries and scenarios, to maintain ITN access at or above 80%. The quantifiers are for only the continuous distribution channel or mass campaign; annual ANC/EPI distribution equivalent to 6% of the population is assumed in each scenario, but is not part of the listed quantifiers. Countries are listed by ISO3 code.

**Additional file 3: **Frontier plots of Person-years of ITN access vs Total nets delivered.

## Data Availability

Code is available at https://github.com/hkoenker/Quantification.
