# Supplementary material for: How many mosquito nets are needed to maintain universal coverage: an update
Source: Malar J. 2023 Jun 30;22:200. doi: 10.1186/s12936-023-04609-z (PMC10314435; doi:10.1186/s12936-023-04609-z)

Supplementary File 1: Estimated ITN access under different distribution strategy scenarios and quantification factors

Hannah Koenker

05 June, 2023

Contents

1 Scenario 1 - Mass campaigns every three years with varying routine ANC/EPI distribution 2

2 Scenario 2 - ANC/EPI distribution at 6%, varying annual school/community distribution 5

3 Scenario 3 - Three-year mass campaigns with ANC/EPI distribution at 6%, varying annual school/community distribution between campaigns 56

4 Scenario 4 - Three-year mass campaigns with ANC/EPI distribution at 6%, varying campaign quantifier 97

5 Scenario 5 - Two-year mass campaigns with ANC/EPI distribution at 6%, varying campaign quantifier 117

6 Scenario 6 - Two-year mass campaigns with varying ANC/EPI distribution 133

1 Scenario 1 - Mass campaigns every three years with varying routine ANC/EPI distribution

3−year mass campaigns with ANC/EPI at 5 % of the population

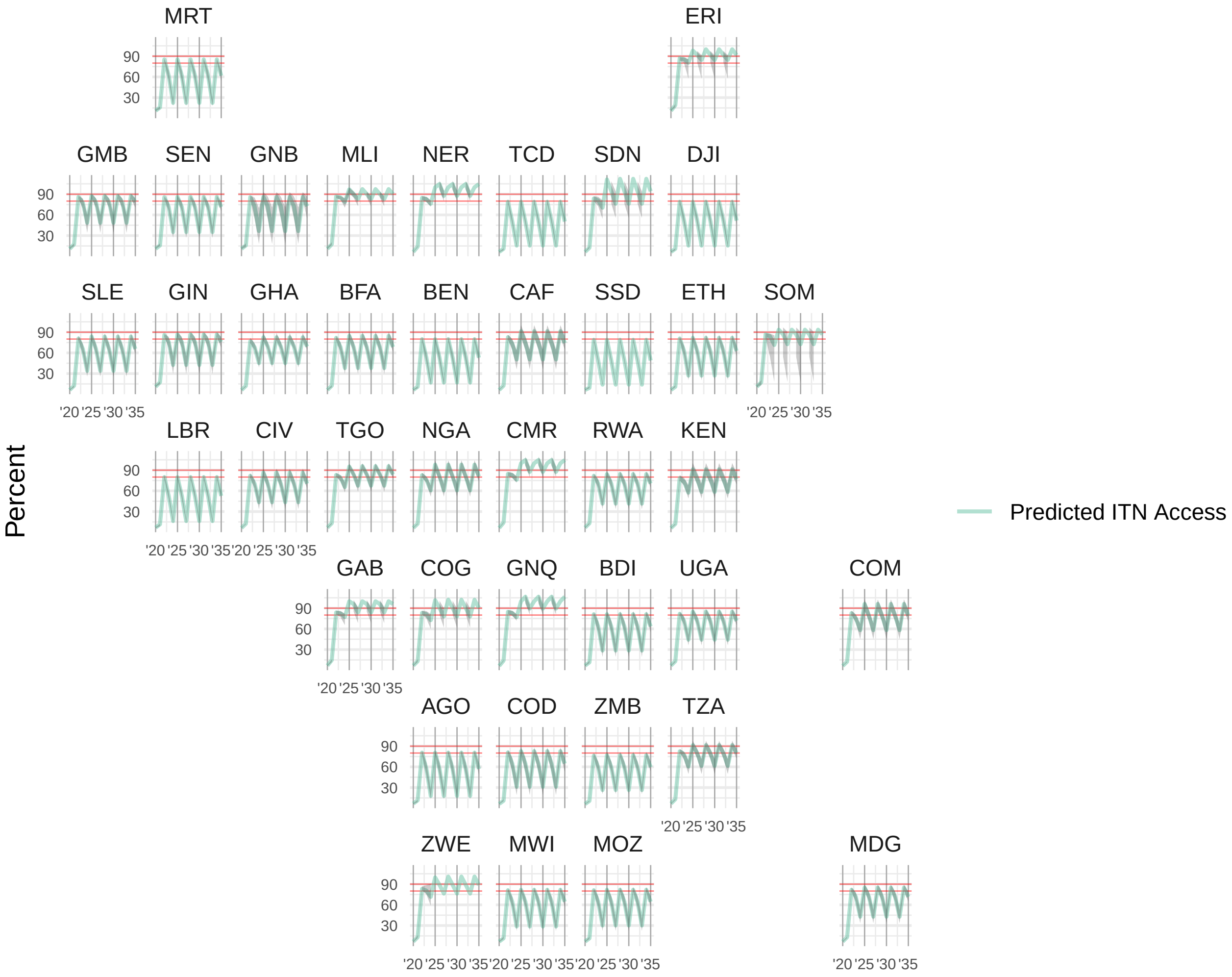

3−year mass campaigns with ANC/EPI at 6 % of the population

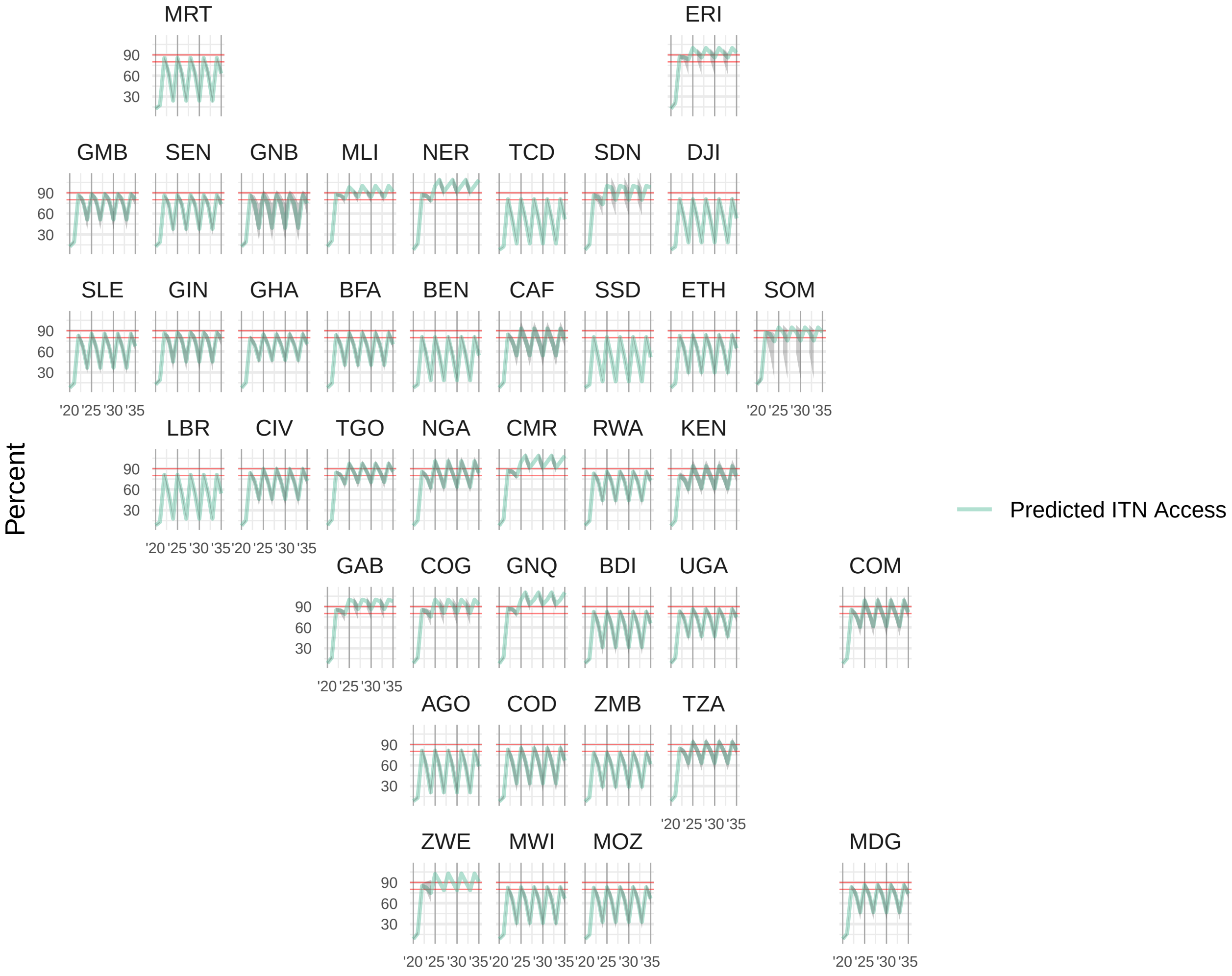

3−year mass campaigns with ANC/EPI at 7 % of the population

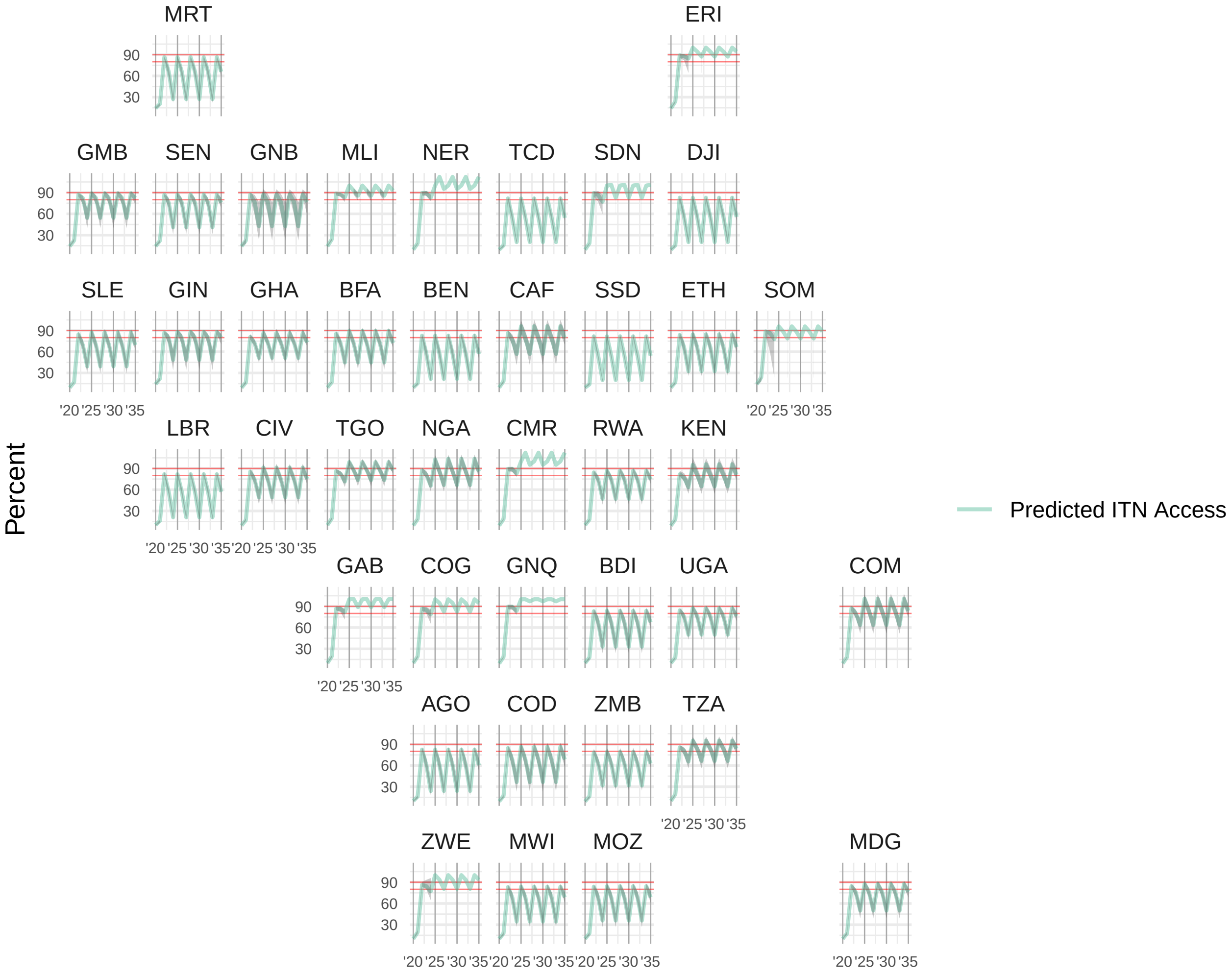

2 Scenario 2 - ANC/EPI distribution at 6%, varying annual school/community distribution

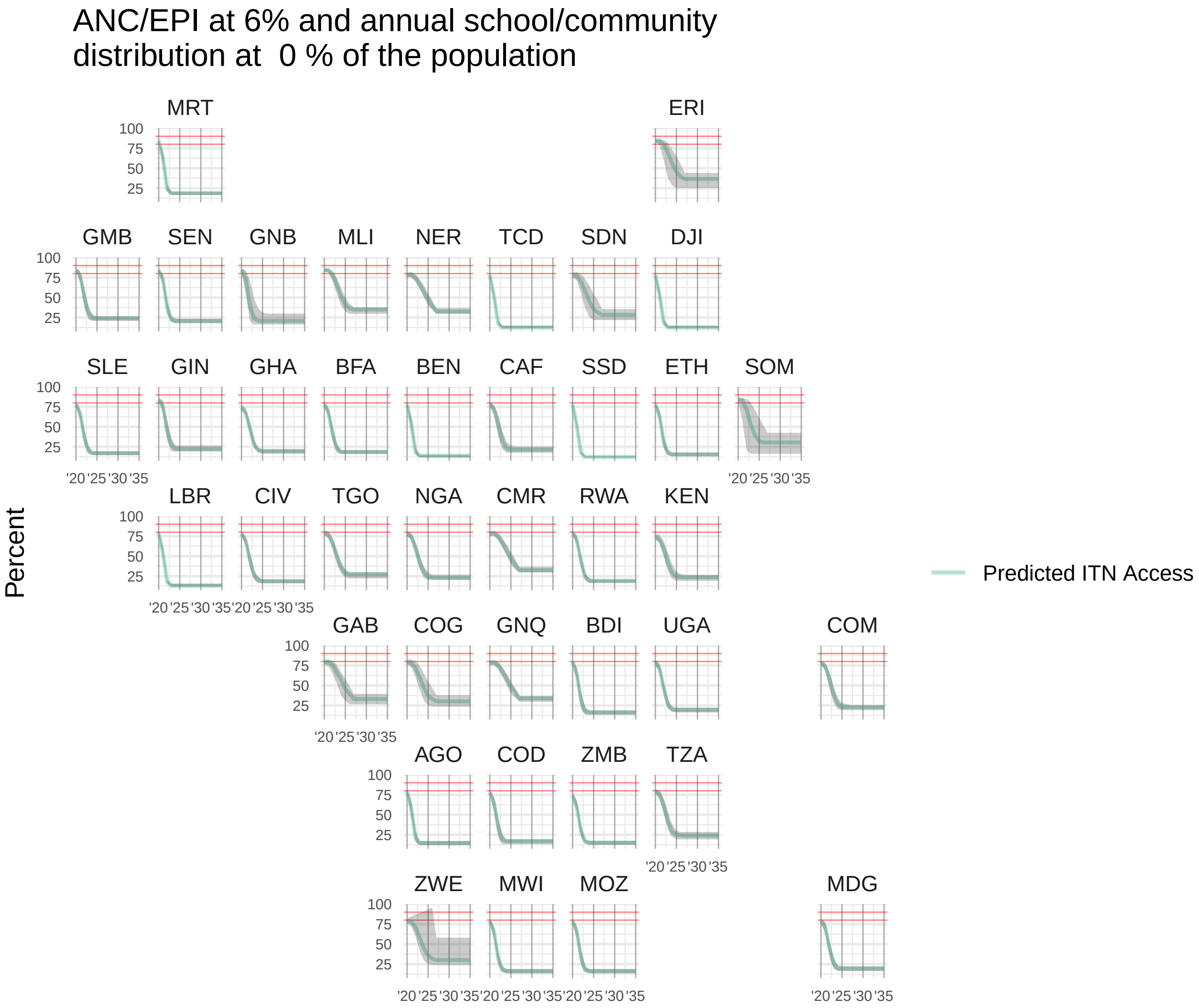

ANC/EPI at 6% and annual school/community distribution at 1 % of the population

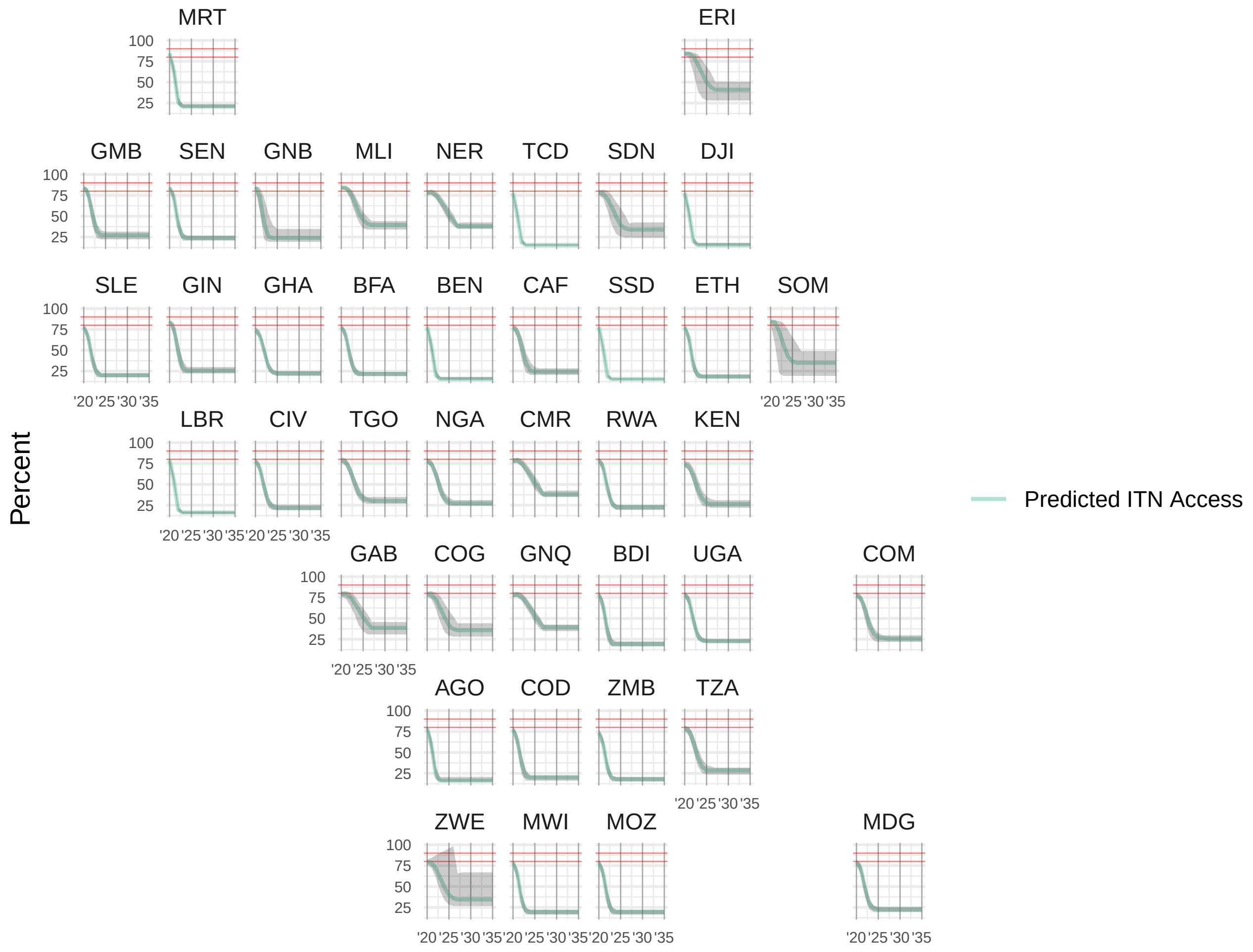

ANC/EPI at 6% and annual school/community distribution at 2 % of the population

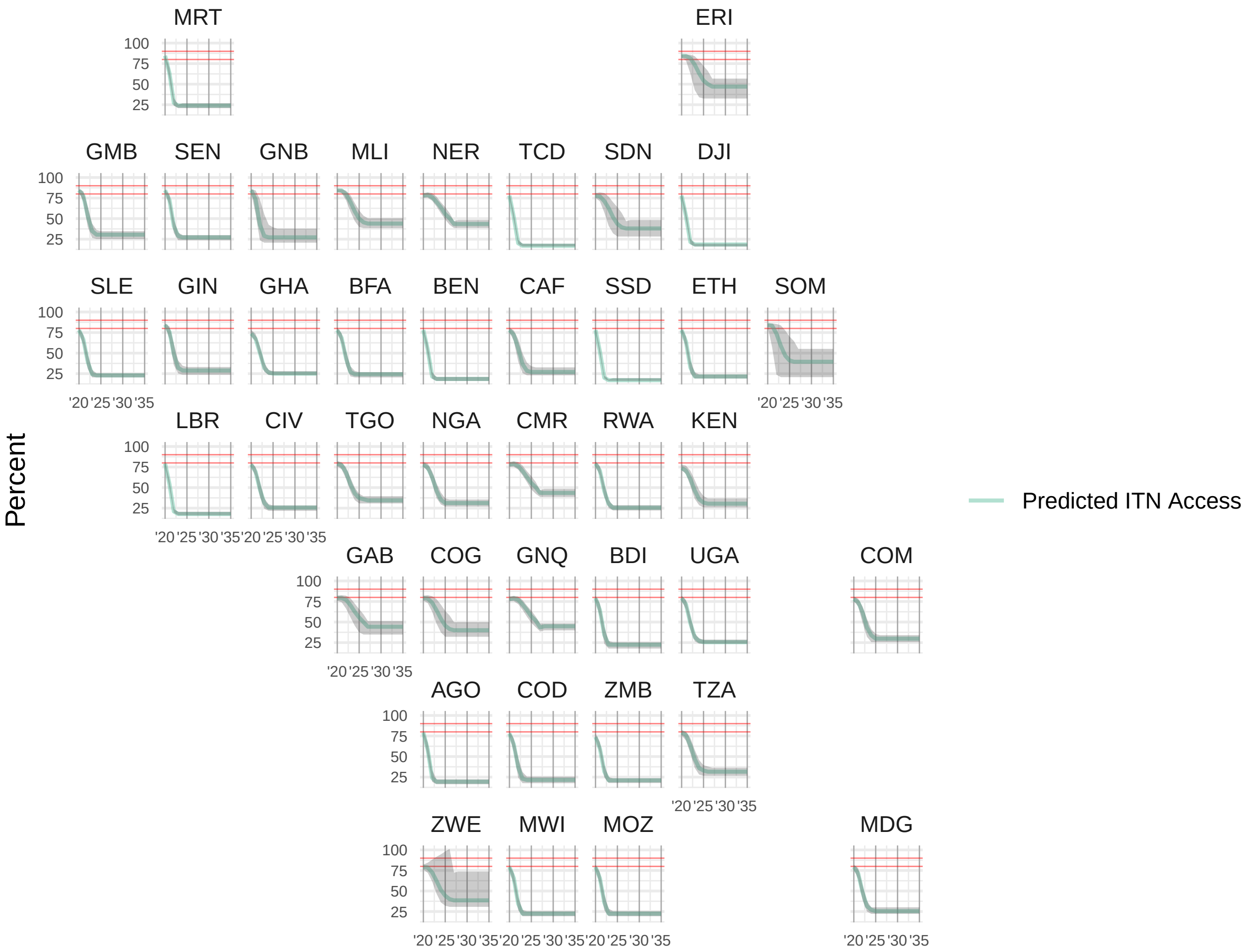

ANC/EPI at 6% and annual school/community distribution at 3 % of the population

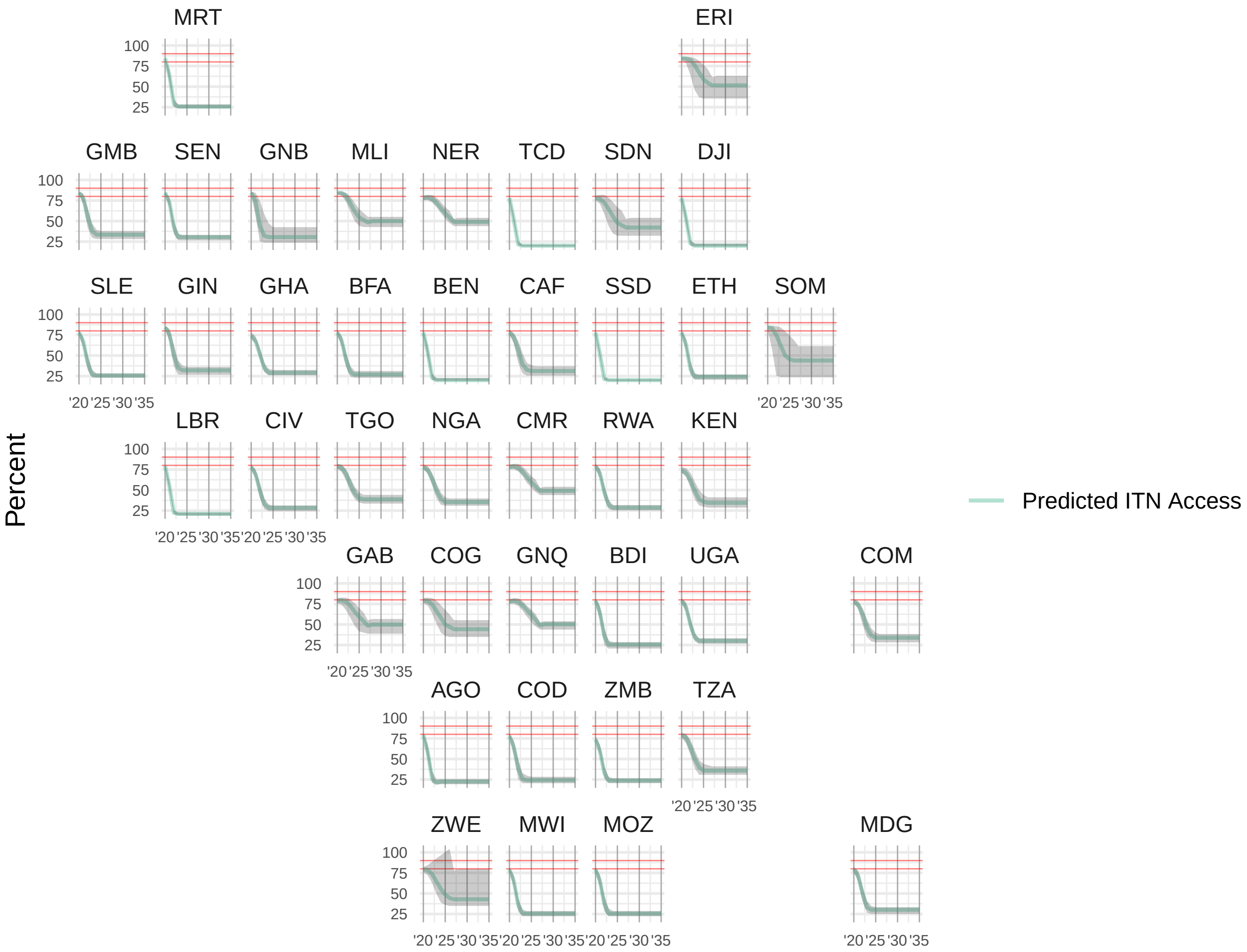

ANC/EPI at 6% and annual school/community distribution at 4 % of the population

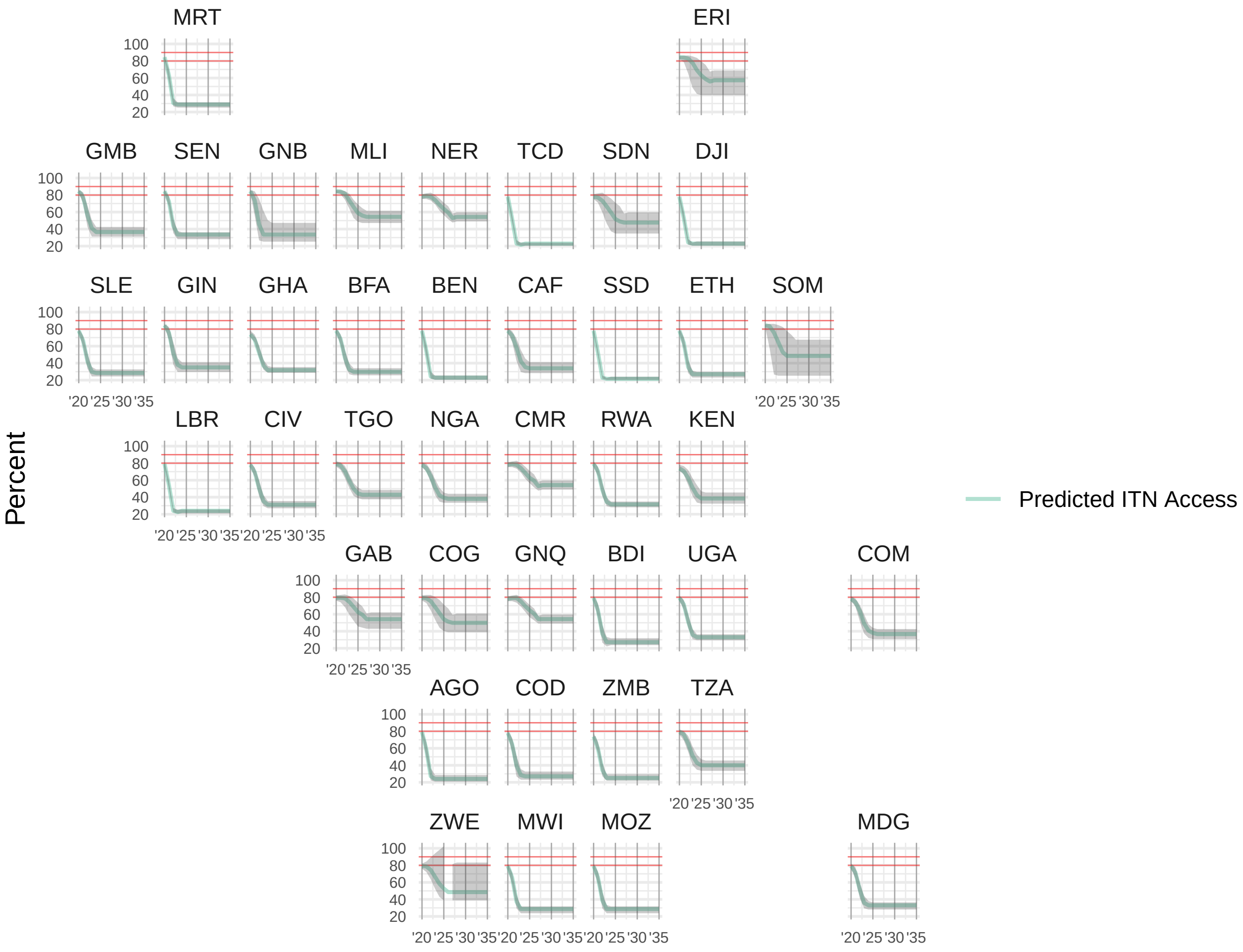

ANC/EPI at 6% and annual school/community distribution at 5 % of the population

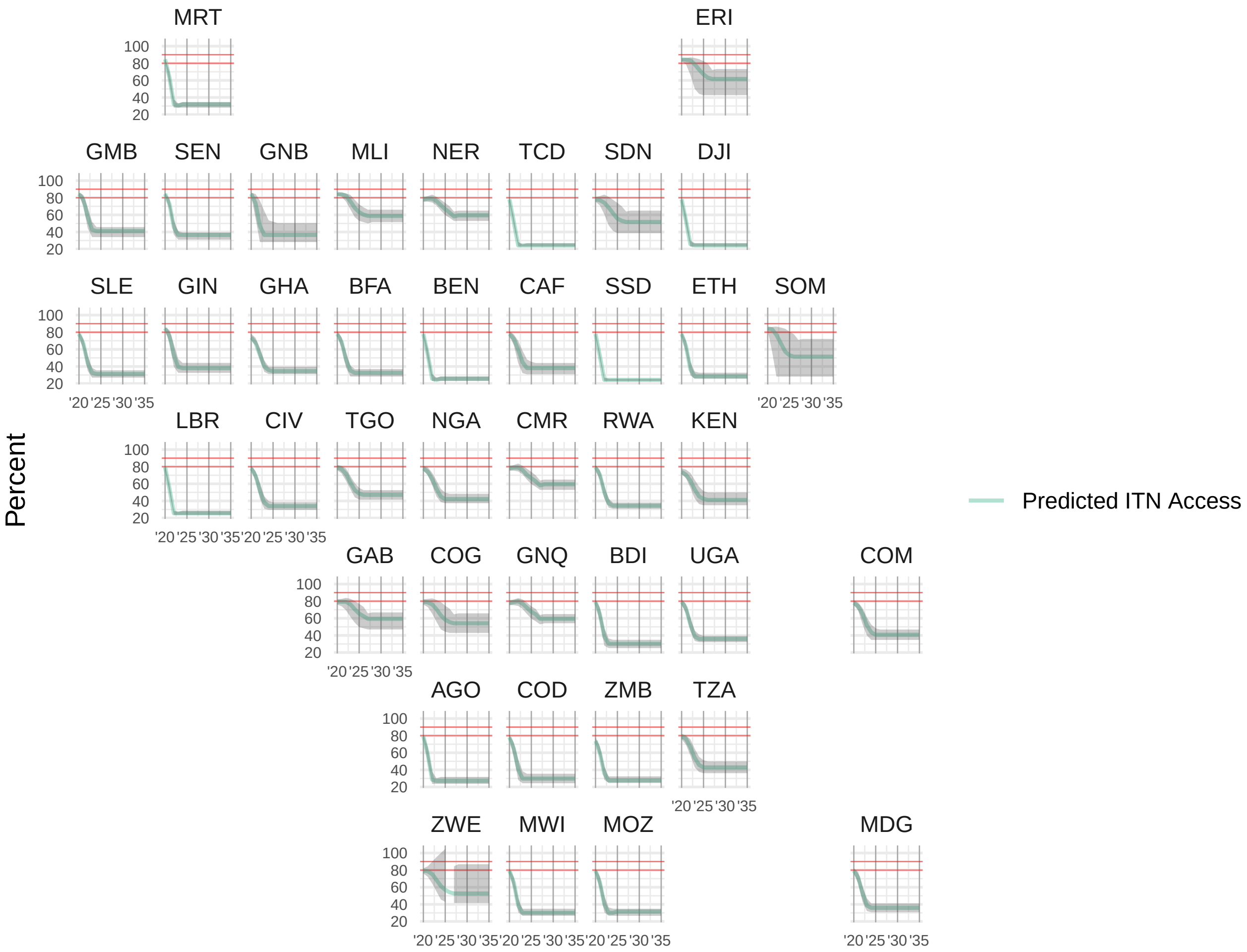

ANC/EPI at 6% and annual school/community distribution at 6 % of the population

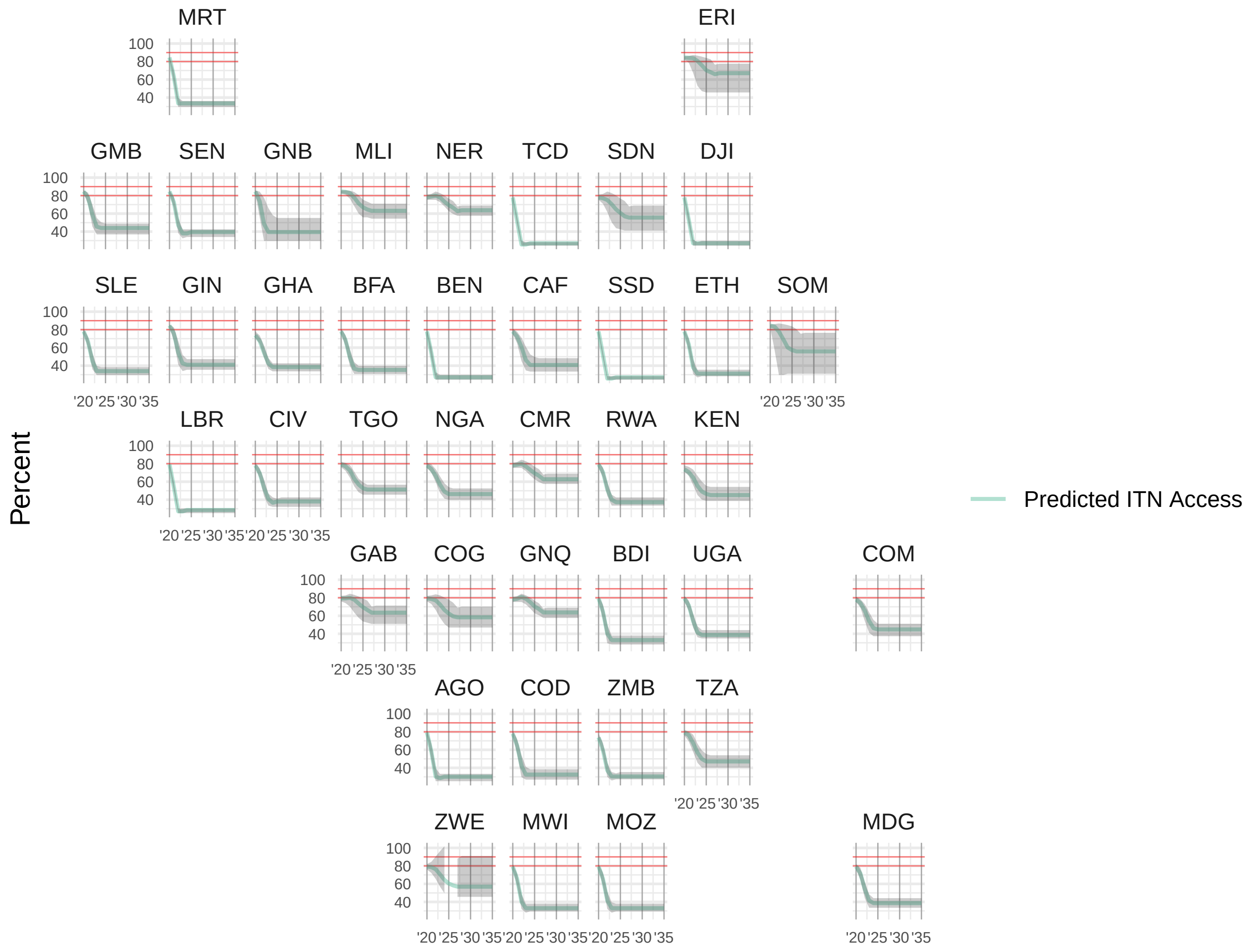

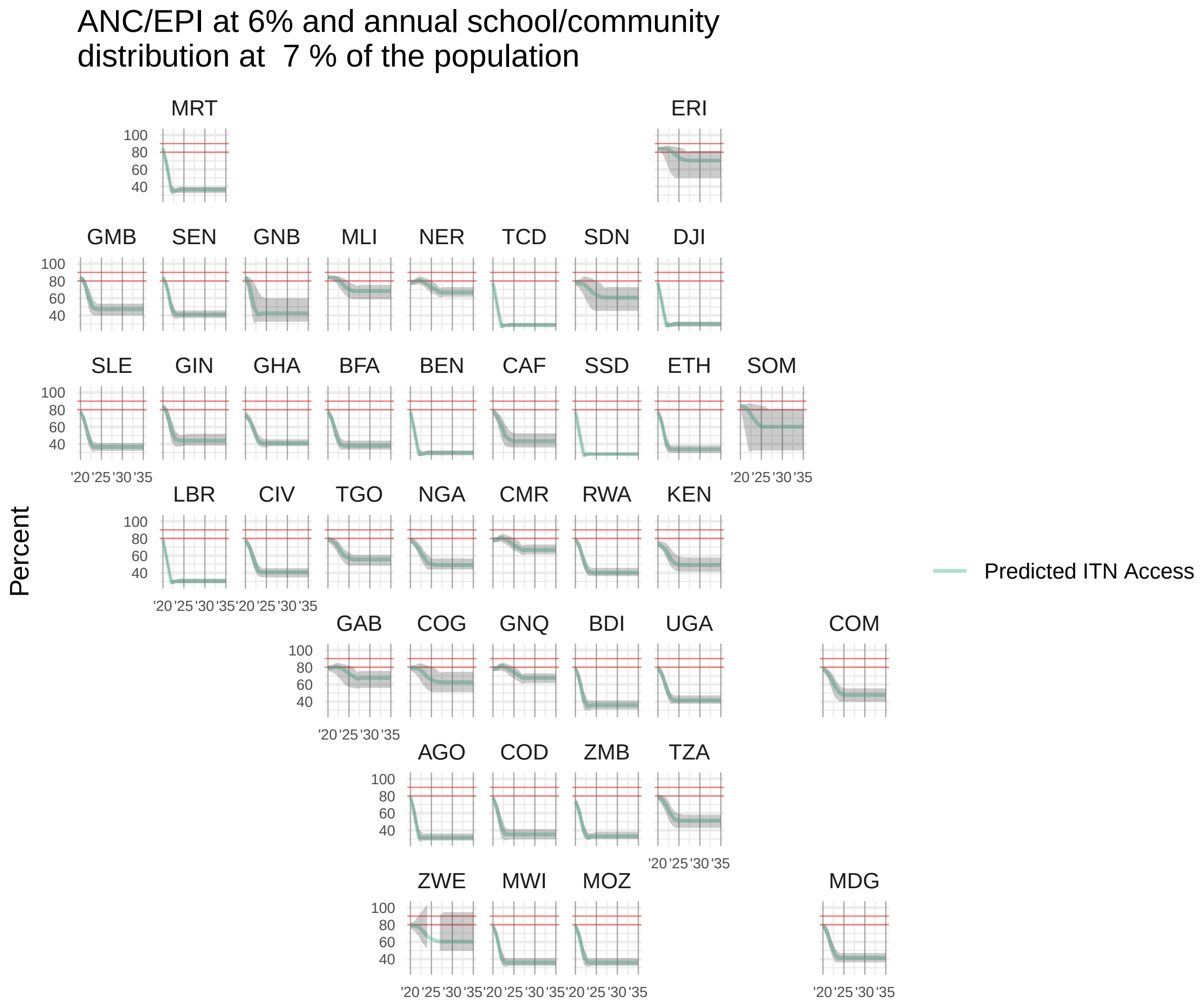
ANC/EPI at 6% and annual school/community distribution at 7 % of the population
MRT
ERI
GMB
SEN
GNB
MLI
NER
TCD
SDN
DJI
SLE
GIN
GHA
BFA
BEN
CAF
SSD
ETH
SOM
LBR
CIV
TGO
NGA
CMR
RWA
KEN
GAB
COG
GNQ
BDI
UGA
COM
AGO
COD
ZMB
TZA
ZWE
MWI
MOZ
MDG
Percent
100
80
60
40
'20 '25 '30 '35
Predicted ITN Access

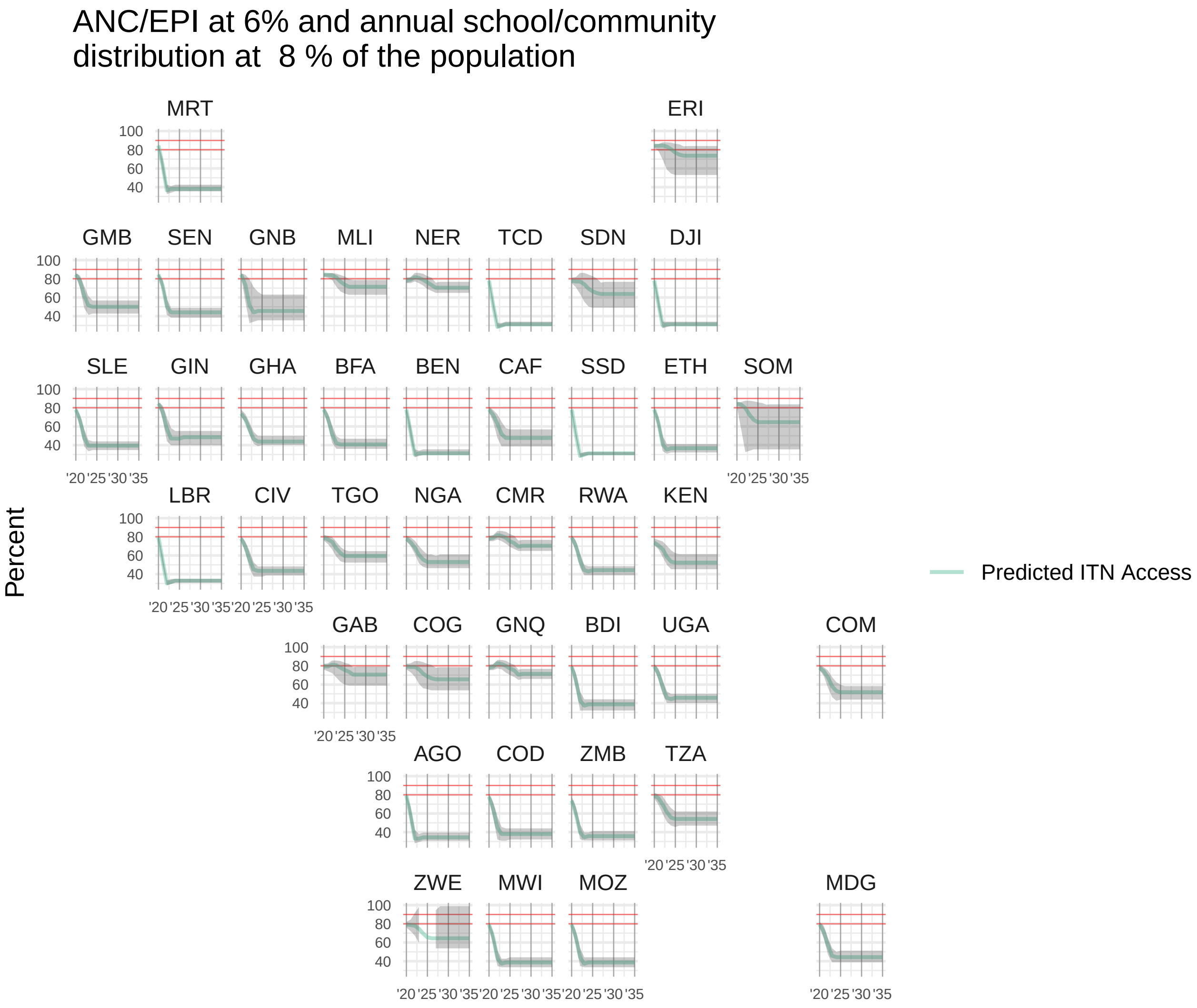
ANC/EPI at 6% and annual school/community
distribution at 8 % of the population
Percent
MRT
ERI
GMB
SEN
GNB
MLI
NER
TCD
SDN
DJI
SLE
GIN
GHA
BFA
BEN
CAF
SSD
ETH
SOM
LBR
CIV
TGO
NGA
CMR
RWA
KEN
GAB
COG
GNQ
BDI
UGA
COM
AGO
COD
ZMB
TZA
ZWE
MWI
MOZ
MDG
100
80
60
40
'20 '25 '30 '35
Predicted ITN Access

ANC/EPI at 6% and annual school/community distribution at 9 % of the population

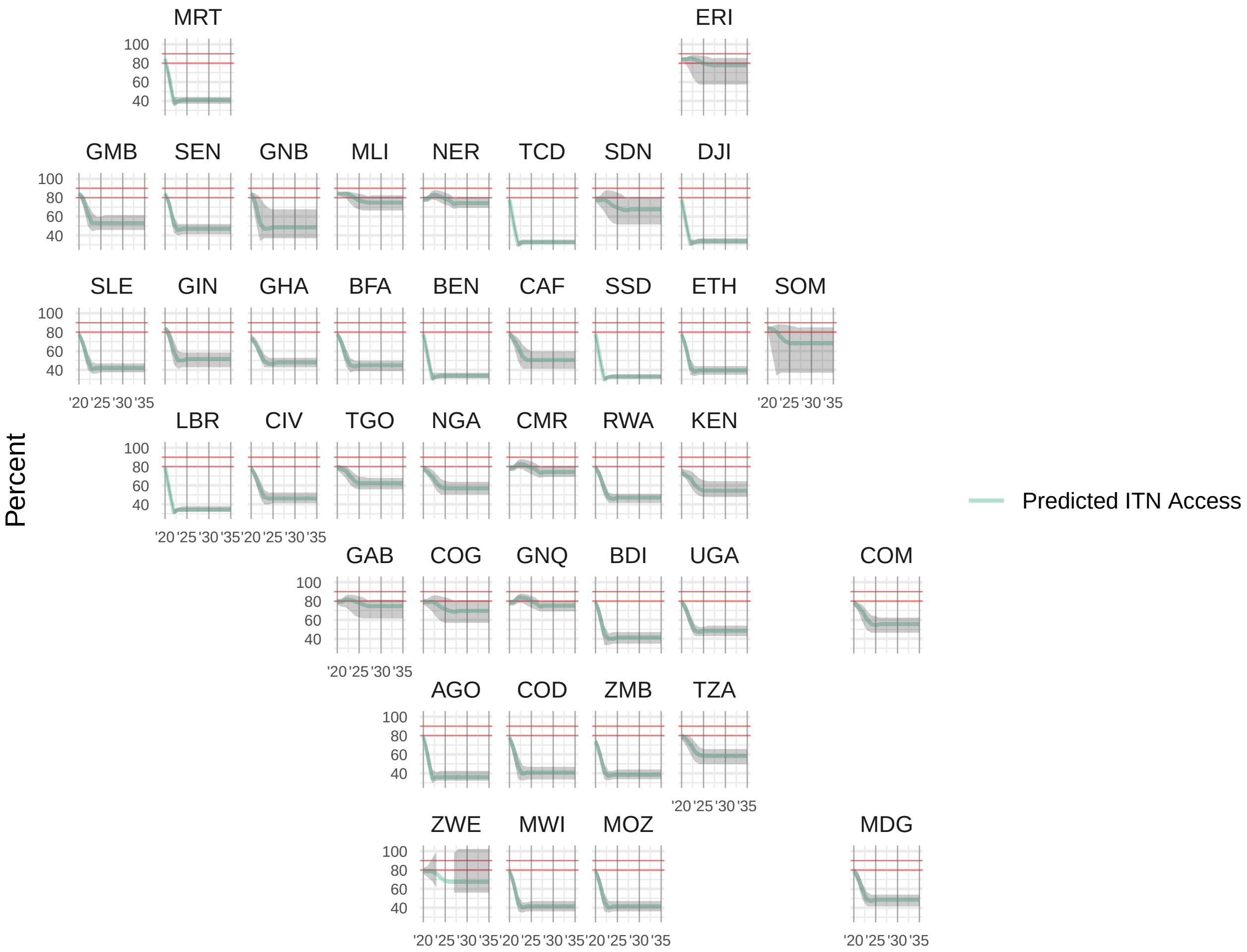

ANC/EPI at 6% and annual school/community distribution at 10 % of the population

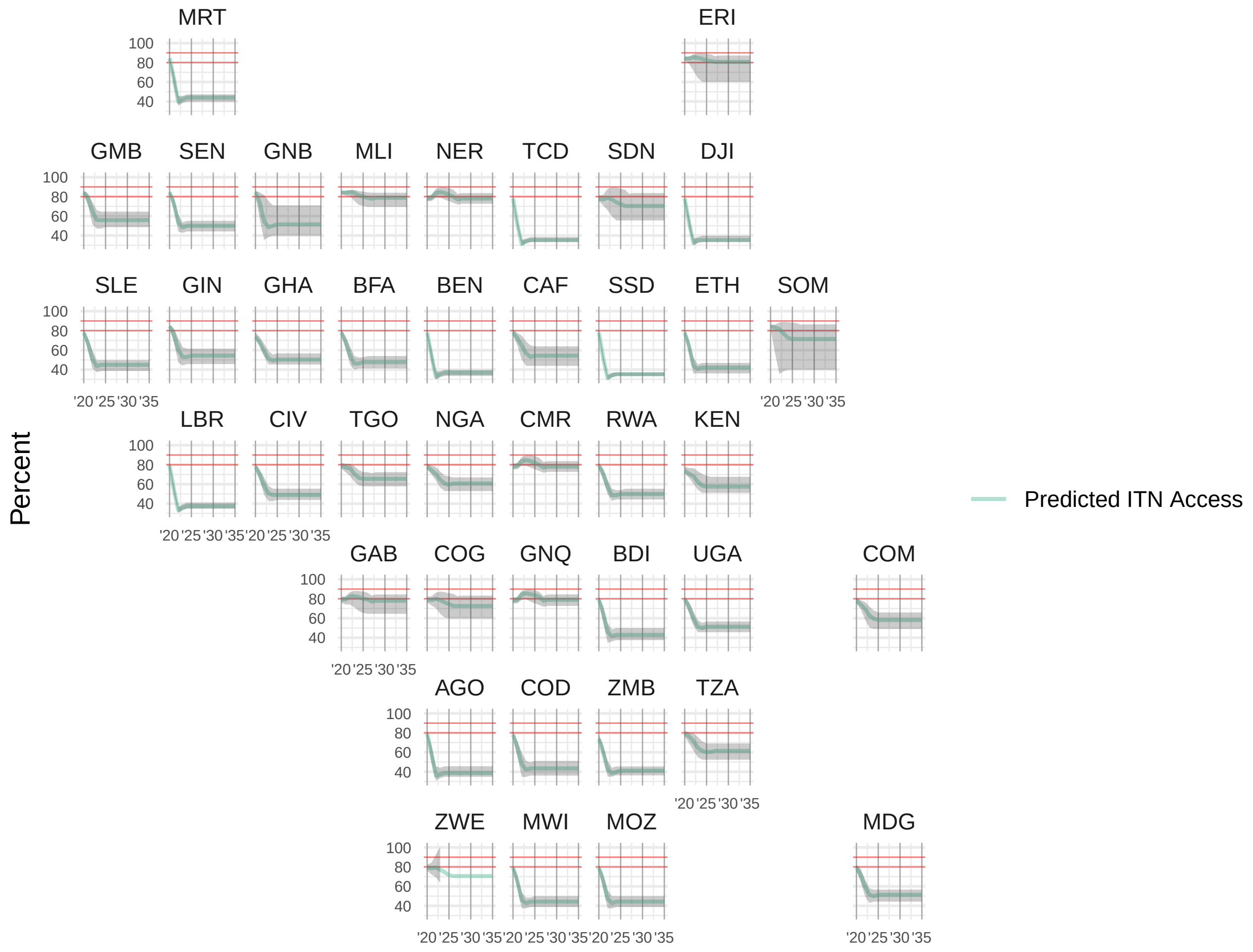

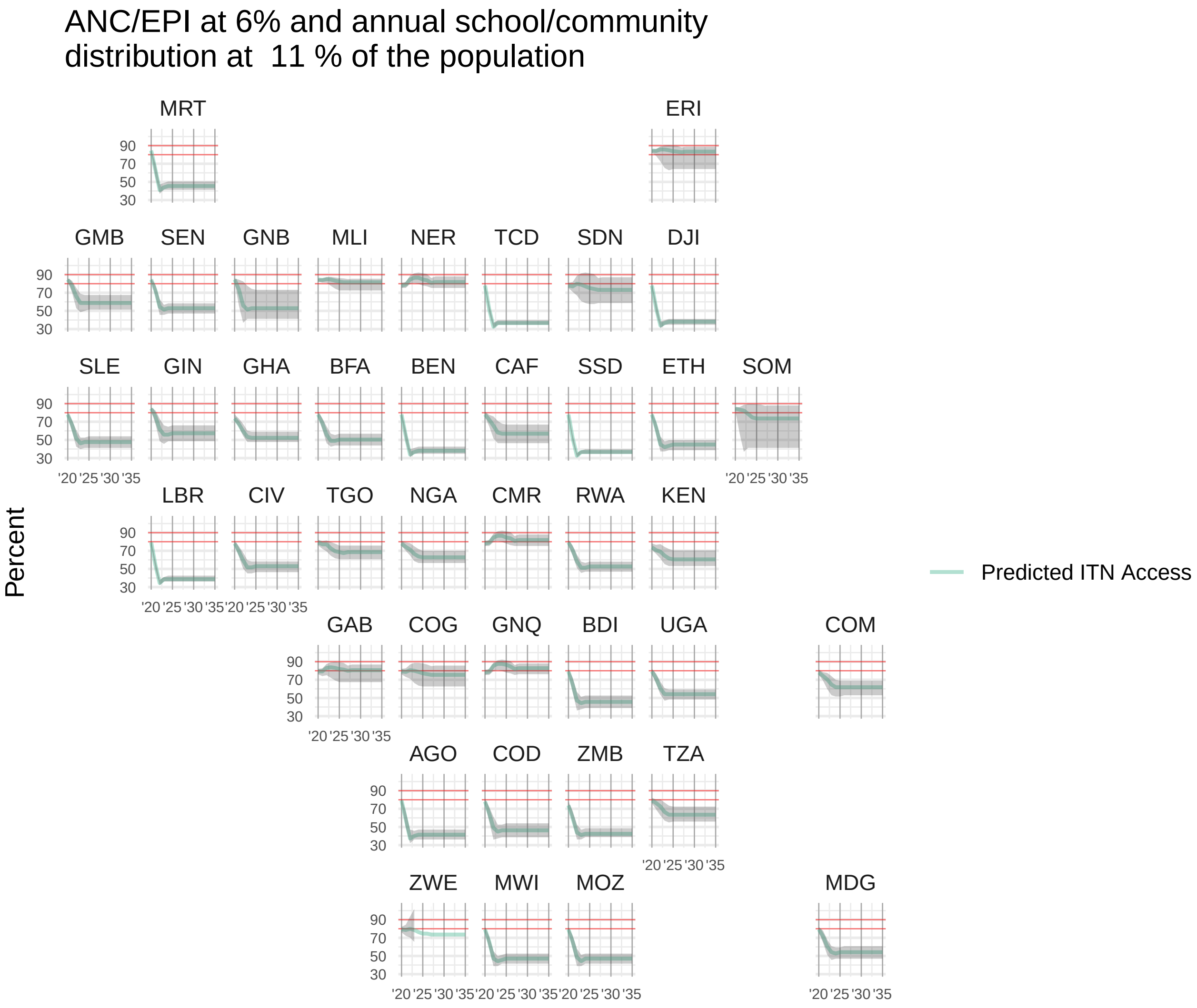
ANC/EPI at 6% and annual school/community distribution at 11 % of the population
MRT
ERI
GMB
SEN
GNB
MLI
NER
TCD
SDN
DJI
SLE
GIN
GHA
BFA
BEN
CAF
SSD
ETH
SOM
LBR
CIV
TGO
NGA
CMR
RWA
KEN
GAB
COG
GNQ
BDI
UGA
COM
AGO
COD
ZMB
TZA
ZWE
MWI
MOZ
MDG
Percent
90
70
50
30
'20 '25 '30 '35
Predicted ITN Access

ANC/EPI at 6% and annual school/community distribution at 12 % of the population

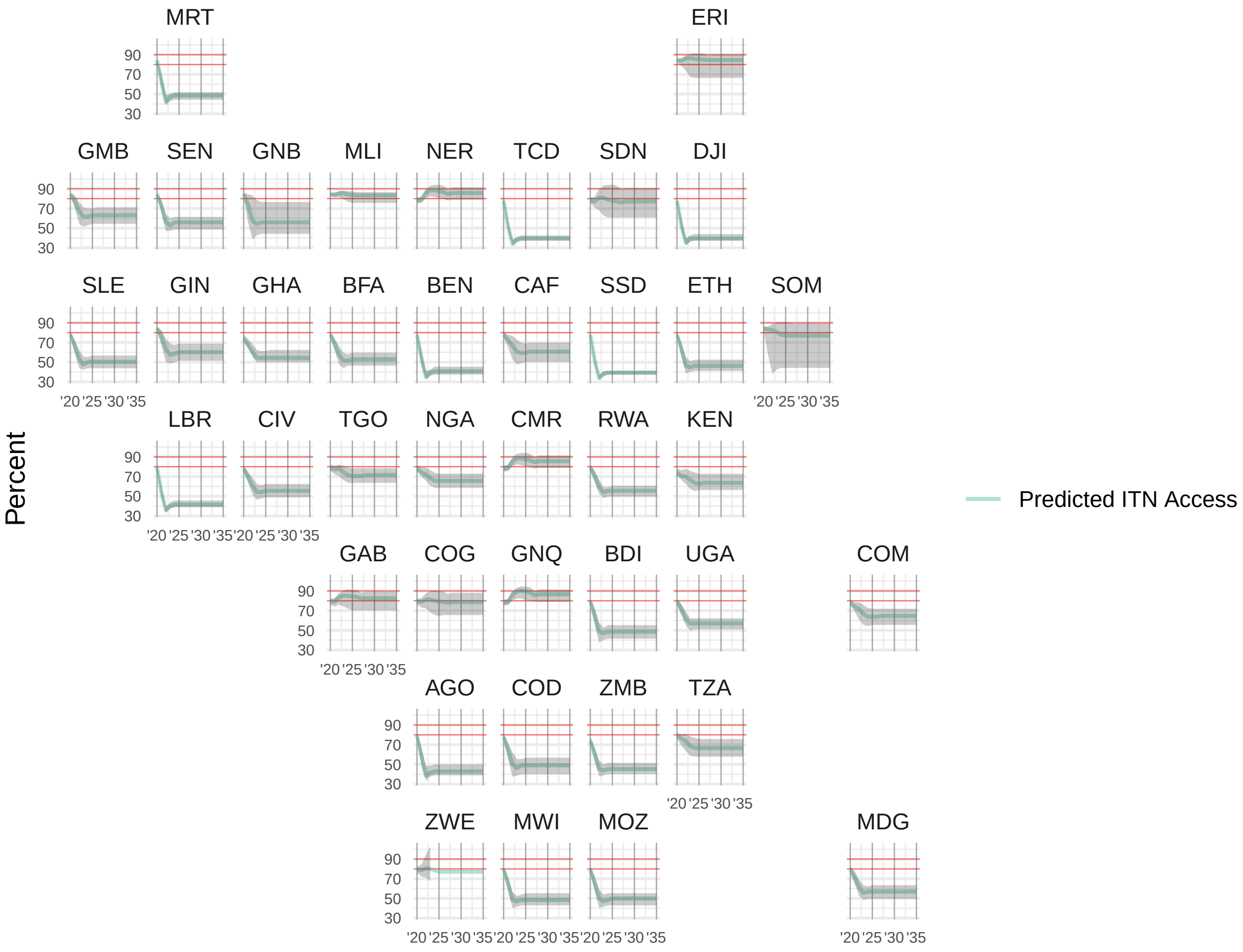

ANC/EPI at 6% and annual school/community distribution at 13 % of the population

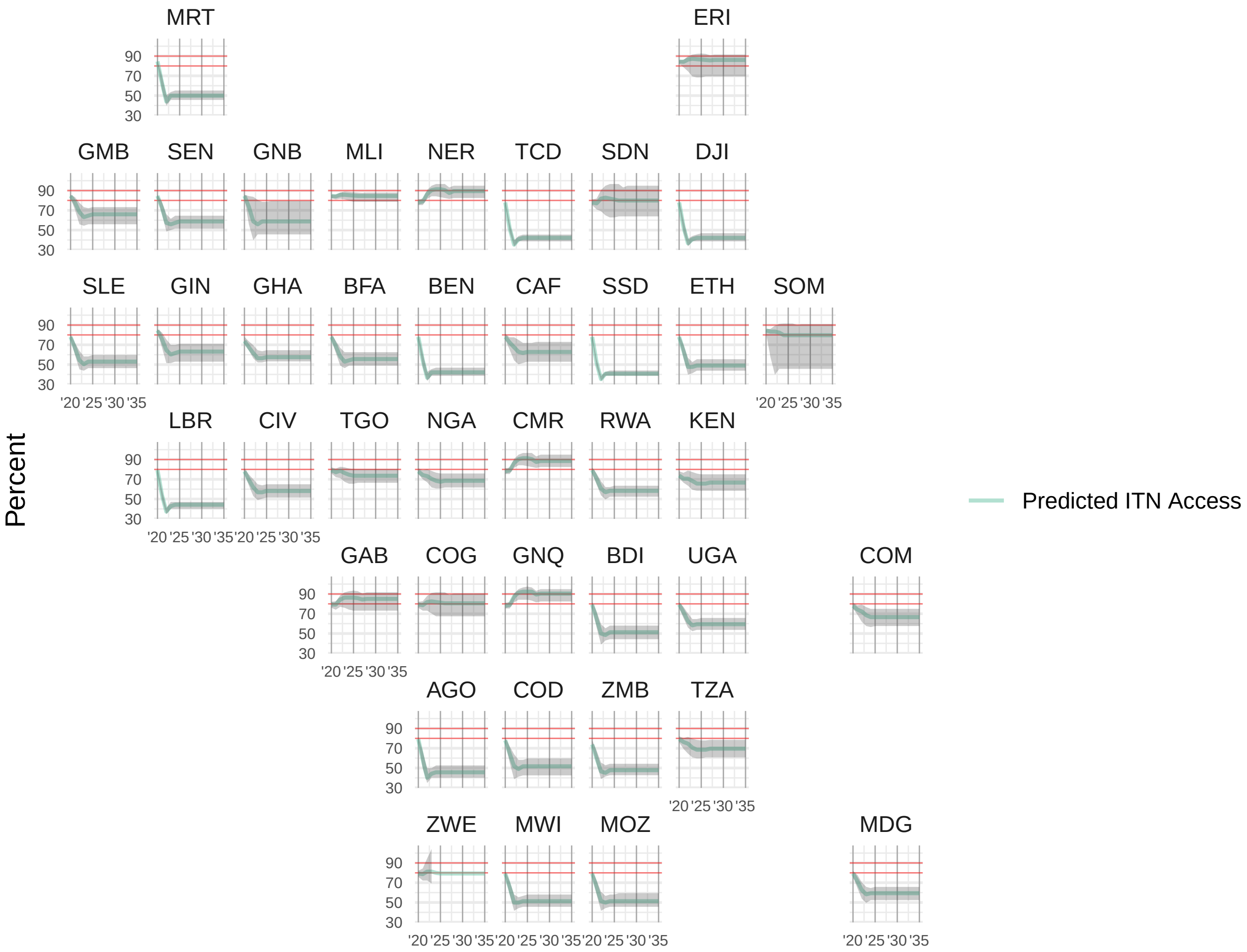

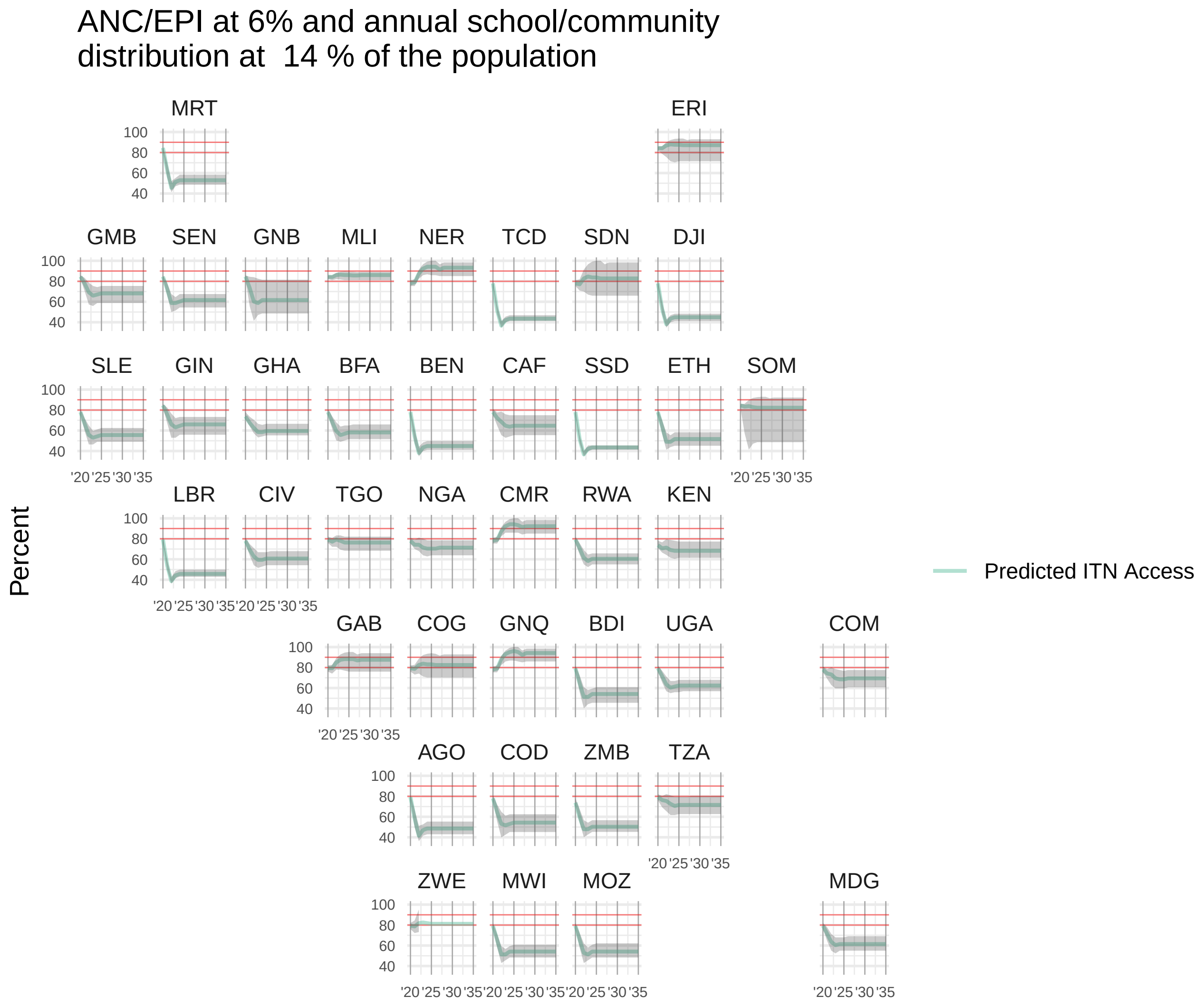
ANC/EPI at 6% and annual school/community
distribution at 14 % of the population
Percent
Predicted ITN Access
MRT
ERI
GMB
SEN
GNB
MLI
NER
TCD
SDN
DJI
SLE
GIN
GHA
BFA
BEN
CAF
SSD
ETH
SOM
LBR
CIV
TGO
NGA
CMR
RWA
KEN
GAB
COG
GNQ
BDI
UGA
COM
AGO
COD
ZMB
TZA
ZWE
MWI
MOZ
MDG
100
80
60
40
'20 '25 '30 '35

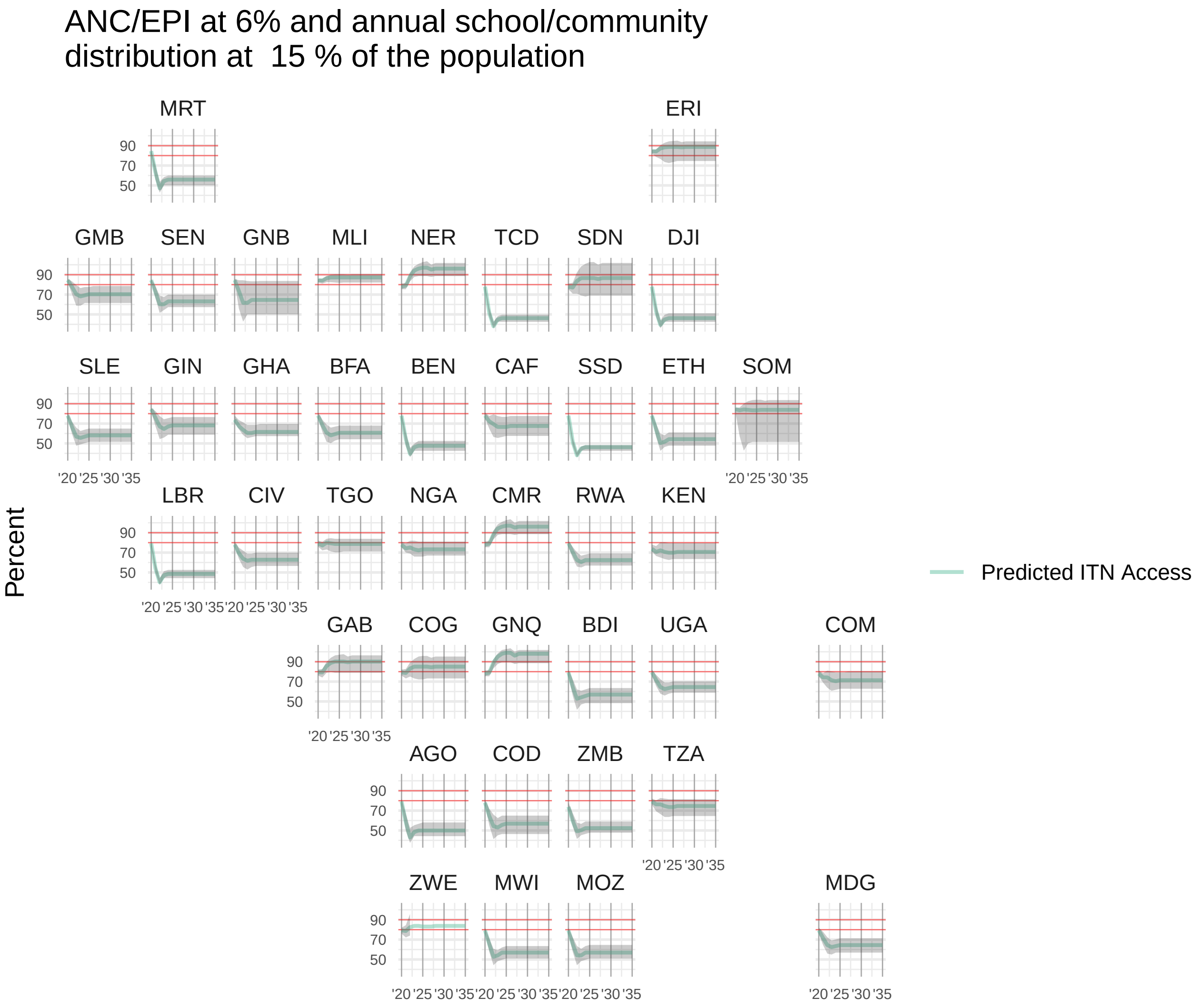
ANC/EPI at 6% and annual school/community distribution at 15 % of the population
MRT
ERI
GMB
SEN
GNB
MLI
NER
TCD
SDN
DJI
SLE
GIN
GHA
BFA
BEN
CAF
SSD
ETH
SOM
LBR
CIV
TGO
NGA
CMR
RWA
KEN
GAB
COG
GNQ
BDI
UGA
COM
AGO
COD
ZMB
TZA
ZWE
MWI
MOZ
MDG
90
70
50
'20 '25 '30 '35
Percent
Predicted ITN Access

ANC/EPI at 6% and annual school/community distribution at 16 % of the population

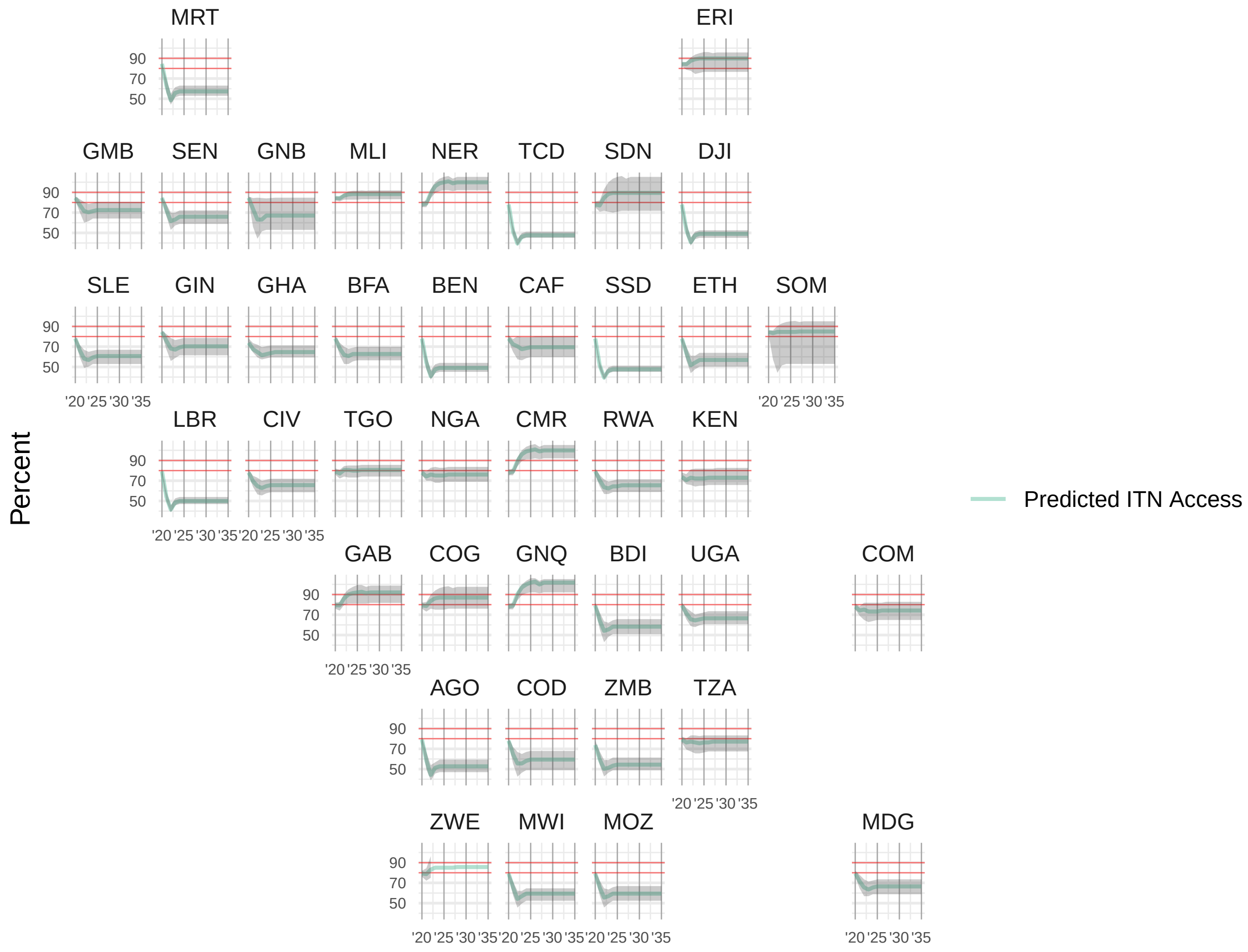

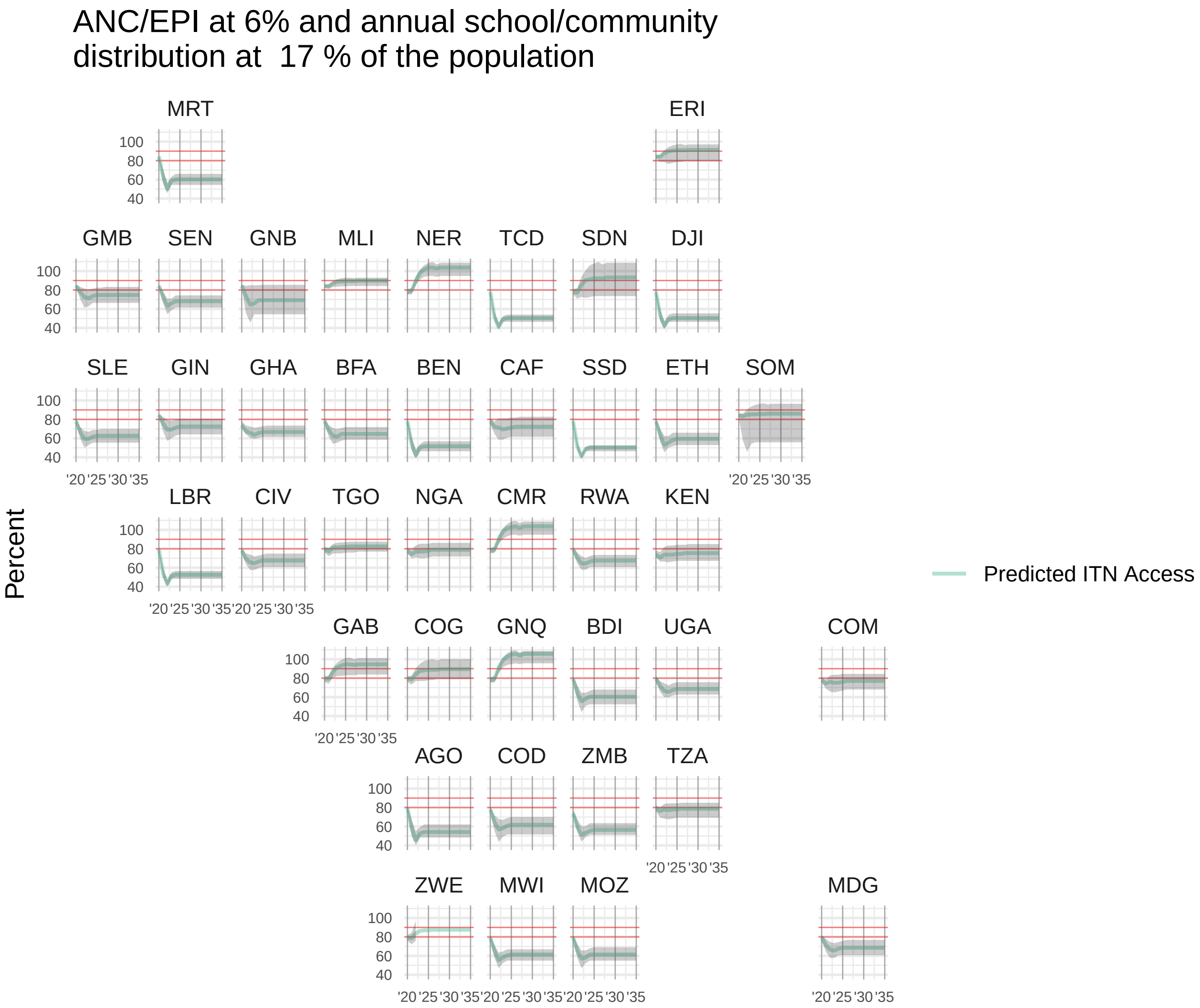

ANC/EPI at 6% and annual school/community distribution at 17 % of the population
MRT
ERI
GMB
SEN
GNB
MLI
NER
TCD
SDN
DJI
SLE
GIN
GHA
BFA
BEN
CAF
SSD
ETH
SOM
LBR
CIV
TGO
NGA
CMR
RWA
KEN
GAB
COG
GNQ
BDI
UGA
COM
AGO
COD
ZMB
TZA
ZWE
MWI
MOZ
MDG
Percent
100
80
60
40
'20 '25 '30 '35
Predicted ITN Access

ANC/EPI at 6% and annual school/community distribution at 18 % of the population

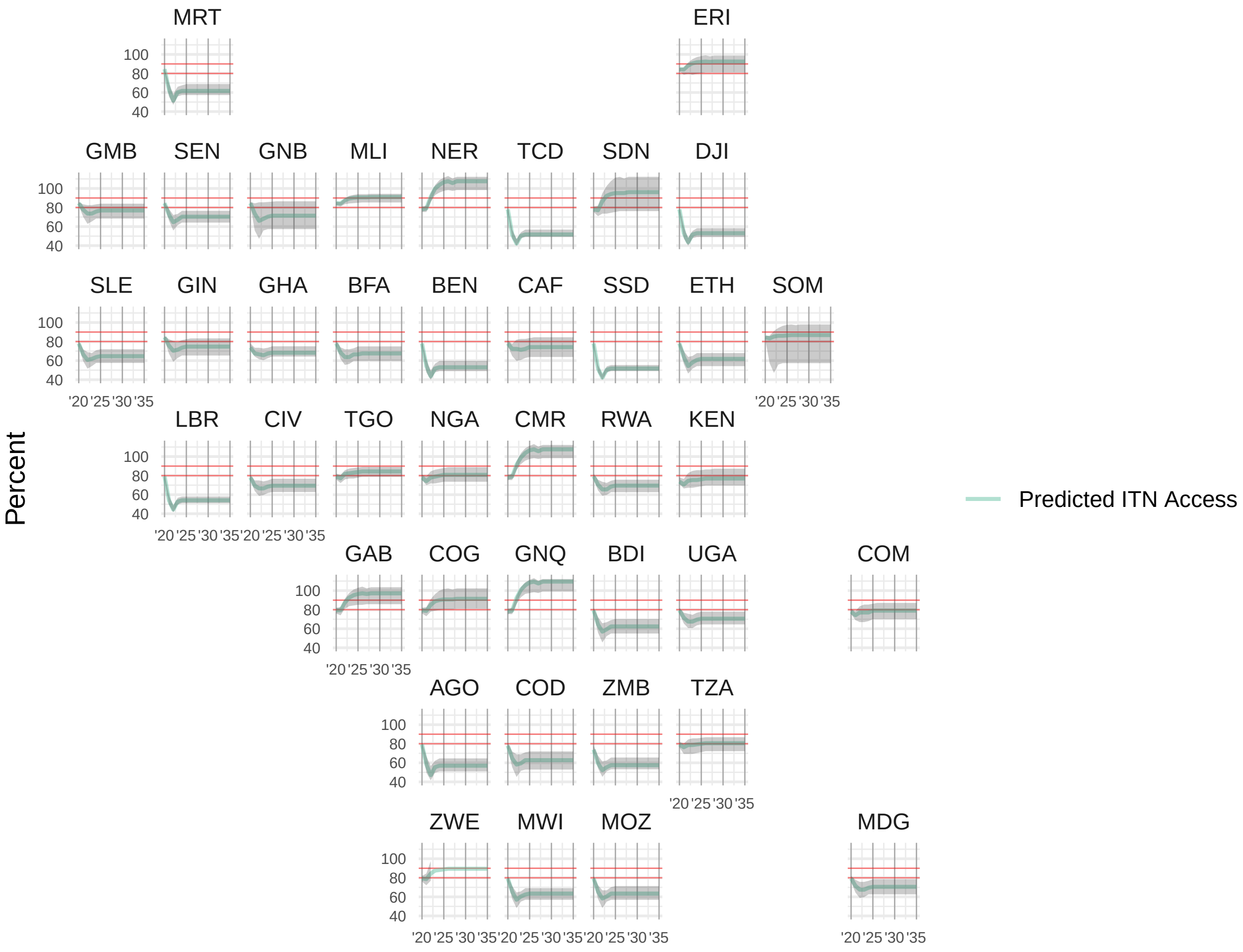

ANC/EPI at 6% and annual school/community distribution at 19 % of the population

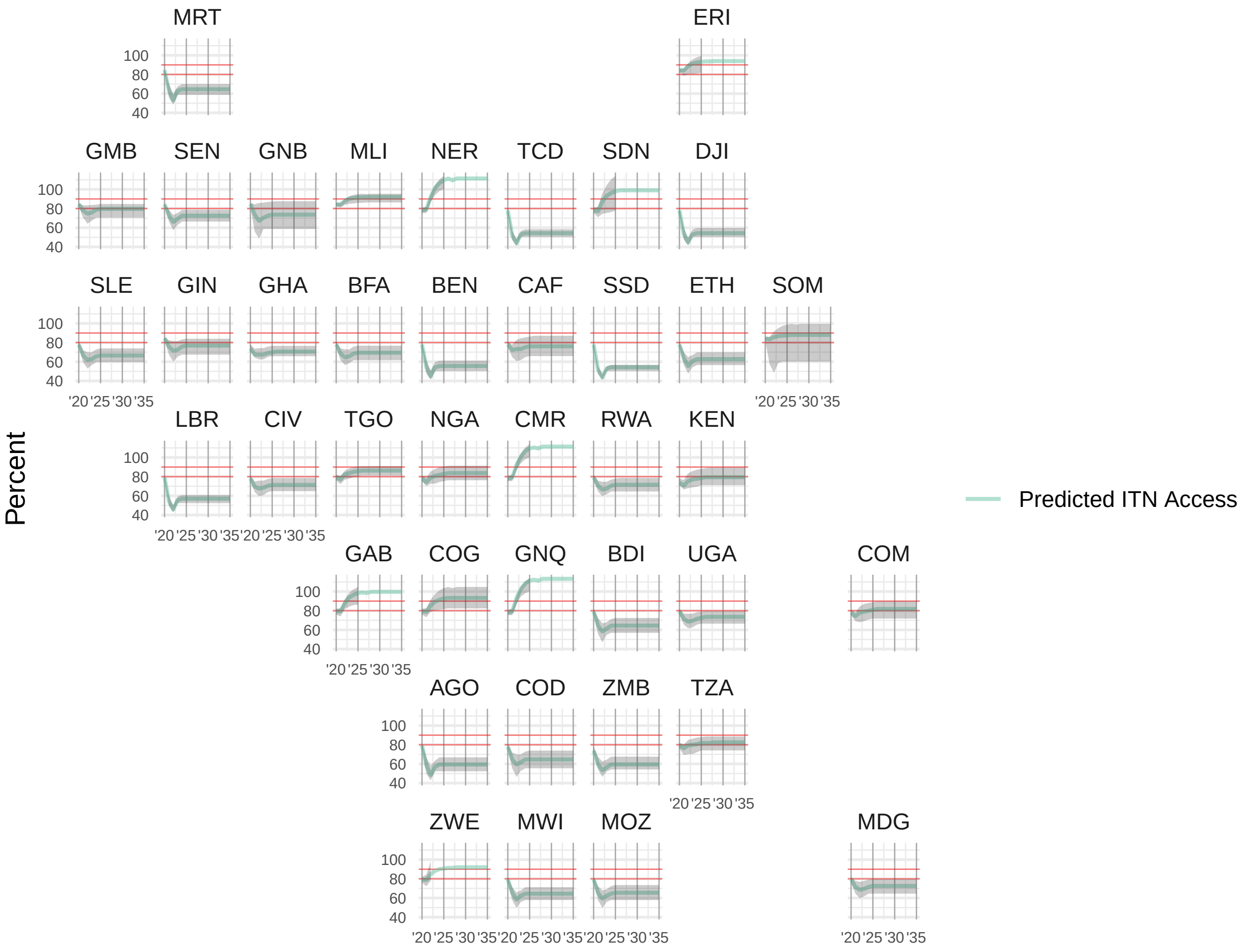

ANC/EPI at 6% and annual school/community distribution at 20 % of the population

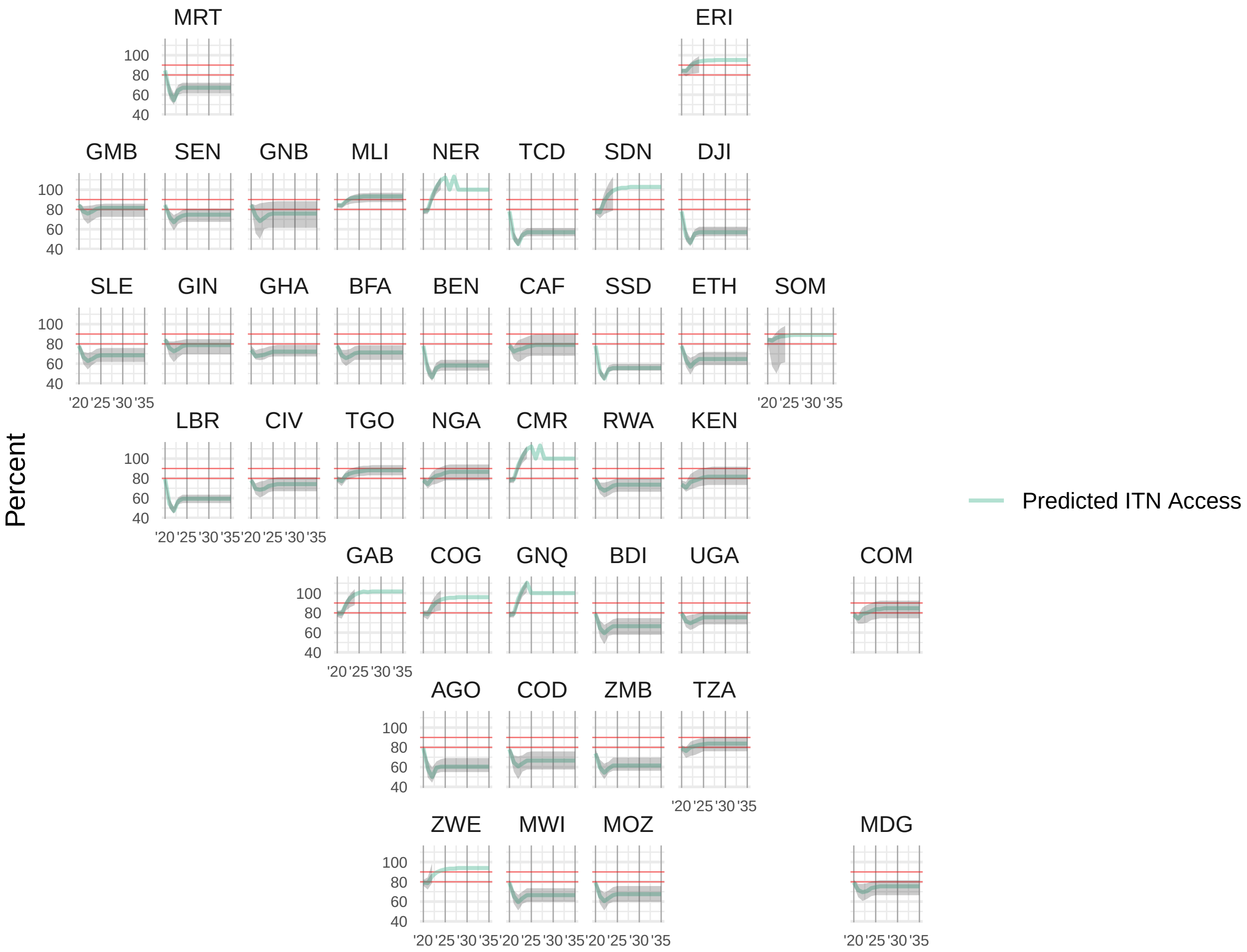

ANC/EPI at 6% and annual school/community distribution at 21 % of the population

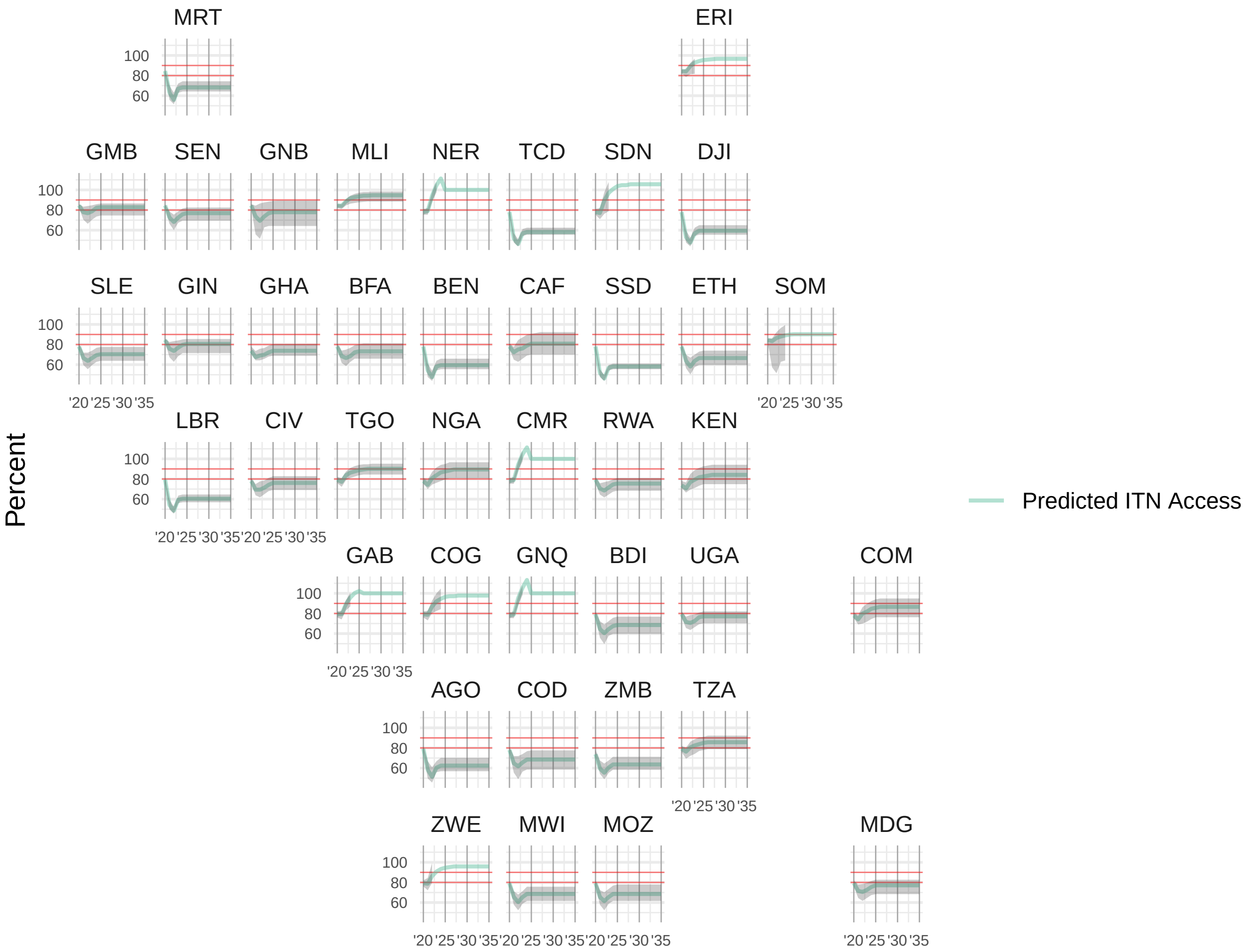

ANC/EPI at 6% and annual school/community distribution at 22 % of the population

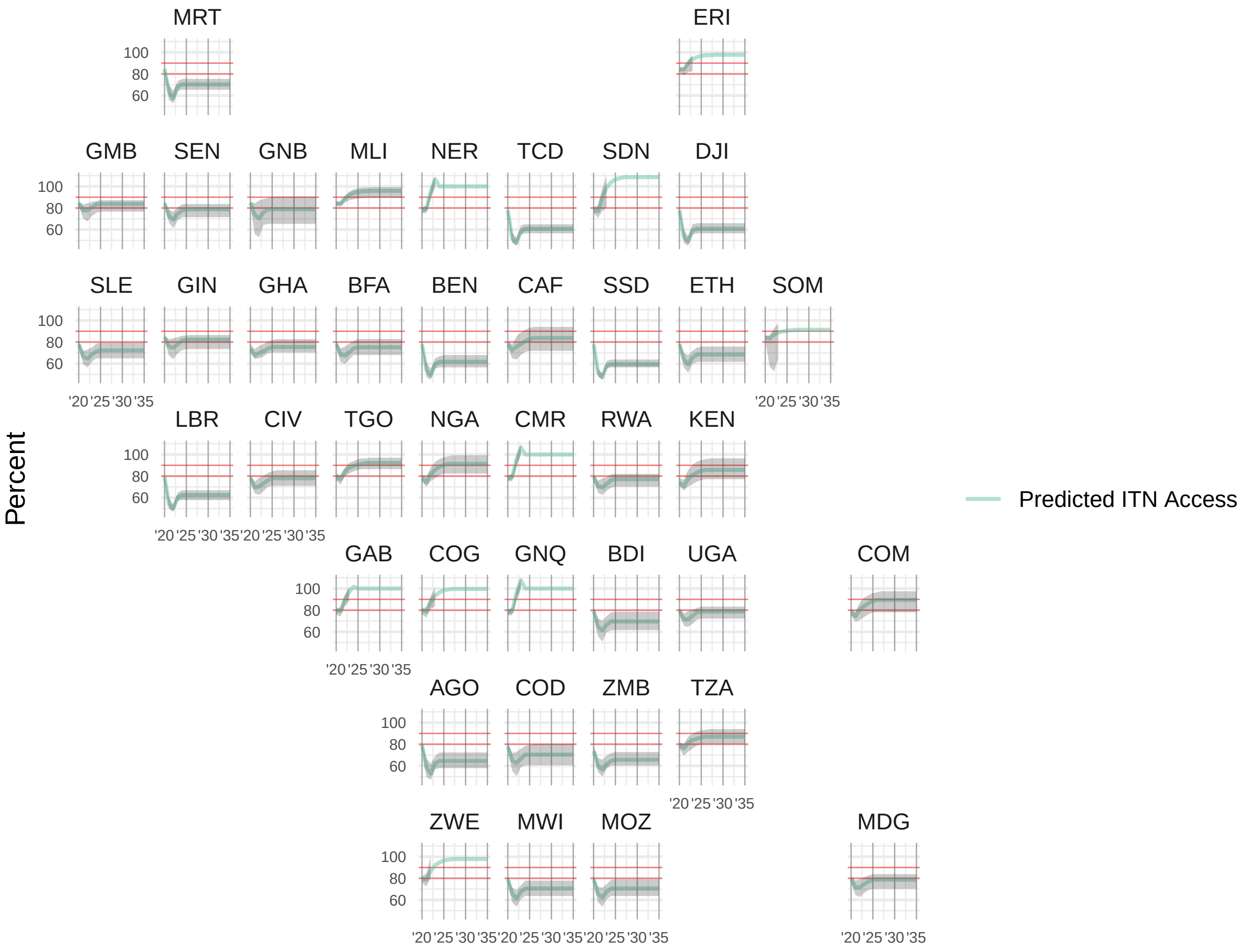

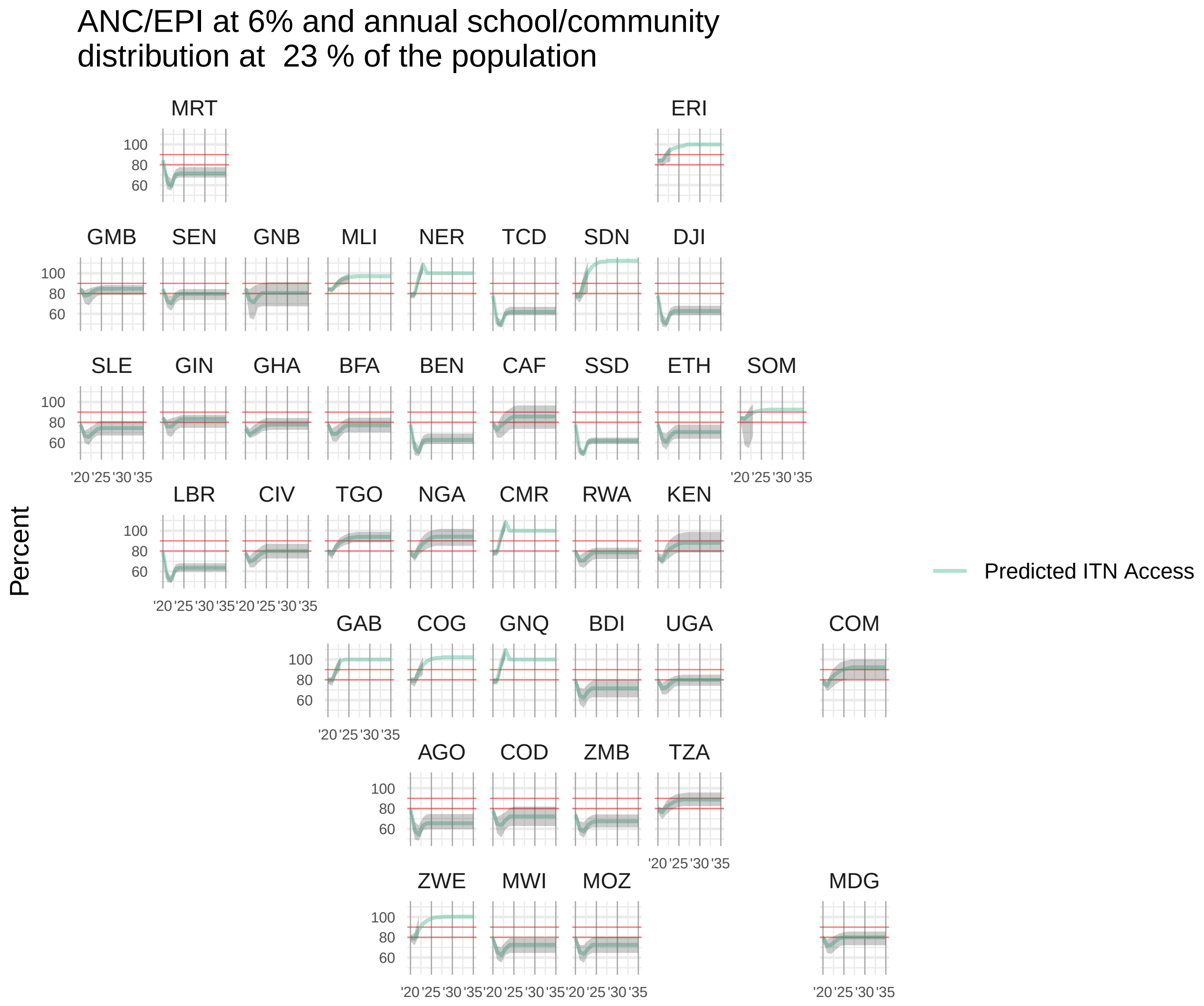
ANC/EPI at 6% and annual school/community distribution at 23 % of the population
MRT
ERI
GMB
SEN
GNB
MLI
NER
TCD
SDN
DJI
SLE
GIN
GHA
BFA
BEN
CAF
SSD
ETH
SOM
LBR
CIV
TGO
NGA
CMR
RWA
KEN
GAB
COG
GNQ
BDI
UGA
COM
AGO
COD
ZMB
TZA
ZWE
MWI
MOZ
MDG
Percent
100
80
60
'20 '25 '30 '35
Predicted ITN Access

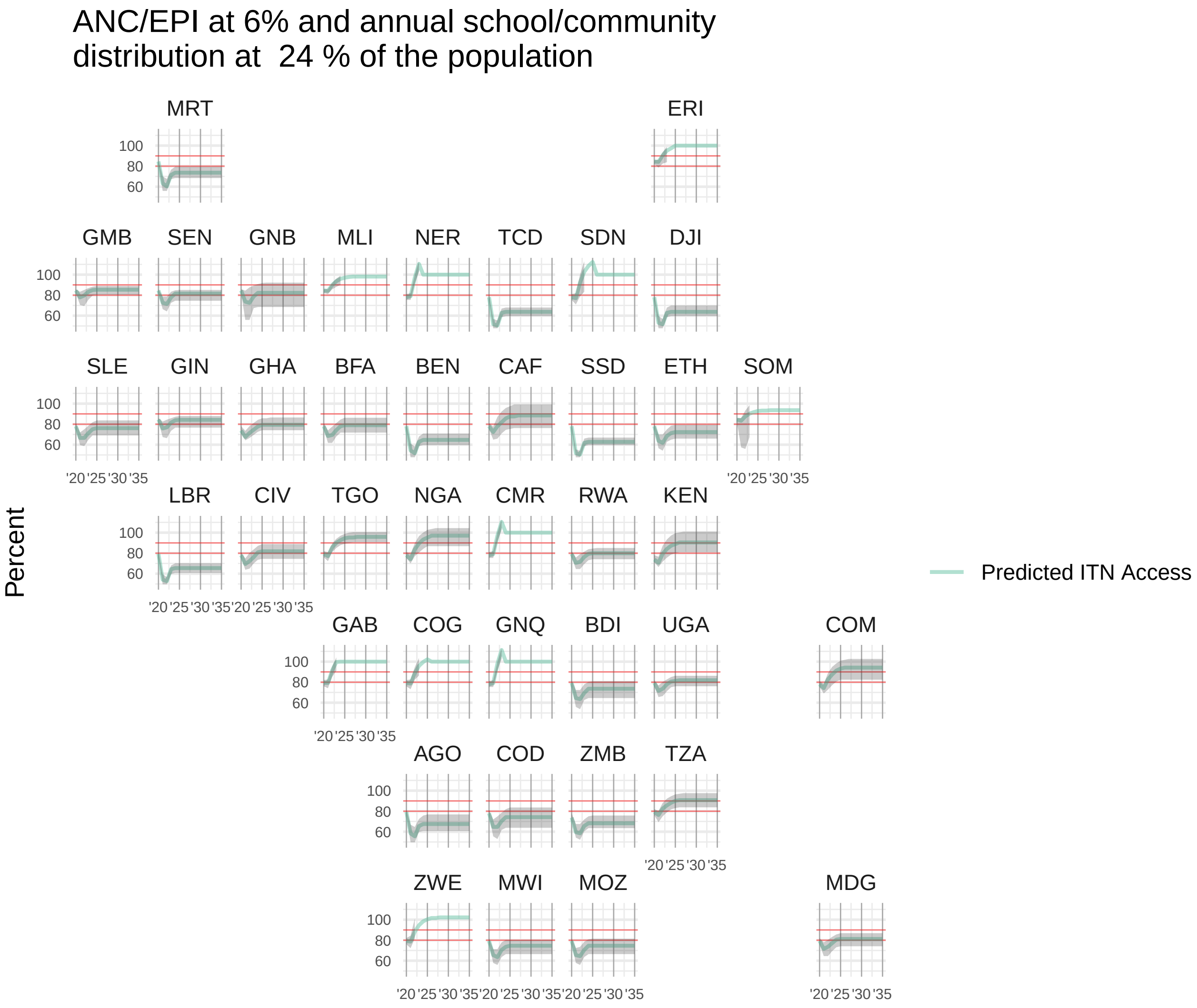
ANC/EPI at 6% and annual school/community distribution at 24 % of the population
Percent
Predicted ITN Access
MRT
ERI
GMB
SEN
GNB
MLI
NER
TCD
SDN
DJI
SLE
GIN
GHA
BFA
BEN
CAF
SSD
ETH
SOM
LBR
CIV
TGO
NGA
CMR
RWA
KEN
GAB
COG
GNQ
BDI
UGA
COM
AGO
COD
ZMB
TZA
ZWE
MWI
MOZ
MDG
100
80
60
'20 '25 '30 '35

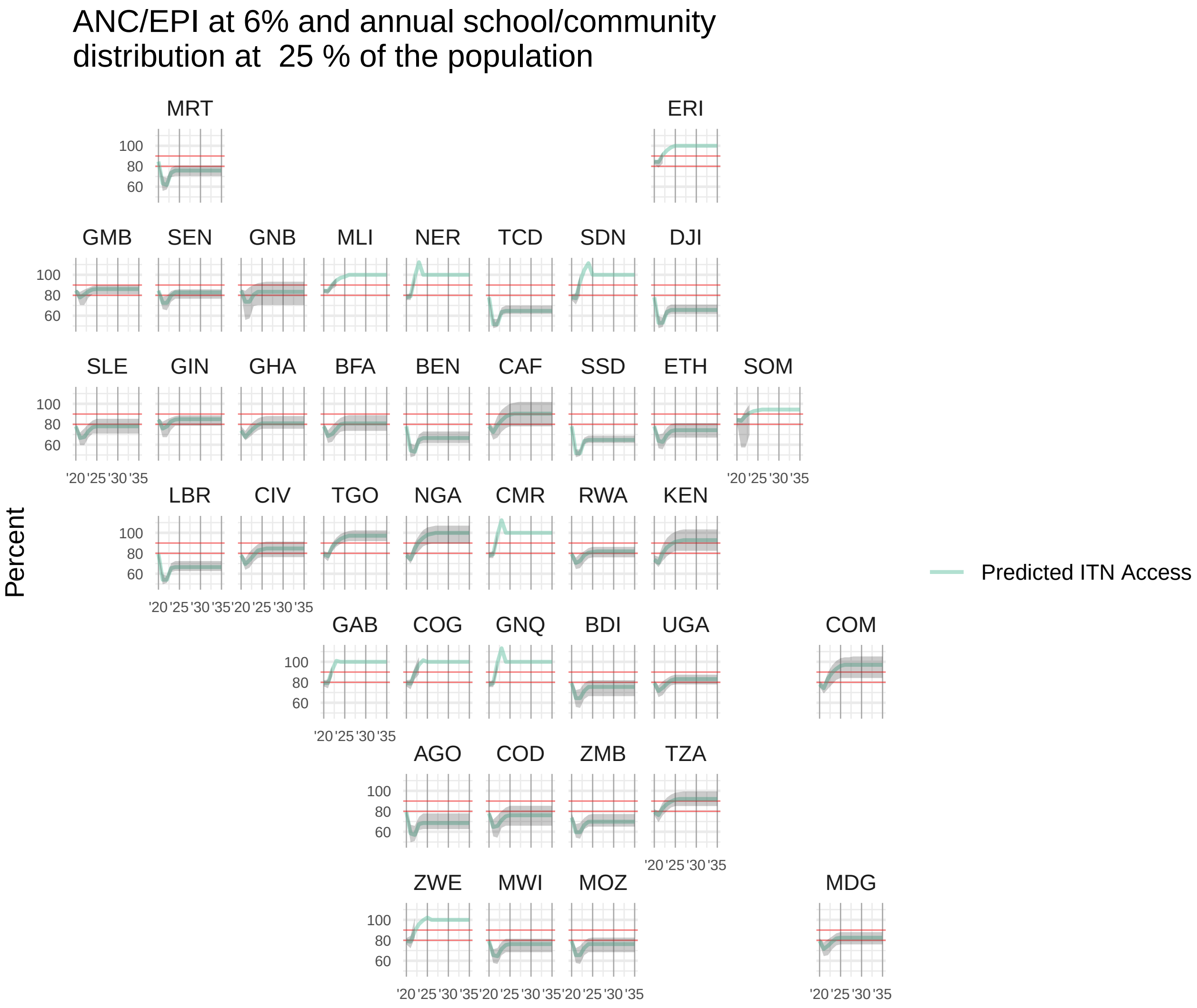
ANC/EPI at 6% and annual school/community distribution at 25 % of the population
MRT
ERI
GMB
SEN
GNB
MLI
NER
TCD
SDN
DJI
SLE
GIN
GHA
BFA
BEN
CAF
SSD
ETH
SOM
LBR
CIV
TGO
NGA
CMR
RWA
KEN
GAB
COG
GNQ
BDI
UGA
COM
AGO
COD
ZMB
TZA
ZWE
MWI
MOZ
MDG
100
80
60
'20 '25 '30 '35
Percent
Predicted ITN Access

ANC/EPI at 6% and annual school/community distribution at 26 % of the population

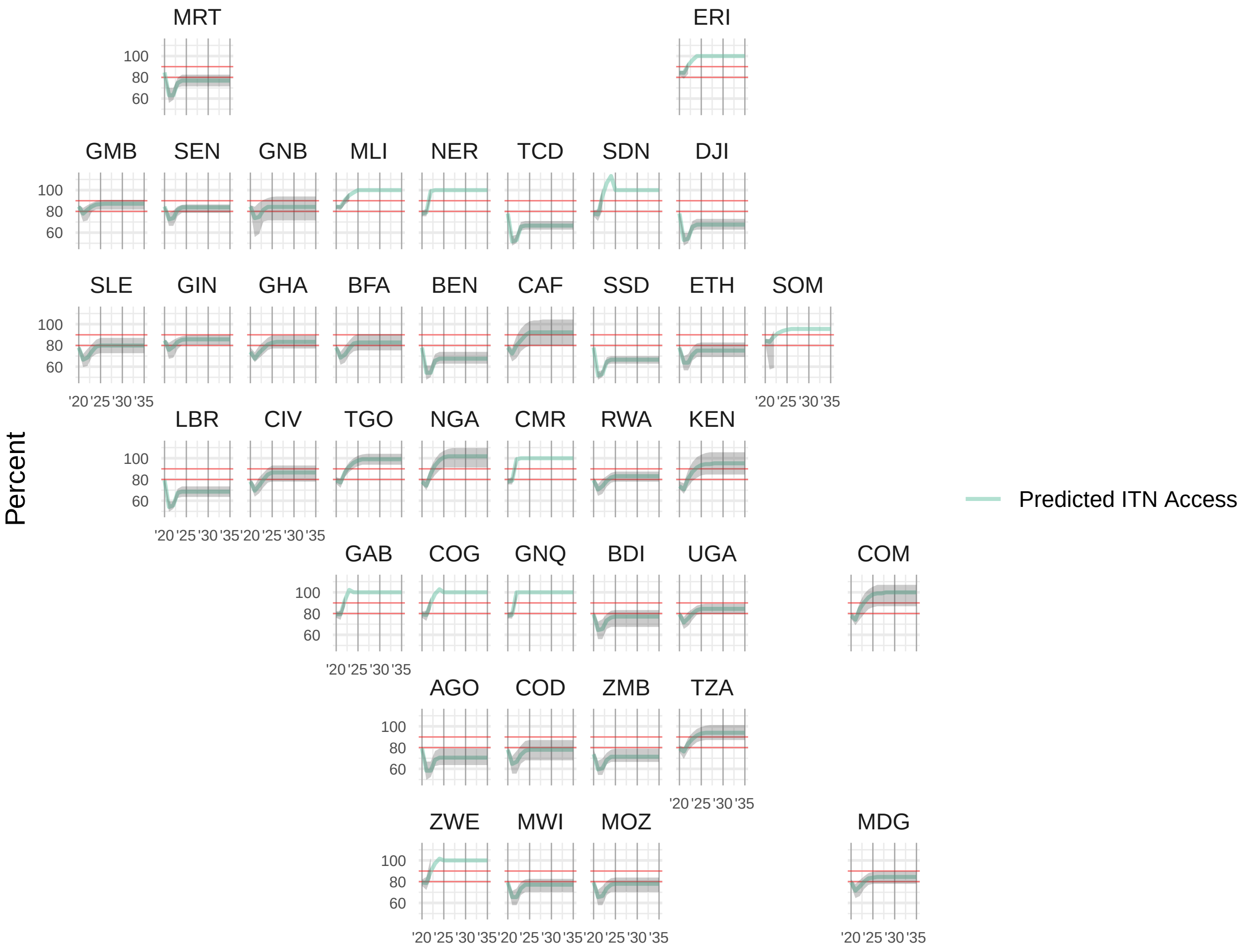

ANC/EPI at 6% and annual school/community distribution at 27 % of the population

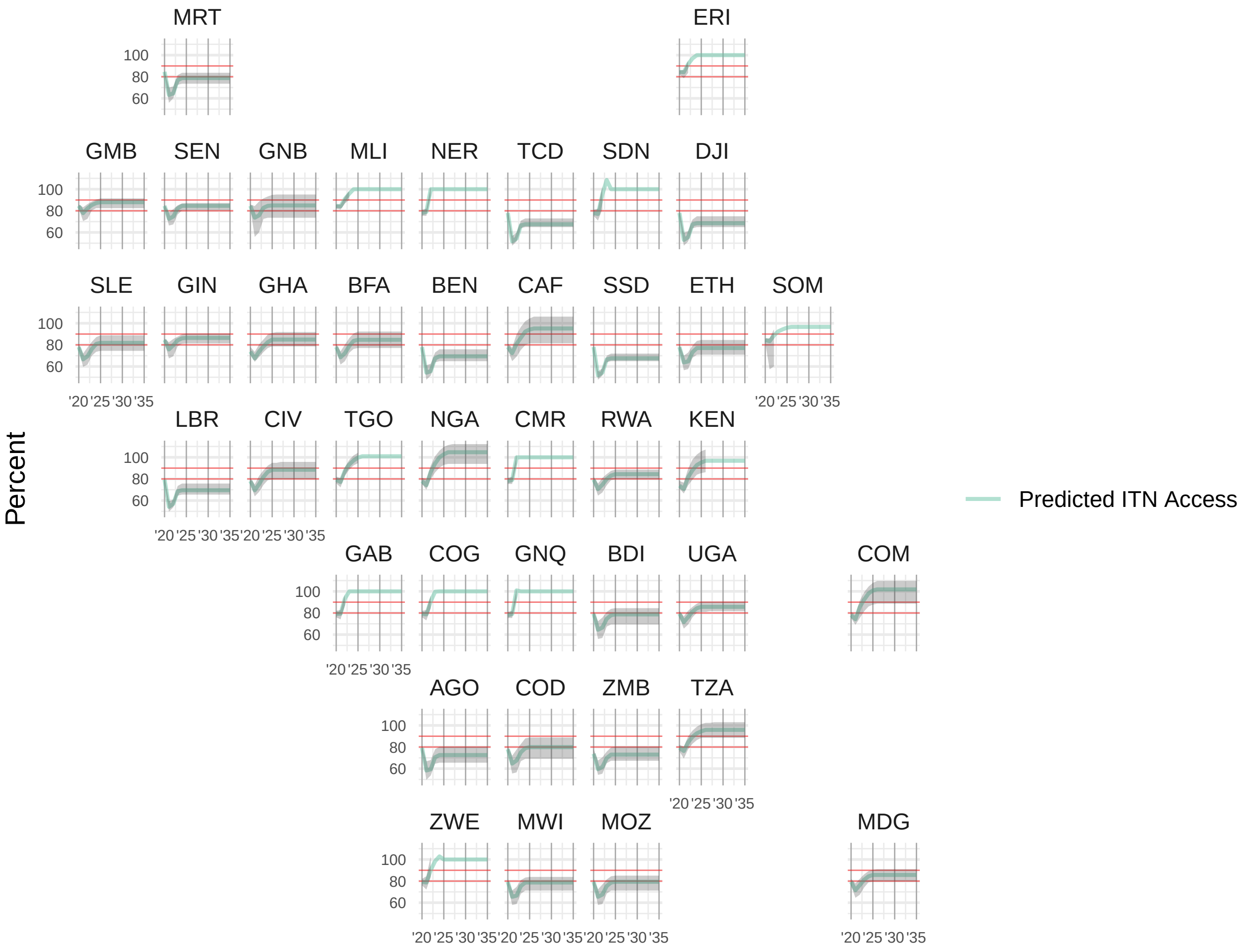

ANC/EPI at 6% and annual school/community distribution at 28 % of the population

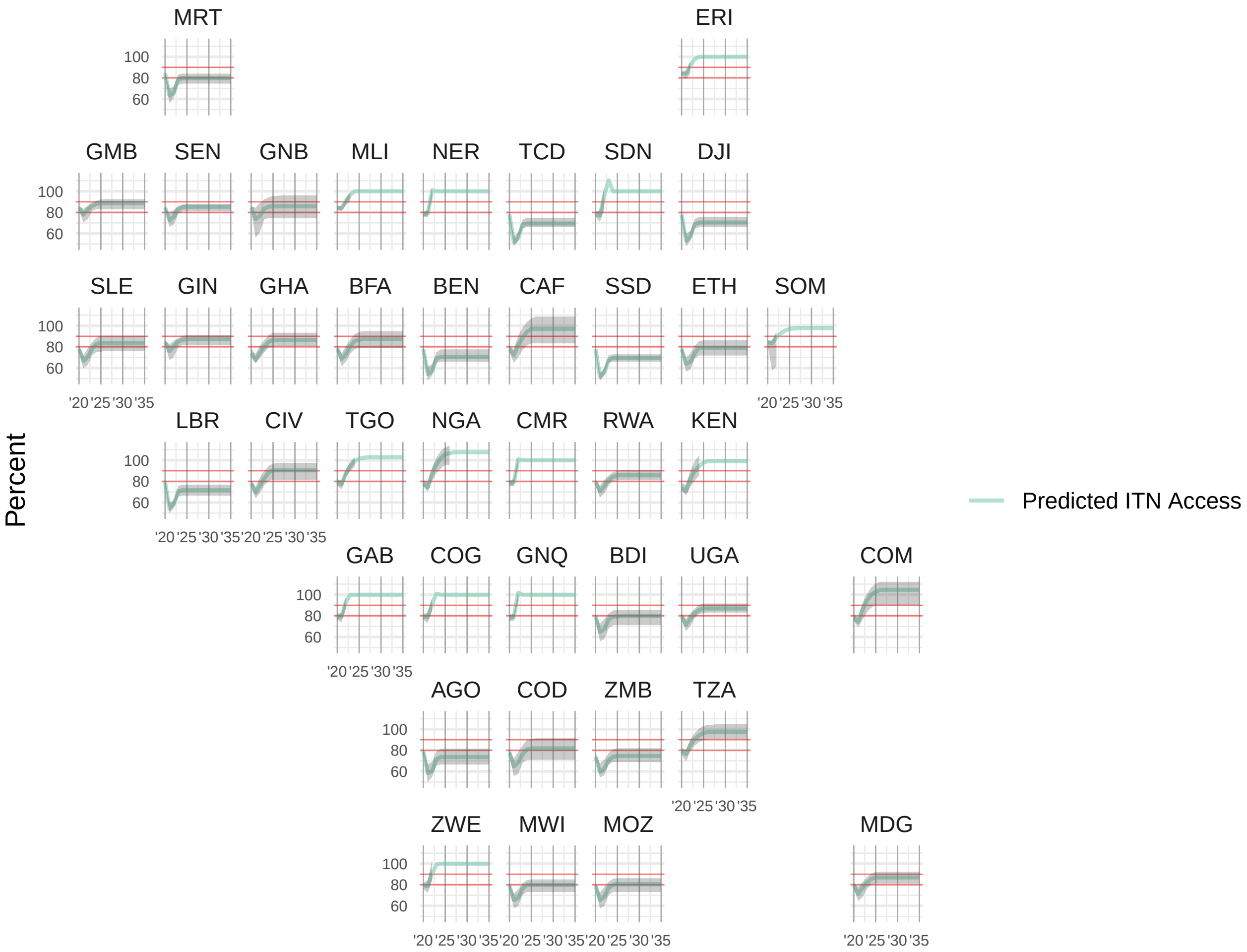

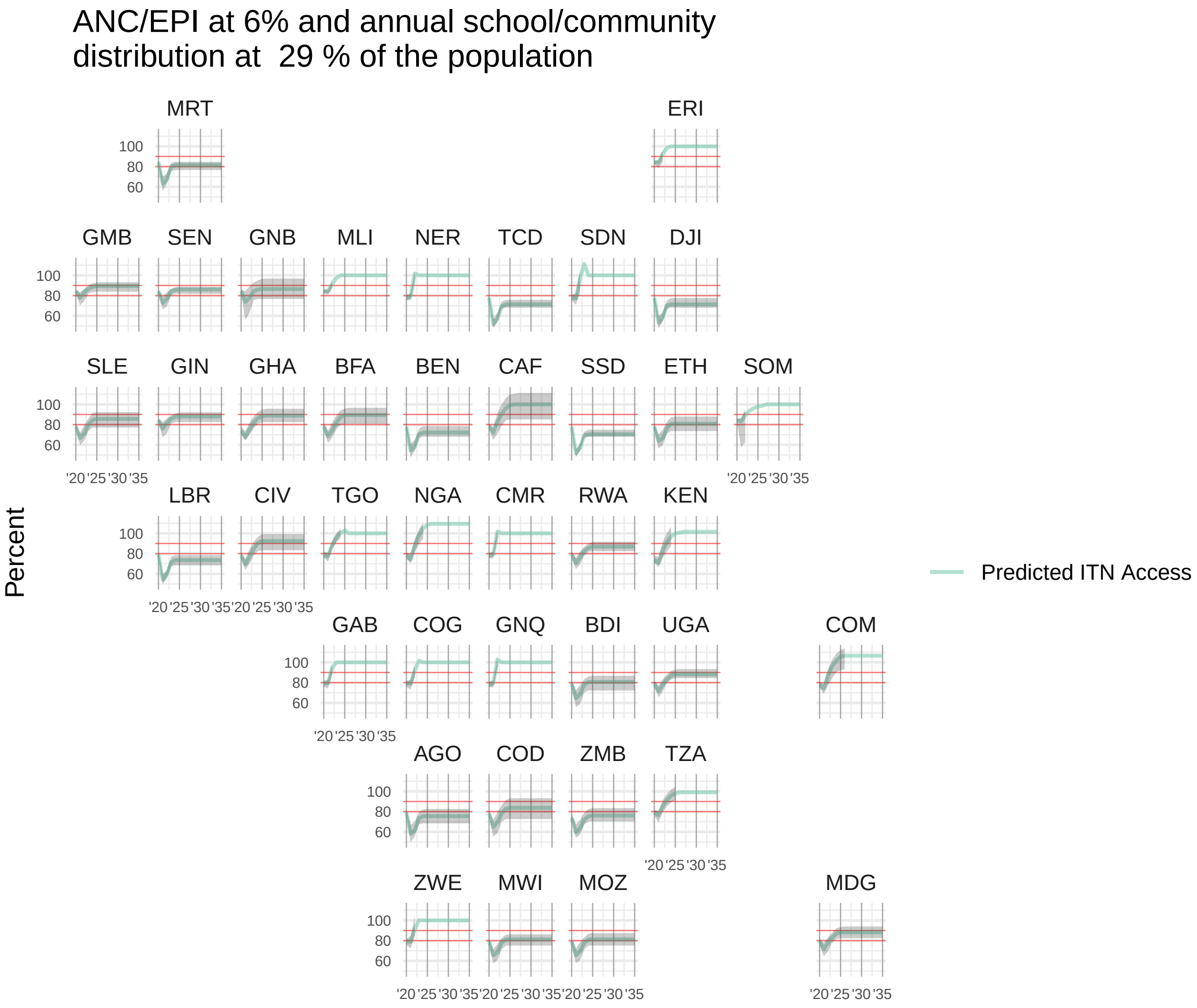
ANC/EPI at 6% and annual school/community distribution at 29 % of the population
MRT
ERI
GMB
SEN
GNB
MLI
NER
TCD
SDN
DJI
SLE
GIN
GHA
BFA
BEN
CAF
SSD
ETH
SOM
LBR
CIV
TGO
NGA
CMR
RWA
KEN
GAB
COG
GNQ
BDI
UGA
COM
AGO
COD
ZMB
TZA
ZWE
MWI
MOZ
MDG
Percent
100
80
60
'20 '25 '30 '35
Predicted ITN Access

ANC/EPI at 6% and annual school/community distribution at 30 % of the population

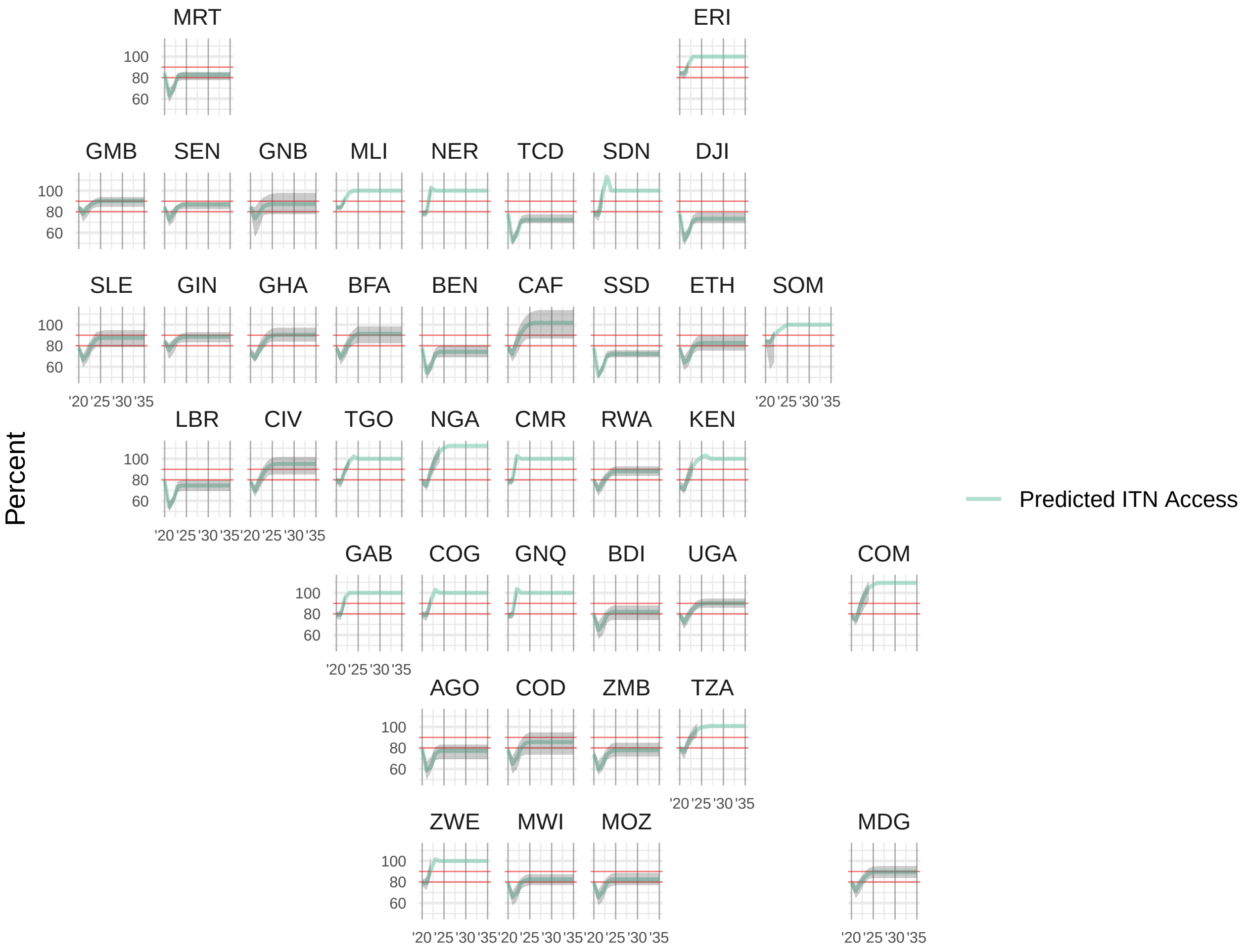

ANC/EPI at 6% and annual school/community distribution at 31 % of the population

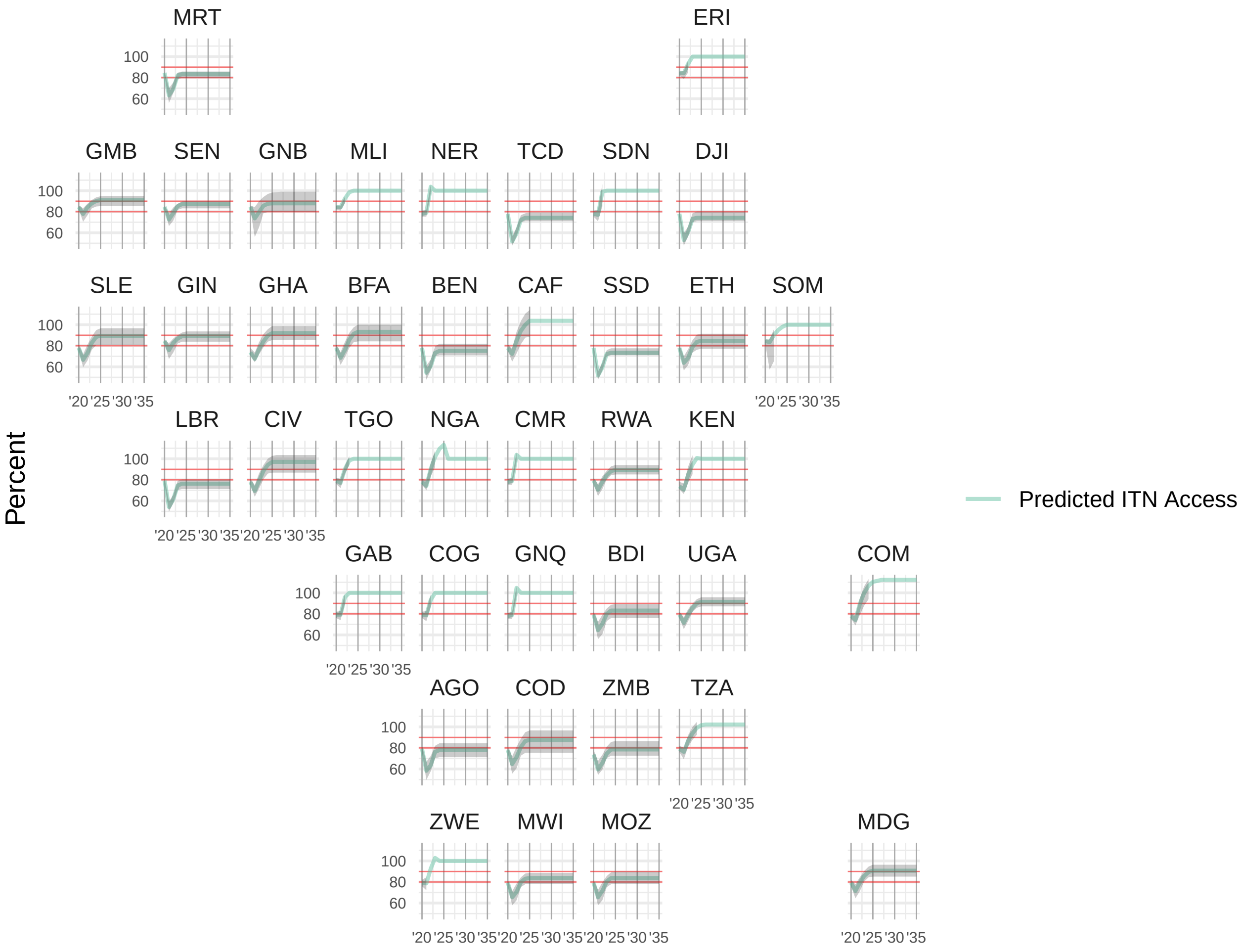

ANC/EPI at 6% and annual school/community distribution at 32 % of the population

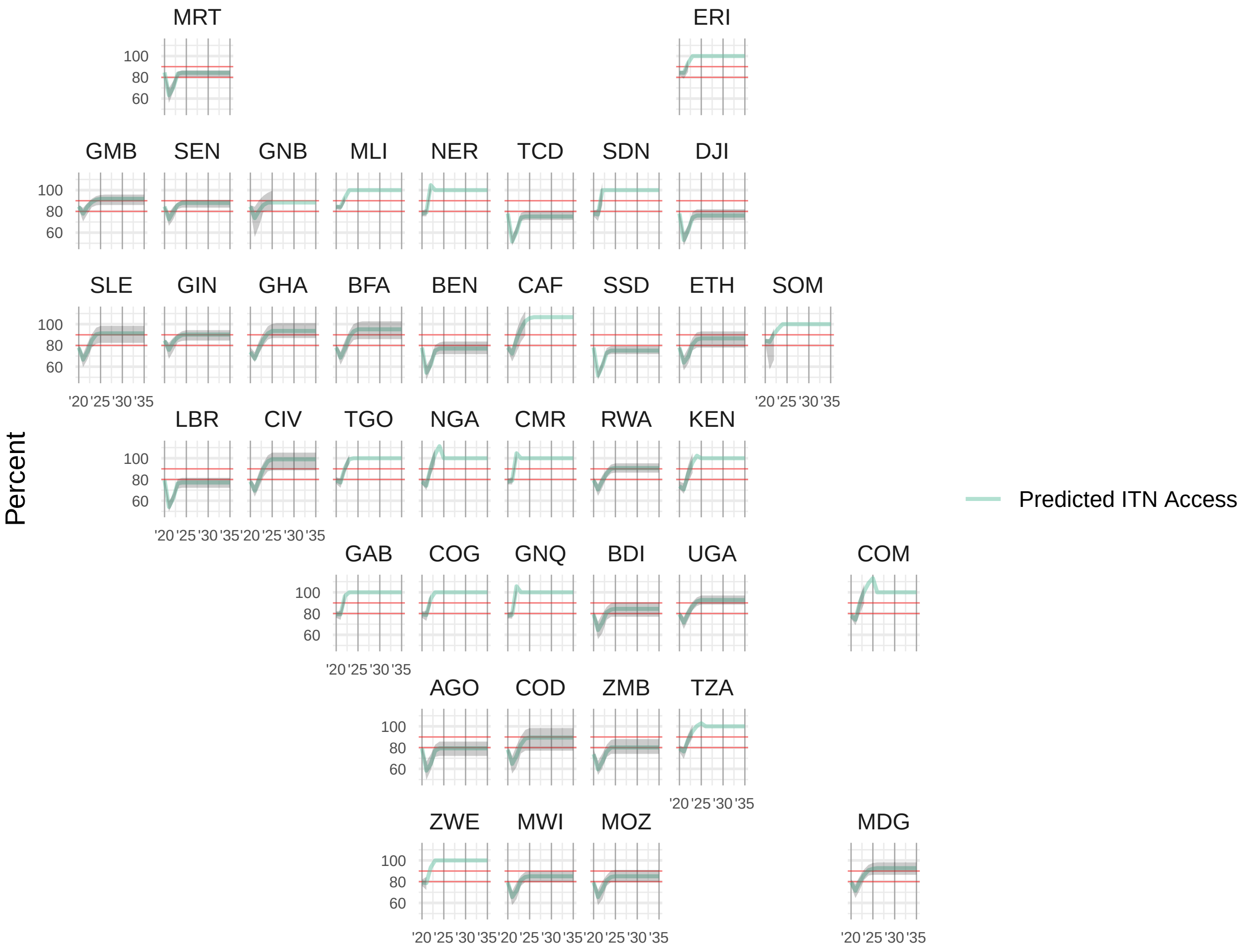

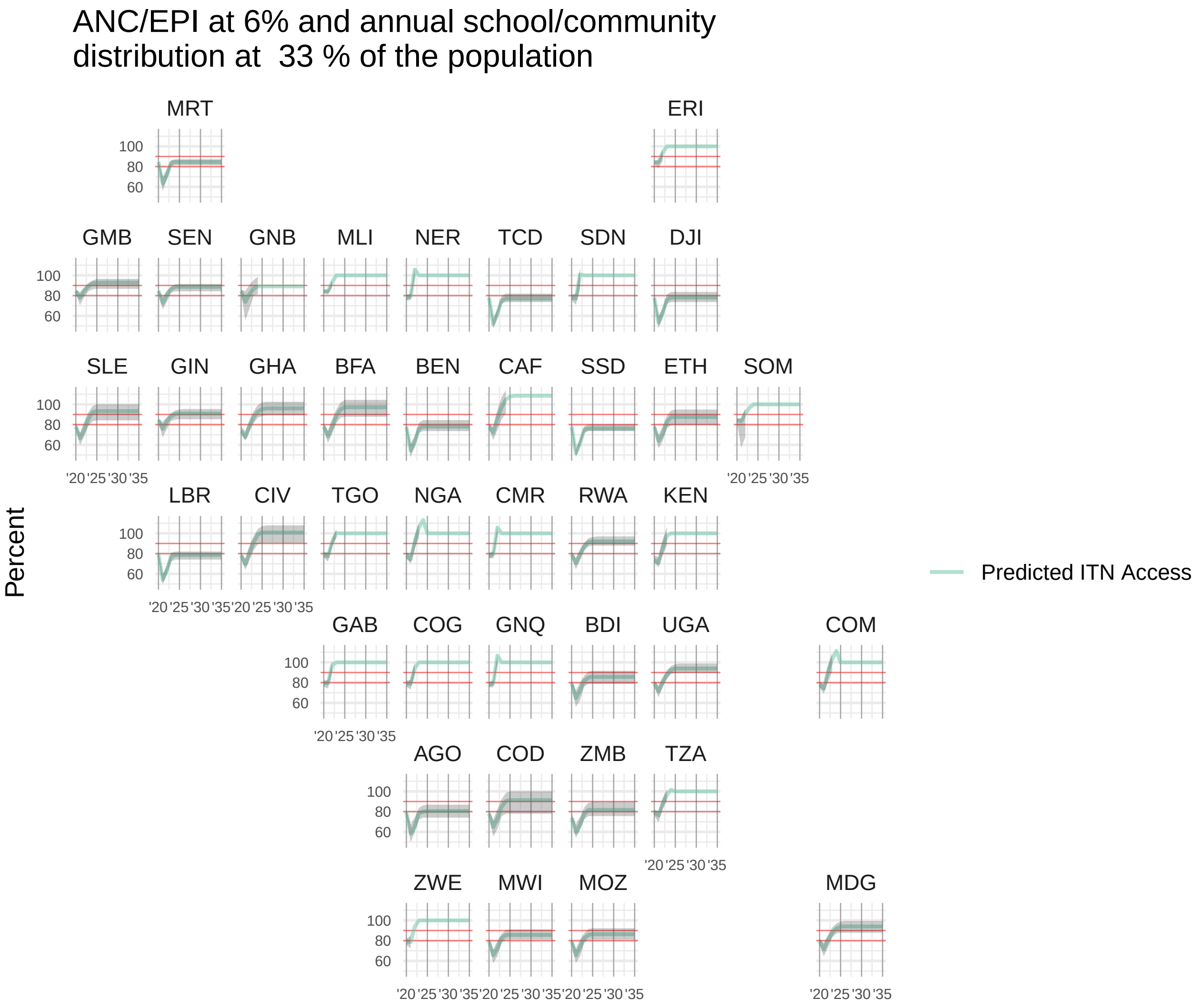

ANC/EPI at 6% and annual school/community distribution at 33 % of the population
MRT
ERI
GMB
SEN
GNB
MLI
NER
TCD
SDN
DJI
SLE
GIN
GHA
BFA
BEN
CAF
SSD
ETH
SOM
LBR
CIV
TGO
NGA
CMR
RWA
KEN
GAB
COG
GNQ
BDI
UGA
COM
AGO
COD
ZMB
TZA
ZWE
MWI
MOZ
MDG
Percent
100
80
60
'20 '25 '30 '35
Predicted ITN Access

ANC/EPI at 6% and annual school/community distribution at 34 % of the population

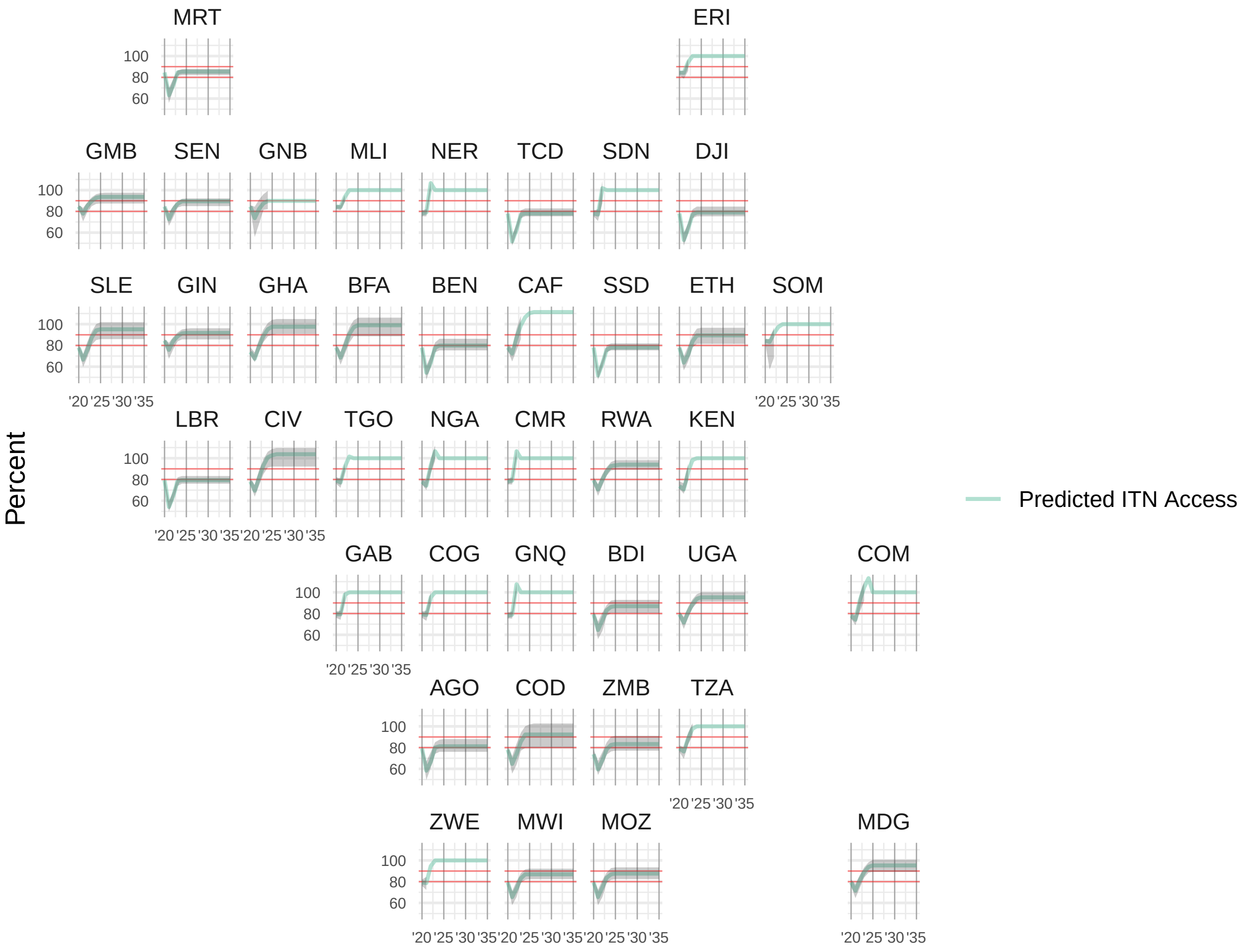

ANC/EPI at 6% and annual school/community distribution at 35 % of the population

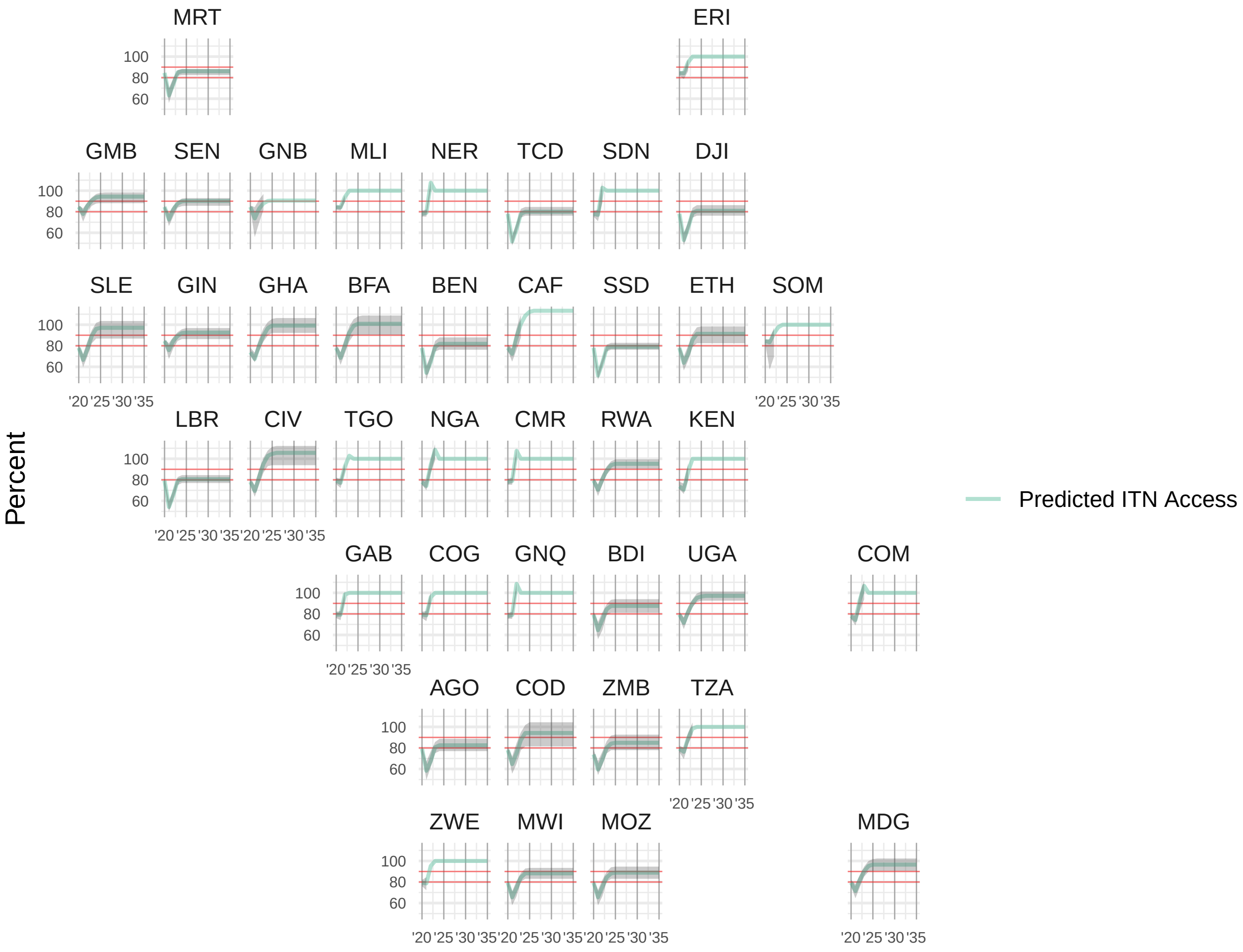

ANC/EPI at 6% and annual school/community distribution at 36 % of the population

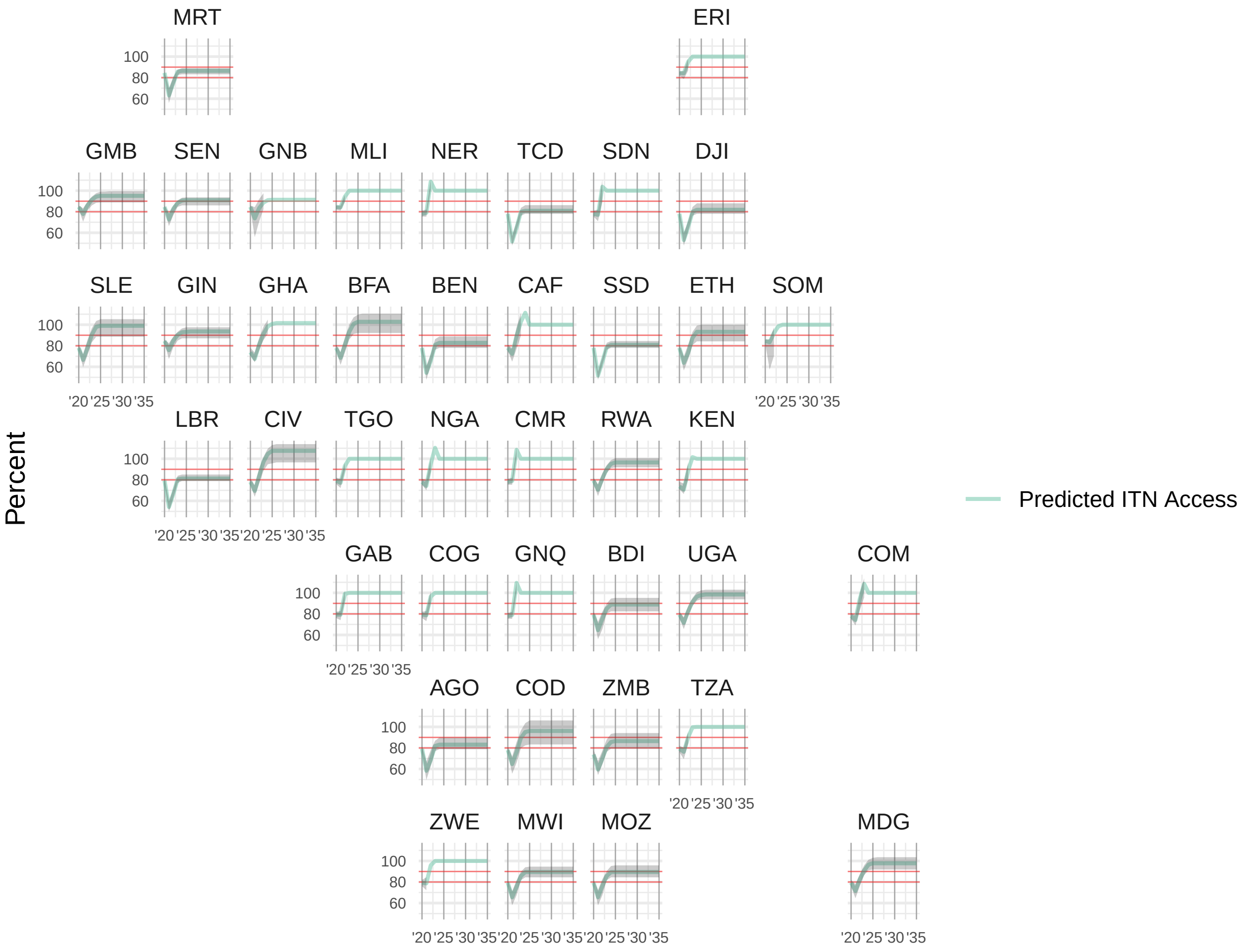

ANC/EPI at 6% and annual school/community distribution at 37 % of the population

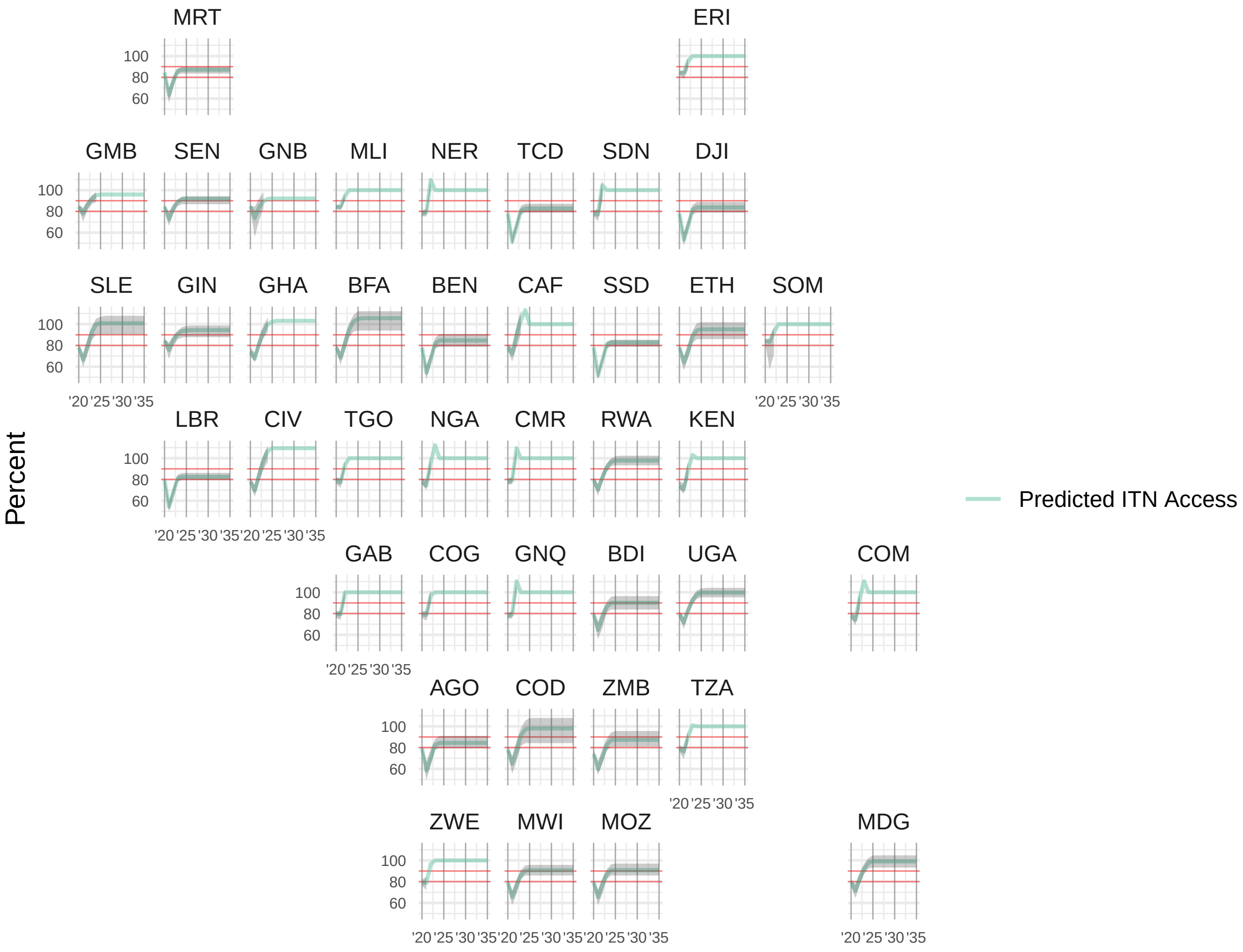

ANC/EPI at 6% and annual school/community distribution at 38 % of the population

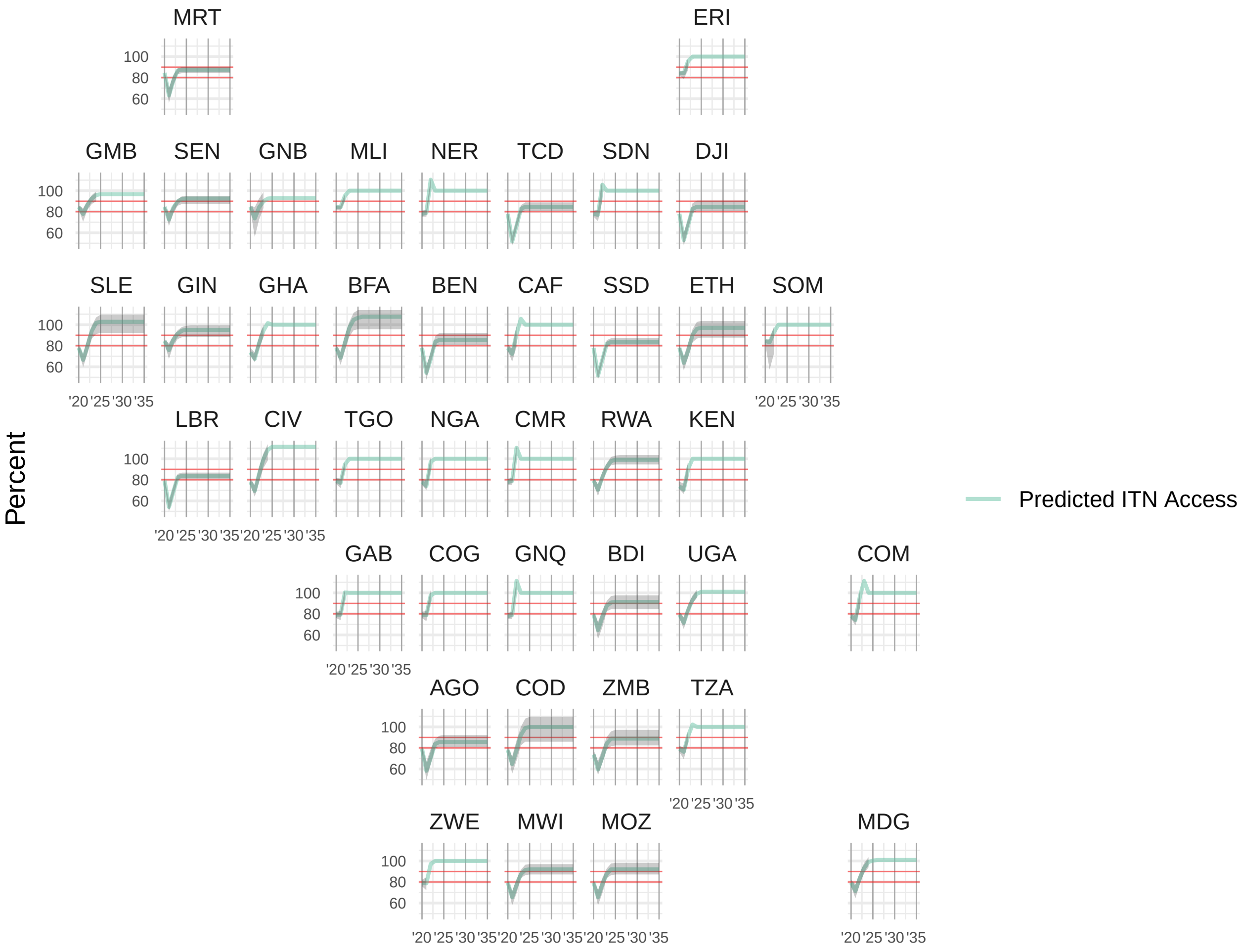

ANC/EPI at 6% and annual school/community distribution at 39 % of the population

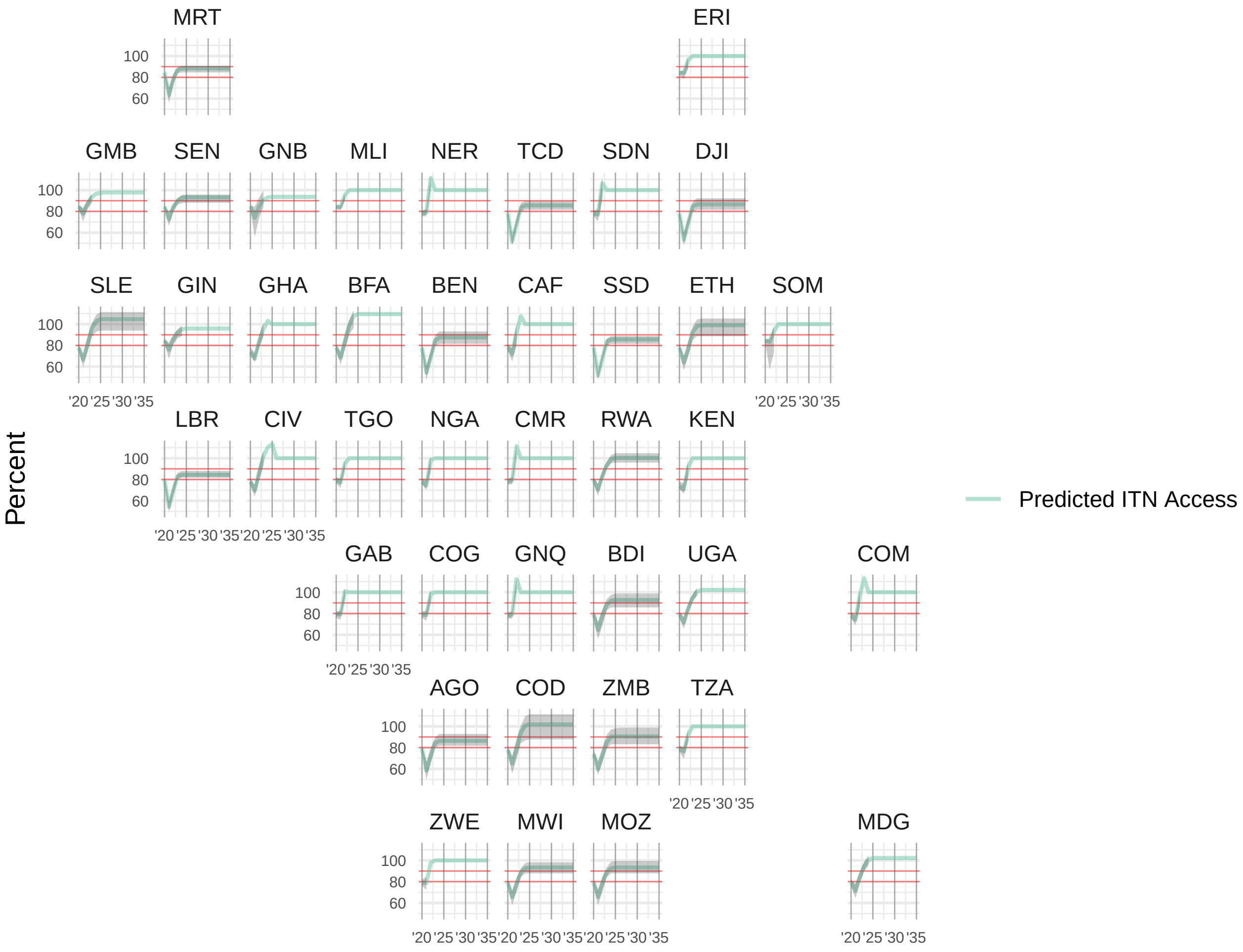

ANC/EPI at 6% and annual school/community distribution at 40 % of the population

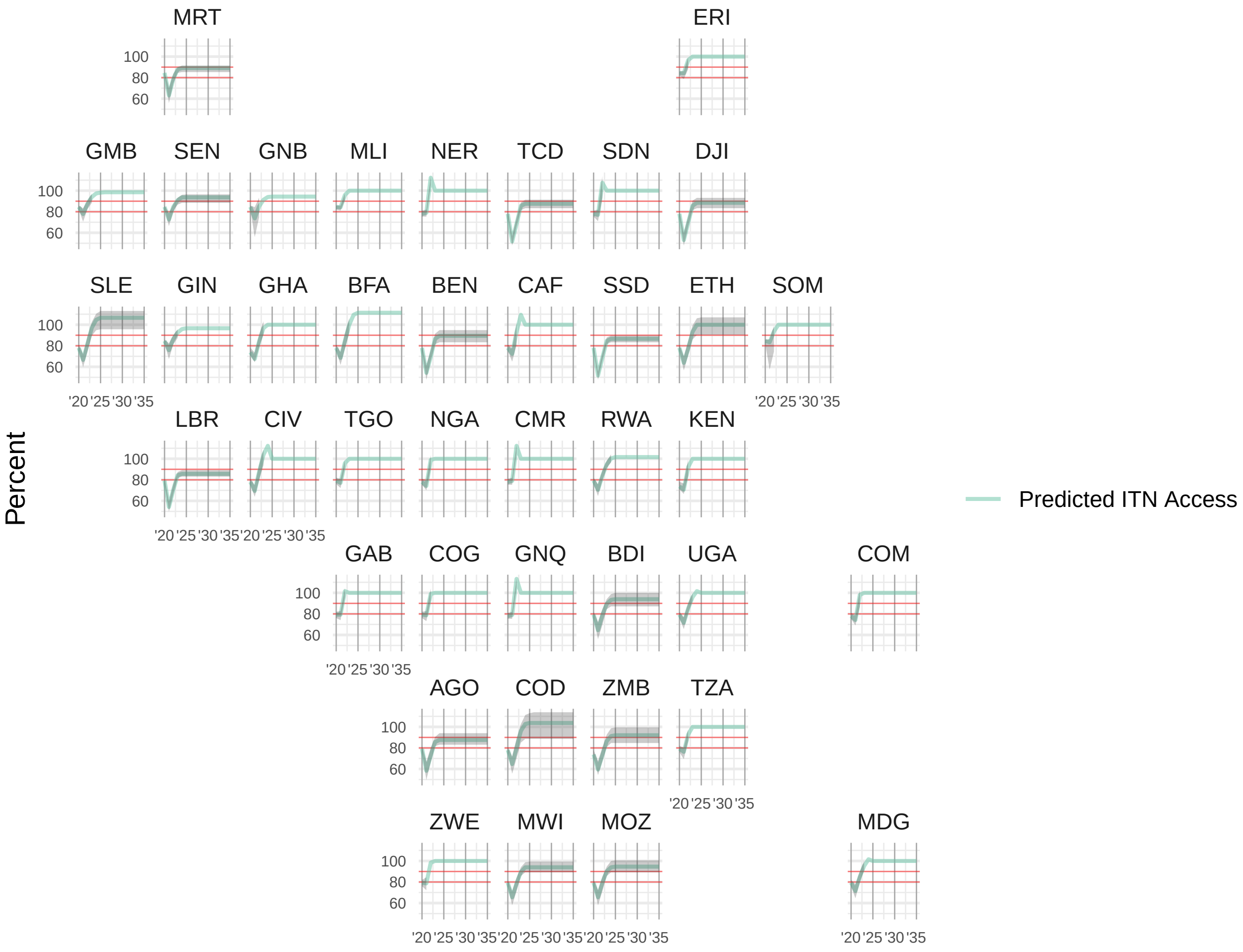

ANC/EPI at 6% and annual school/community distribution at 41 % of the population

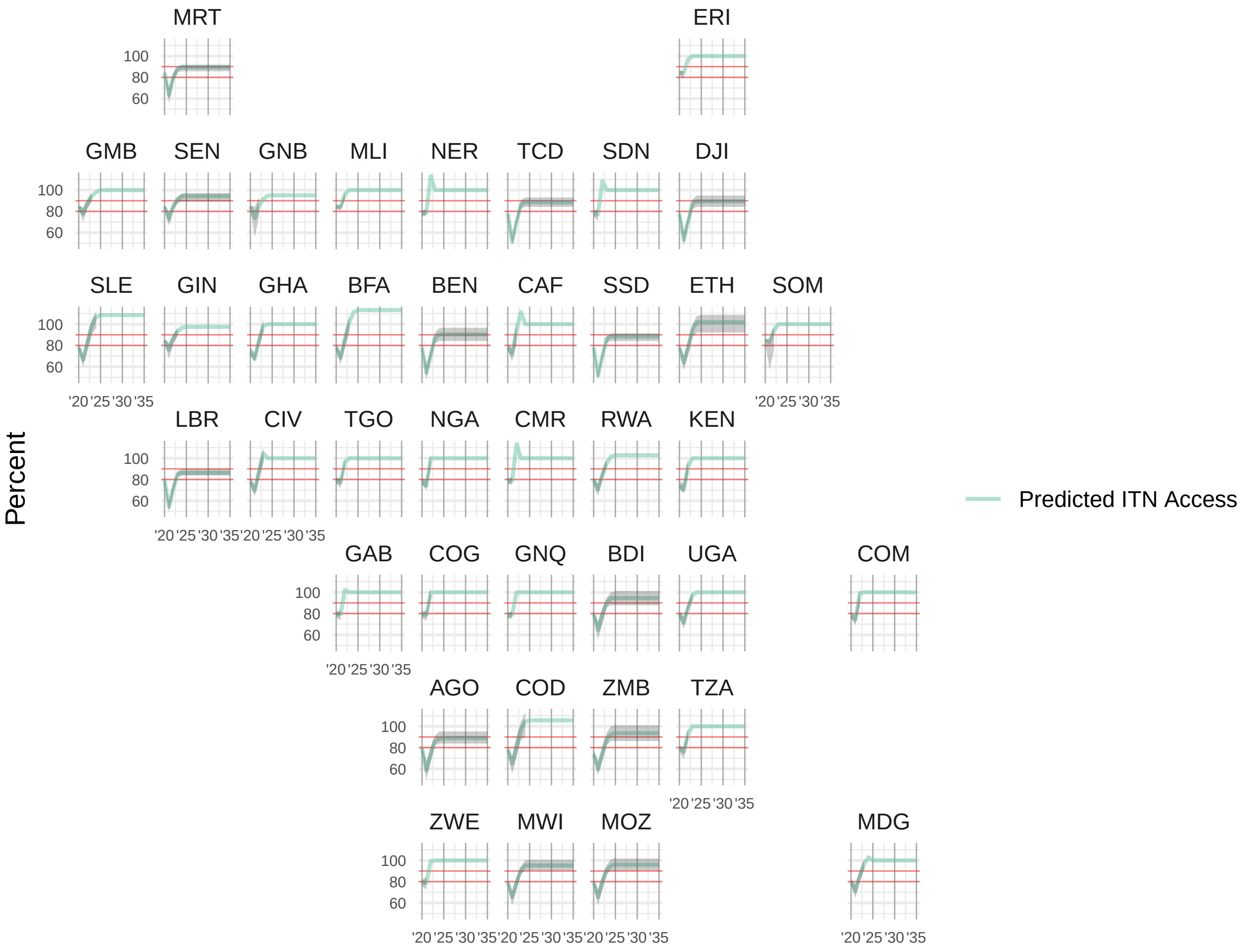

ANC/EPI at 6% and annual school/community distribution at 42 % of the population

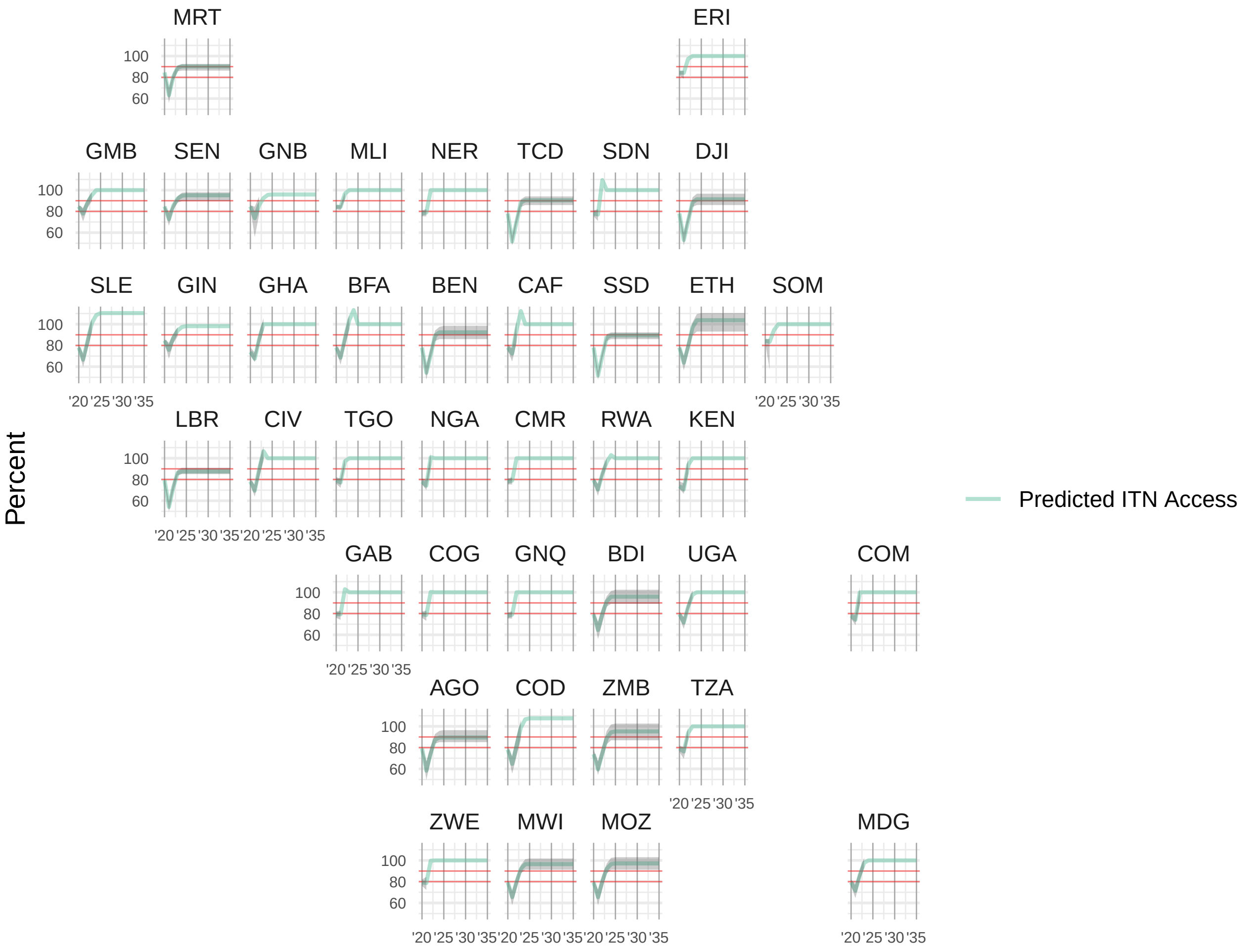

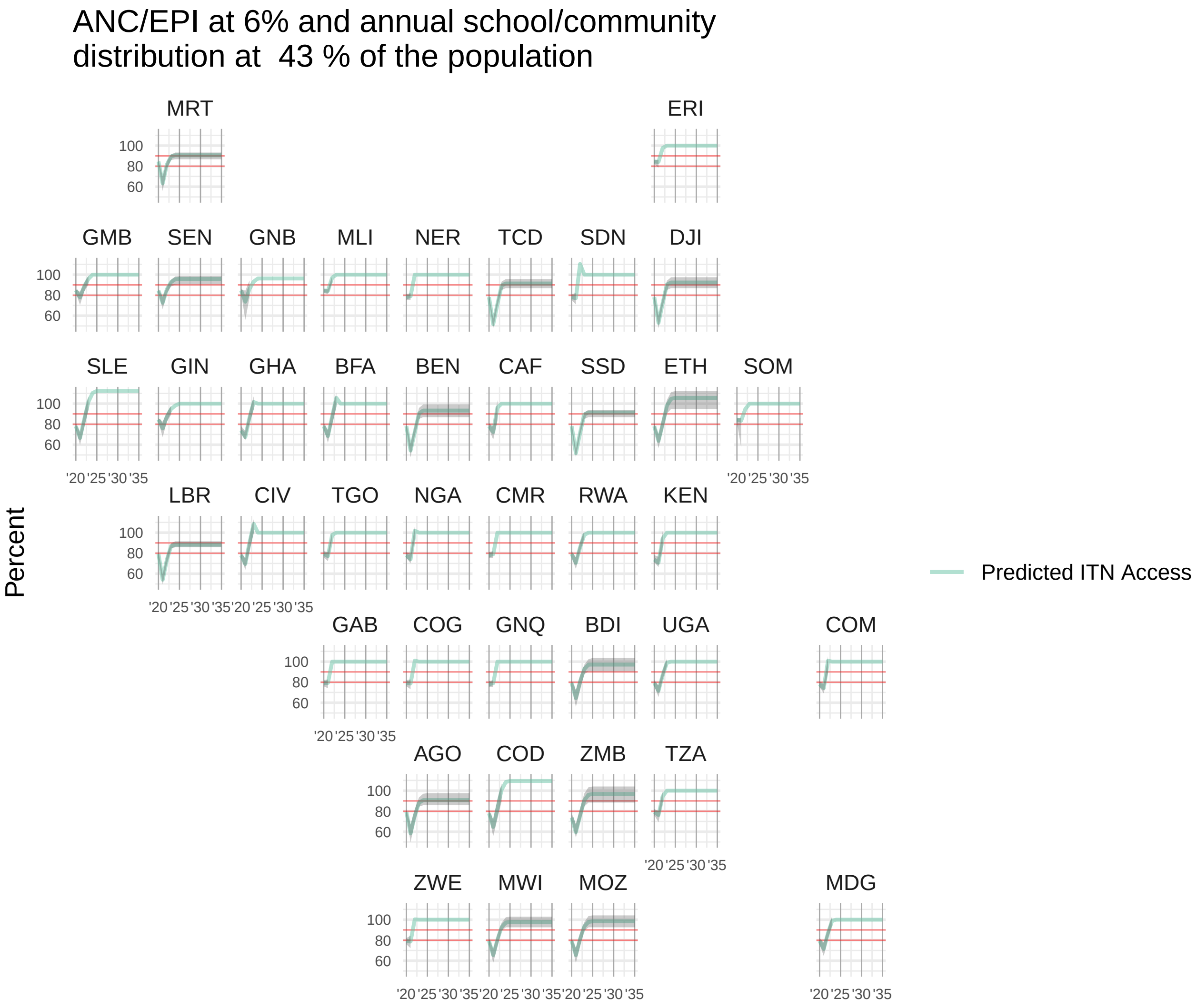
ANC/EPI at 6% and annual school/community distribution at 43 % of the population
MRT
ERI
GMB
SEN
GNB
MLI
NER
TCD
SDN
DJI
SLE
GIN
GHA
BFA
BEN
CAF
SSD
ETH
SOM
LBR
CIV
TGO
NGA
CMR
RWA
KEN
GAB
COG
GNQ
BDI
UGA
COM
AGO
COD
ZMB
TZA
ZWE
MWI
MOZ
MDG
Percent
100
80
60
'20 '25 '30 '35
Predicted ITN Access

ANC/EPI at 6% and annual school/community distribution at 44 % of the population

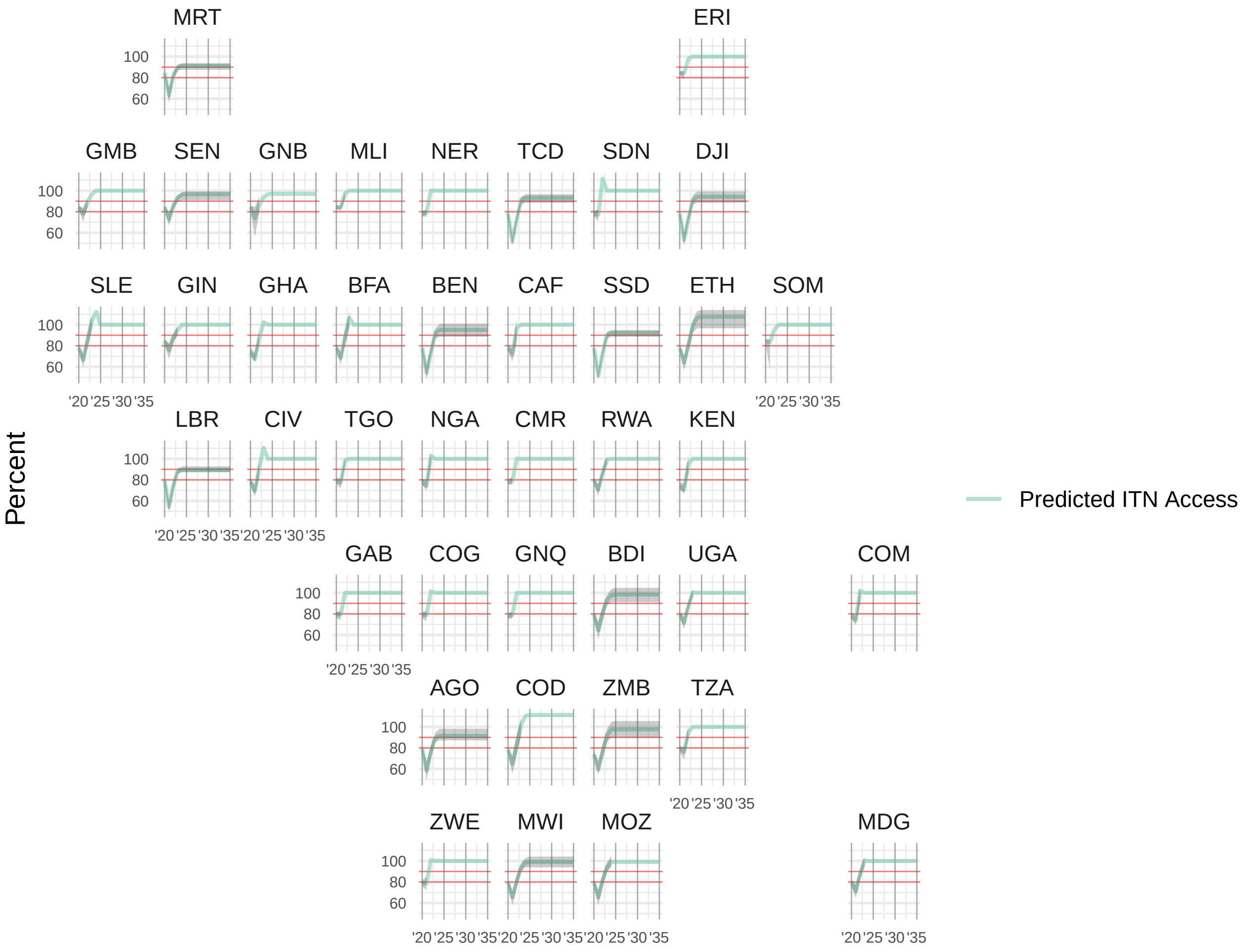

ANC/EPI at 6% and annual school/community distribution at 45 % of the population

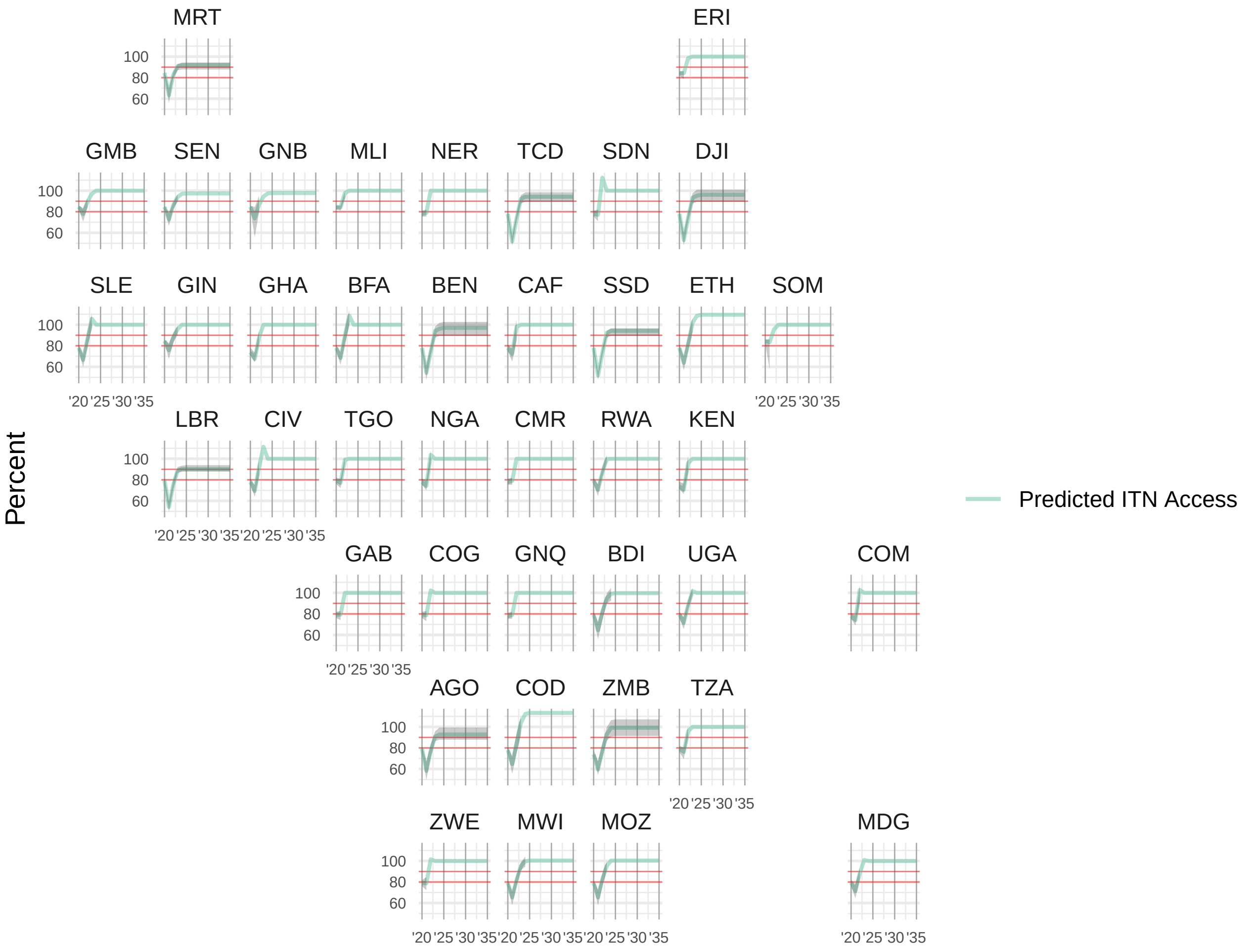

ANC/EPI at 6% and annual school/community distribution at 46 % of the population

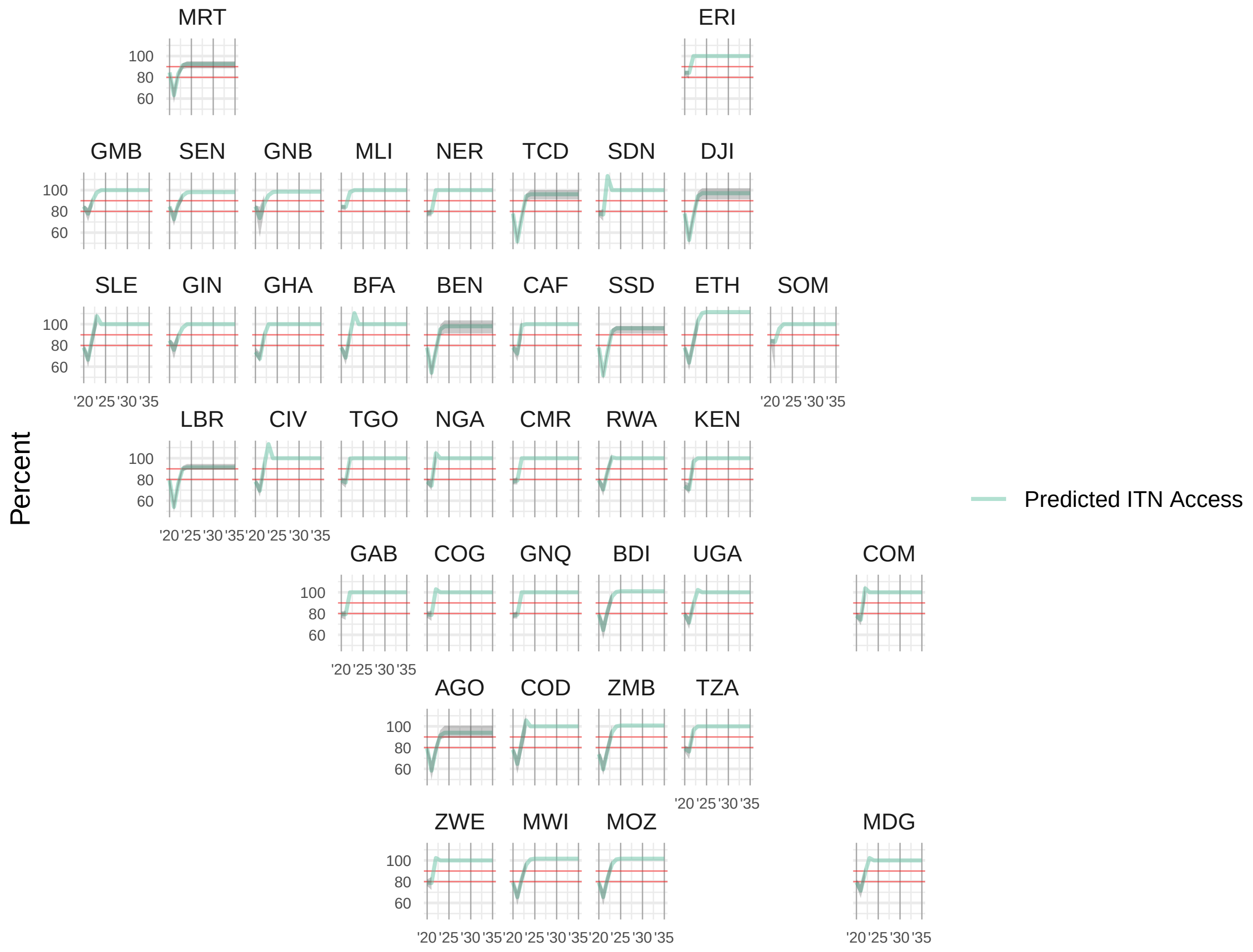

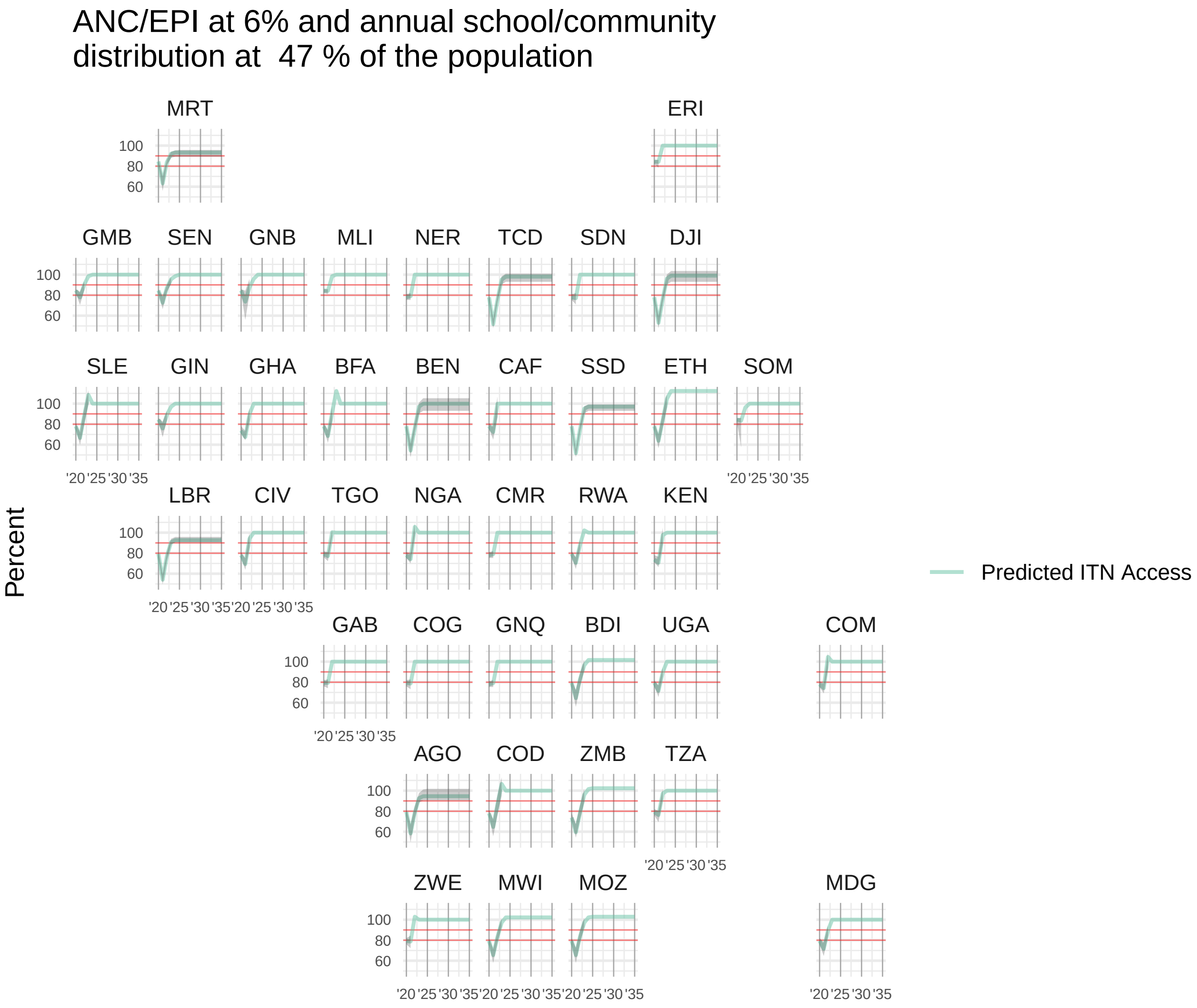
ANC/EPI at 6% and annual school/community distribution at 47 % of the population
Percent
Predicted ITN Access
MRT
ERI
GMB
SEN
GNB
MLI
NER
TCD
SDN
DJI
SLE
GIN
GHA
BFA
BEN
CAF
SSD
ETH
SOM
LBR
CIV
TGO
NGA
CMR
RWA
KEN
GAB
COG
GNQ
BDI
UGA
COM
AGO
COD
ZMB
TZA
ZWE
MWI
MOZ
MDG
100
80
60
'20 '25 '30 '35

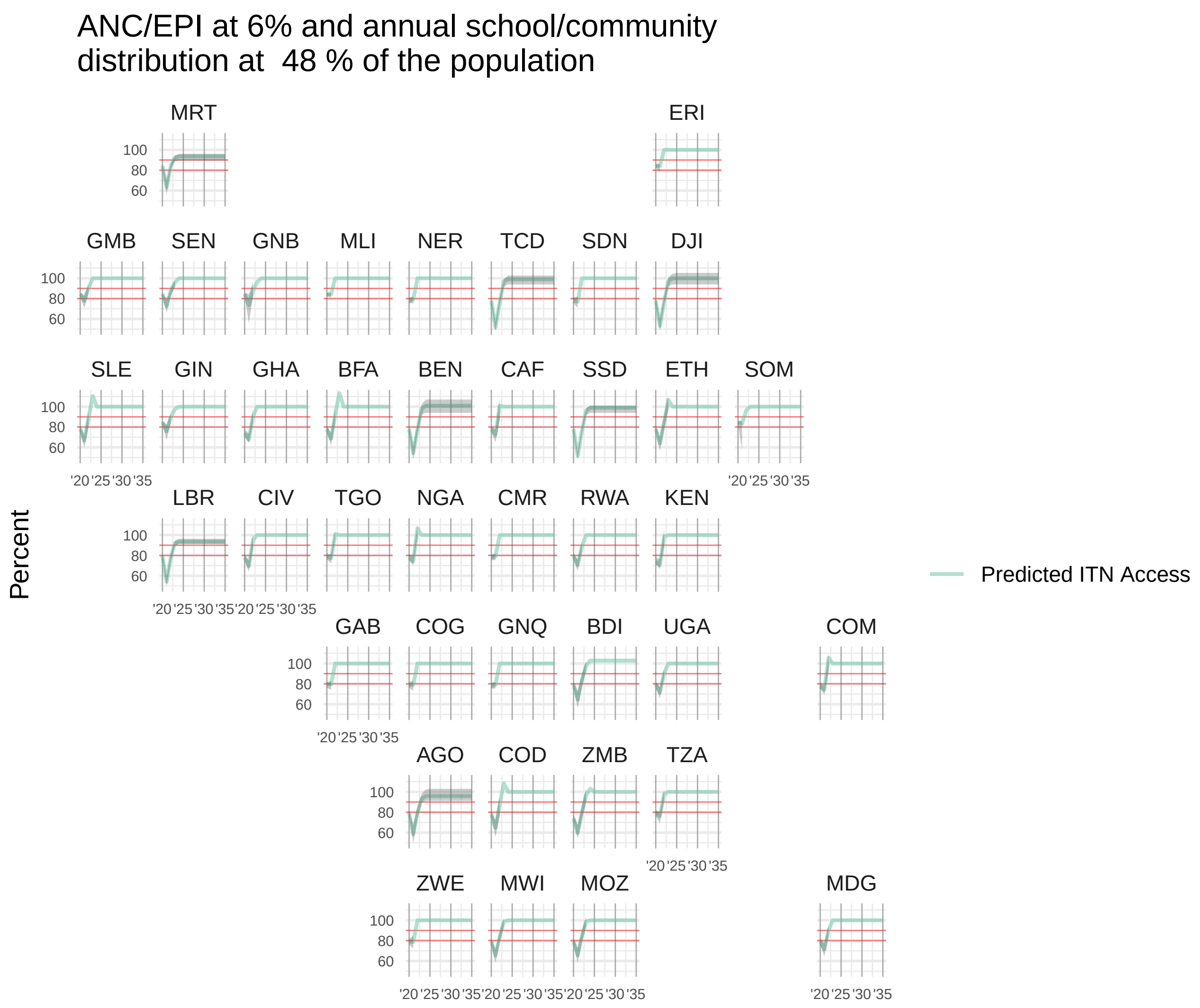
ANC/EPI at 6% and annual school/community distribution at 48 % of the population
MRT
ERI
GMB
SEN
GNB
MLI
NER
TCD
SDN
DJI
SLE
GIN
GHA
BFA
BEN
CAF
SSD
ETH
SOM
LBR
CIV
TGO
NGA
CMR
RWA
KEN
GAB
COG
GNQ
BDI
UGA
COM
AGO
COD
ZMB
TZA
ZWE
MWI
MOZ
MDG
100
80
60
'20 '25 '30 '35
Percent
Predicted ITN Access

ANC/EPI at 6% and annual school/community distribution at 49 % of the population

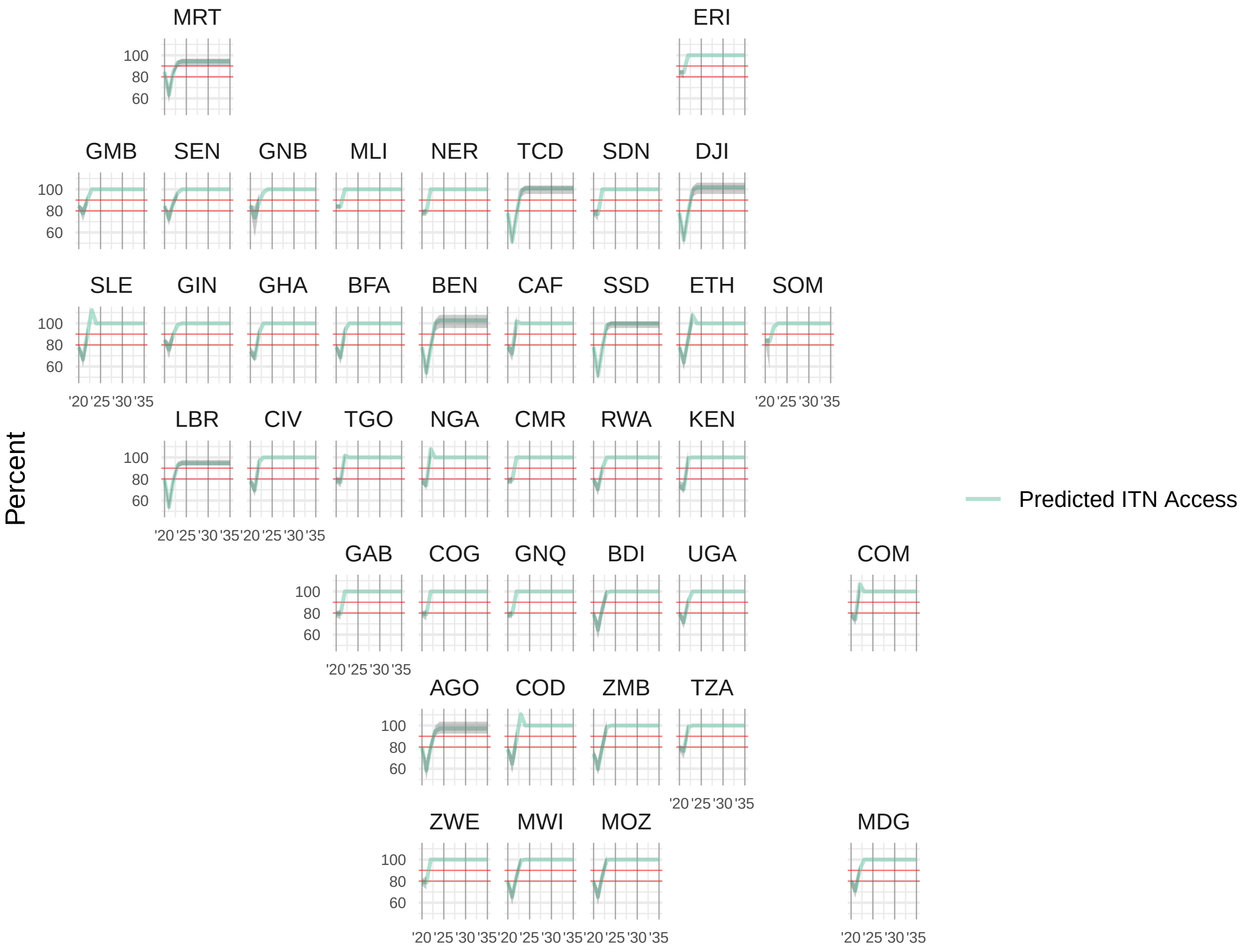

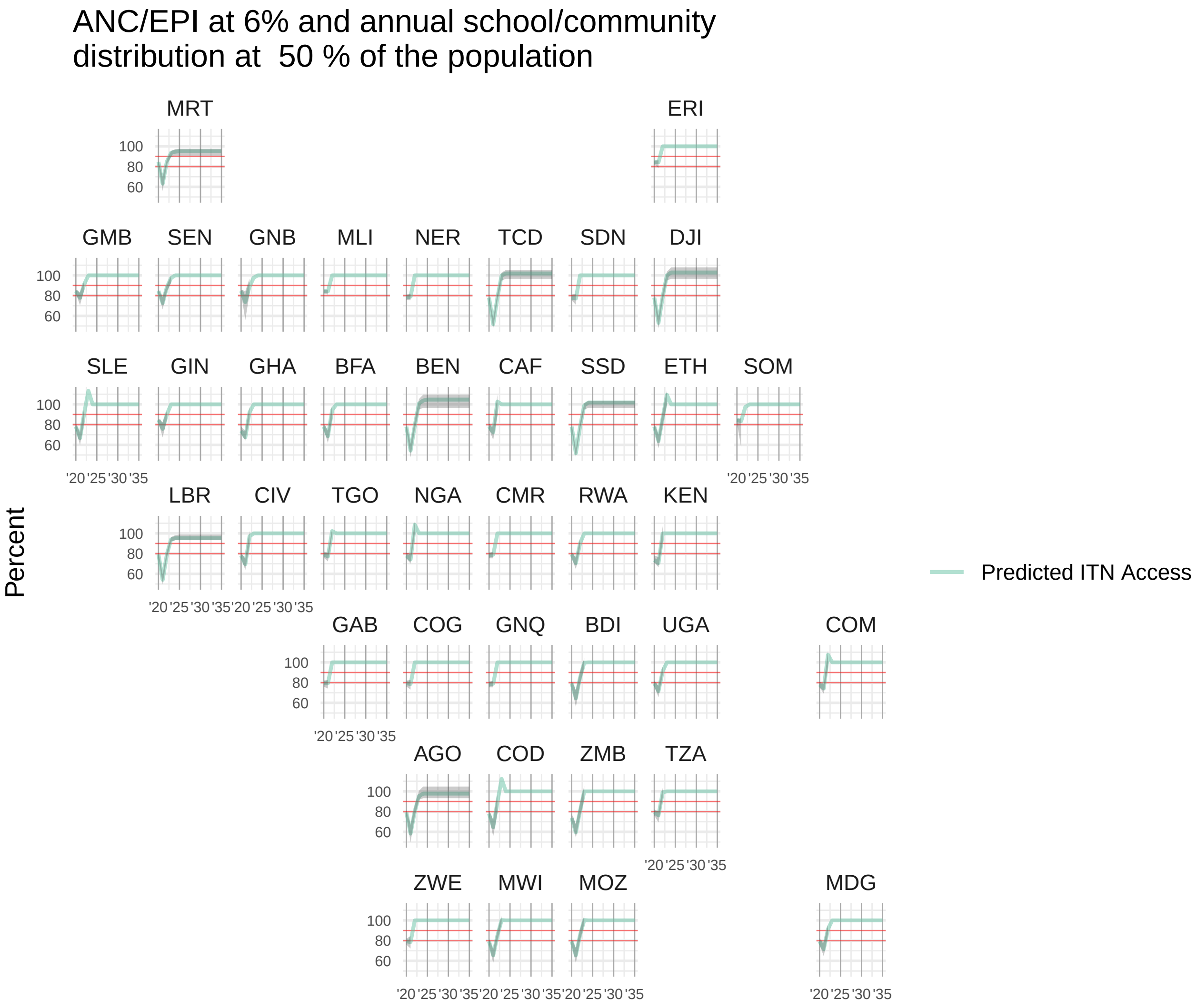
ANC/EPI at 6% and annual school/community distribution at 50 % of the population
Percent
MRT
ERI
GMB
SEN
GNB
MLI
NER
TCD
SDN
DJI
SLE
GIN
GHA
BFA
BEN
CAF
SSD
ETH
SOM
LBR
CIV
TGO
NGA
CMR
RWA
KEN
GAB
COG
GNQ
BDI
UGA
COM
AGO
COD
ZMB
TZA
ZWE
MWI
MOZ
MDG
100
80
60
'20 '25 '30 '35
Predicted ITN Access

3 Scenario 3 - Three-year mass campaigns with ANC/EPI distribution at 6%, varying annual school/community distribution between campaigns

3−year mass campaigns with ANC/EPI at 6%
and between−campaign school/community distribution
at 0 % of the population

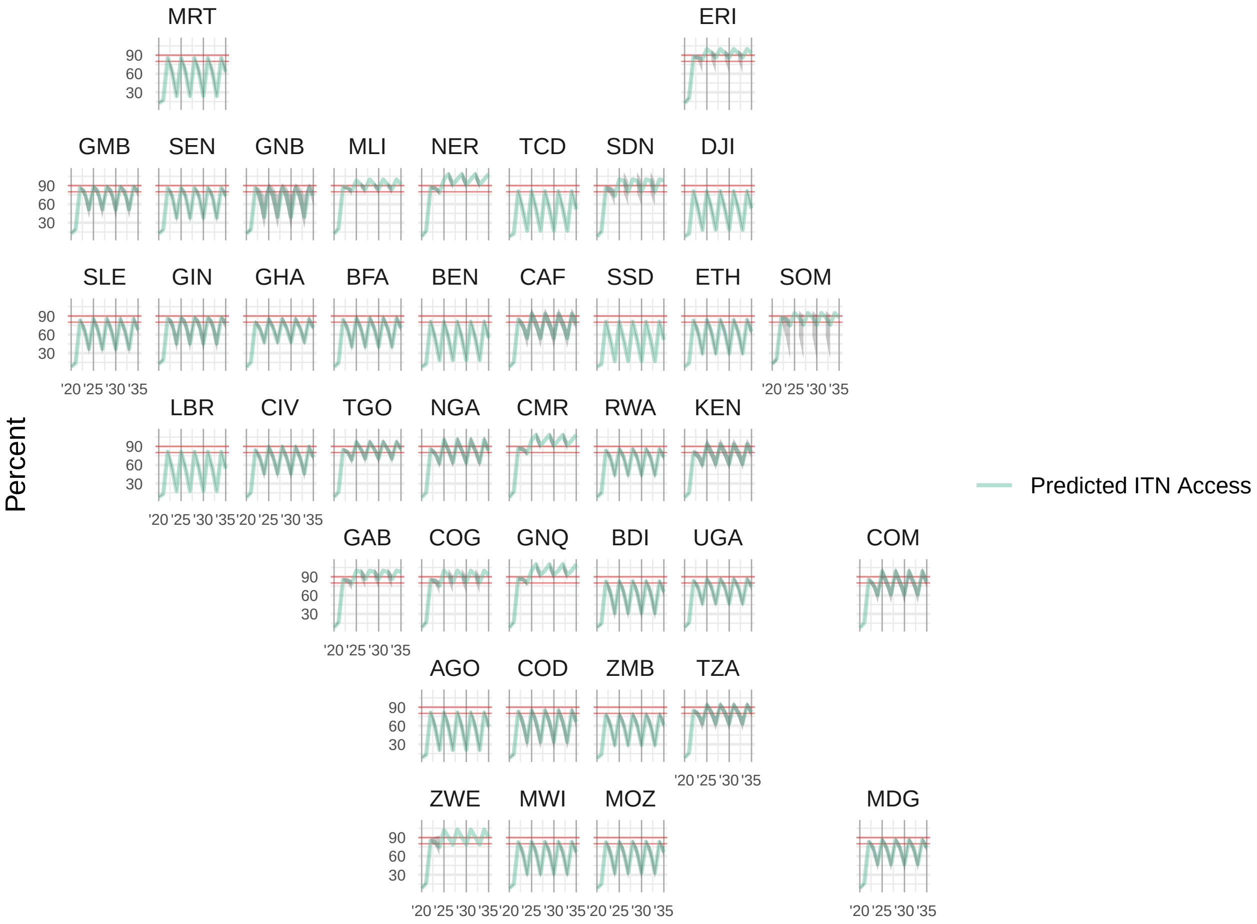

3−year mass campaigns with ANC/EPI at 6%
and between−campaign school/community distribution
at 1 % of the population

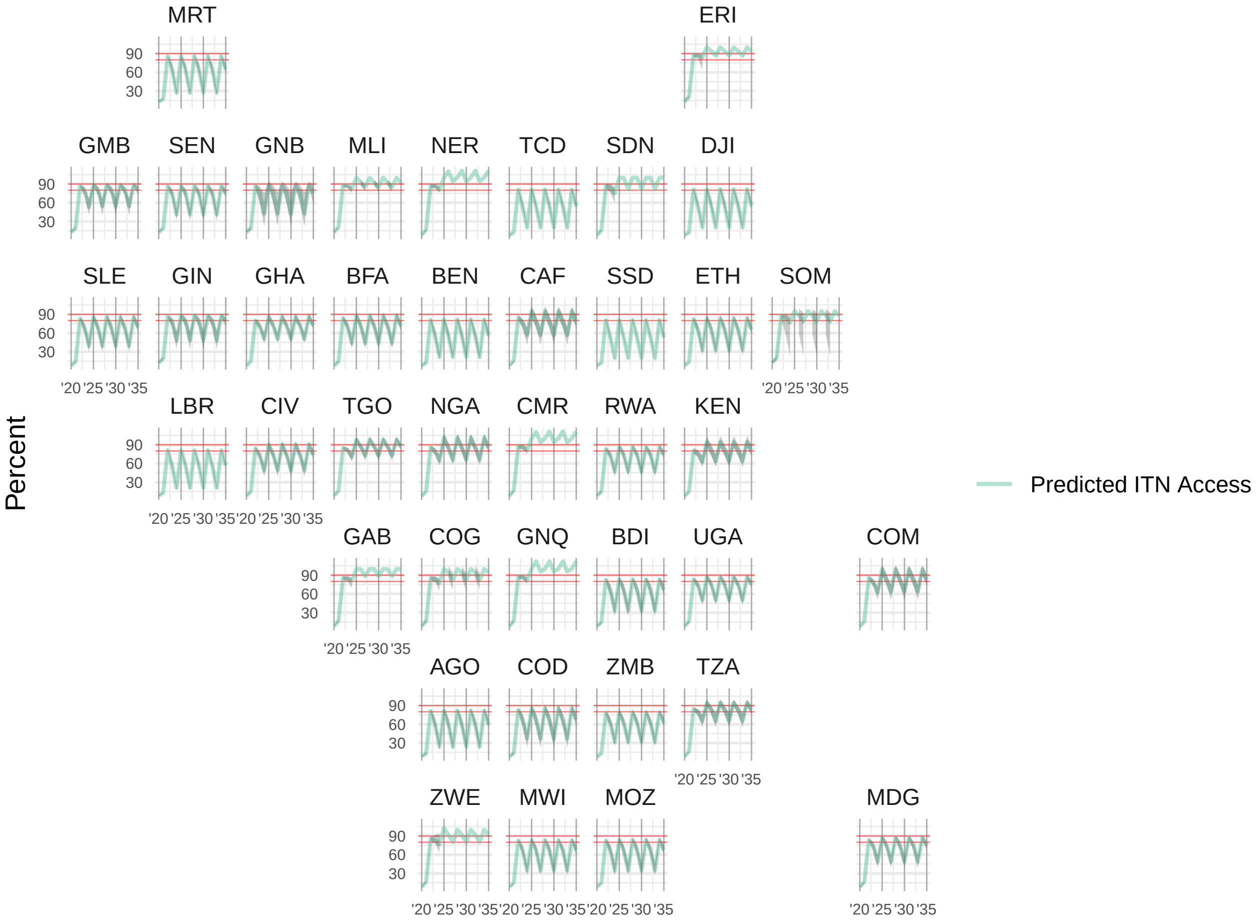

3−year mass campaigns with ANC/EPI at 6%
and between−campaign school/community distribution
at 2 % of the population

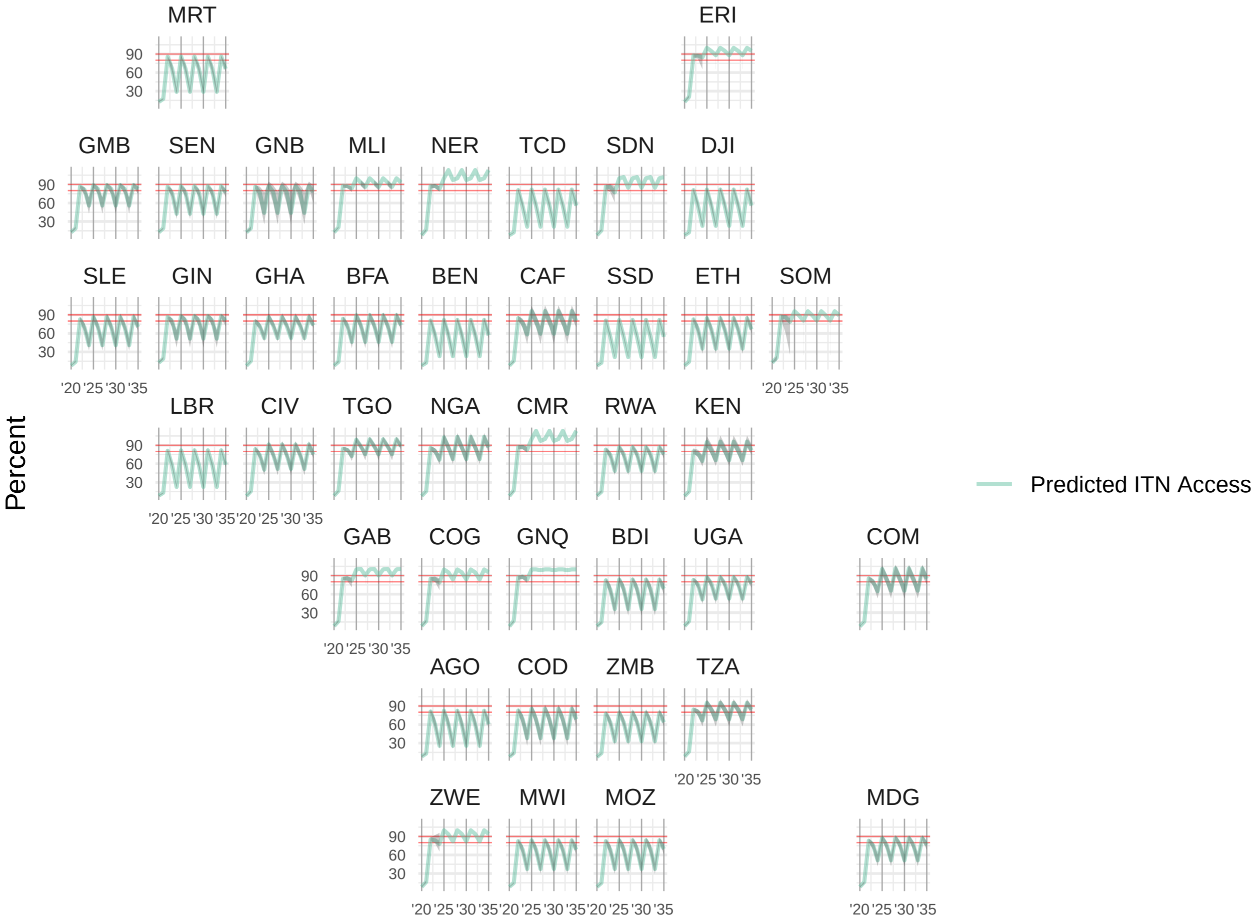

3−year mass campaigns with ANC/EPI at 6%
and between−campaign school/community distribution
at 3 % of the population

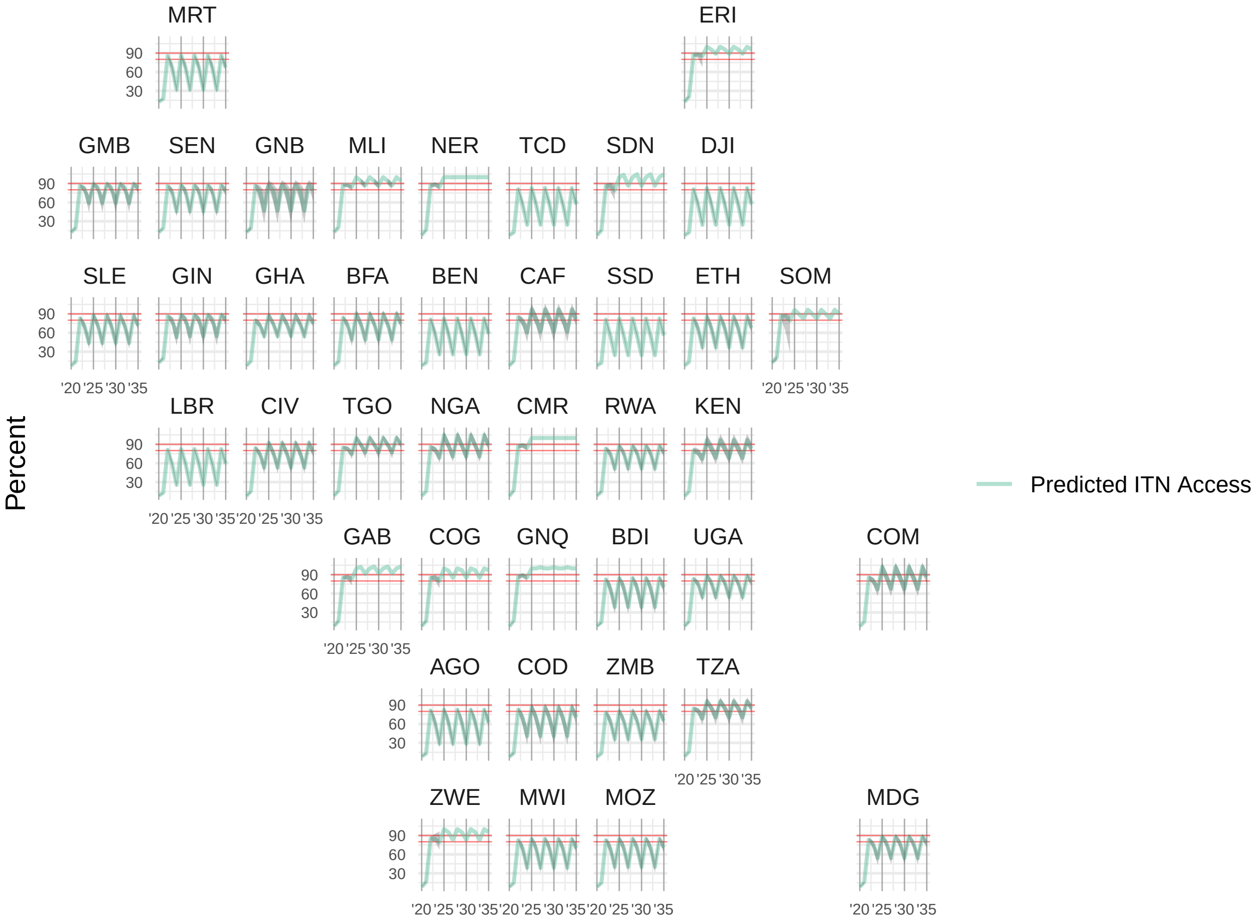

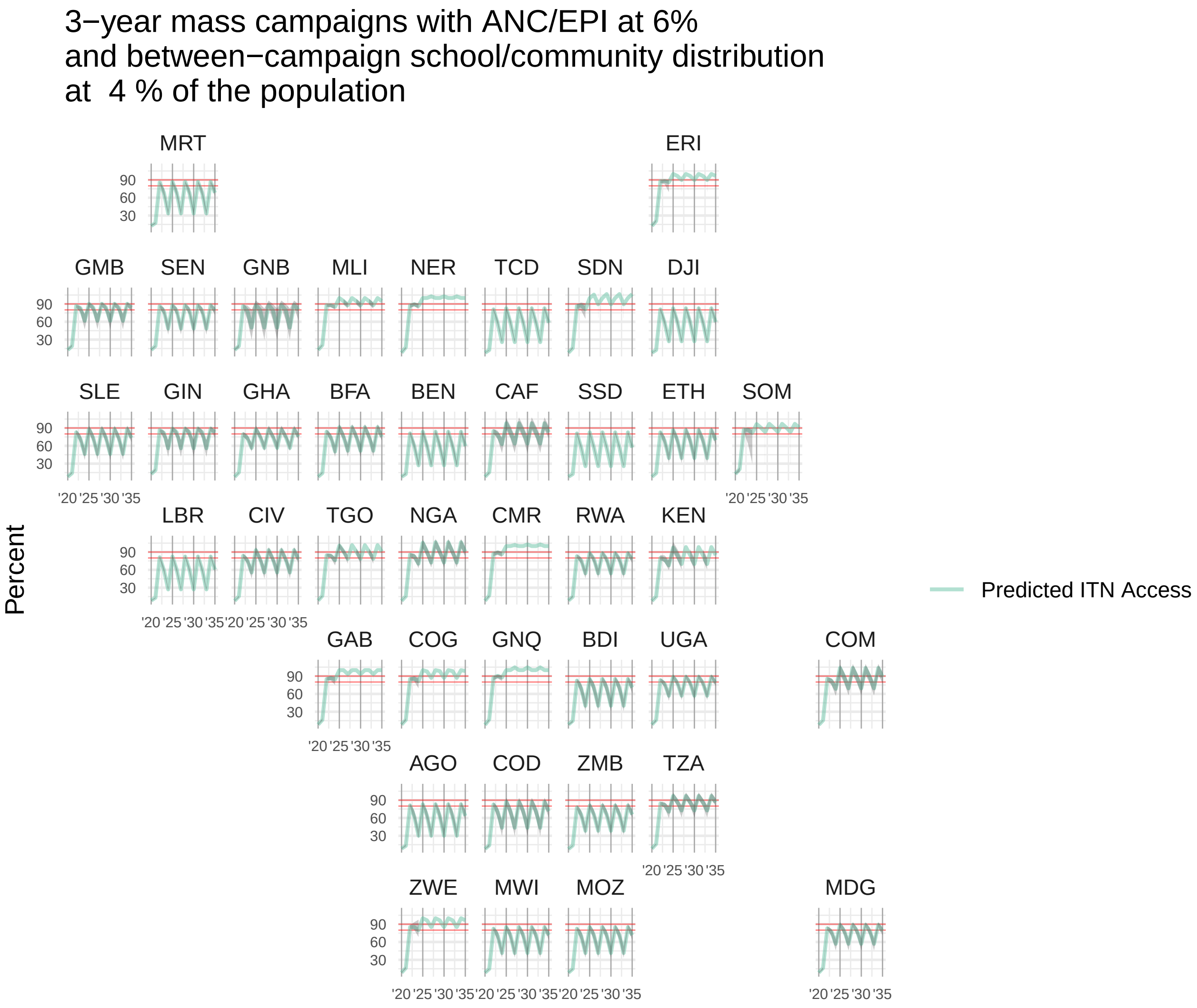
3−year mass campaigns with ANC/EPI at 6%
and between−campaign school/community distribution
at 4 % of the population
MRT
ERI
GMB
SEN
GNB
MLI
NER
TCD
SDN
DJI
SLE
GIN
GHA
BFA
BEN
CAF
SSD
ETH
SOM
LBR
CIV
TGO
NGA
CMR
RWA
KEN
GAB
COG
GNQ
BDI
UGA
COM
AGO
COD
ZMB
TZA
ZWE
MWI
MOZ
MDG
90
60
30
'20 '25 '30 '35
Percent
Predicted ITN Access

3−year mass campaigns with ANC/EPI at 6%
and between−campaign school/community distribution
at 5 % of the population

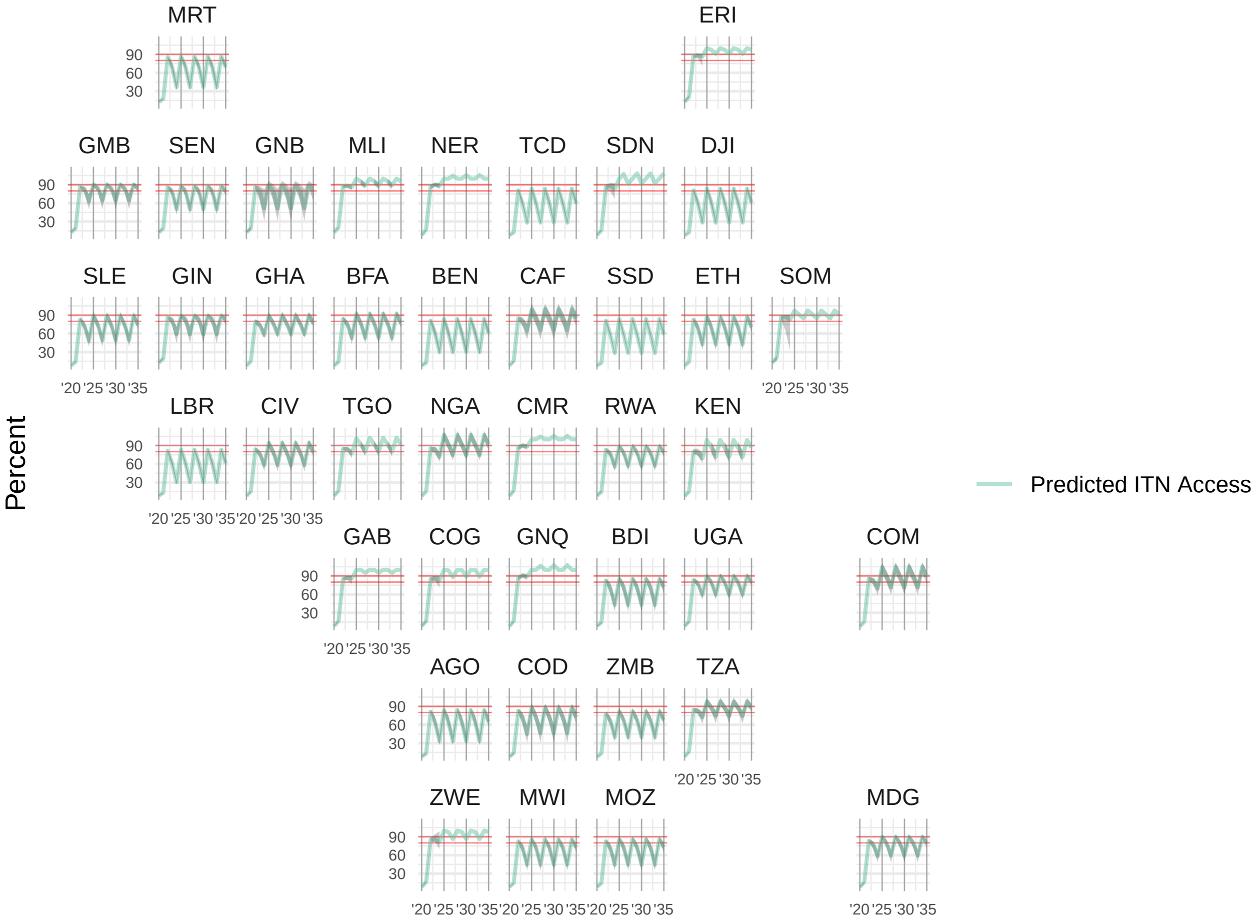

3−year mass campaigns with ANC/EPI at 6%
and between−campaign school/community distribution
at 6 % of the population

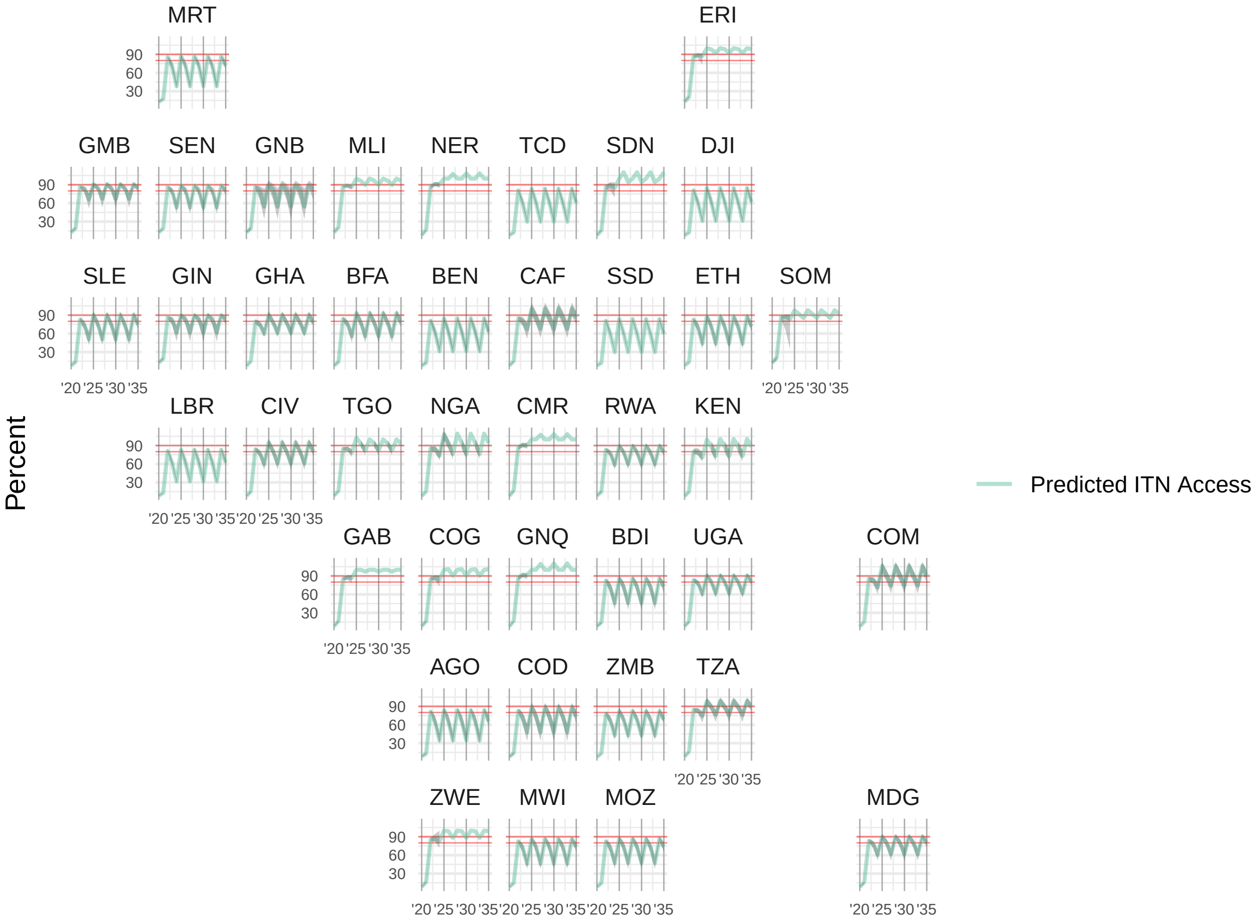

3−year mass campaigns with ANC/EPI at 6%
and between−campaign school/community distribution
at 7 % of the population

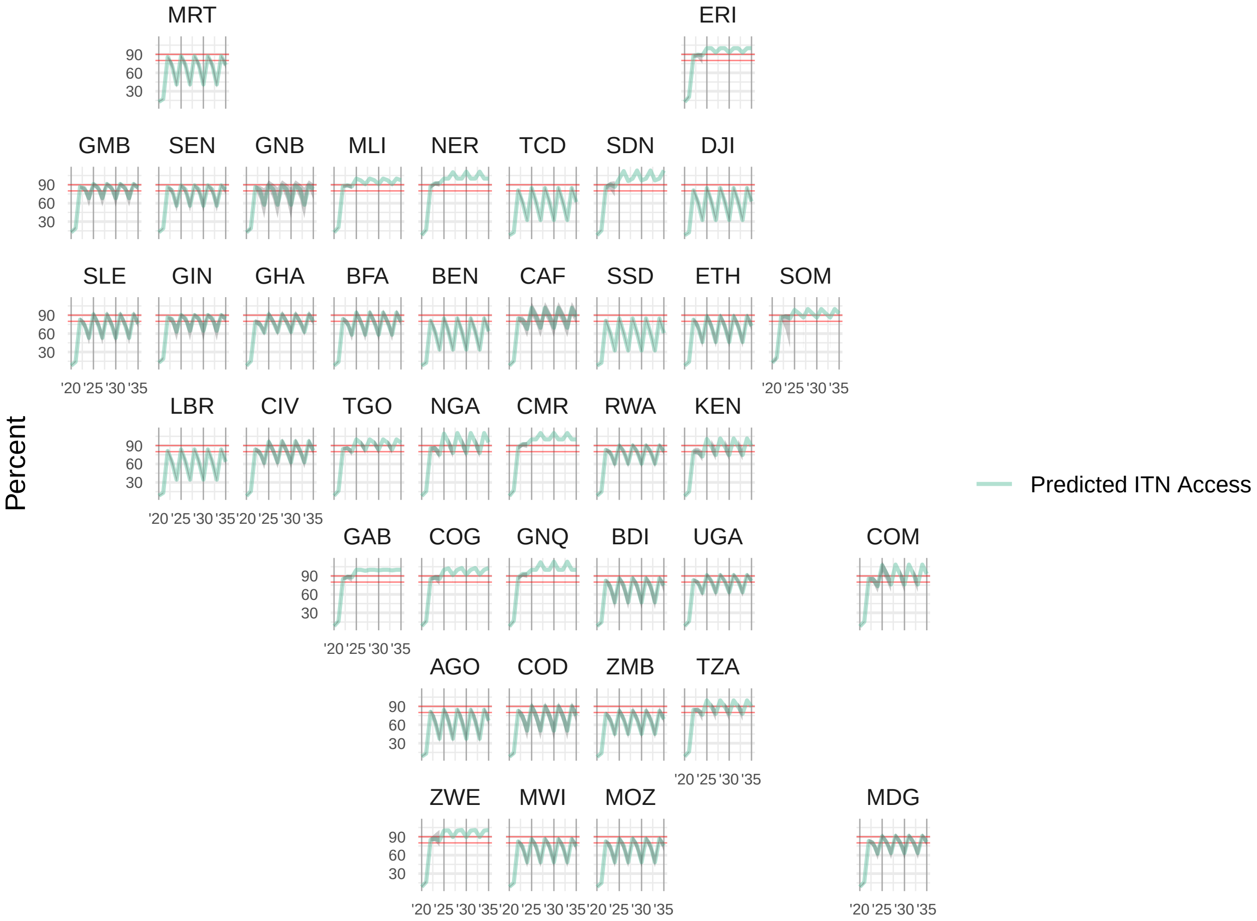

3−year mass campaigns with ANC/EPI at 6% and between−campaign school/community distribution at 8 % of the population

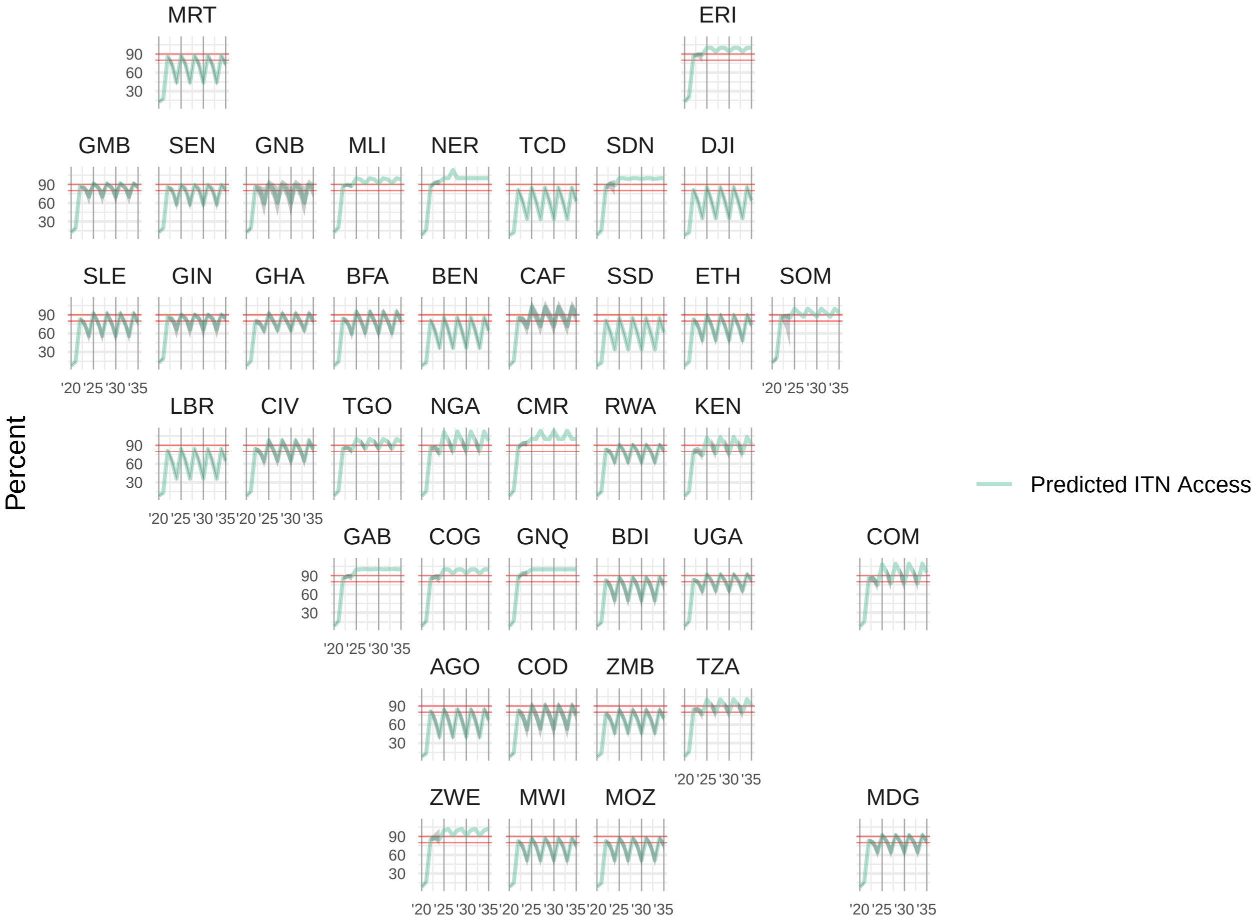

3−year mass campaigns with ANC/EPI at 6%
and between−campaign school/community distribution
at 9 % of the population

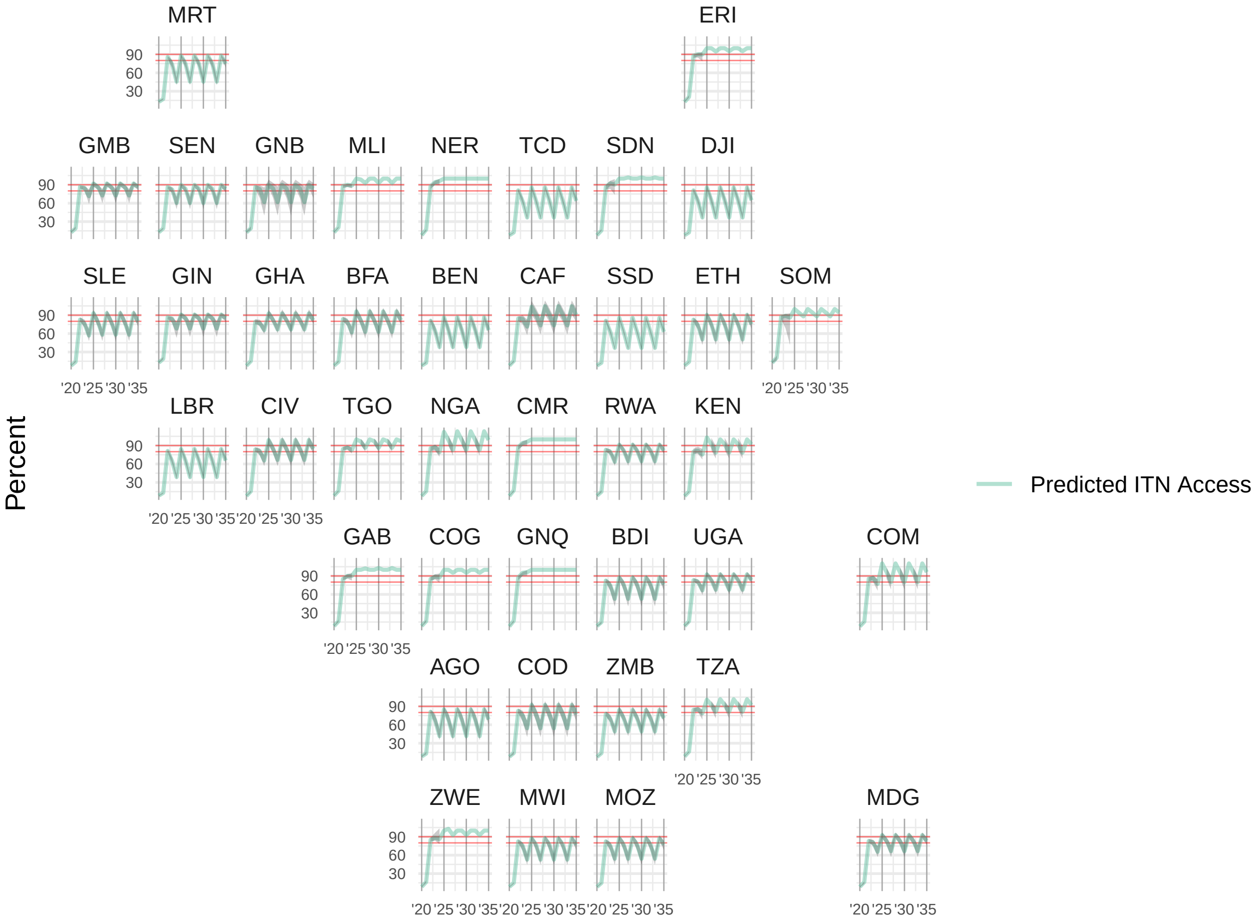

3−year mass campaigns with ANC/EPI at 6%
and between−campaign school/community distribution
at 10 % of the population

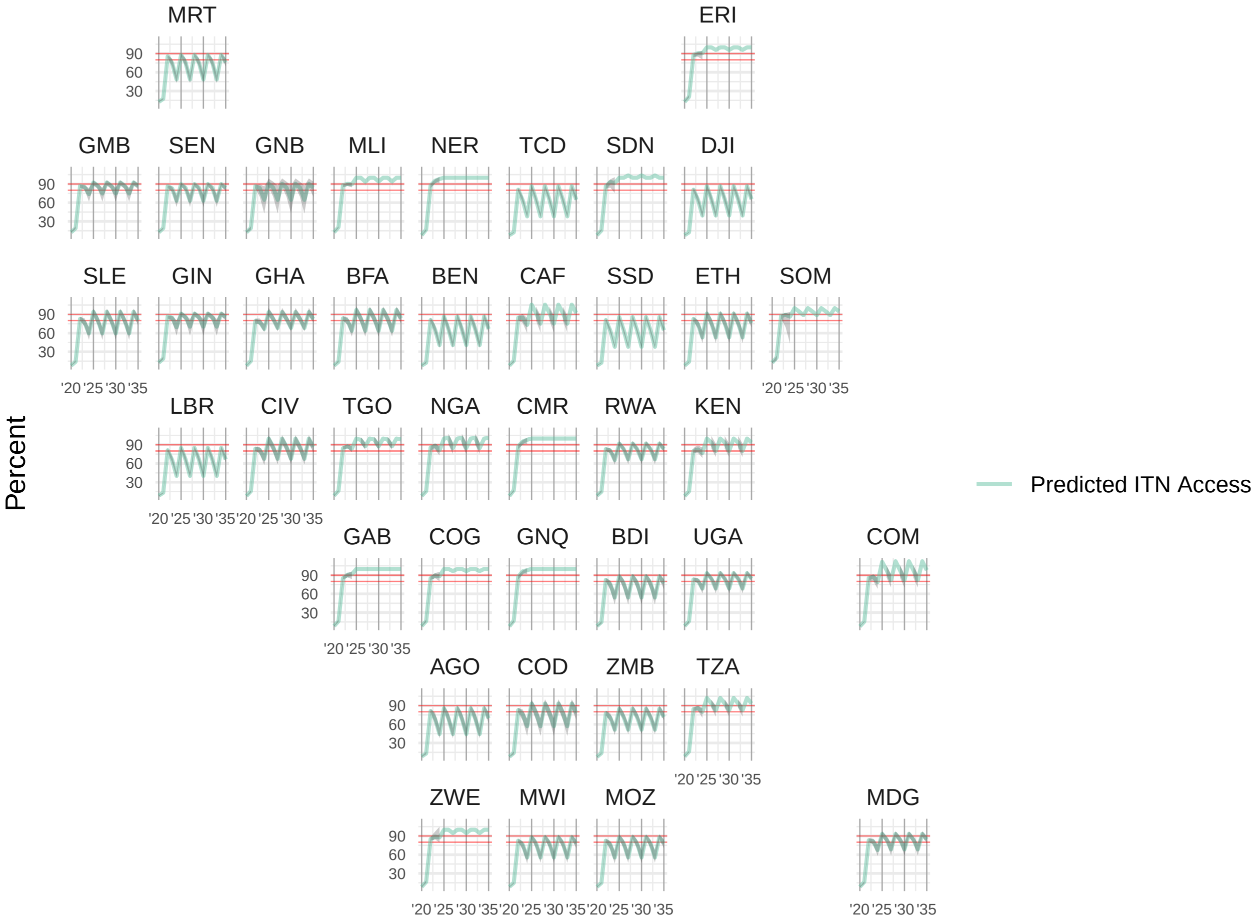

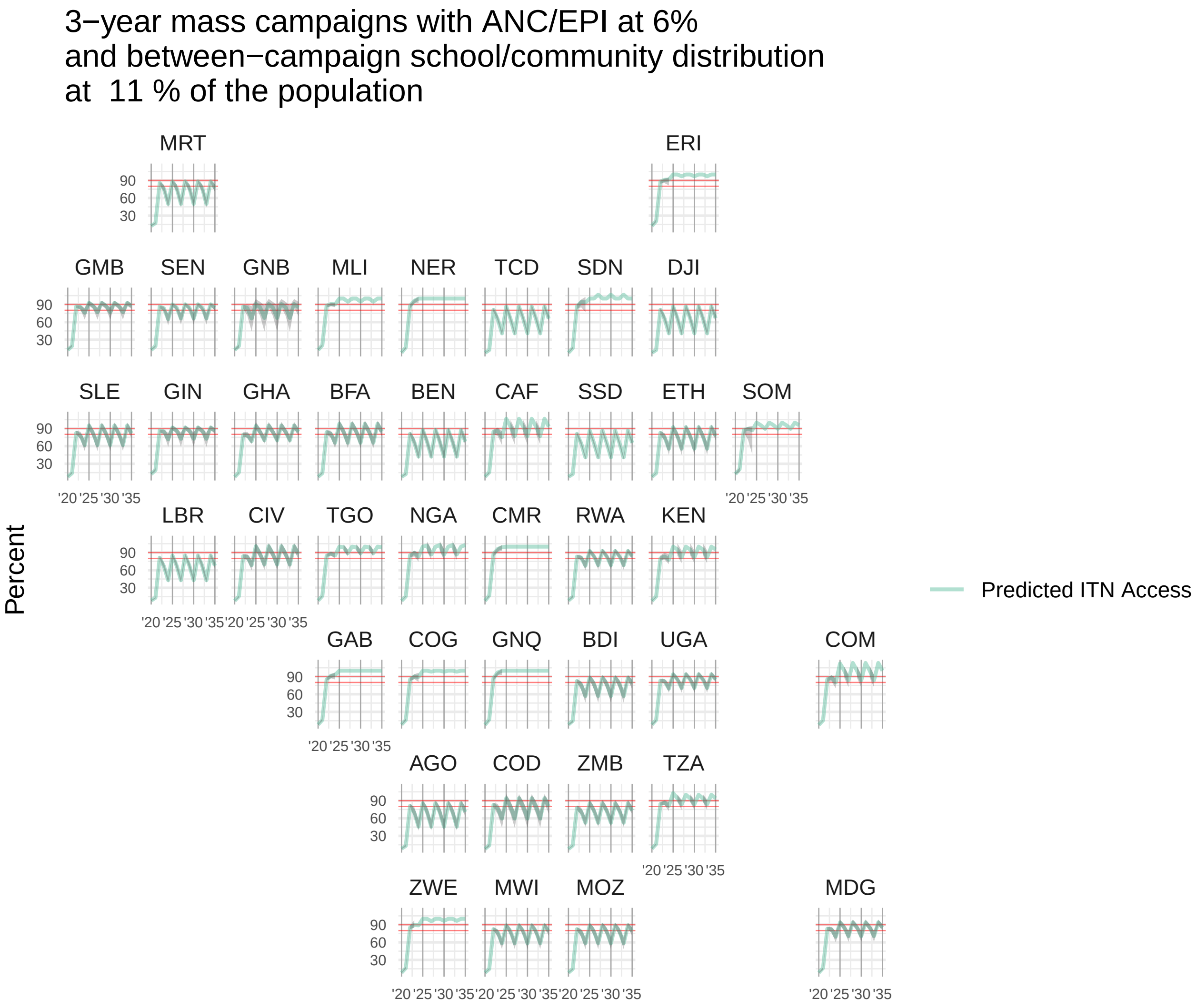
3−year mass campaigns with ANC/EPI at 6%
and between−campaign school/community distribution
at 11 % of the population
MRT
ERI
GMB
SEN
GNB
MLI
NER
TCD
SDN
DJI
SLE
GIN
GHA
BFA
BEN
CAF
SSD
ETH
SOM
LBR
CIV
TGO
NGA
CMR
RWA
KEN
GAB
COG
GNQ
BDI
UGA
COM
AGO
COD
ZMB
TZA
ZWE
MWI
MOZ
MDG
90
60
30
'20 '25 '30 '35
Percent
Predicted ITN Access

3−year mass campaigns with ANC/EPI at 6%
and between−campaign school/community distribution
at 12 % of the population

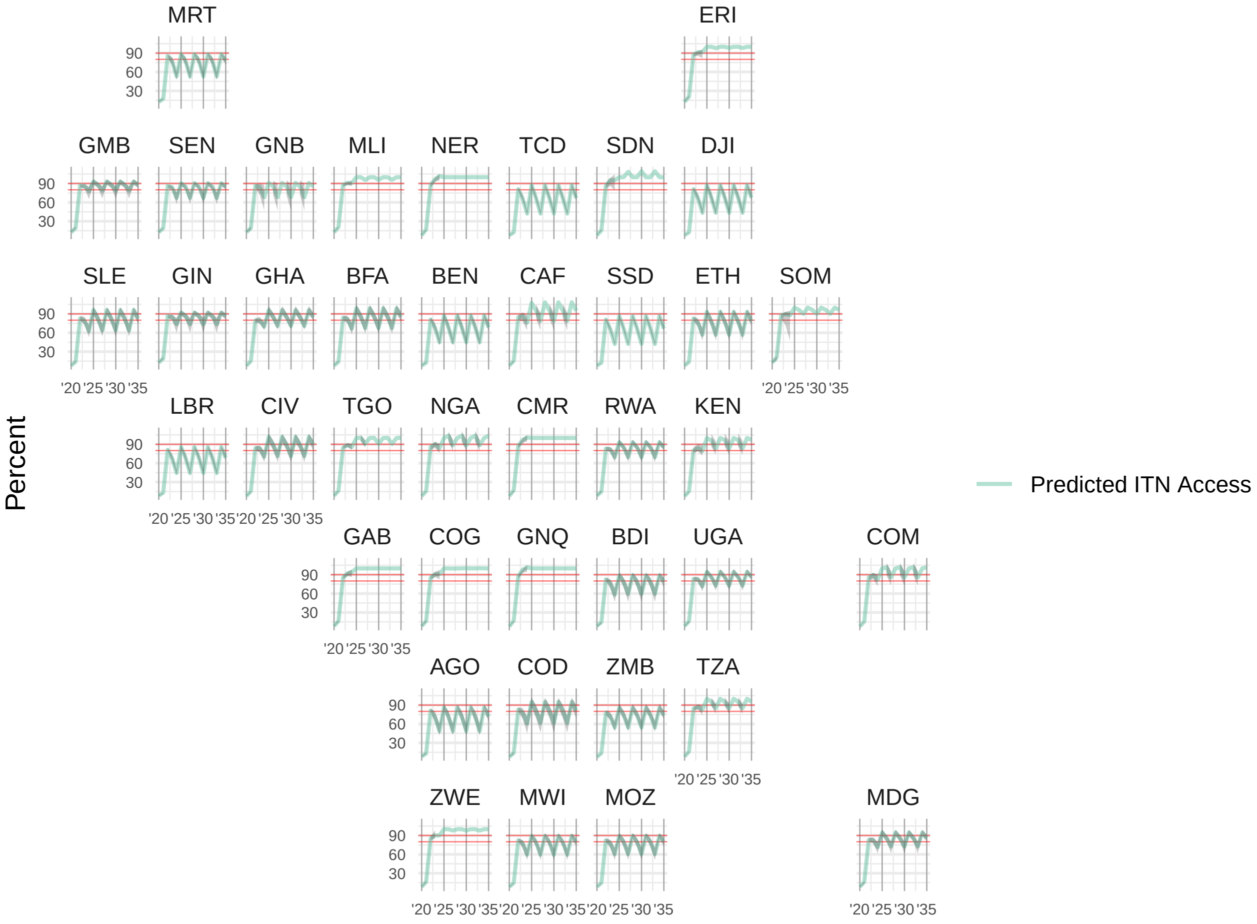

3−year mass campaigns with ANC/EPI at 6%
and between−campaign school/community distribution
at 13 % of the population

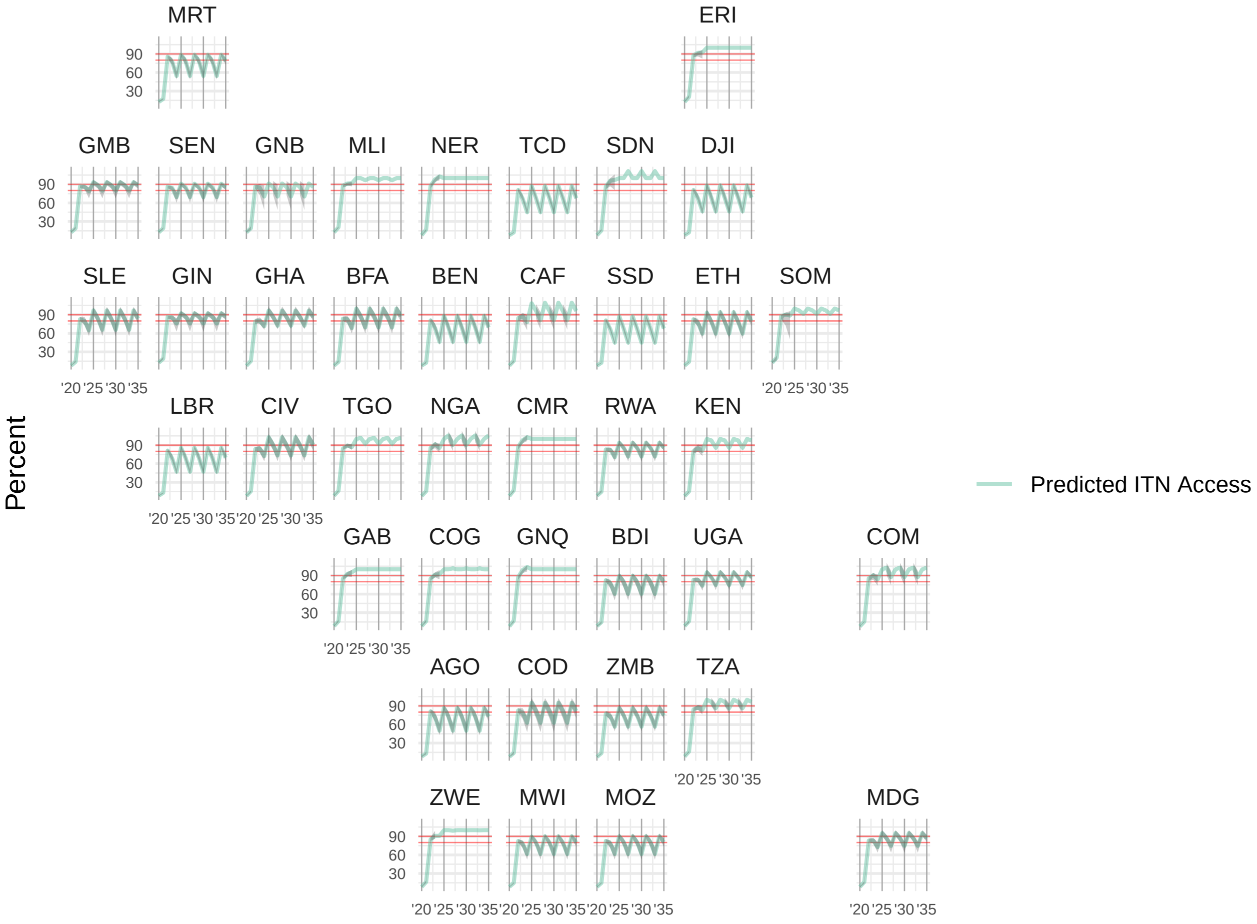

3−year mass campaigns with ANC/EPI at 6%
and between−campaign school/community distribution
at 14 % of the population

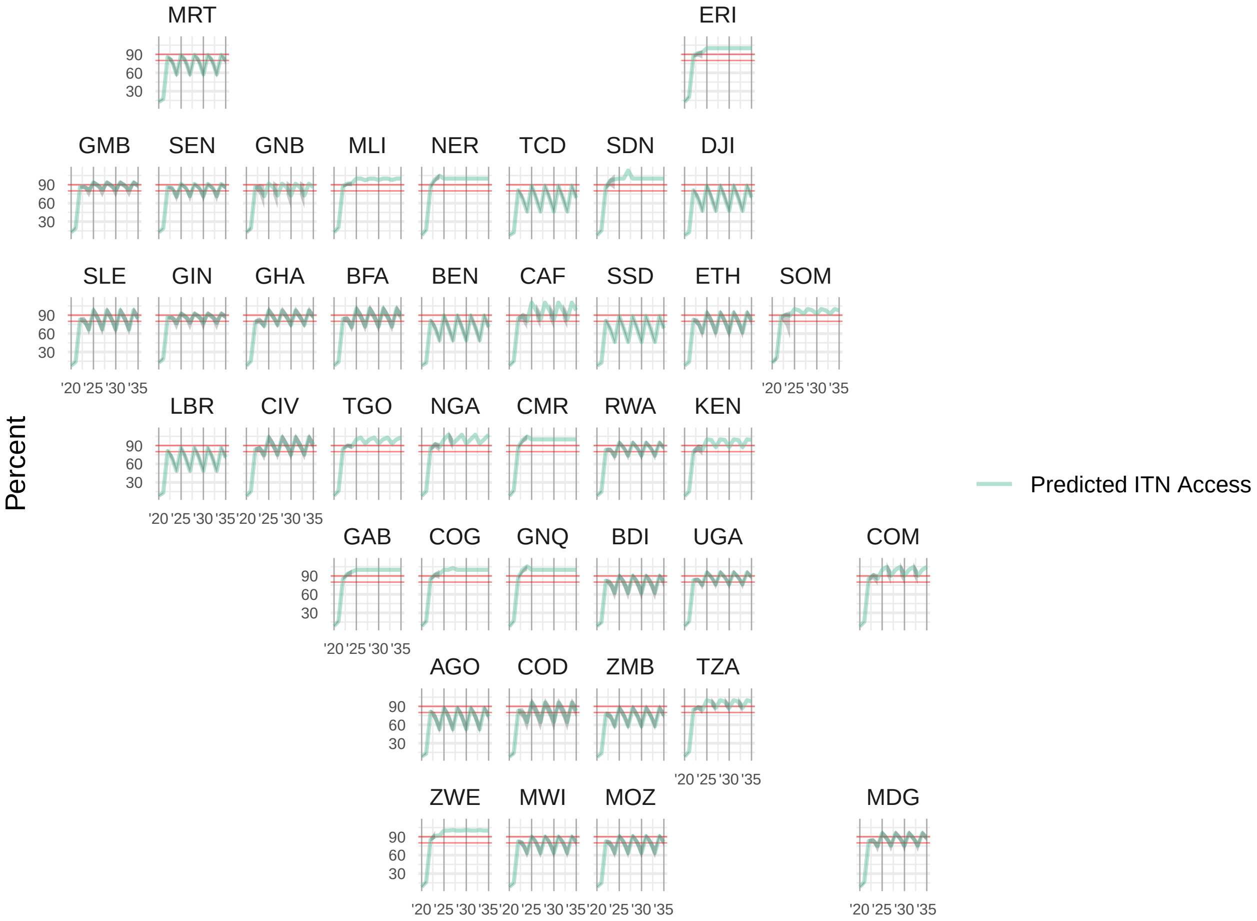

3−year mass campaigns with ANC/EPI at 6%
and between−campaign school/community distribution
at 15 % of the population

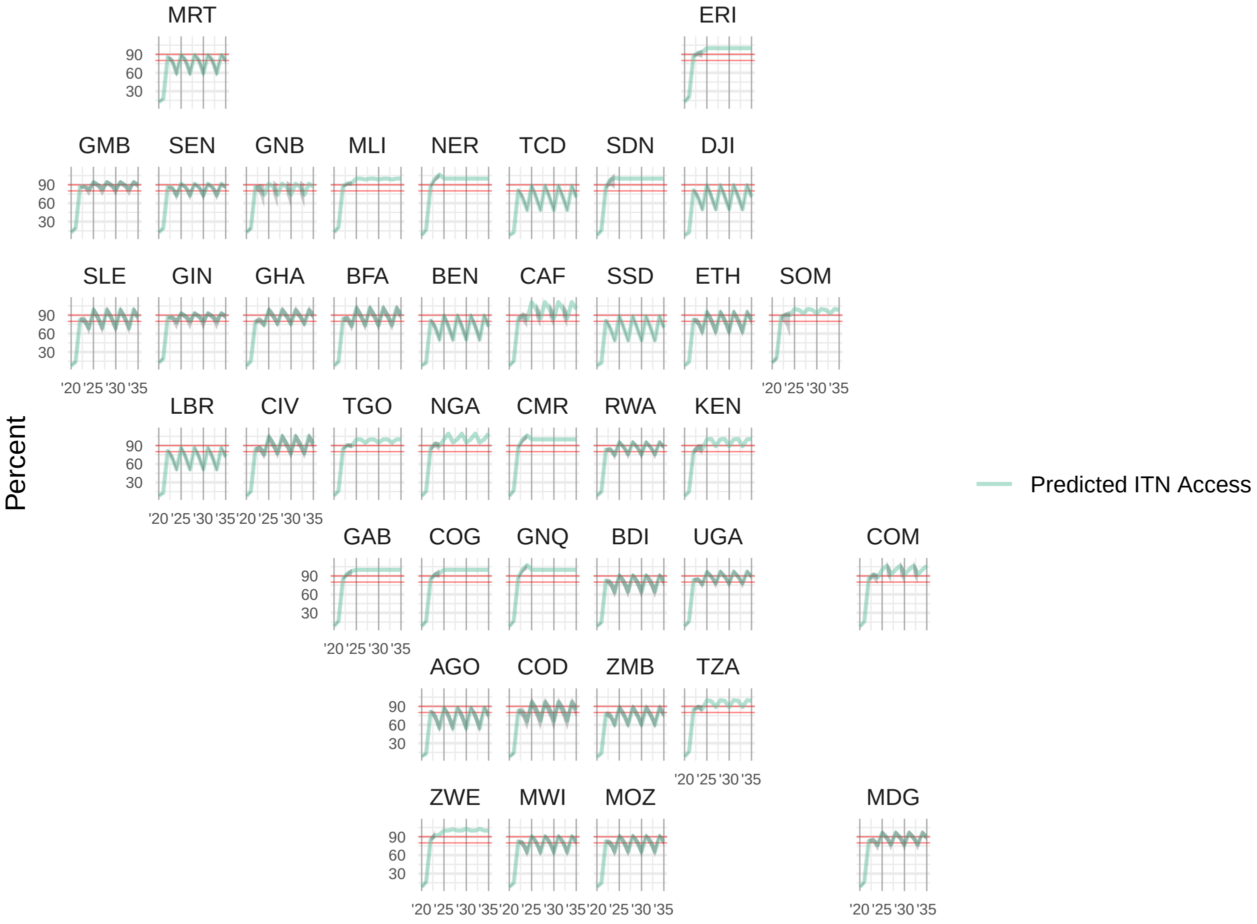

3−year mass campaigns with ANC/EPI at 6%
and between−campaign school/community distribution
at 16 % of the population

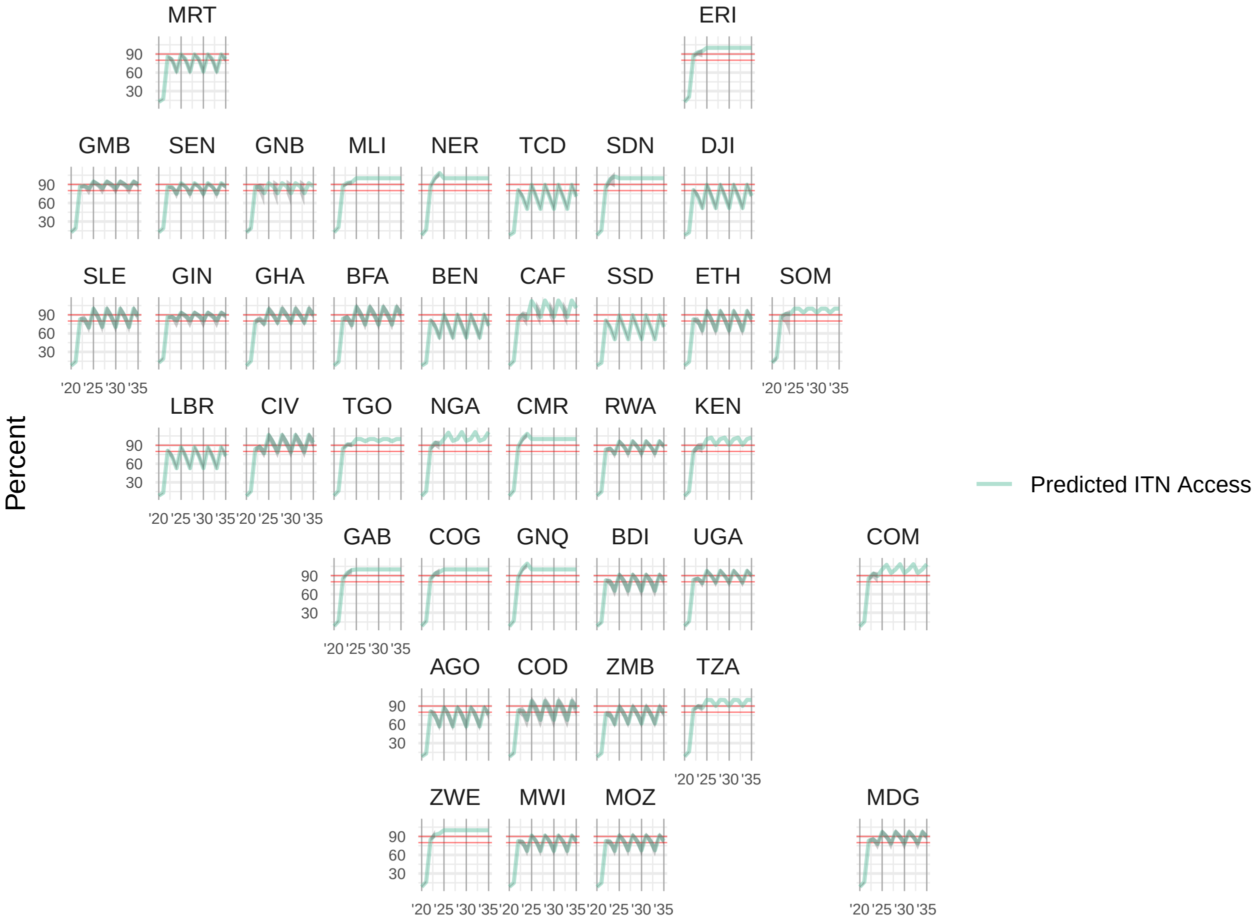

3−year mass campaigns with ANC/EPI at 6%
and between−campaign school/community distribution
at 17 % of the population

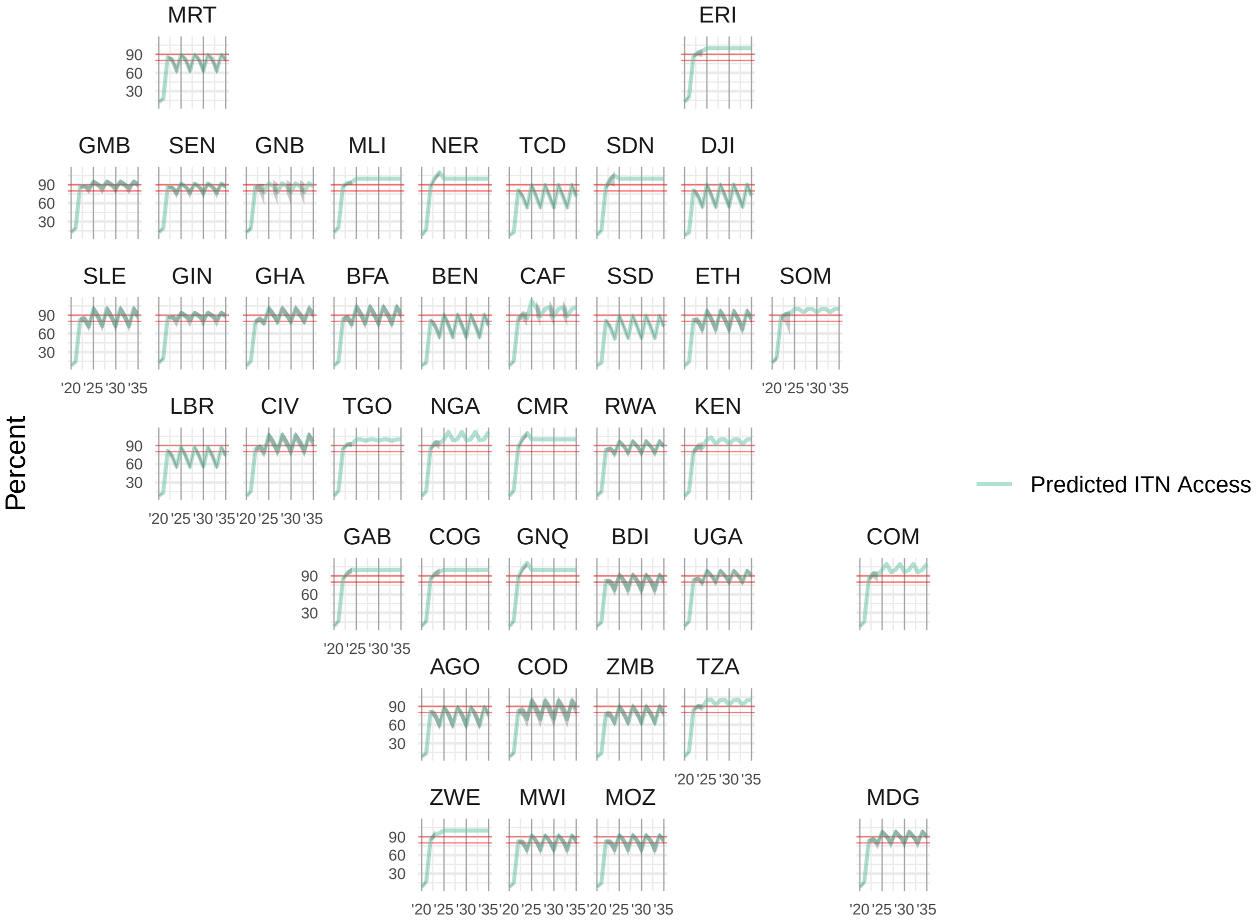

3−year mass campaigns with ANC/EPI at 6% and between−campaign school/community distribution at 18 % of the population

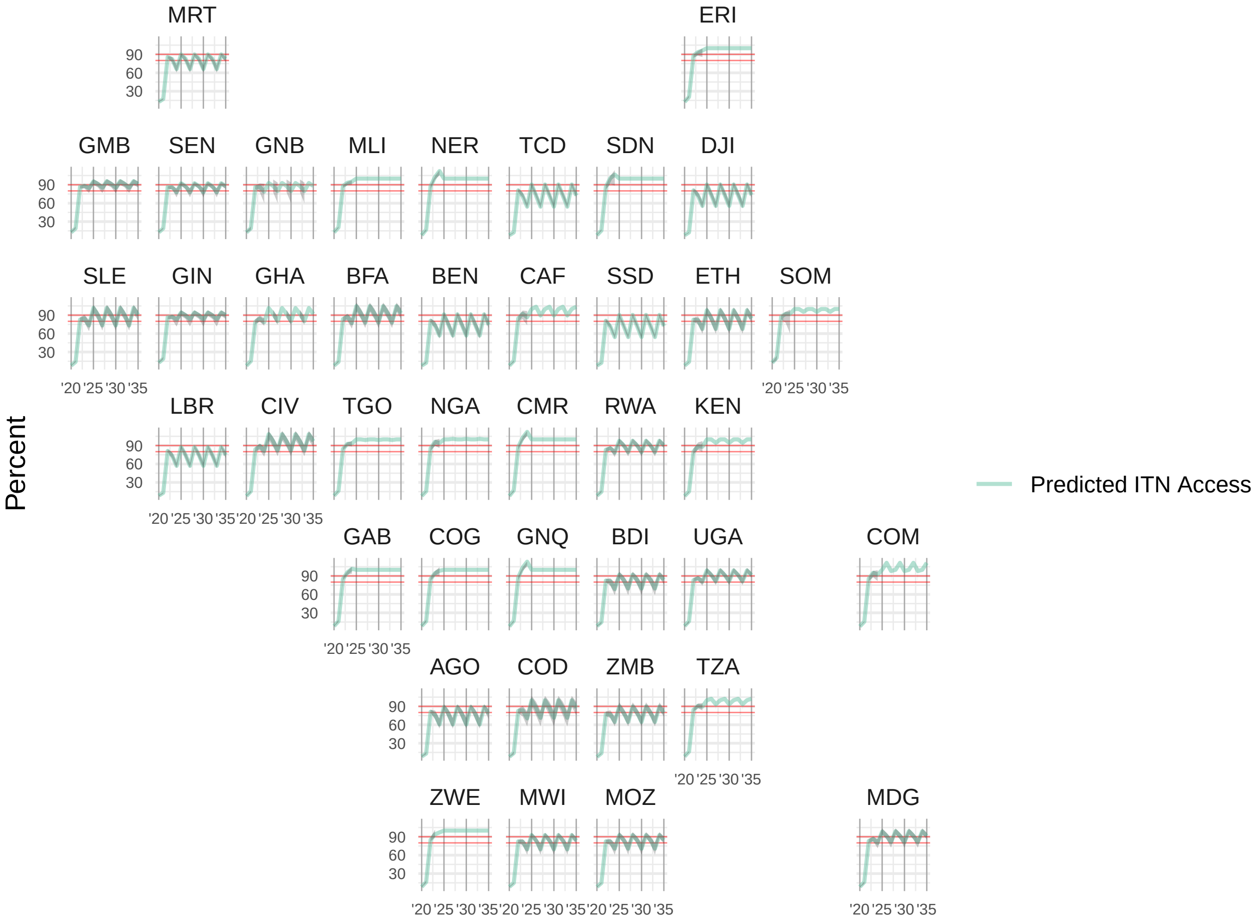

3−year mass campaigns with ANC/EPI at 6%
and between−campaign school/community distribution
at 19 % of the population

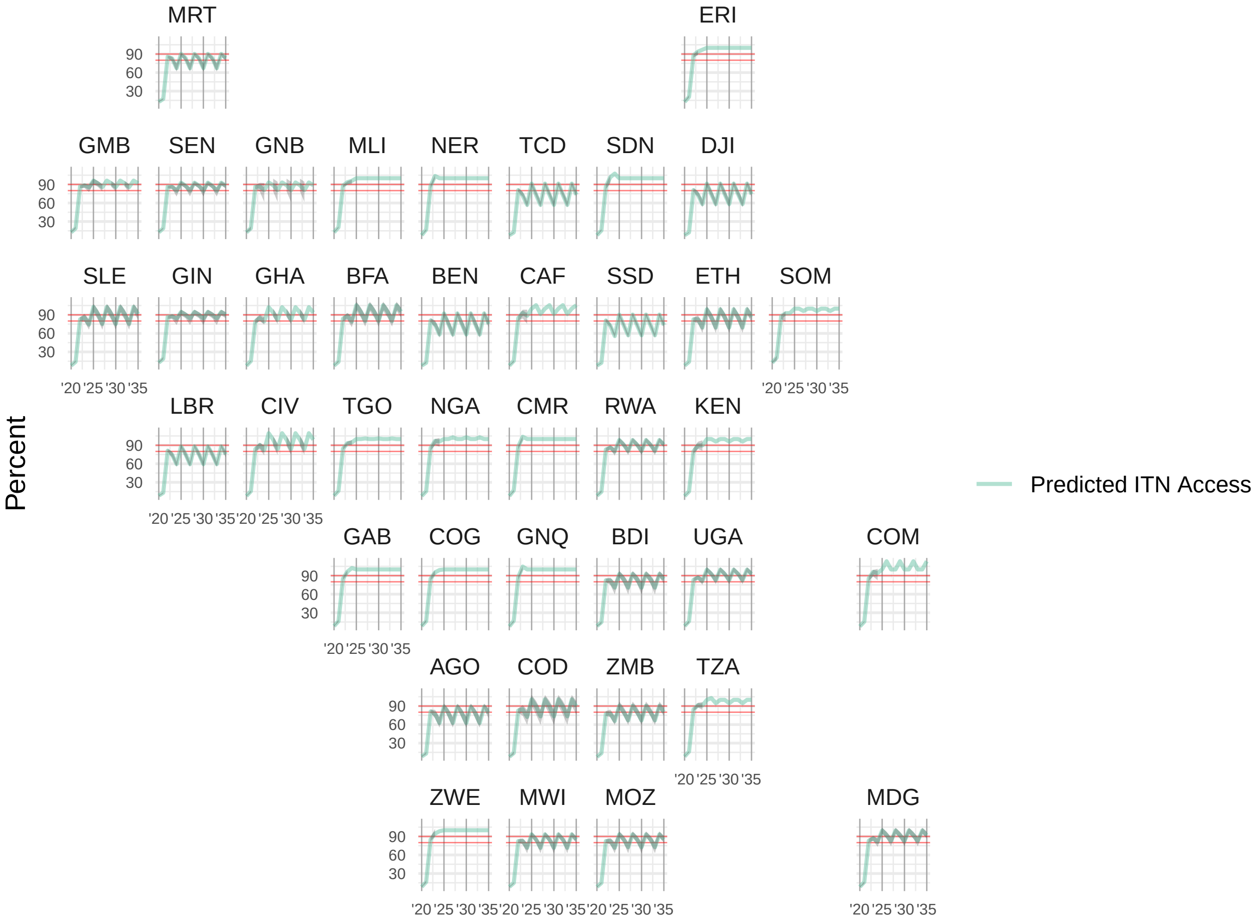

3−year mass campaigns with ANC/EPI at 6%
and between−campaign school/community distribution
at 20 % of the population

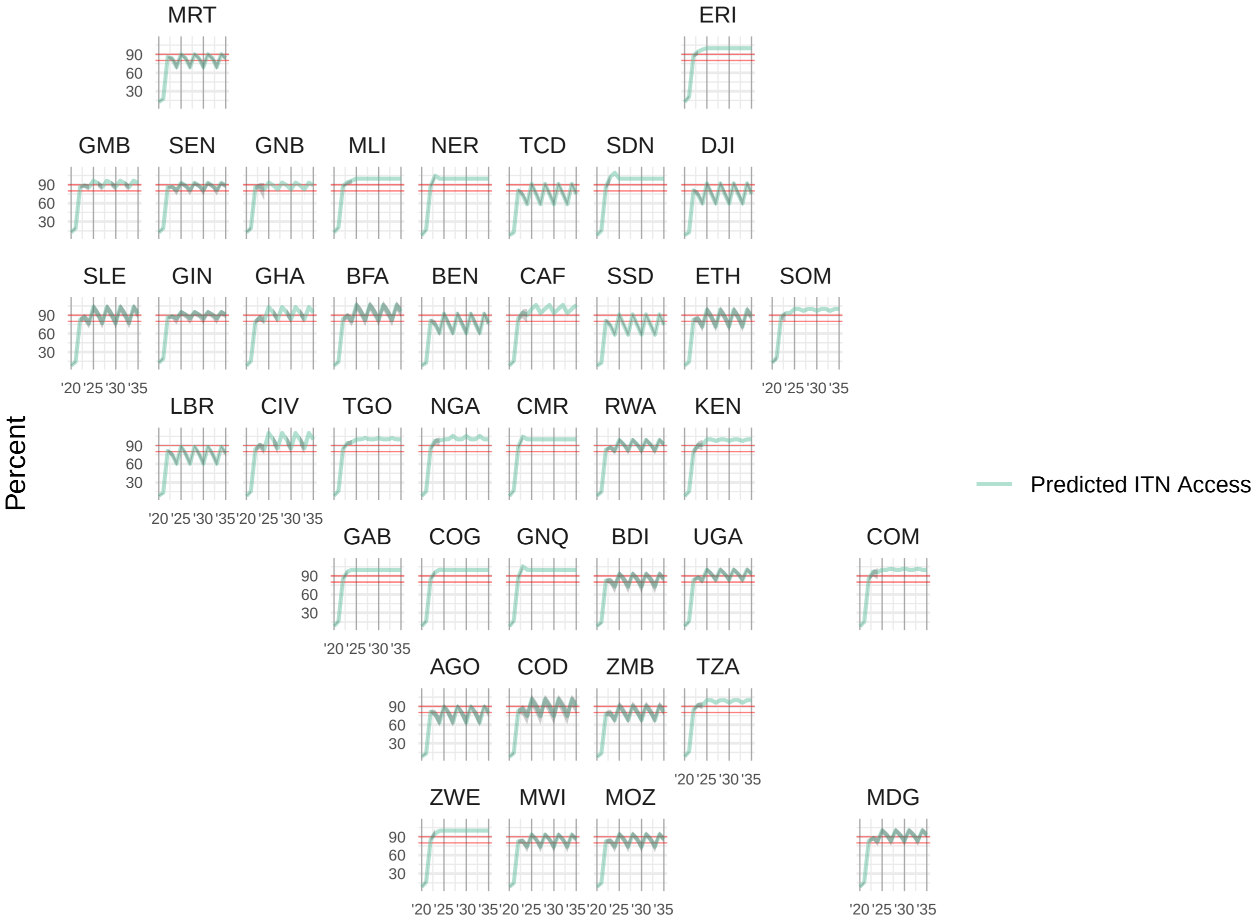

3−year mass campaigns with ANC/EPI at 6%
and between−campaign school/community distribution
at 21 % of the population

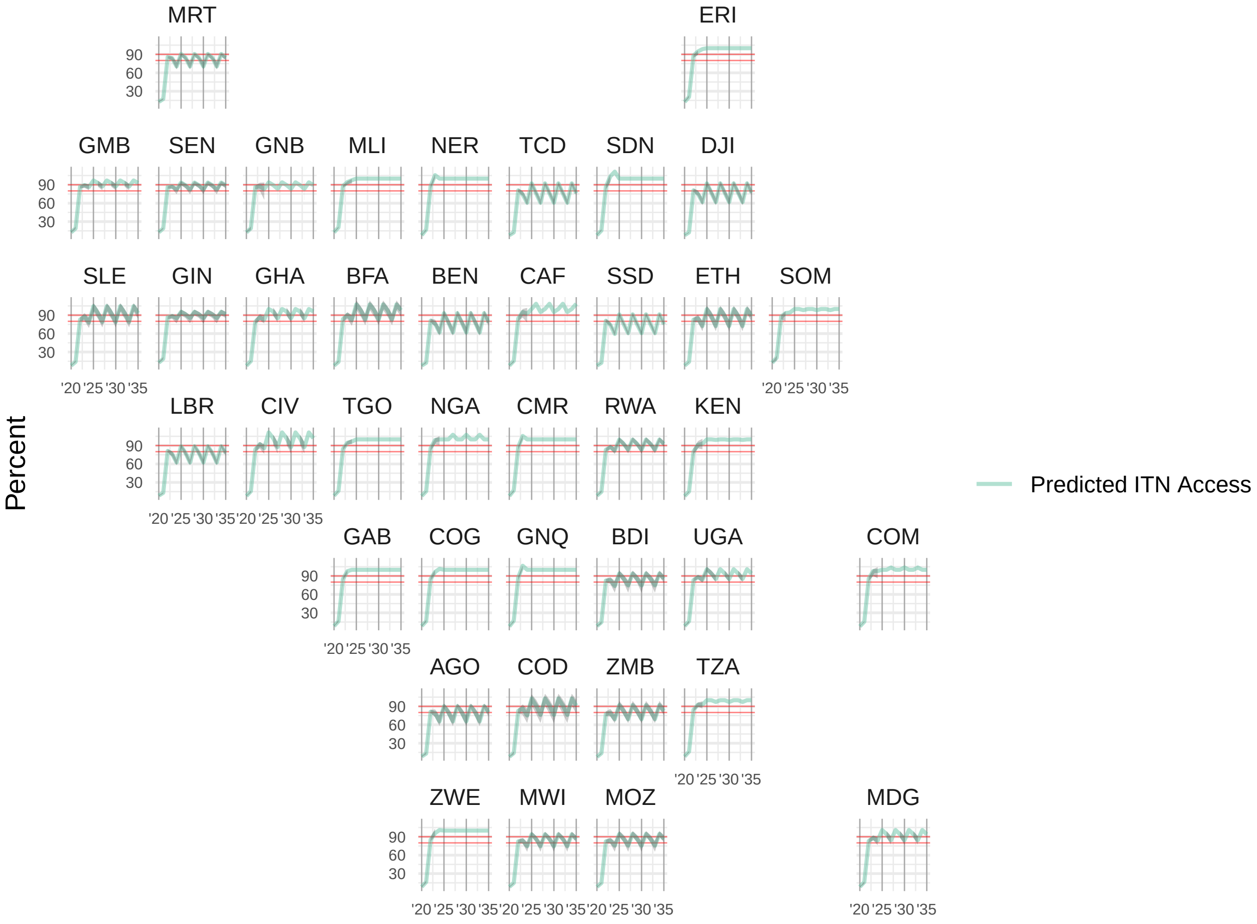

3−year mass campaigns with ANC/EPI at 6%
and between−campaign school/community distribution
at 22 % of the population

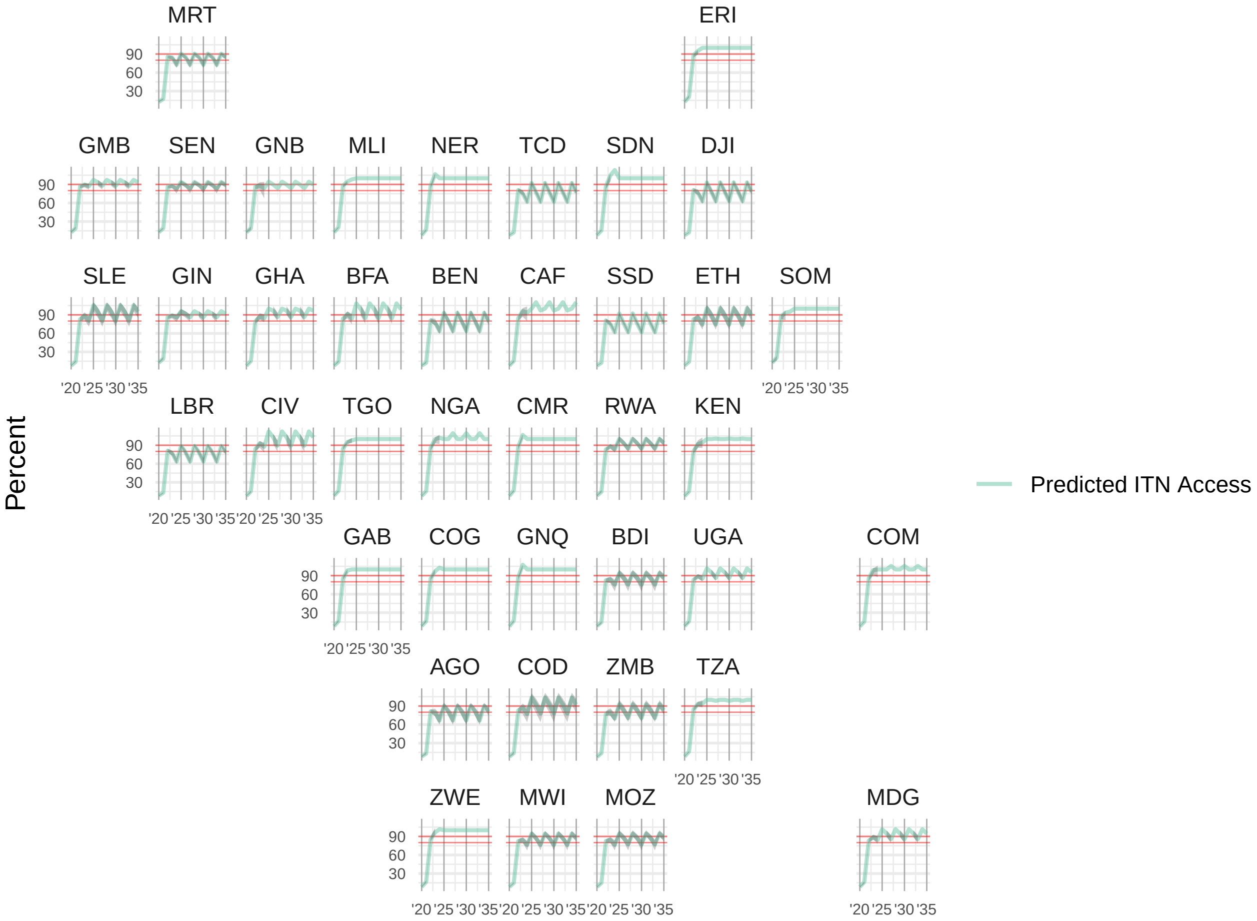

3−year mass campaigns with ANC/EPI at 6%
and between−campaign school/community distribution
at 23 % of the population

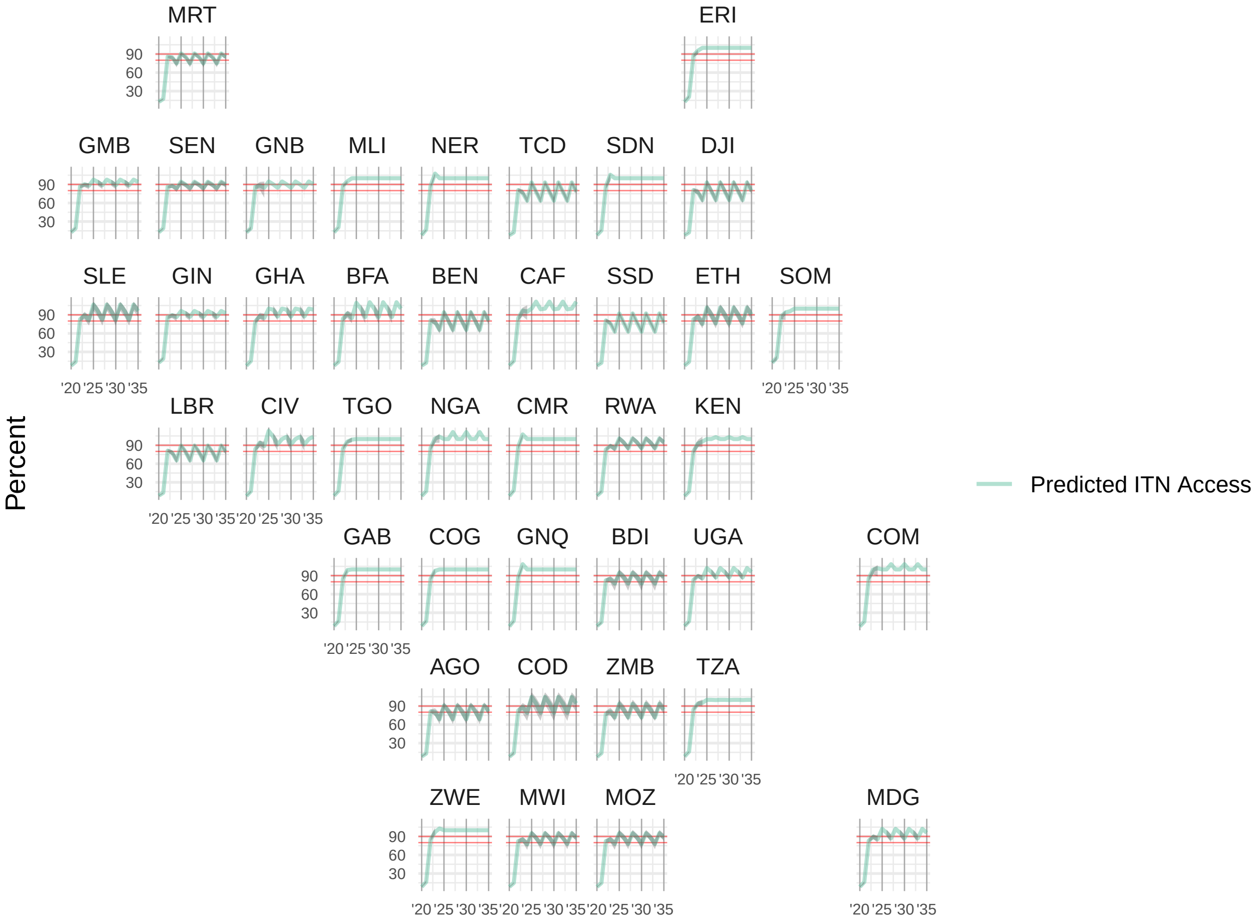

3−year mass campaigns with ANC/EPI at 6%
and between−campaign school/community distribution
at 24 % of the population

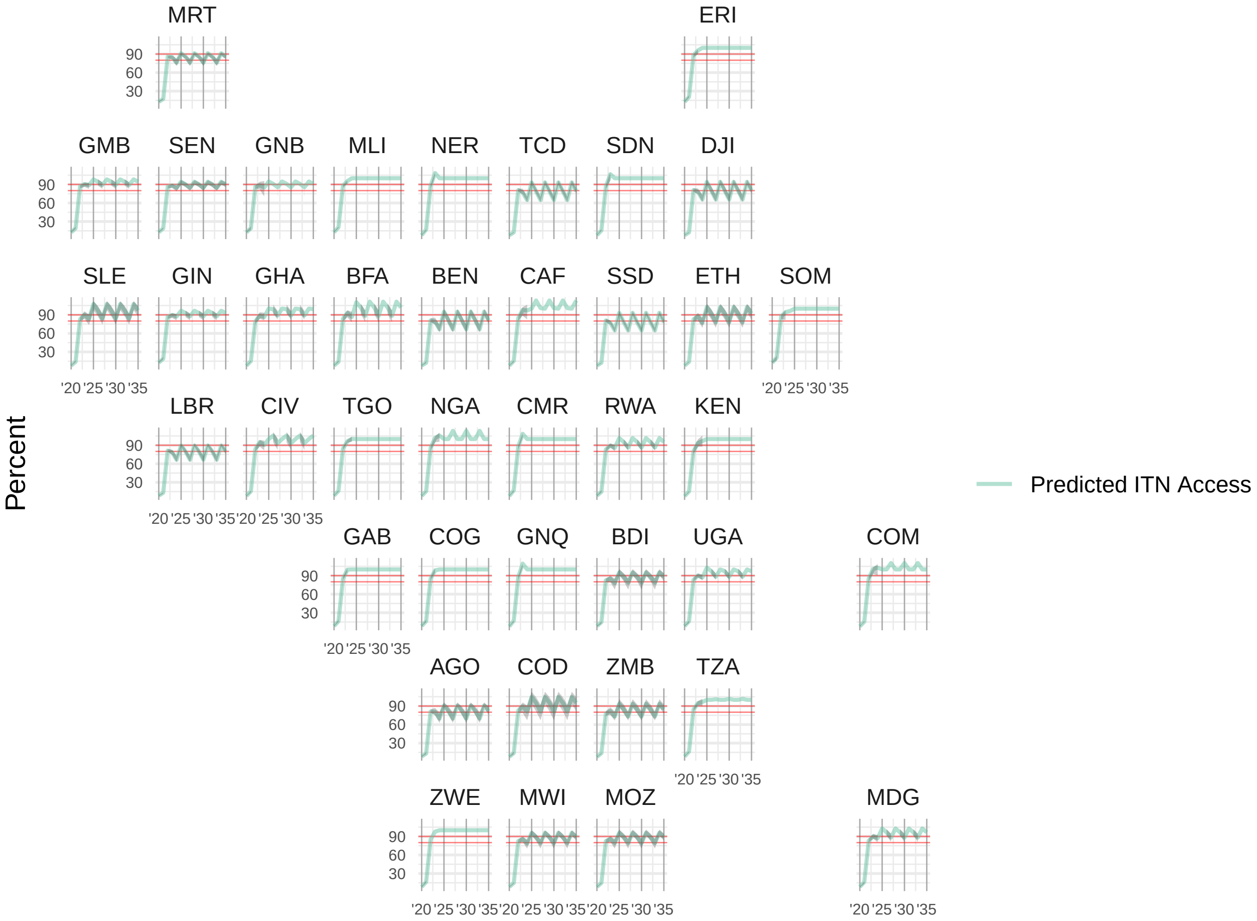

3−year mass campaigns with ANC/EPI at 6%
and between−campaign school/community distribution
at 25 % of the population

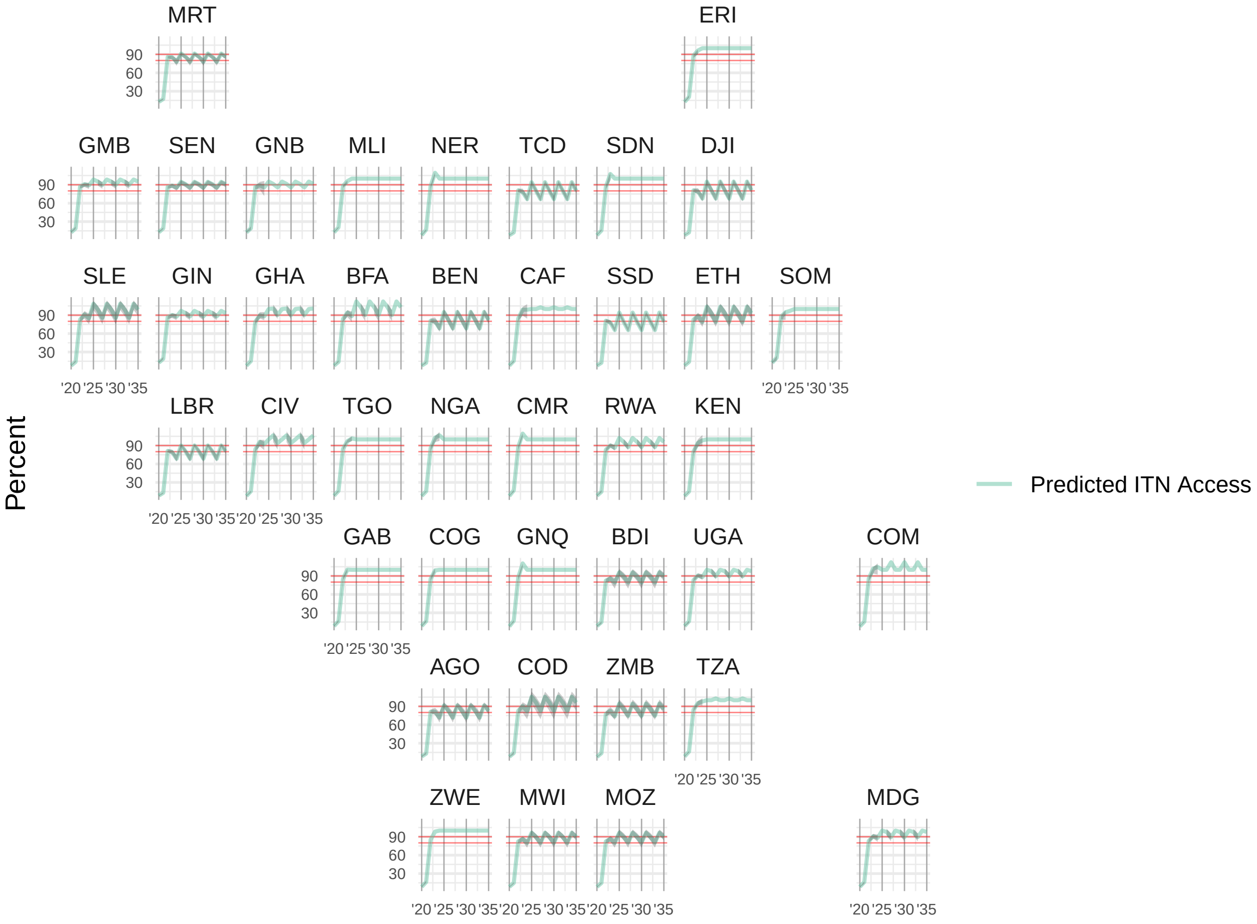

3−year mass campaigns with ANC/EPI at 6%
and between−campaign school/community distribution
at 26 % of the population

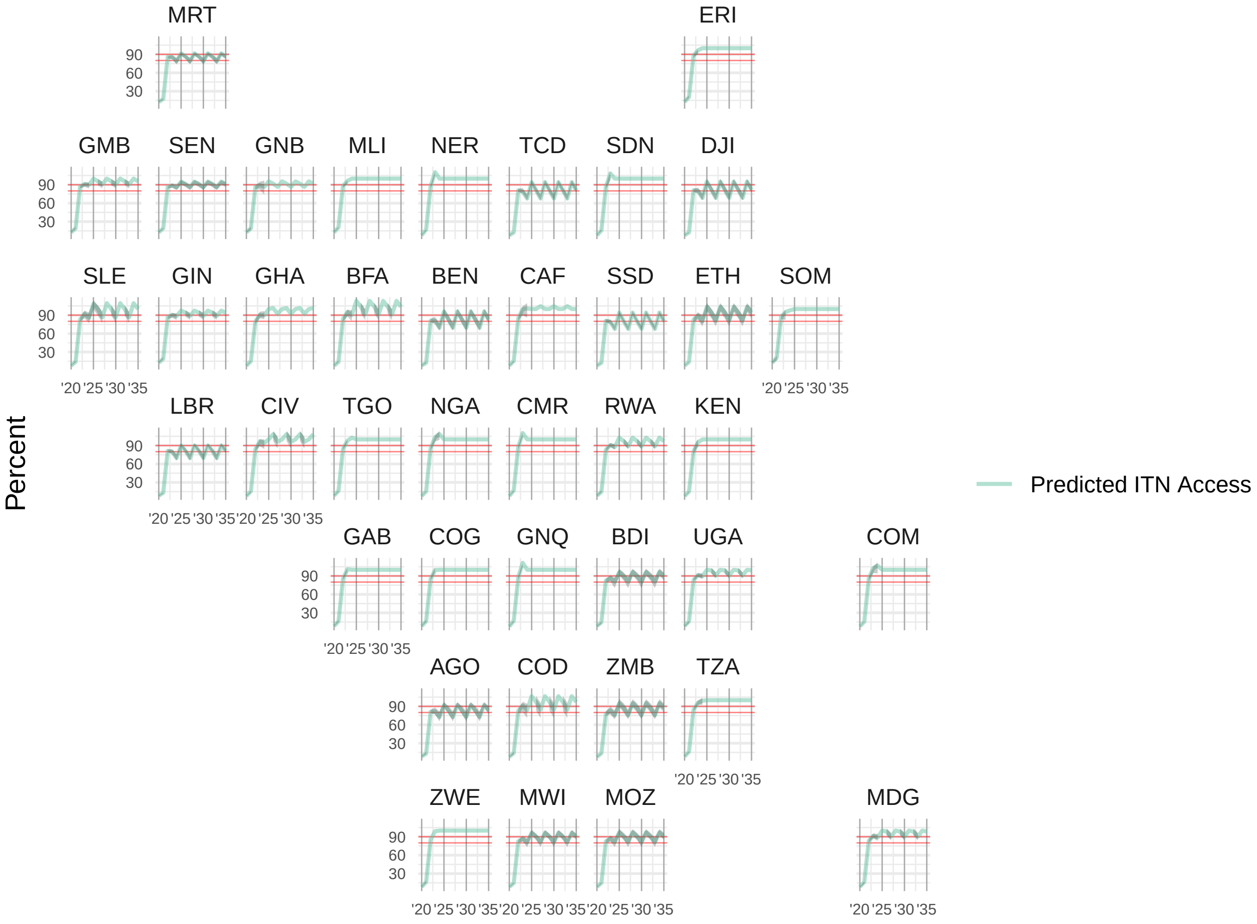

3−year mass campaigns with ANC/EPI at 6%
and between−campaign school/community distribution
at 27 % of the population

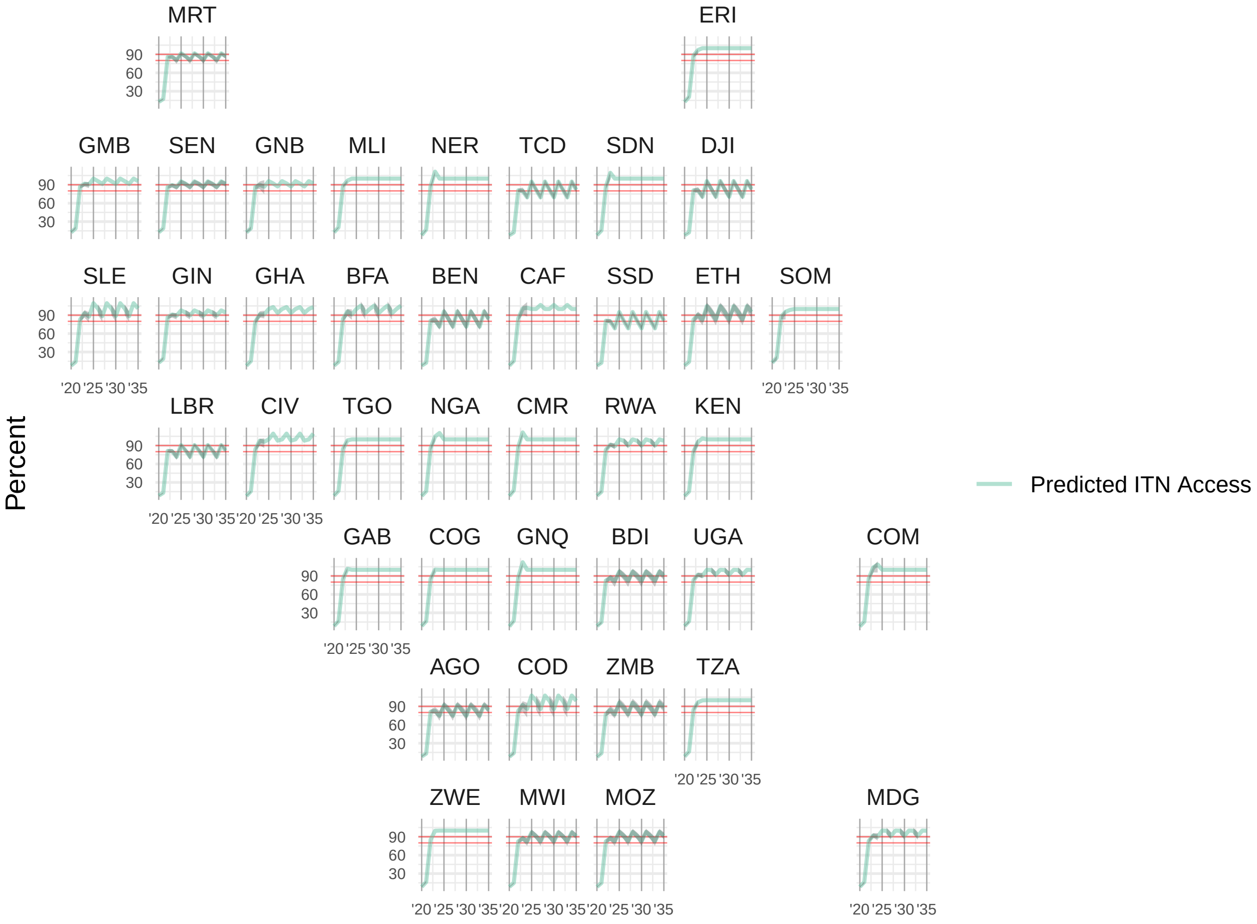

3−year mass campaigns with ANC/EPI at 6%
and between−campaign school/community distribution
at 28 % of the population

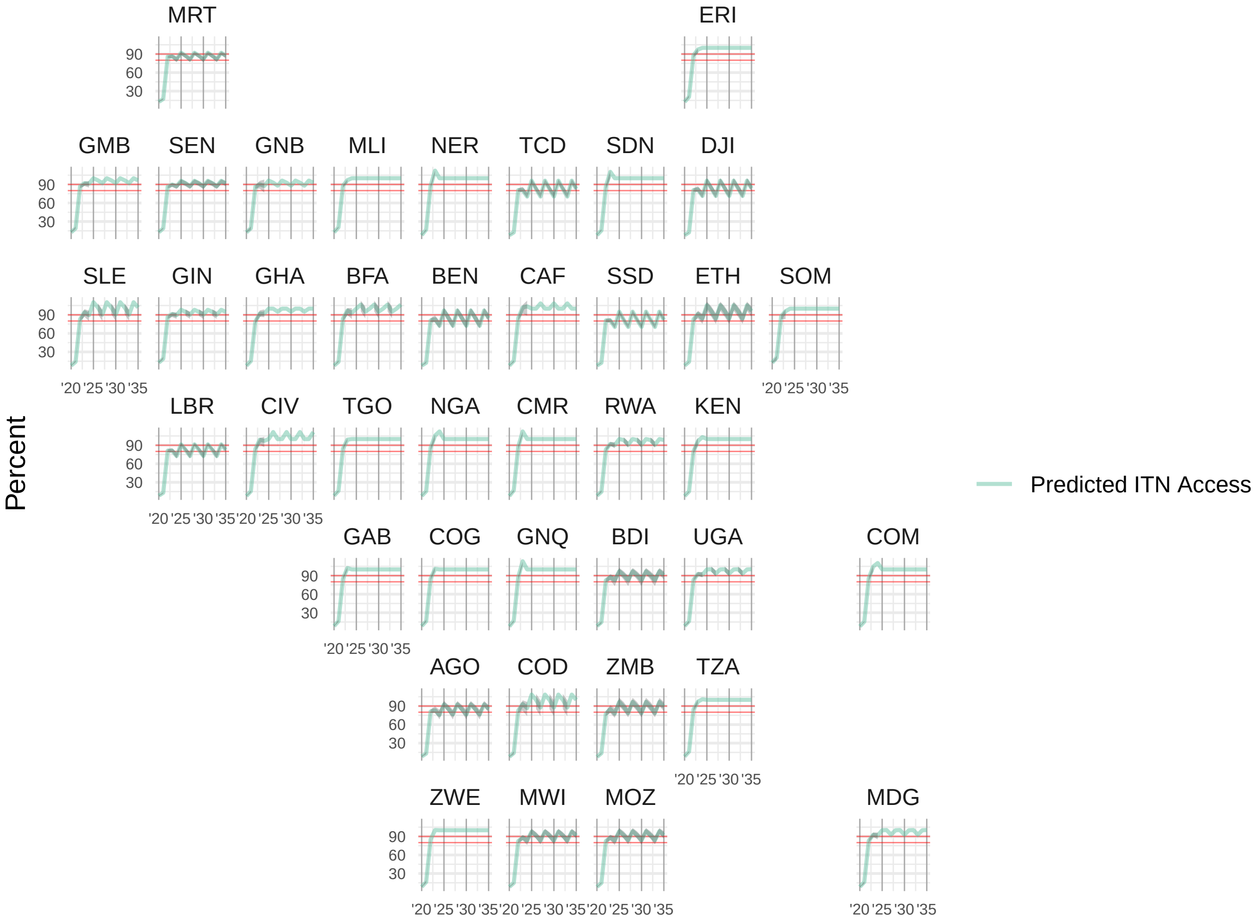

3−year mass campaigns with ANC/EPI at 6%
and between−campaign school/community distribution
at 29 % of the population

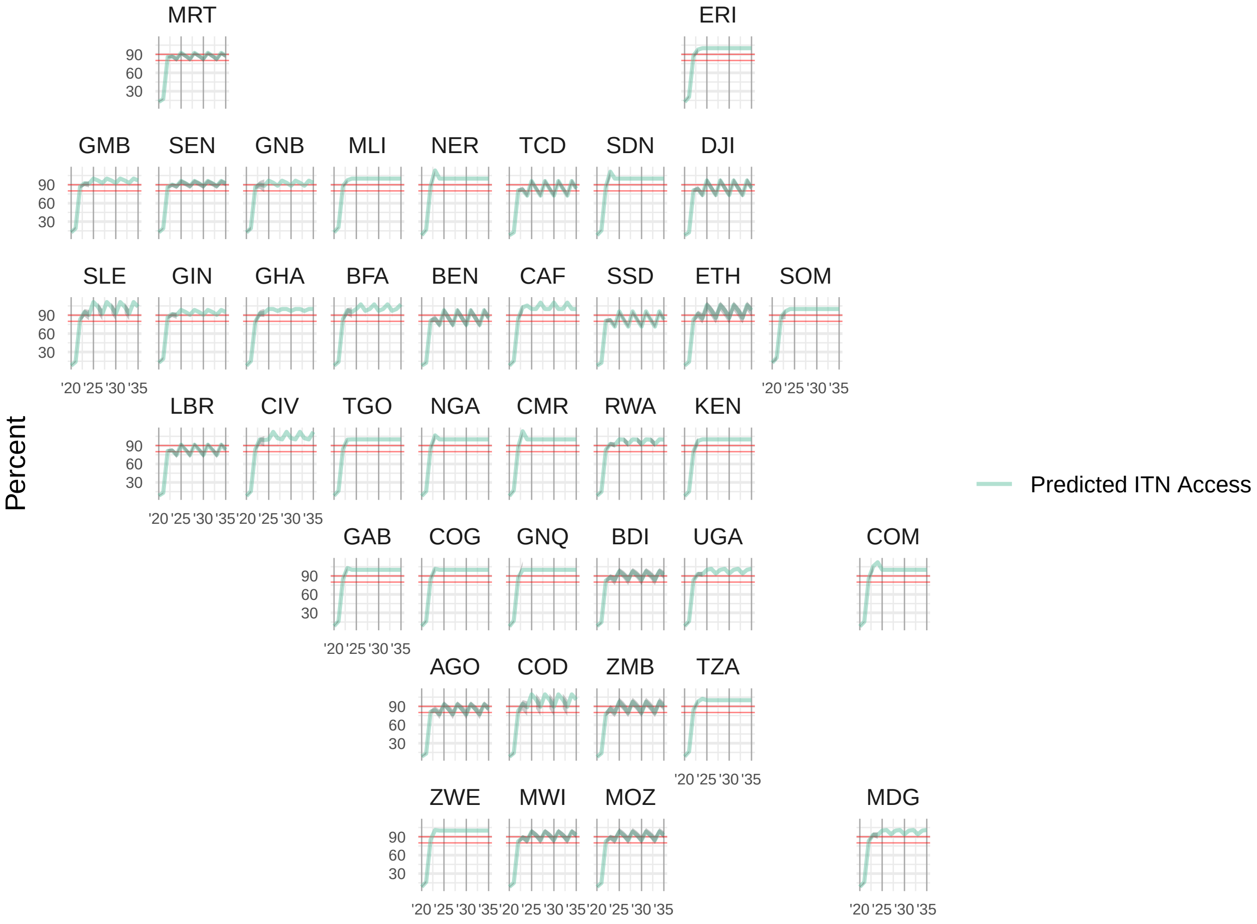

3−year mass campaigns with ANC/EPI at 6%
and between−campaign school/community distribution
at 30 % of the population

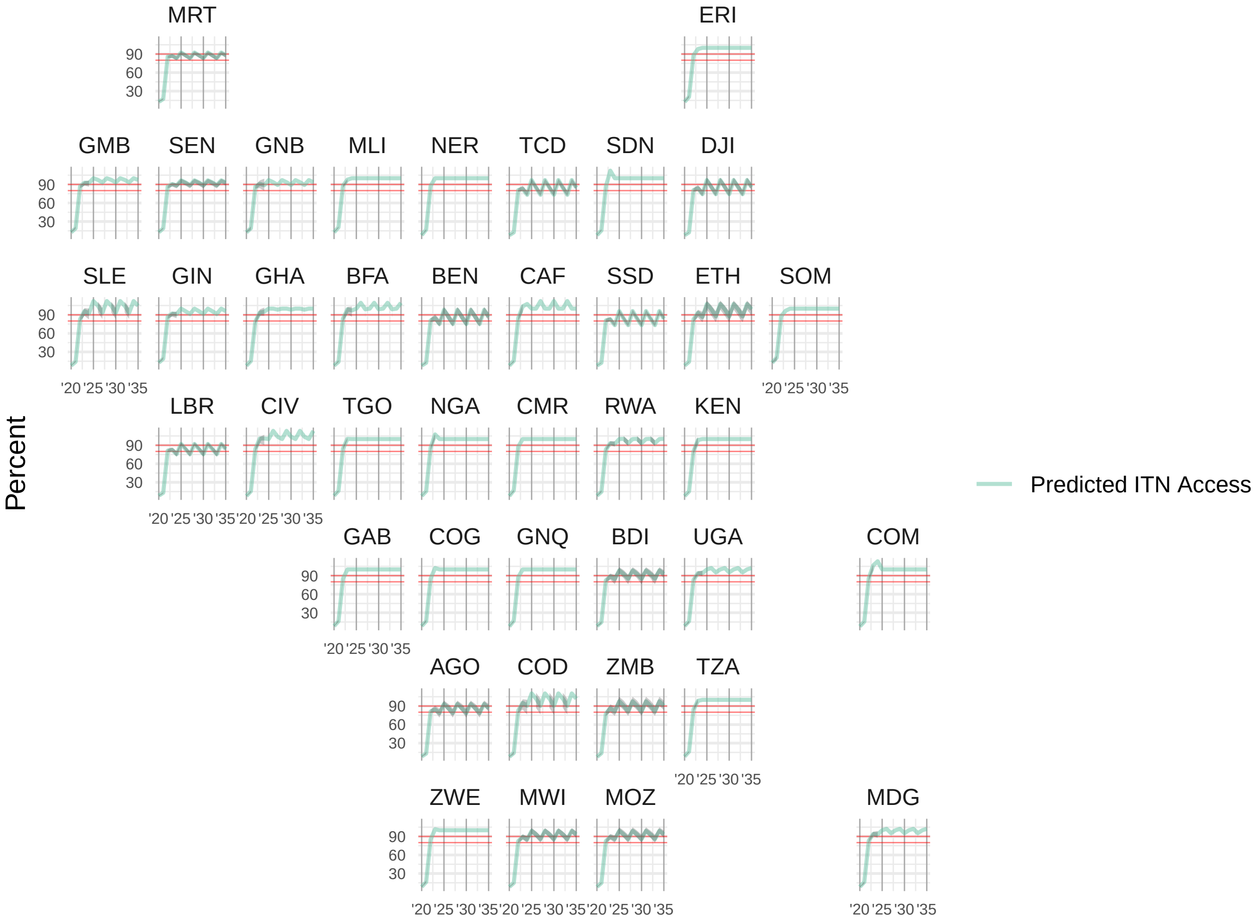

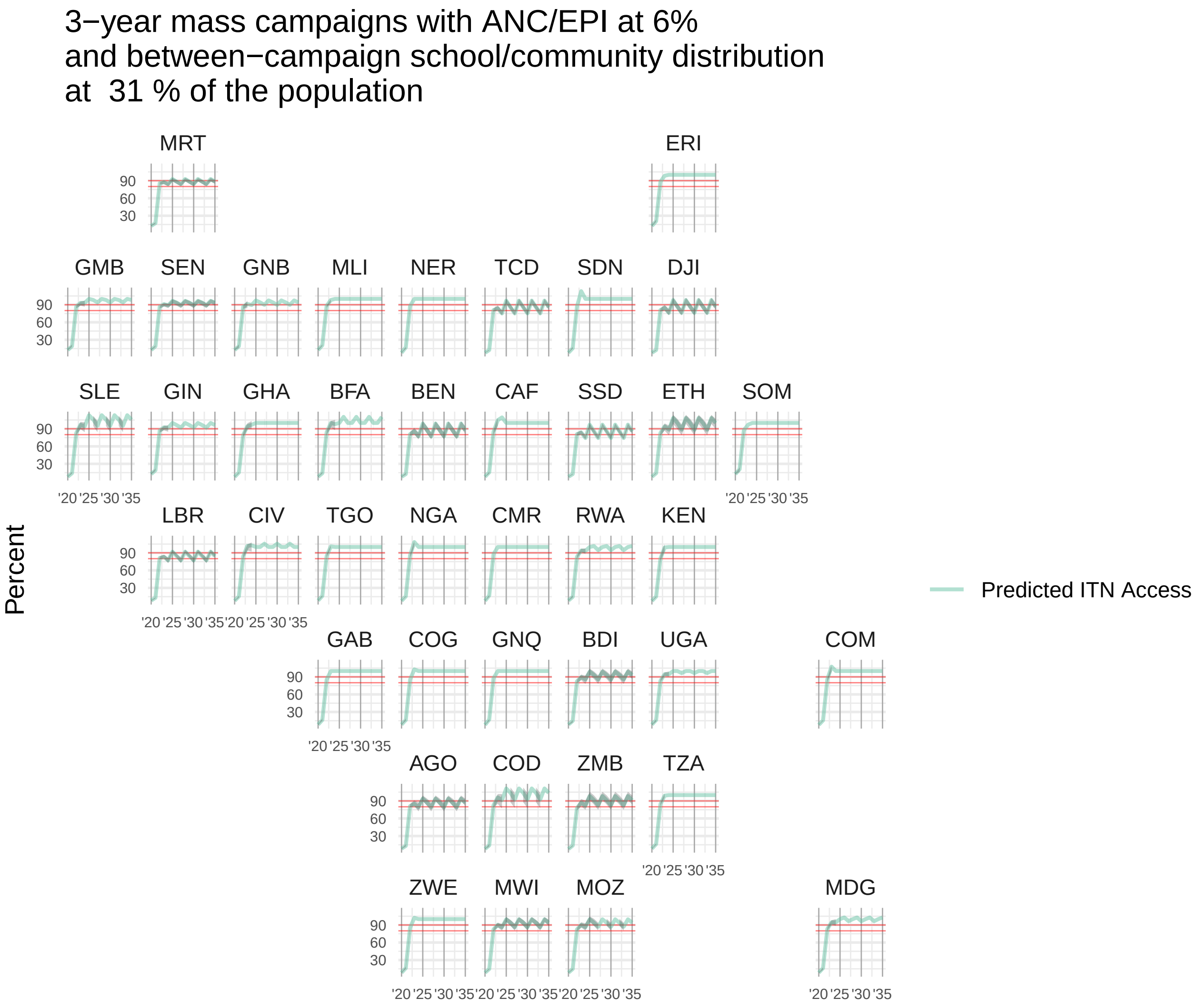
3−year mass campaigns with ANC/EPI at 6%
and between−campaign school/community distribution
at 31 % of the population
MRT
ERI
GMB
SEN
GNB
MLI
NER
TCD
SDN
DJI
SLE
GIN
GHA
BFA
BEN
CAF
SSD
ETH
SOM
LBR
CIV
TGO
NGA
CMR
RWA
KEN
GAB
COG
GNQ
BDI
UGA
COM
AGO
COD
ZMB
TZA
ZWE
MWI
MOZ
MDG
90
60
30
'20 '25 '30 '35
Percent
Predicted ITN Access

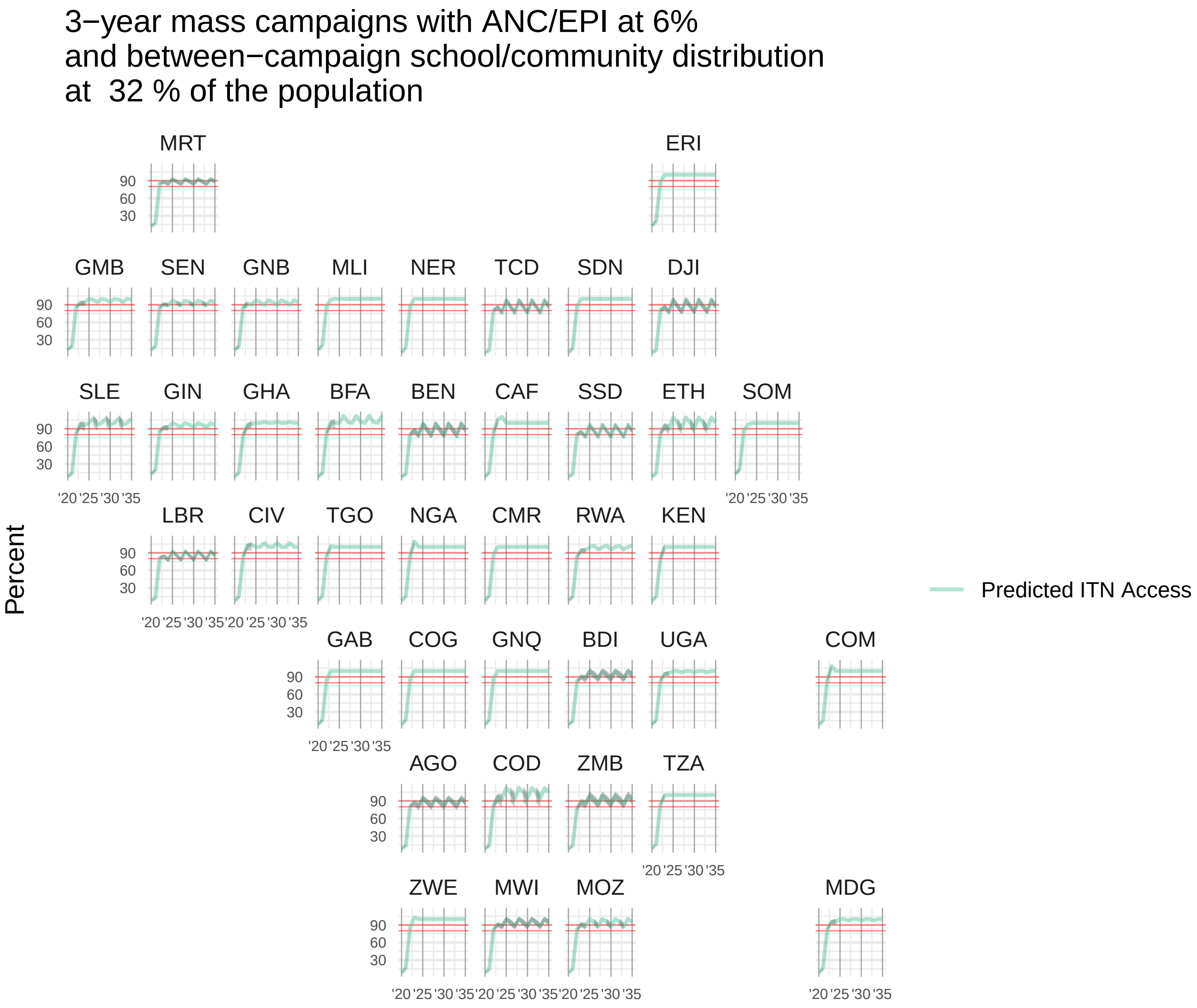

3−year mass campaigns with ANC/EPI at 6%
and between−campaign school/community distribution
at 32 % of the population
MRT
ERI
GMB
SEN
GNB
MLI
NER
TCD
SDN
DJI
SLE
GIN
GHA
BFA
BEN
CAF
SSD
ETH
SOM
LBR
CIV
TGO
NGA
CMR
RWA
KEN
GAB
COG
GNQ
BDI
UGA
COM
AGO
COD
ZMB
TZA
ZWE
MWI
MOZ
MDG
90
60
30
'20 '25 '30 '35
Percent
Predicted ITN Access

3−year mass campaigns with ANC/EPI at 6%
and between−campaign school/community distribution
at 33 % of the population

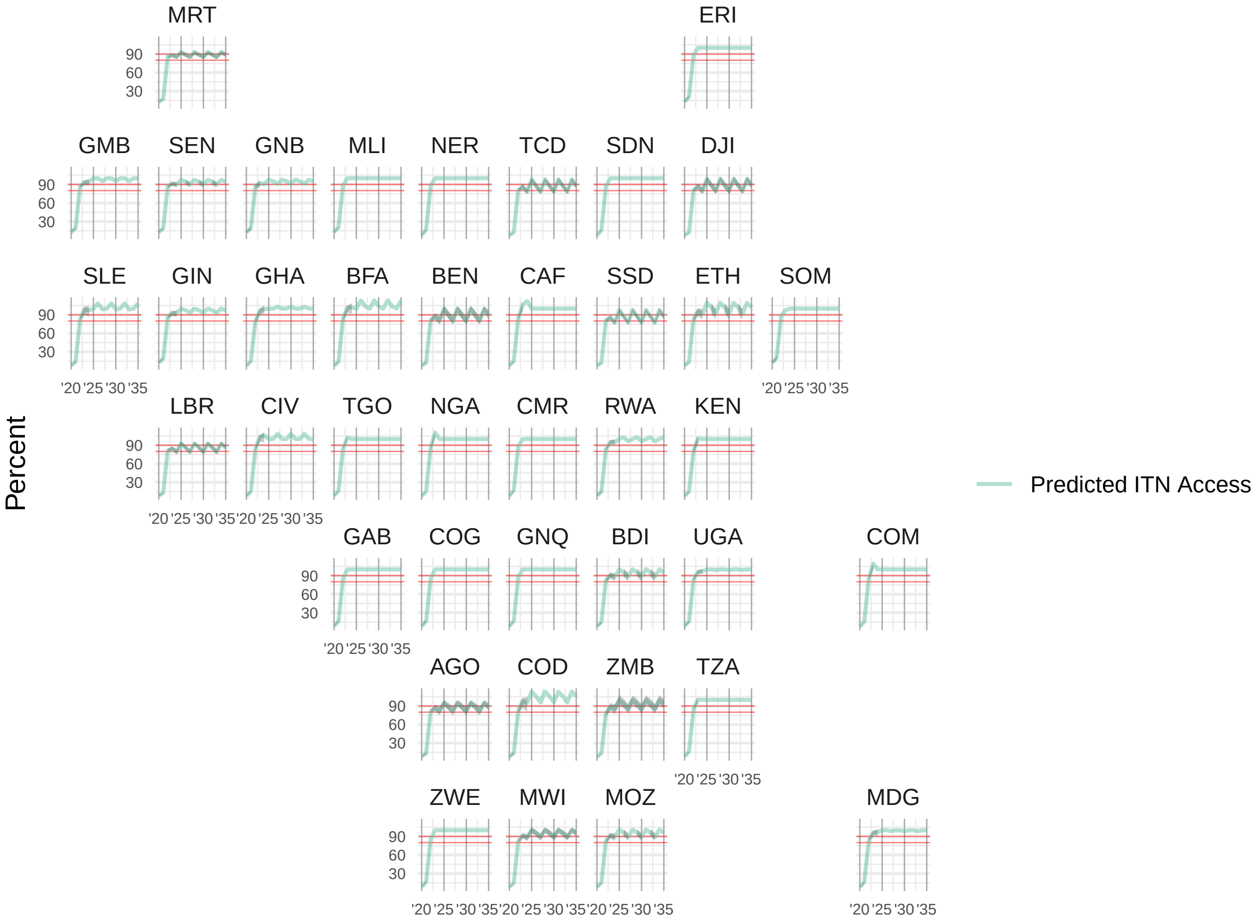

3−year mass campaigns with ANC/EPI at 6%
and between−campaign school/community distribution
at 34 % of the population

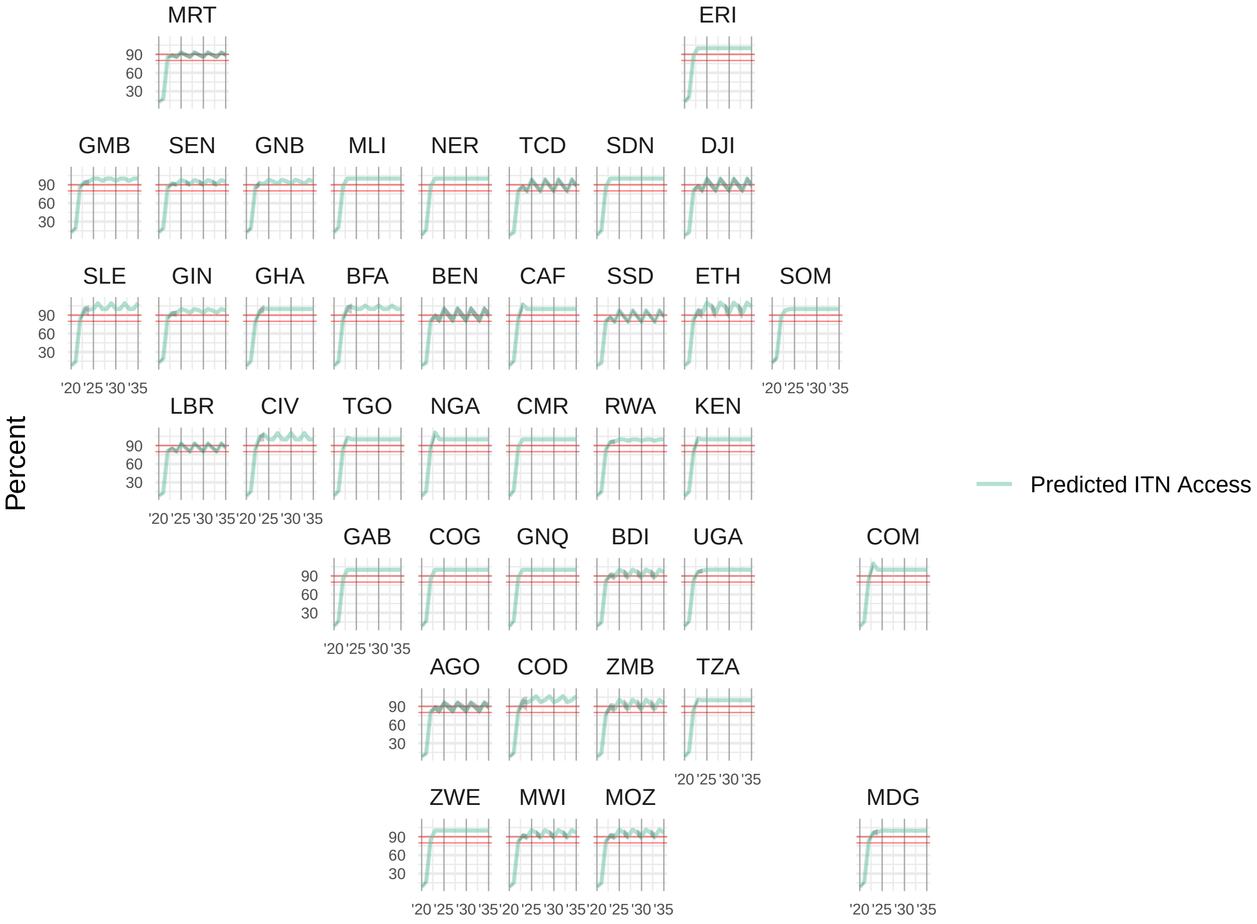

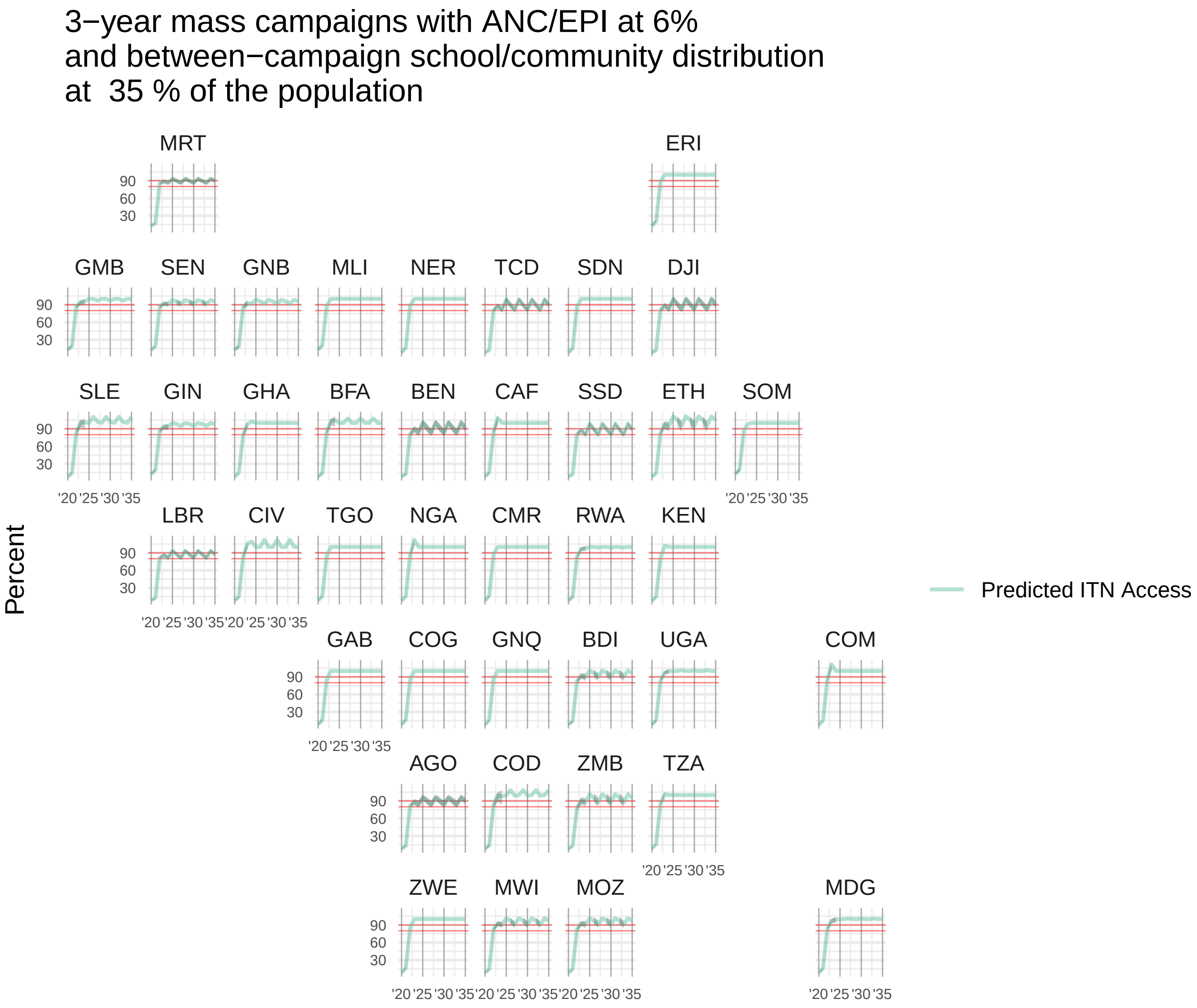
3−year mass campaigns with ANC/EPI at 6%
and between−campaign school/community distribution
at 35 % of the population
Percent
MRT
ERI
GMB
SEN
GNB
MLI
NER
TCD
SDN
DJI
SLE
GIN
GHA
BFA
BEN
CAF
SSD
ETH
SOM
LBR
CIV
TGO
NGA
CMR
RWA
KEN
GAB
COG
GNQ
BDI
UGA
COM
AGO
COD
ZMB
TZA
ZWE
MWI
MOZ
MDG
90
60
30
'20 '25 '30 '35
Predicted ITN Access

3−year mass campaigns with ANC/EPI at 6%
and between−campaign school/community distribution
at 36 % of the population

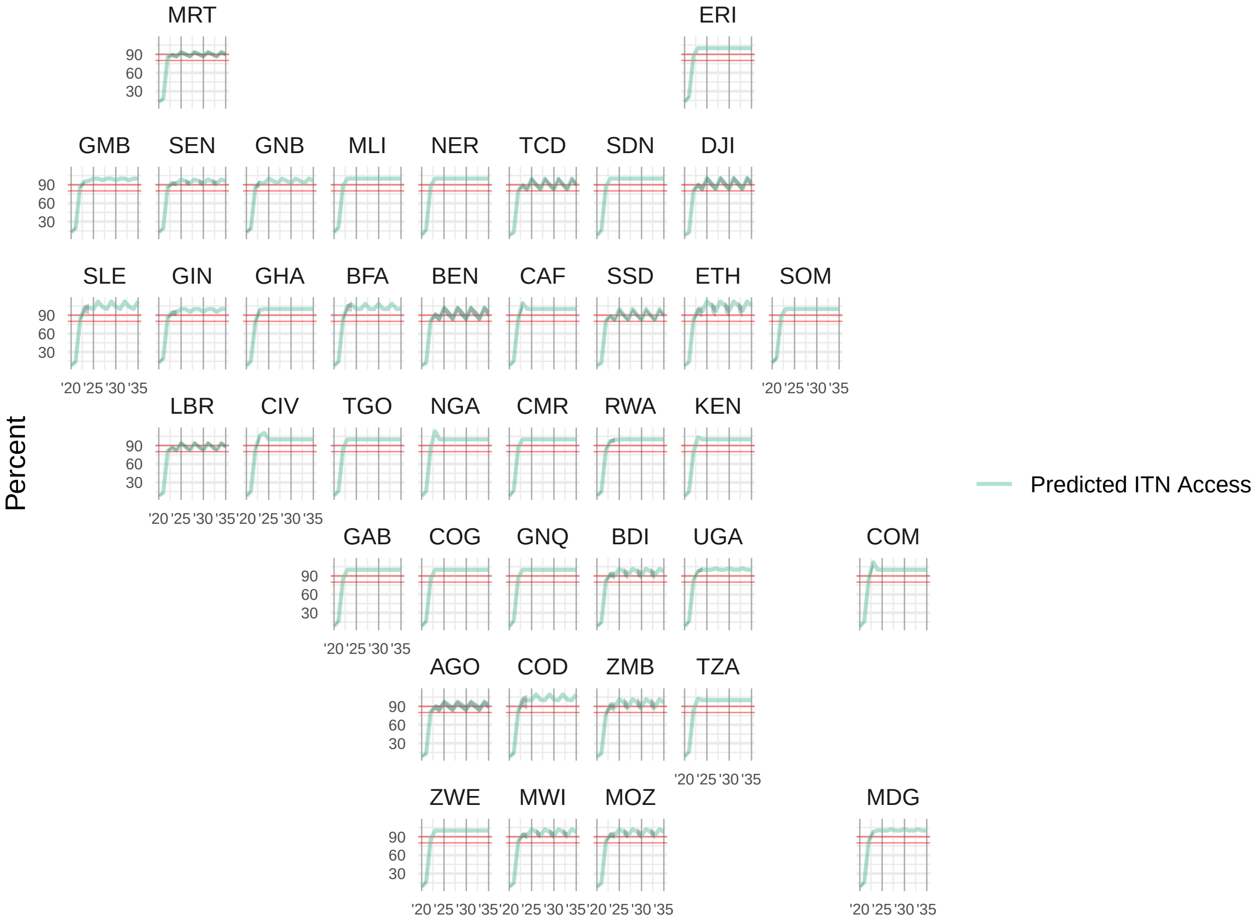

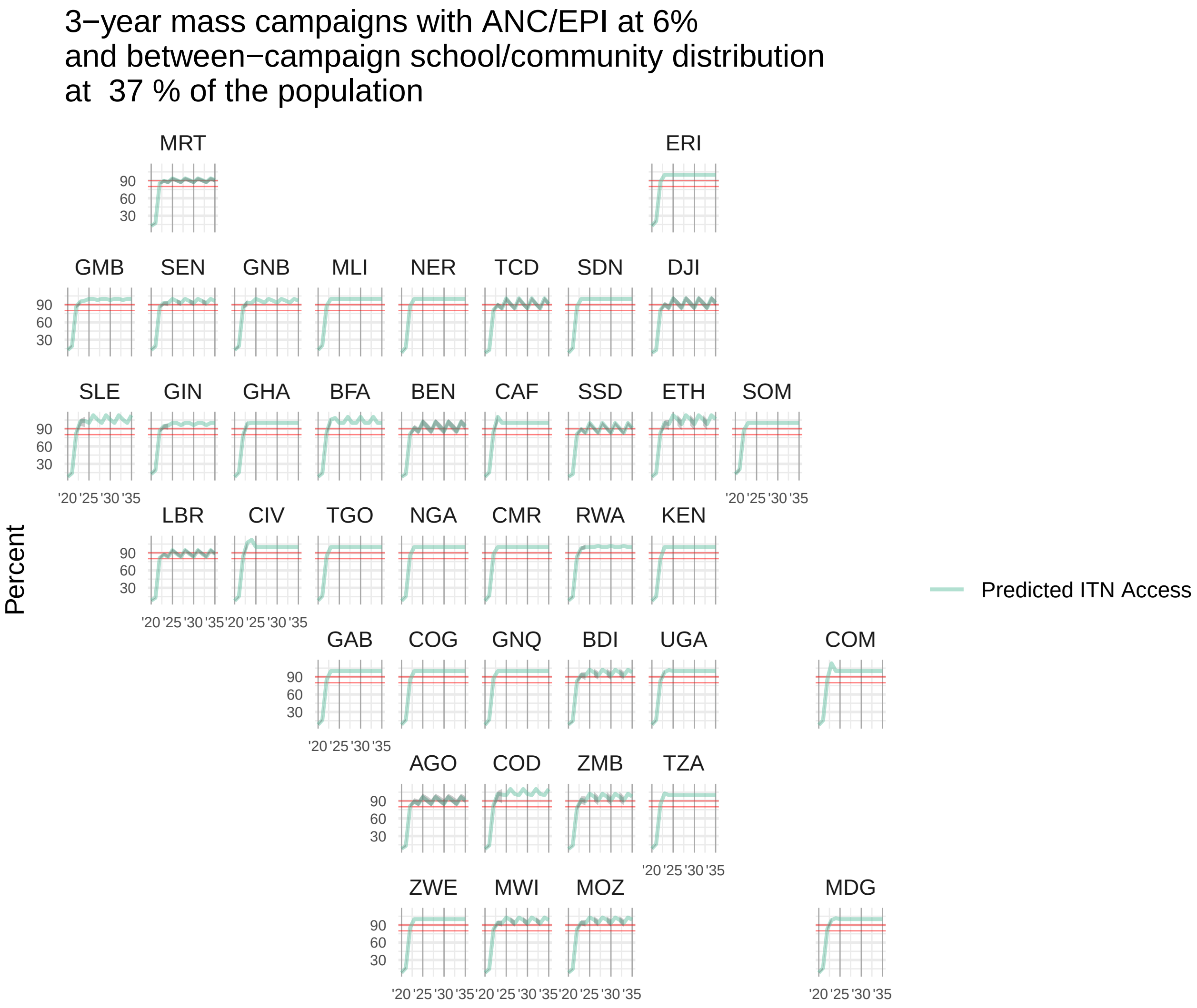
3−year mass campaigns with ANC/EPI at 6%
and between−campaign school/community distribution
at 37 % of the population
MRT
ERI
GMB
SEN
GNB
MLI
NER
TCD
SDN
DJI
SLE
GIN
GHA
BFA
BEN
CAF
SSD
ETH
SOM
LBR
CIV
TGO
NGA
CMR
RWA
KEN
GAB
COG
GNQ
BDI
UGA
COM
AGO
COD
ZMB
TZA
ZWE
MWI
MOZ
MDG
90
60
30
'20 '25 '30 '35
Percent
Predicted ITN Access

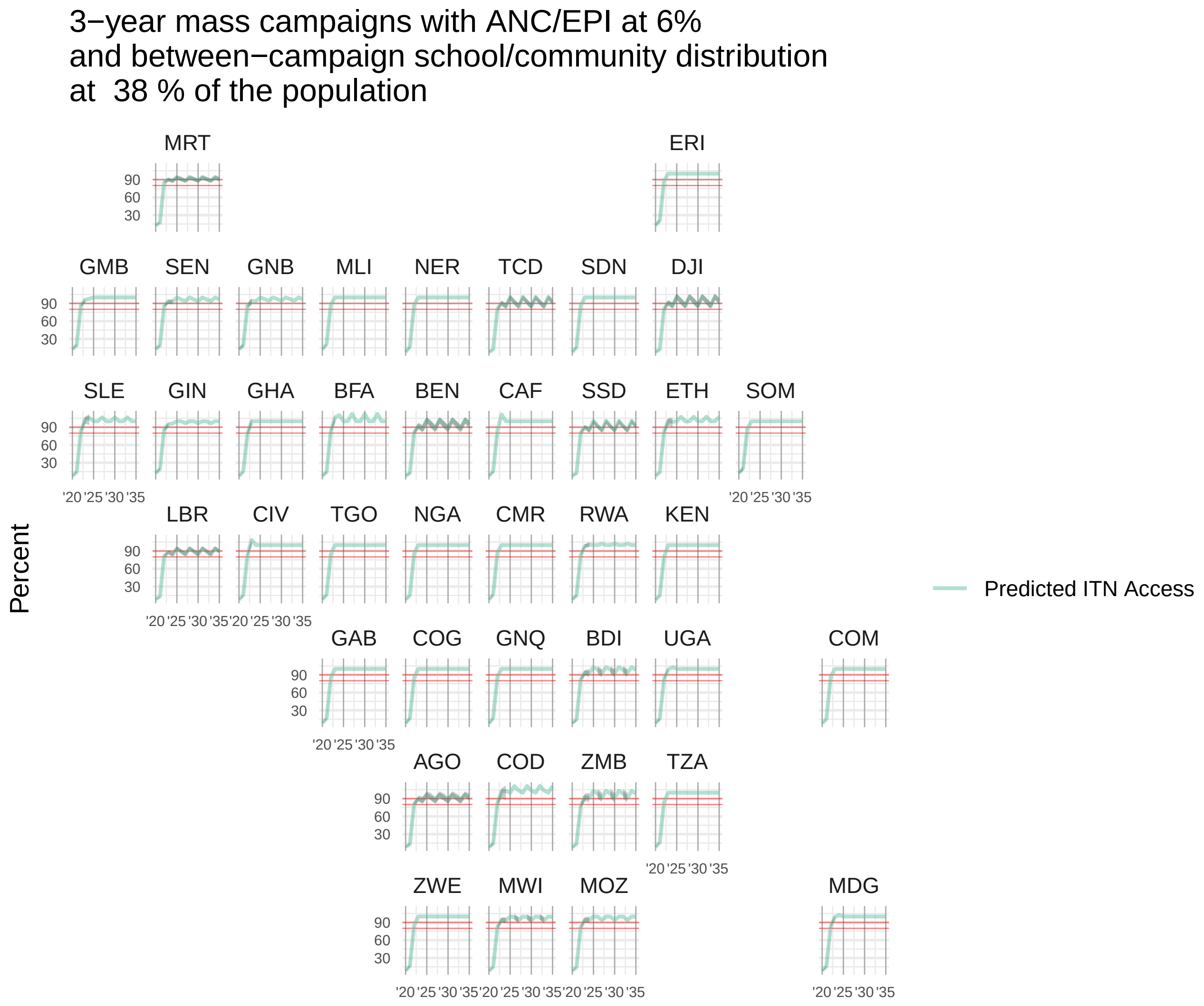
3−year mass campaigns with ANC/EPI at 6%
and between−campaign school/community distribution
at 38 % of the population
MRT
ERI
GMB
SEN
GNB
MLI
NER
TCD
SDN
DJI
SLE
GIN
GHA
BFA
BEN
CAF
SSD
ETH
SOM
LBR
CIV
TGO
NGA
CMR
RWA
KEN
GAB
COG
GNQ
BDI
UGA
COM
AGO
COD
ZMB
TZA
ZWE
MWI
MOZ
MDG
Percent
90
60
30
'20 '25 '30 '35
Predicted ITN Access

3−year mass campaigns with ANC/EPI at 6%
and between−campaign school/community distribution
at 39 % of the population

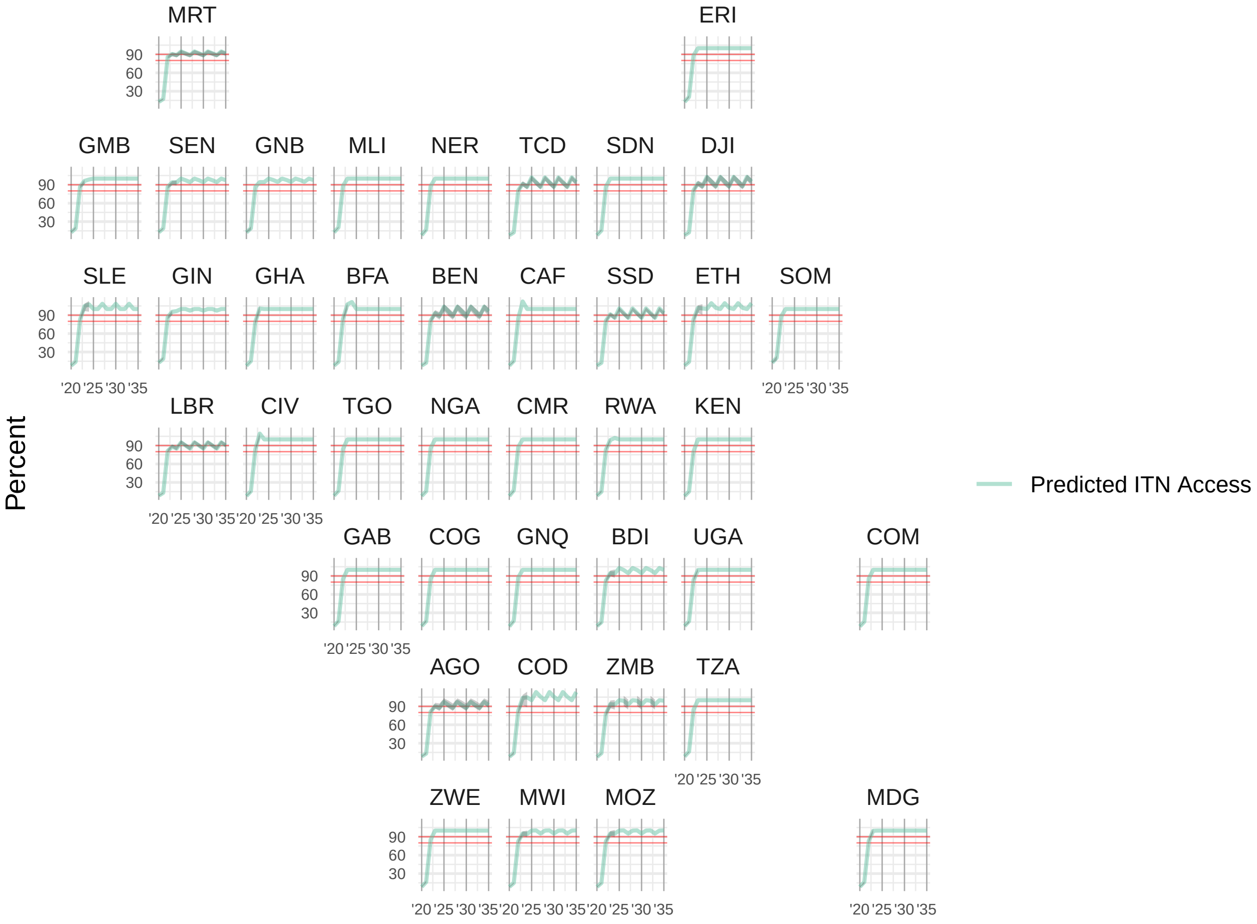

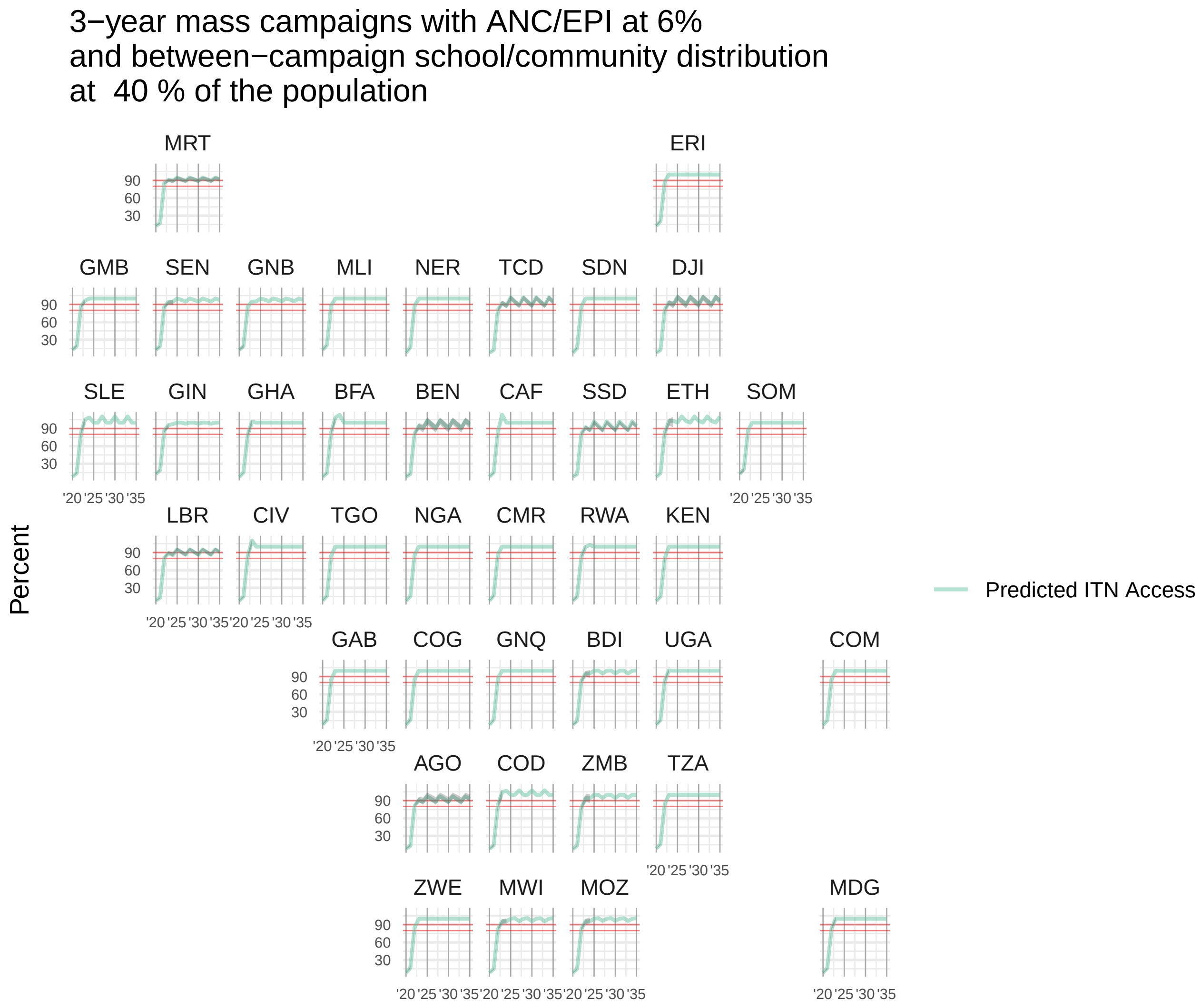
3−year mass campaigns with ANC/EPI at 6%
and between−campaign school/community distribution
at 40 % of the population
MRT
ERI
GMB
SEN
GNB
MLI
NER
TCD
SDN
DJI
SLE
GIN
GHA
BFA
BEN
CAF
SSD
ETH
SOM
LBR
CIV
TGO
NGA
CMR
RWA
KEN
GAB
COG
GNQ
BDI
UGA
COM
AGO
COD
ZMB
TZA
ZWE
MWI
MOZ
MDG
90
60
30
'20 '25 '30 '35
Percent
Predicted ITN Access

4 Scenario 4 - Three-year mass campaigns with ANC/EPI distribution at 6%, varying campaign quantifier

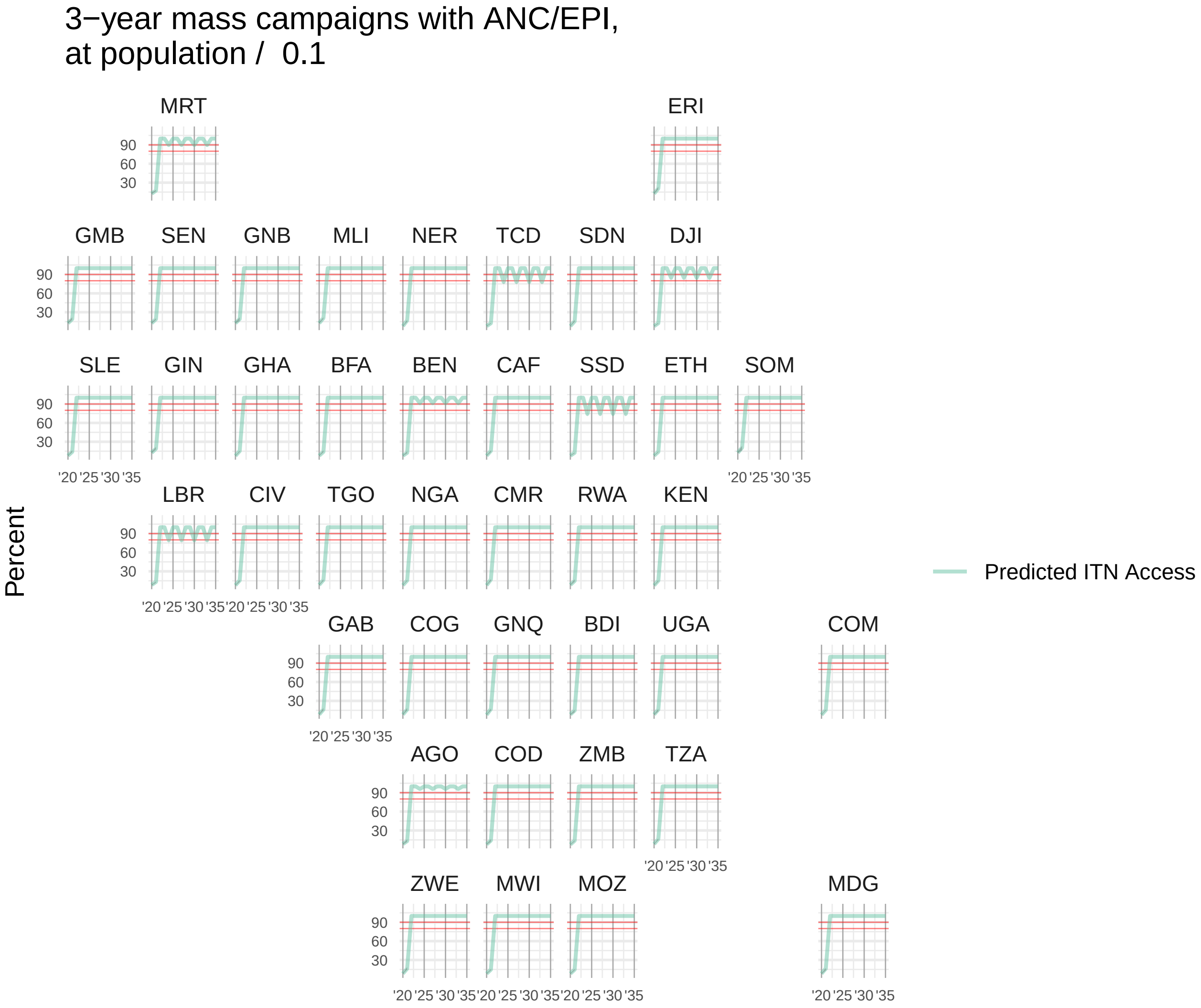

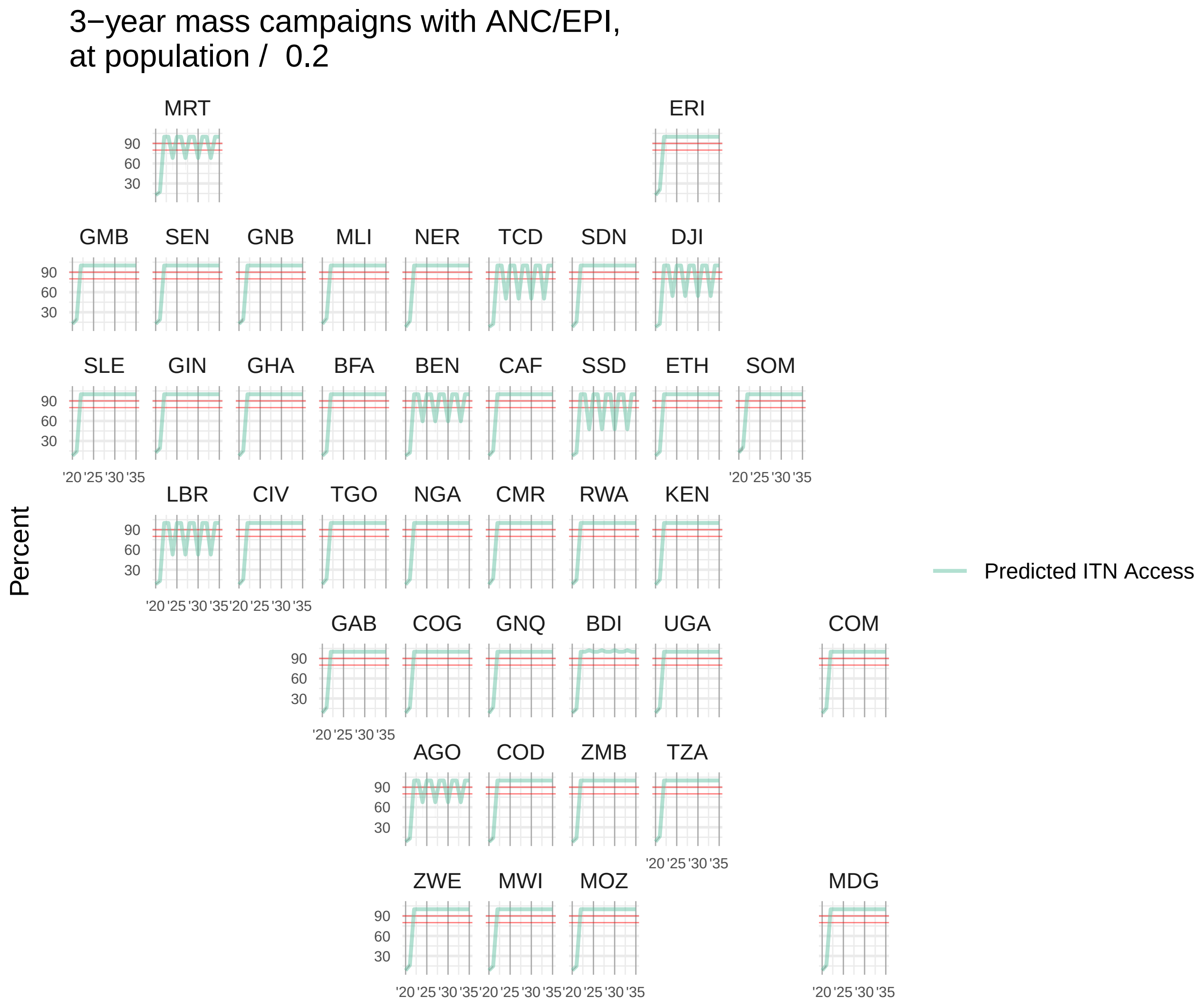
3−year mass campaigns with ANC/EPI,
at population / 0.2
MRT
ERI
GMB
SEN
GNB
MLI
NER
TCD
SDN
DJI
SLE
GIN
GHA
BFA
BEN
CAF
SSD
ETH
SOM
LBR
CIV
TGO
NGA
CMR
RWA
KEN
GAB
COG
GNQ
BDI
UGA
COM
AGO
COD
ZMB
TZA
ZWE
MWI
MOZ
MDG
Percent
90
60
30
'20 '25 '30 '35
Predicted ITN Access

3−year mass campaigns with ANC/EPI,
at population / 0.3

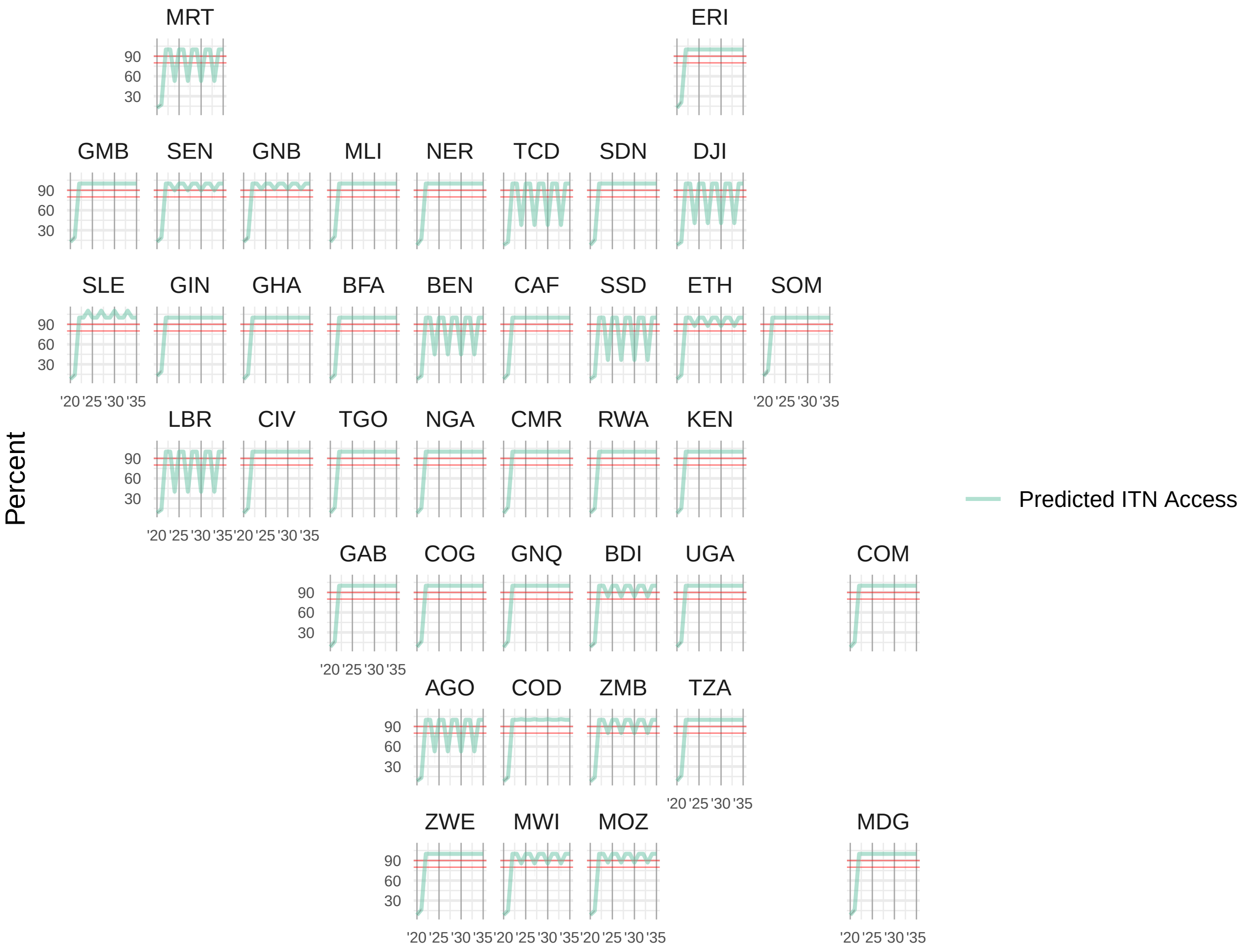

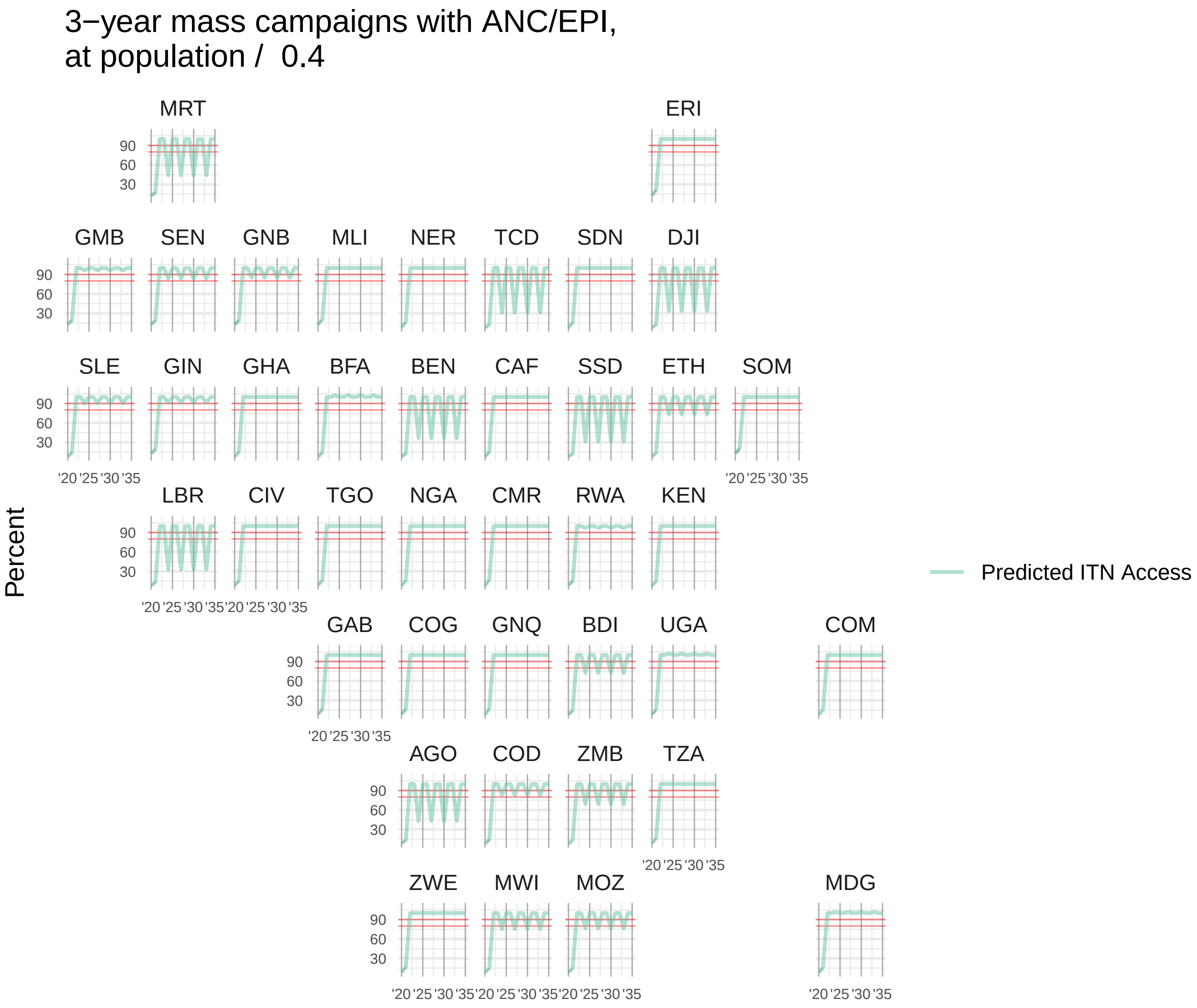
3−year mass campaigns with ANC/EPI,
at population / 0.4
MRT
ERI
GMB
SEN
GNB
MLI
NER
TCD
SDN
DJI
SLE
GIN
GHA
BFA
BEN
CAF
SSD
ETH
SOM
LBR
CIV
TGO
NGA
CMR
RWA
KEN
GAB
COG
GNQ
BDI
UGA
COM
AGO
COD
ZMB
TZA
ZWE
MWI
MOZ
MDG
90
60
30
'20 '25 '30 '35
Percent
Predicted ITN Access

3−year mass campaigns with ANC/EPI,
at population / 0.5

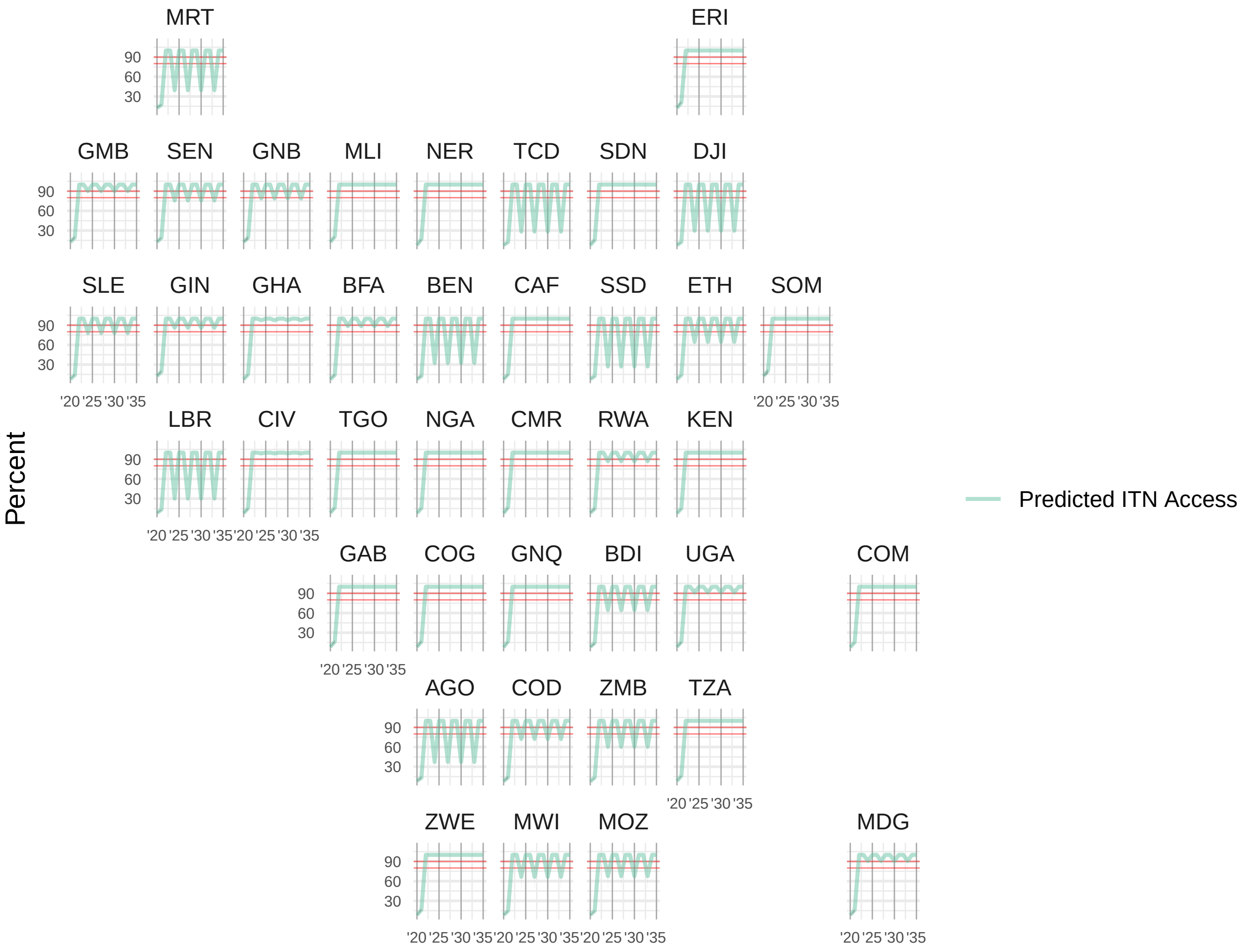

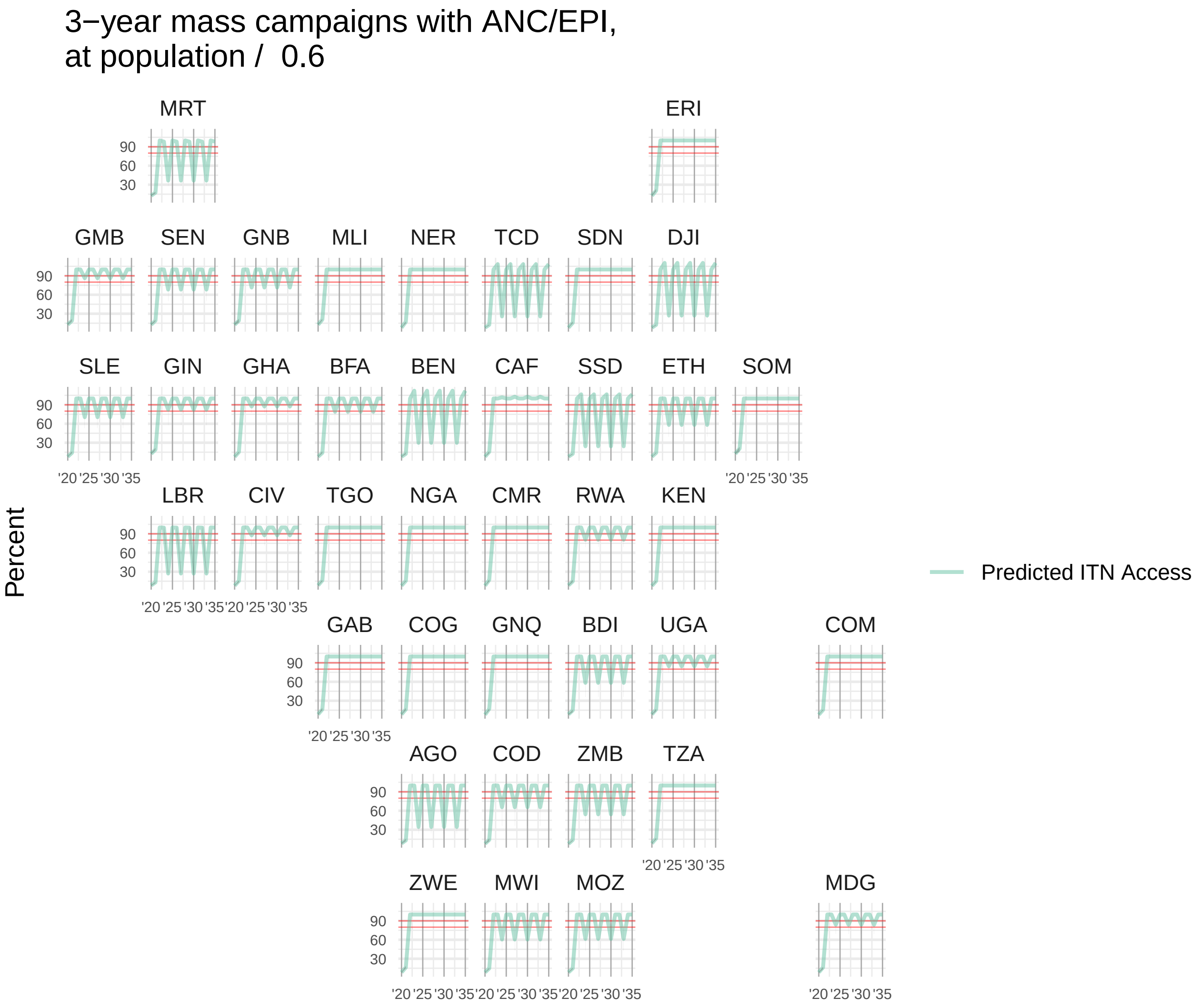

3−year mass campaigns with ANC/EPI,
at population / 0.6
MRT
ERI
GMB
SEN
GNB
MLI
NER
TCD
SDN
DJI
SLE
GIN
GHA
BFA
BEN
CAF
SSD
ETH
SOM
LBR
CIV
TGO
NGA
CMR
RWA
KEN
GAB
COG
GNQ
BDI
UGA
COM
AGO
COD
ZMB
TZA
ZWE
MWI
MOZ
MDG
Percent
90
60
30
'20 '25 '30 '35
Predicted ITN Access

3−year mass campaigns with ANC/EPI,
at population / 0.7

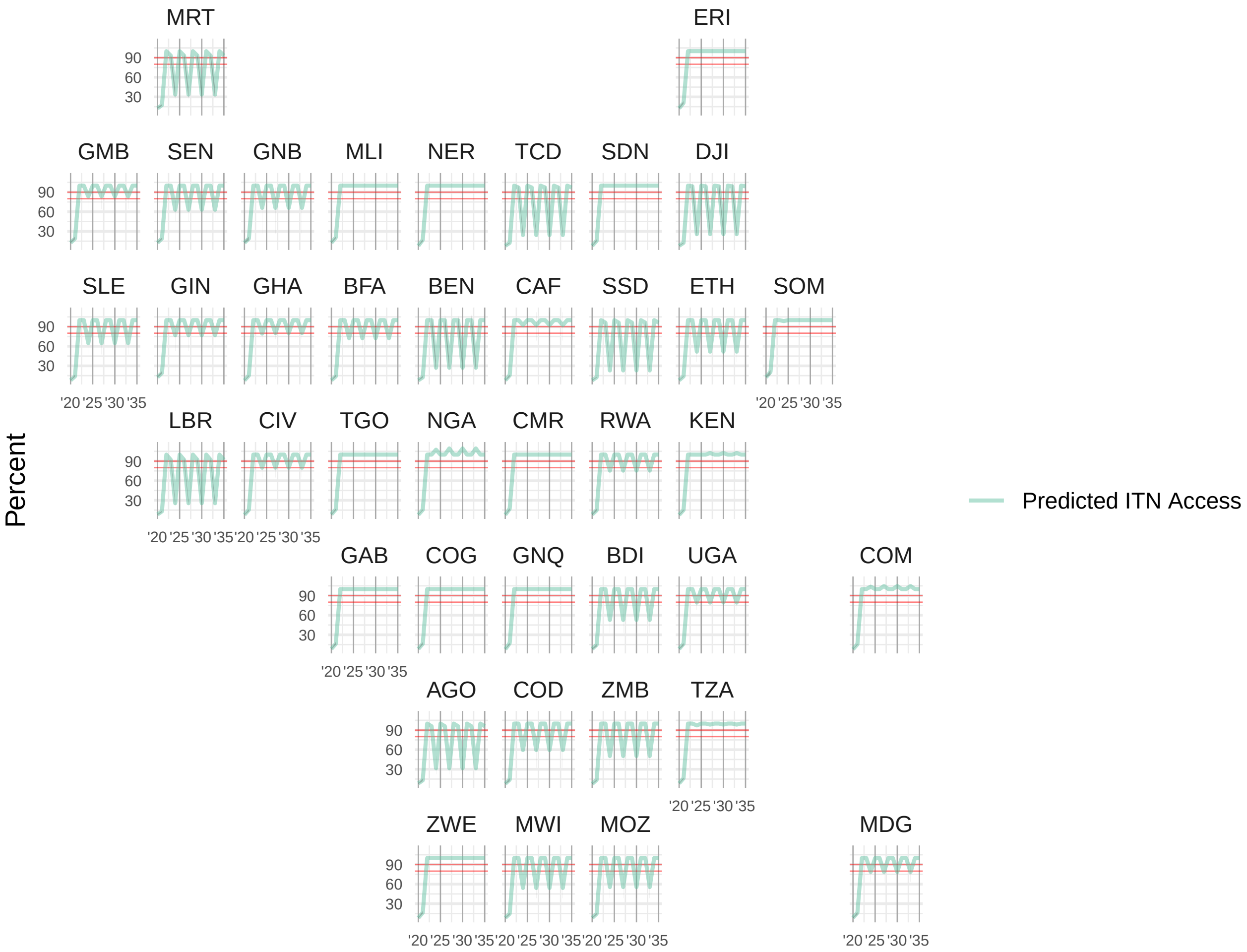

3−year mass campaigns with ANC/EPI,
at population / 0.8

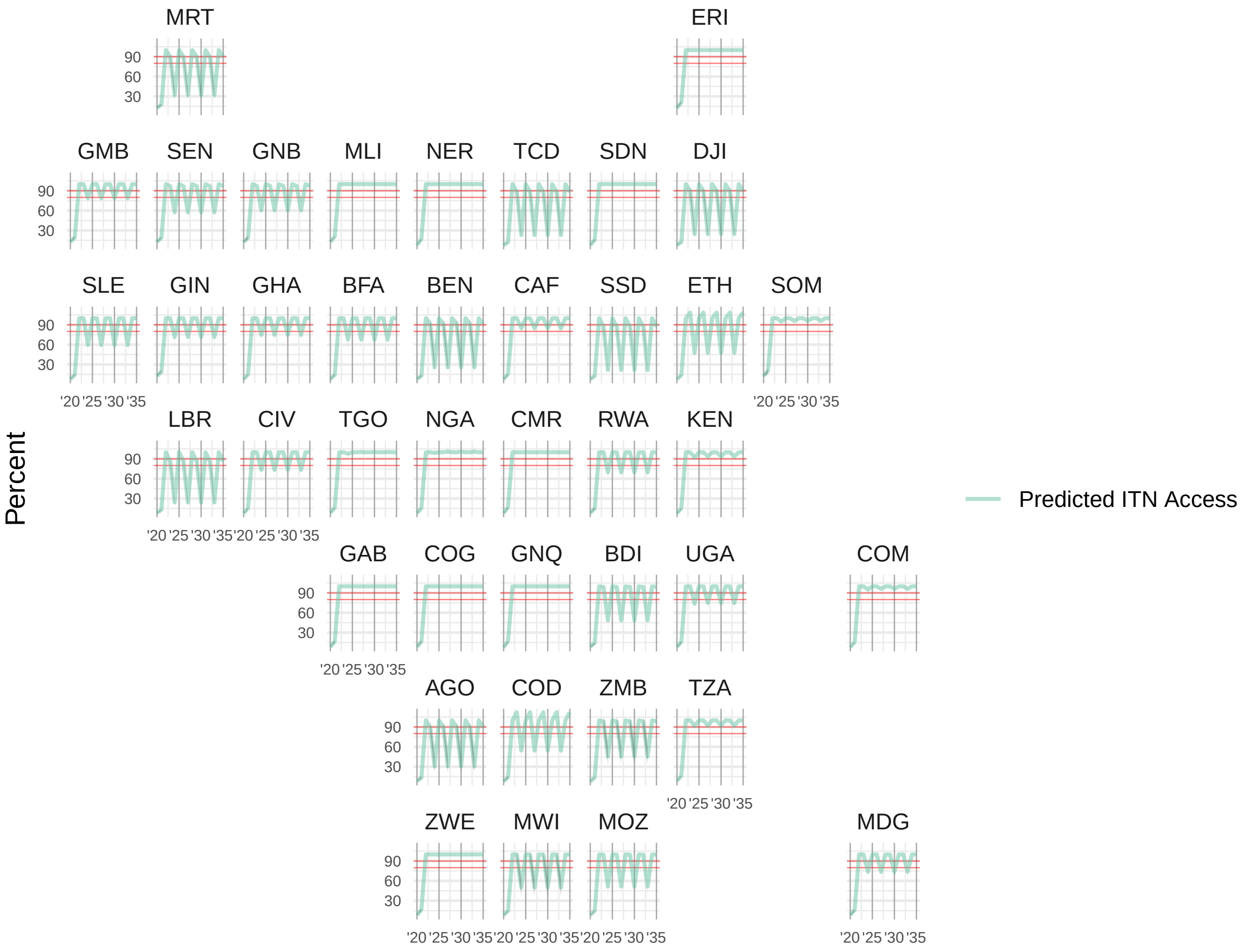

3−year mass campaigns with ANC/EPI,
at population / 0.9

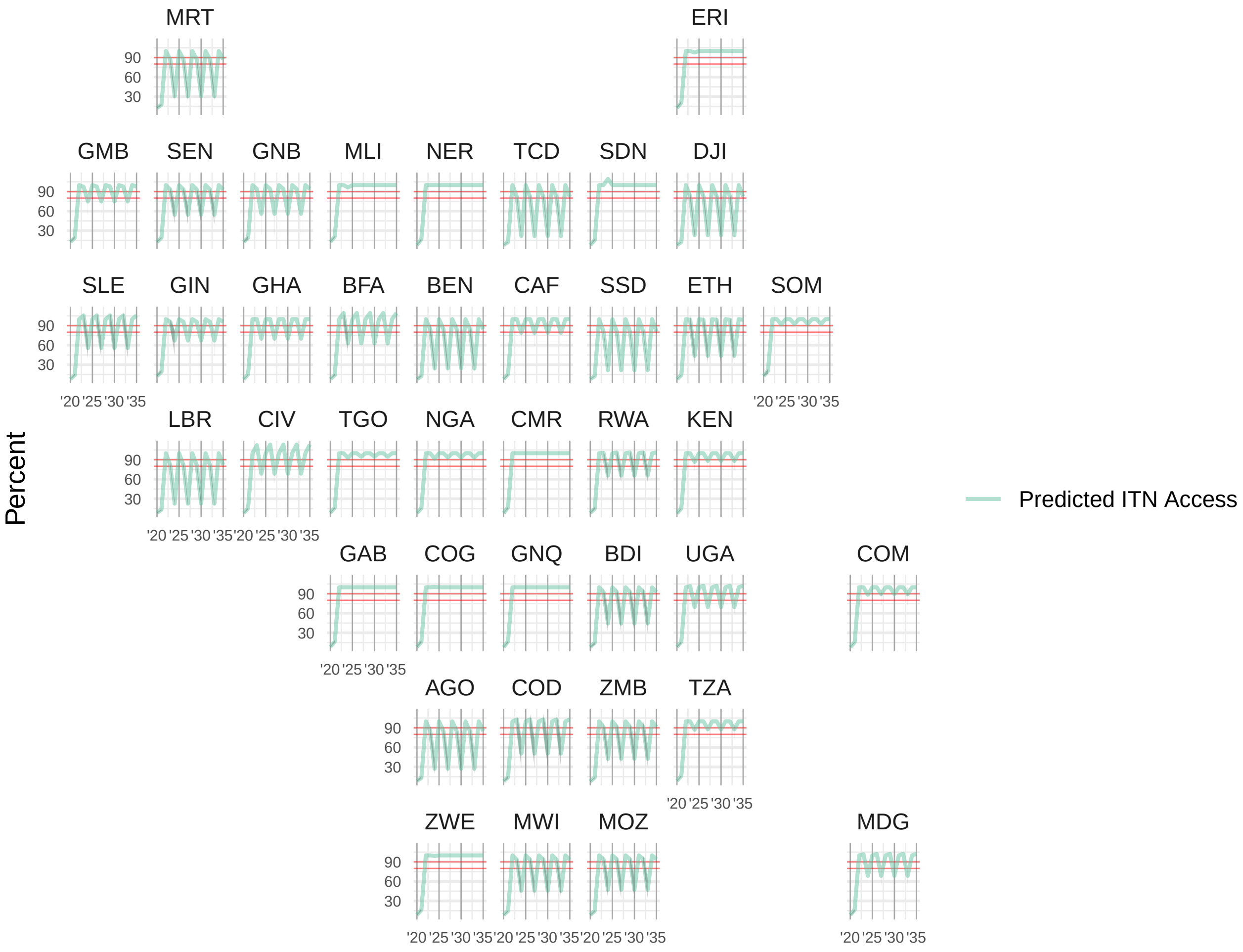

3−year mass campaigns with ANC/EPI,
at population / 1

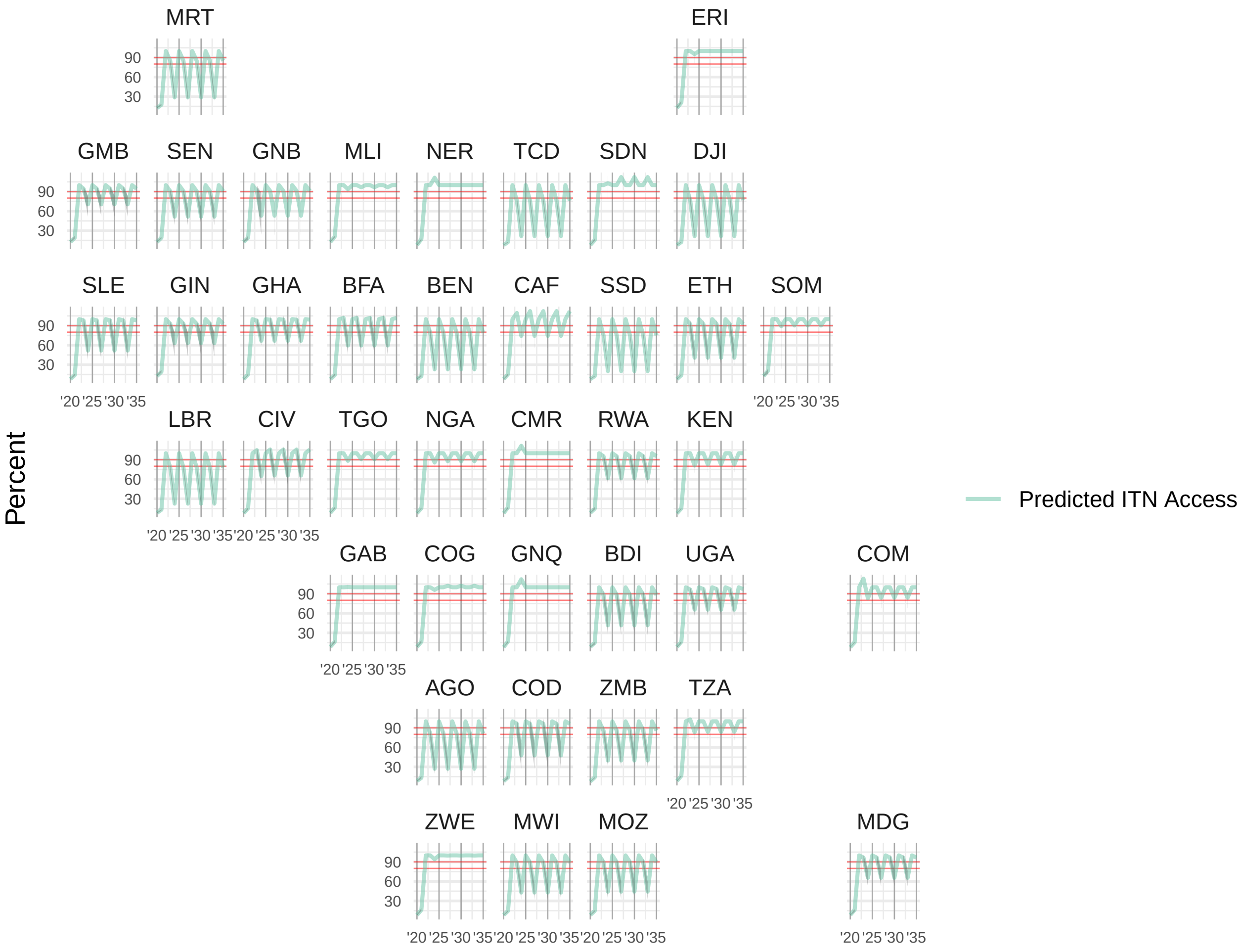

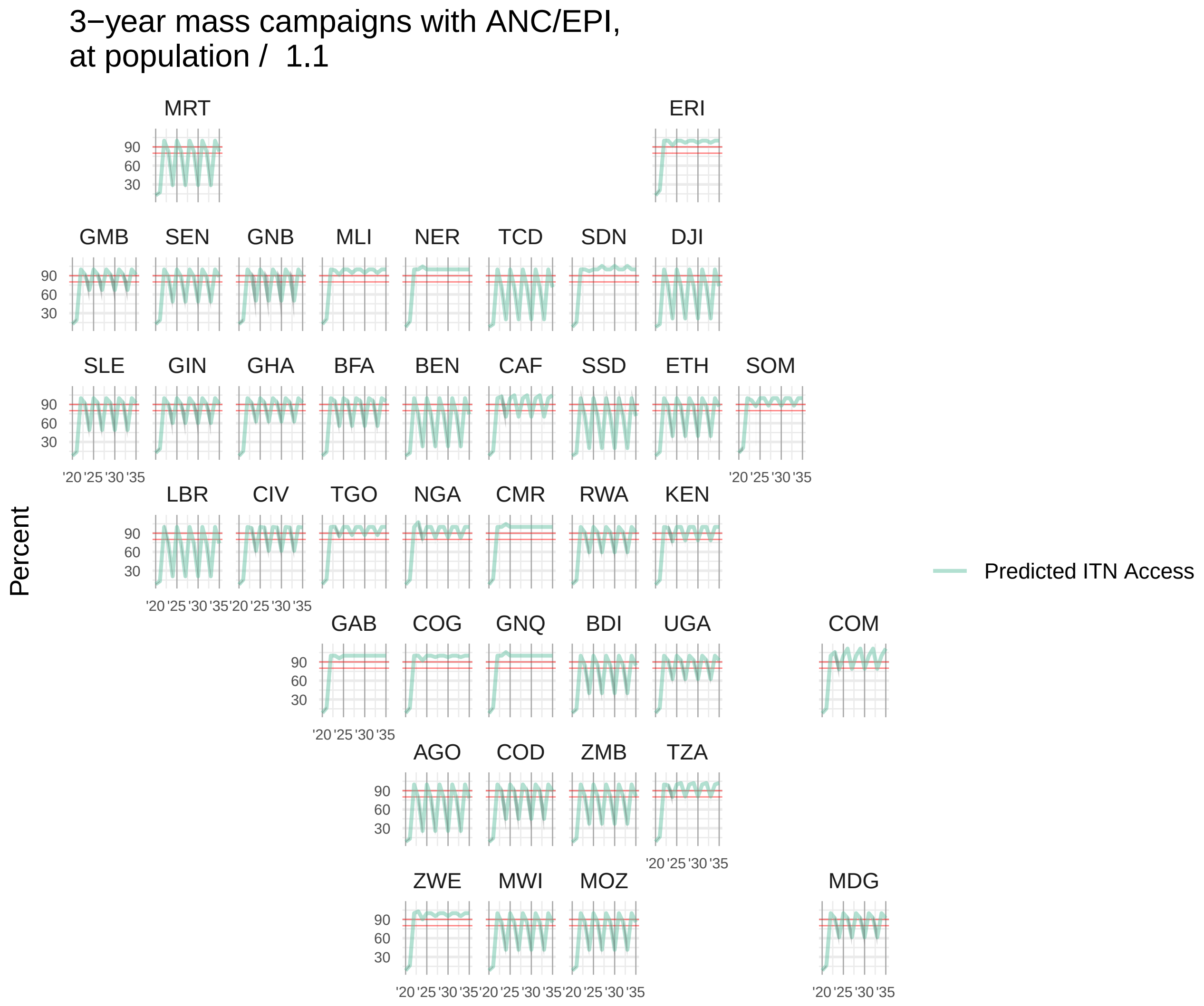

3−year mass campaigns with ANC/EPI,
at population / 1.1
MRT
ERI
GMB
SEN
GNB
MLI
NER
TCD
SDN
DJI
SLE
GIN
GHA
BFA
BEN
CAF
SSD
ETH
SOM
LBR
CIV
TGO
NGA
CMR
RWA
KEN
GAB
COG
GNQ
BDI
UGA
COM
AGO
COD
ZMB
TZA
ZWE
MWI
MOZ
MDG
90
60
30
'20 '25 '30 '35
Percent
Predicted ITN Access

3−year mass campaigns with ANC/EPI,
at population / 1.2

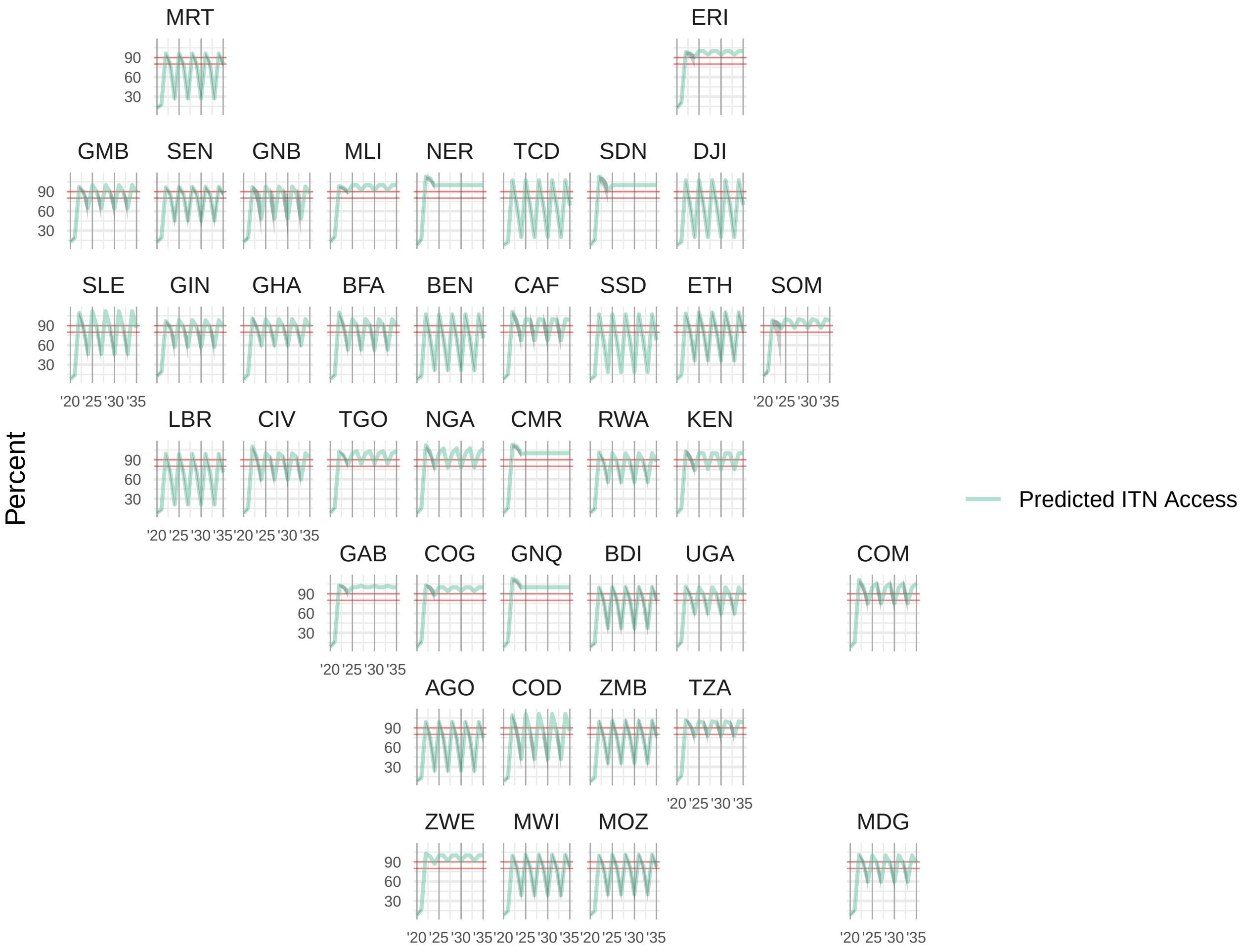

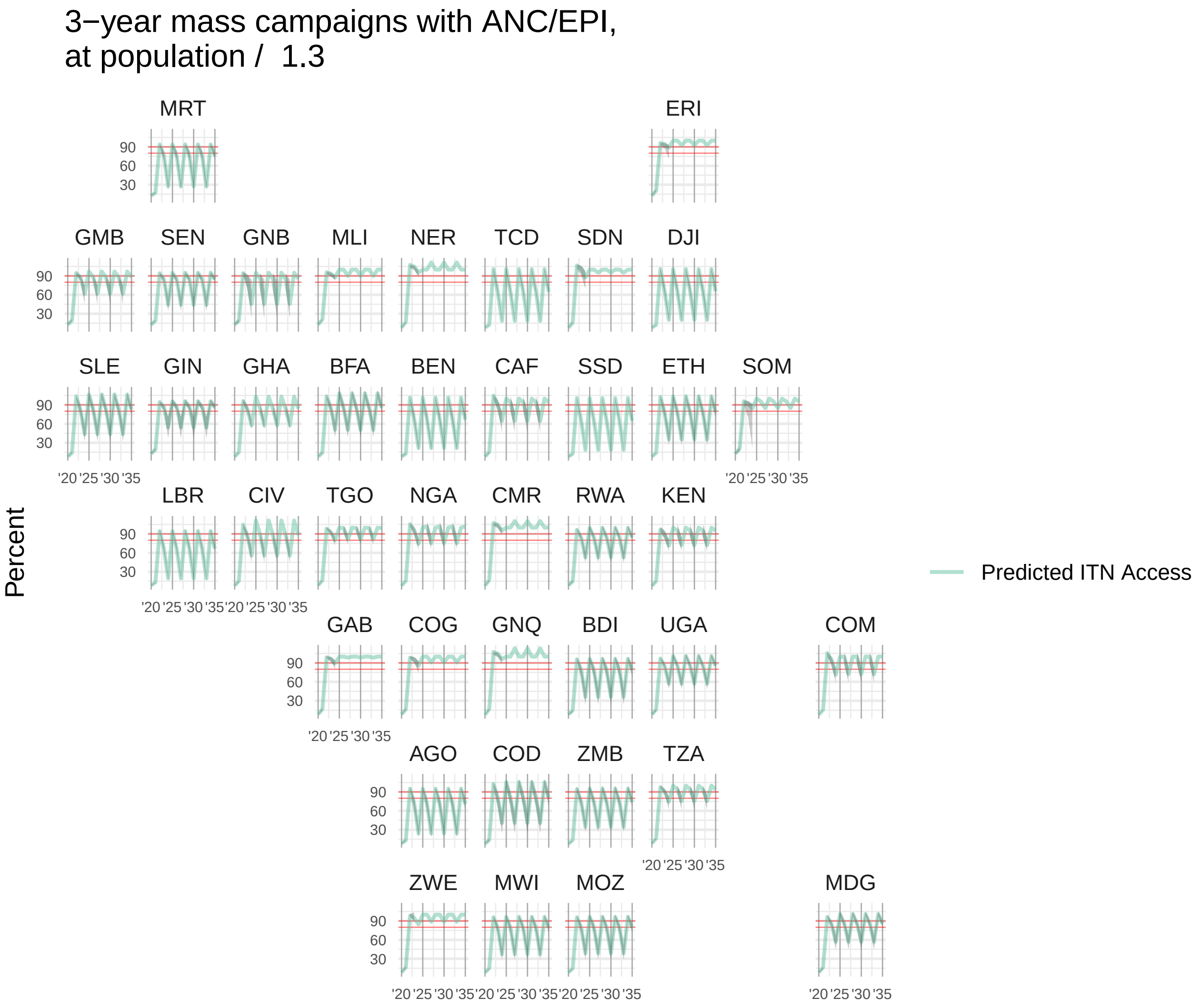
3−year mass campaigns with ANC/EPI,
at population / 1.3
MRT
ERI
GMB
SEN
GNB
MLI
NER
TCD
SDN
DJI
SLE
GIN
GHA
BFA
BEN
CAF
SSD
ETH
SOM
LBR
CIV
TGO
NGA
CMR
RWA
KEN
GAB
COG
GNQ
BDI
UGA
COM
AGO
COD
ZMB
TZA
ZWE
MWI
MOZ
MDG
Percent
90
60
30
'20 '25 '30 '35
Predicted ITN Access

3−year mass campaigns with ANC/EPI, at population / 1.4

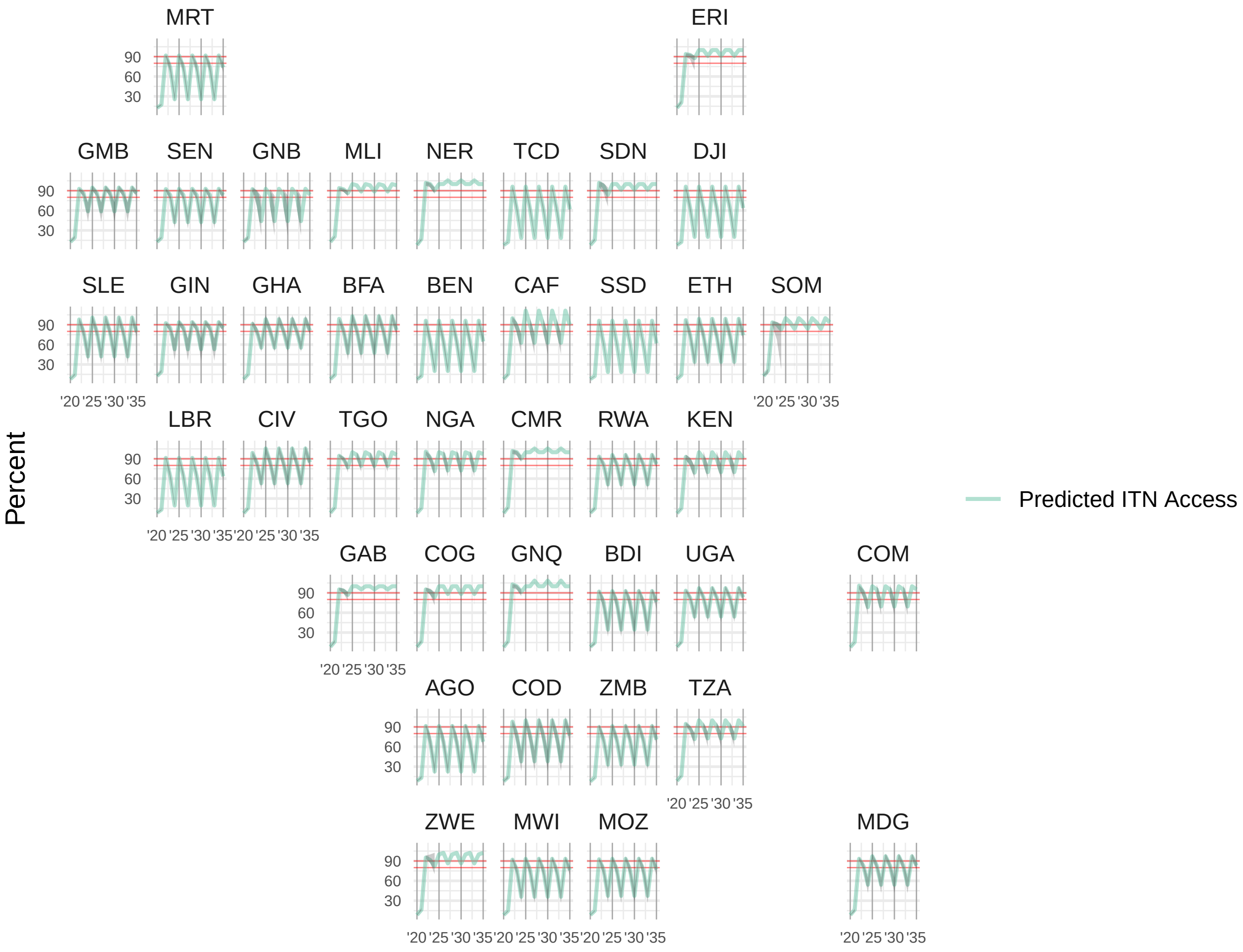

3−year mass campaigns with ANC/EPI,
at population / 1.5

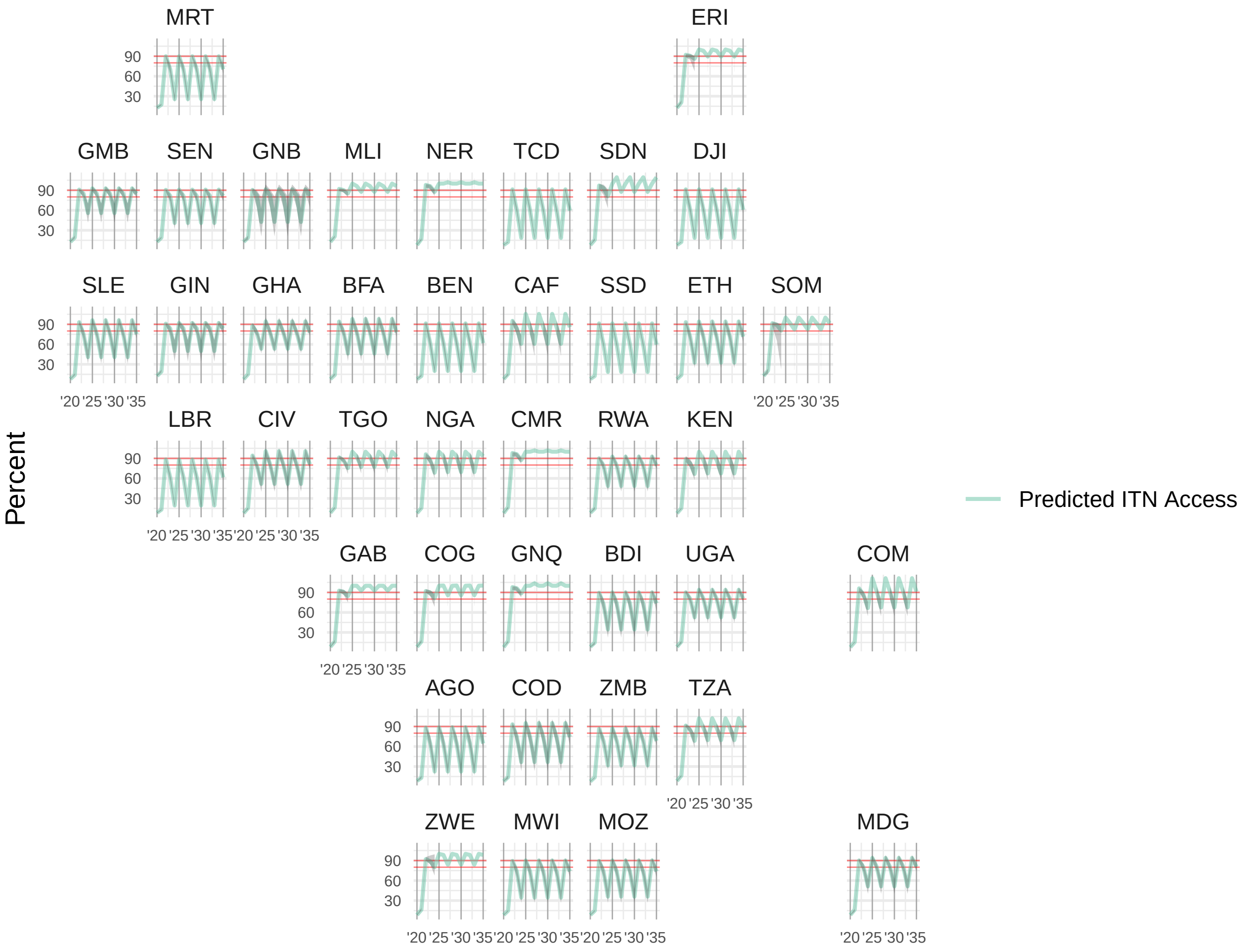

3−year mass campaigns with ANC/EPI, at population / 1.6

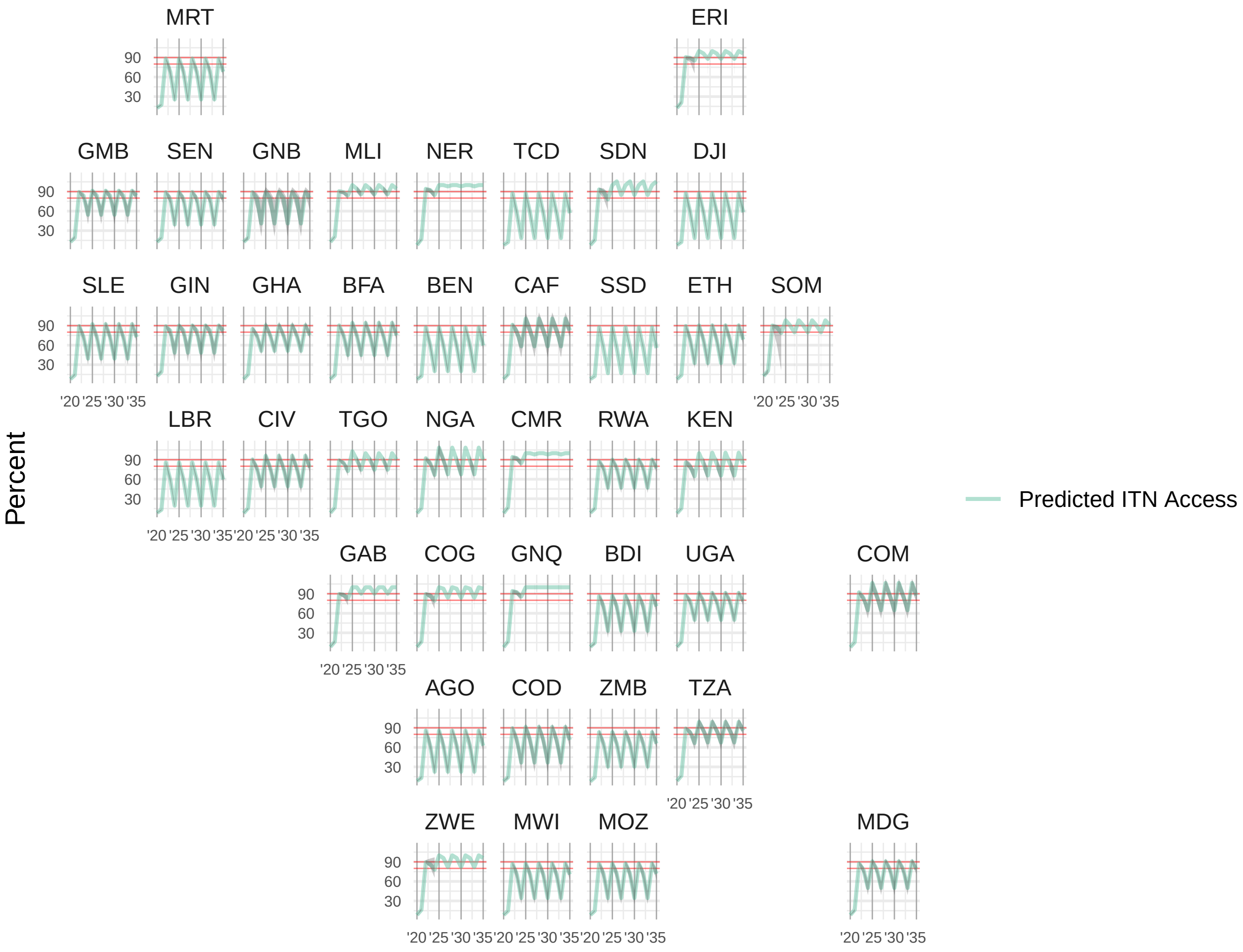

3−year mass campaigns with ANC/EPI,
at population / 1.7

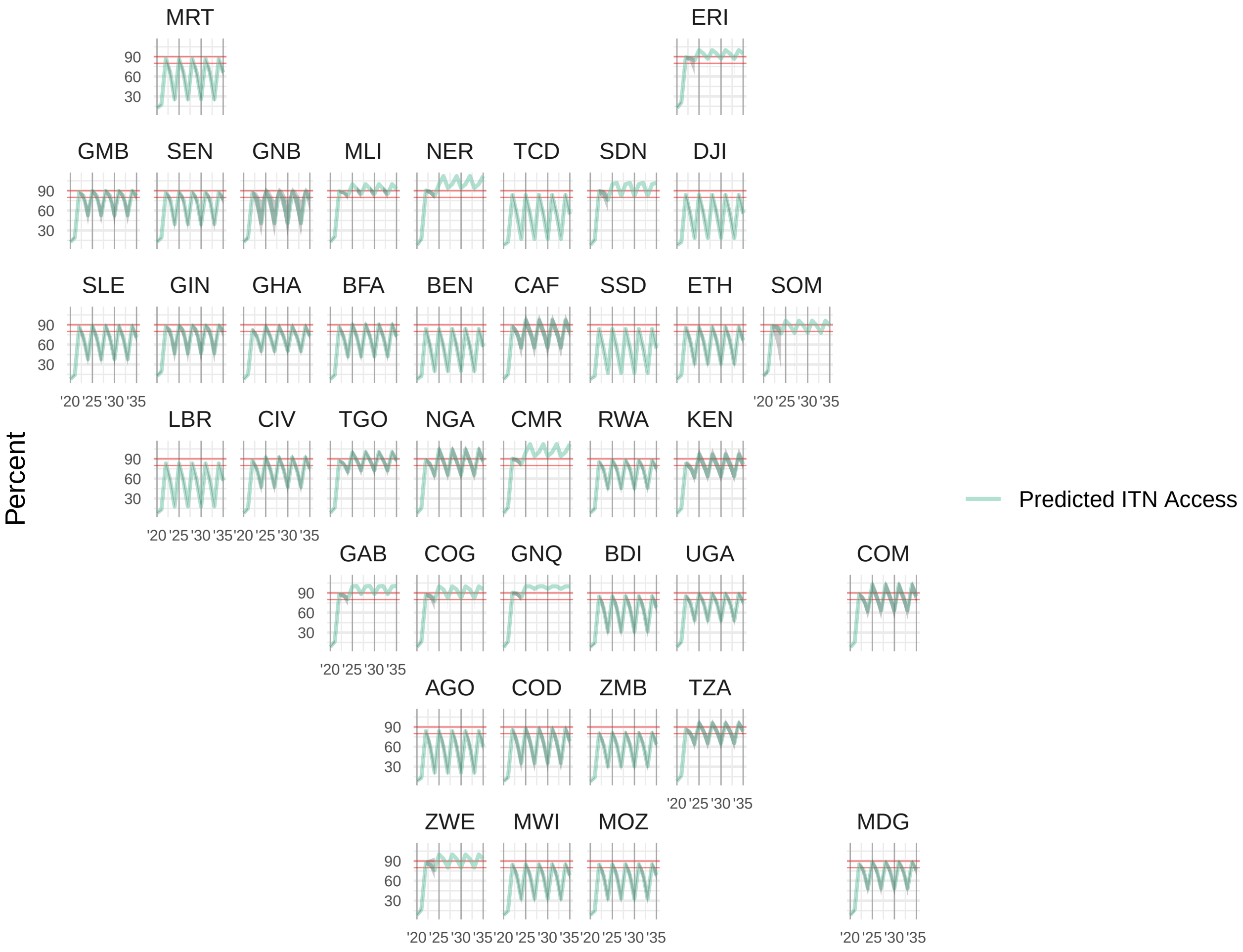

3−year mass campaigns with ANC/EPI, at population / 1.8

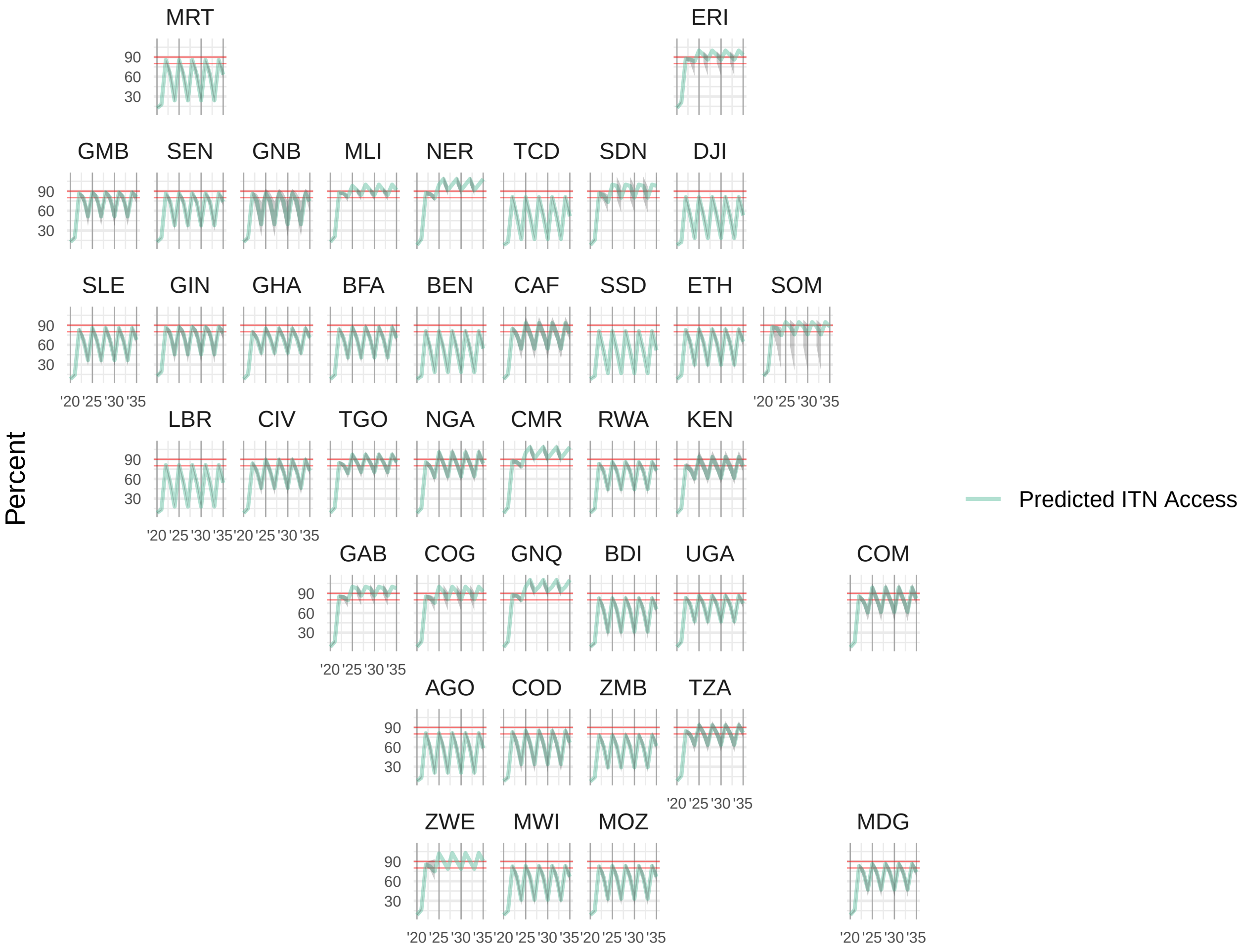

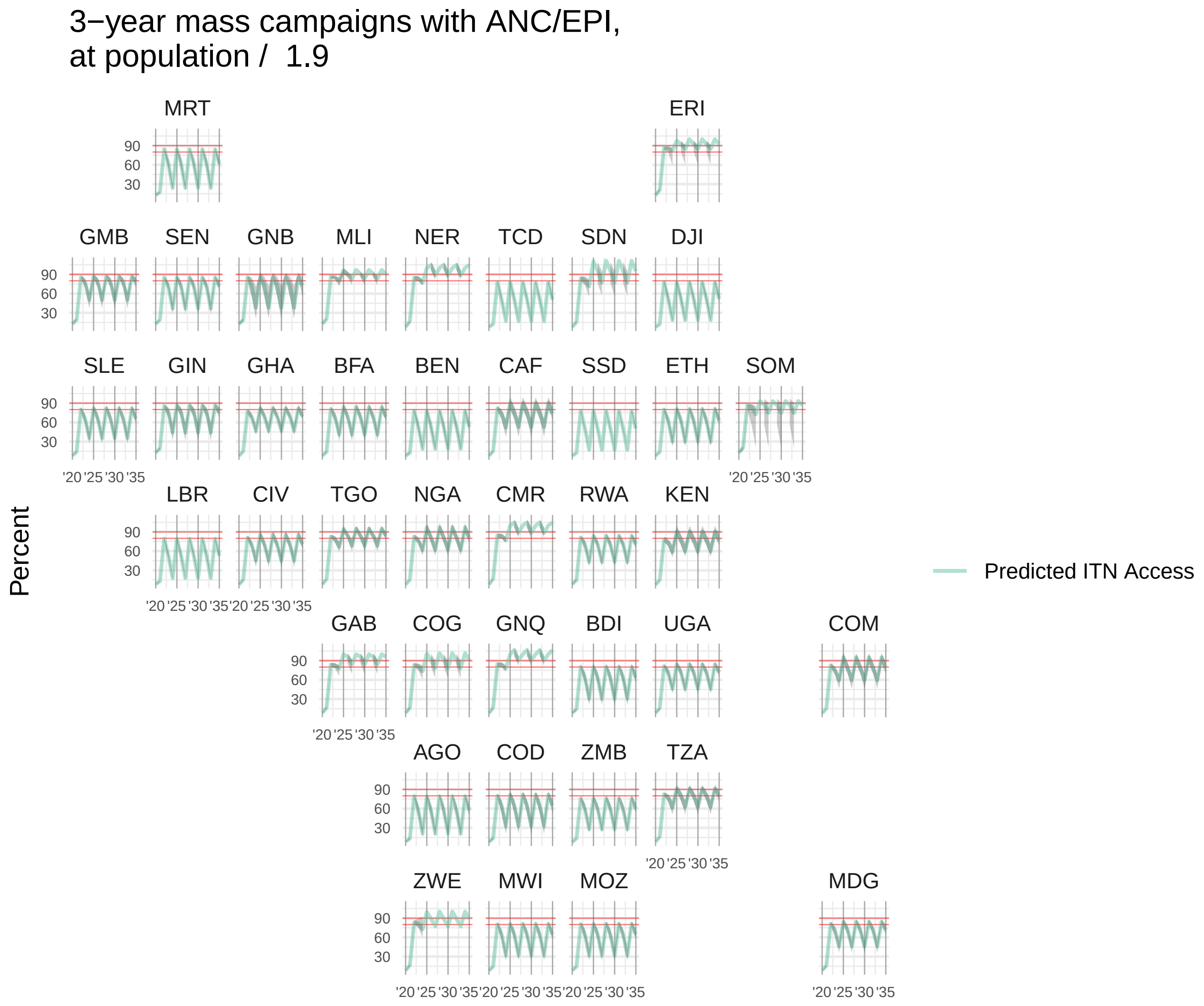
3−year mass campaigns with ANC/EPI,
at population / 1.9
MRT
ERI
GMB
SEN
GNB
MLI
NER
TCD
SDN
DJI
SLE
GIN
GHA
BFA
BEN
CAF
SSD
ETH
SOM
LBR
CIV
TGO
NGA
CMR
RWA
KEN
GAB
COG
GNQ
BDI
UGA
COM
AGO
COD
ZMB
TZA
ZWE
MWI
MOZ
MDG
Percent
90
60
30
'20 '25 '30 '35
Predicted ITN Access

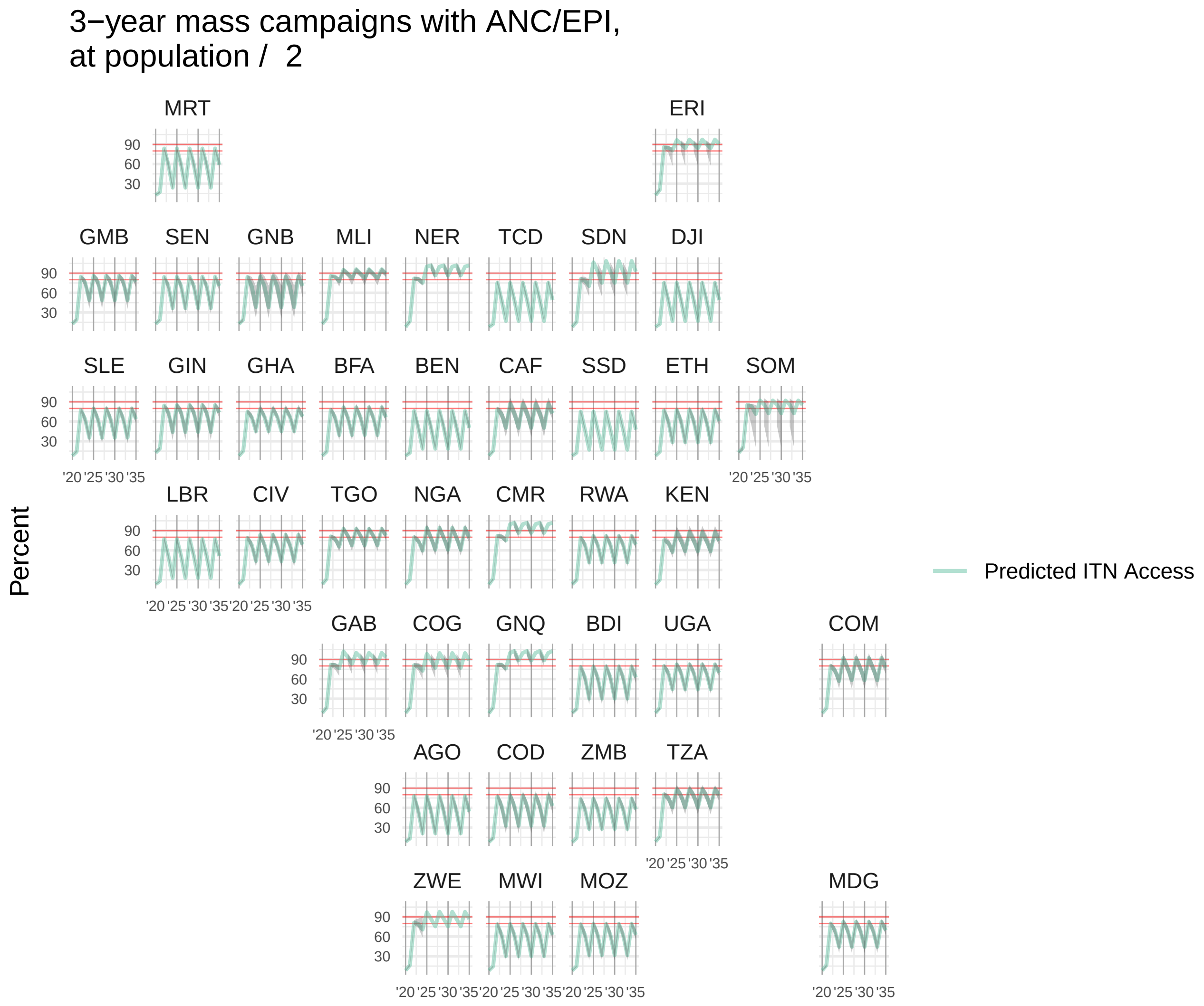
3−year mass campaigns with ANC/EPI,
at population / 2
MRT
ERI
GMB
SEN
GNB
MLI
NER
TCD
SDN
DJI
SLE
GIN
GHA
BFA
BEN
CAF
SSD
ETH
SOM
LBR
CIV
TGO
NGA
CMR
RWA
KEN
GAB
COG
GNQ
BDI
UGA
COM
AGO
COD
ZMB
TZA
ZWE
MWI
MOZ
MDG
90
60
30
'20 '25 '30 '35
Percent
Predicted ITN Access

5 Scenario 5 - Two-year mass campaigns with ANC/EPI distribution at 6%, varying campaign quantifier

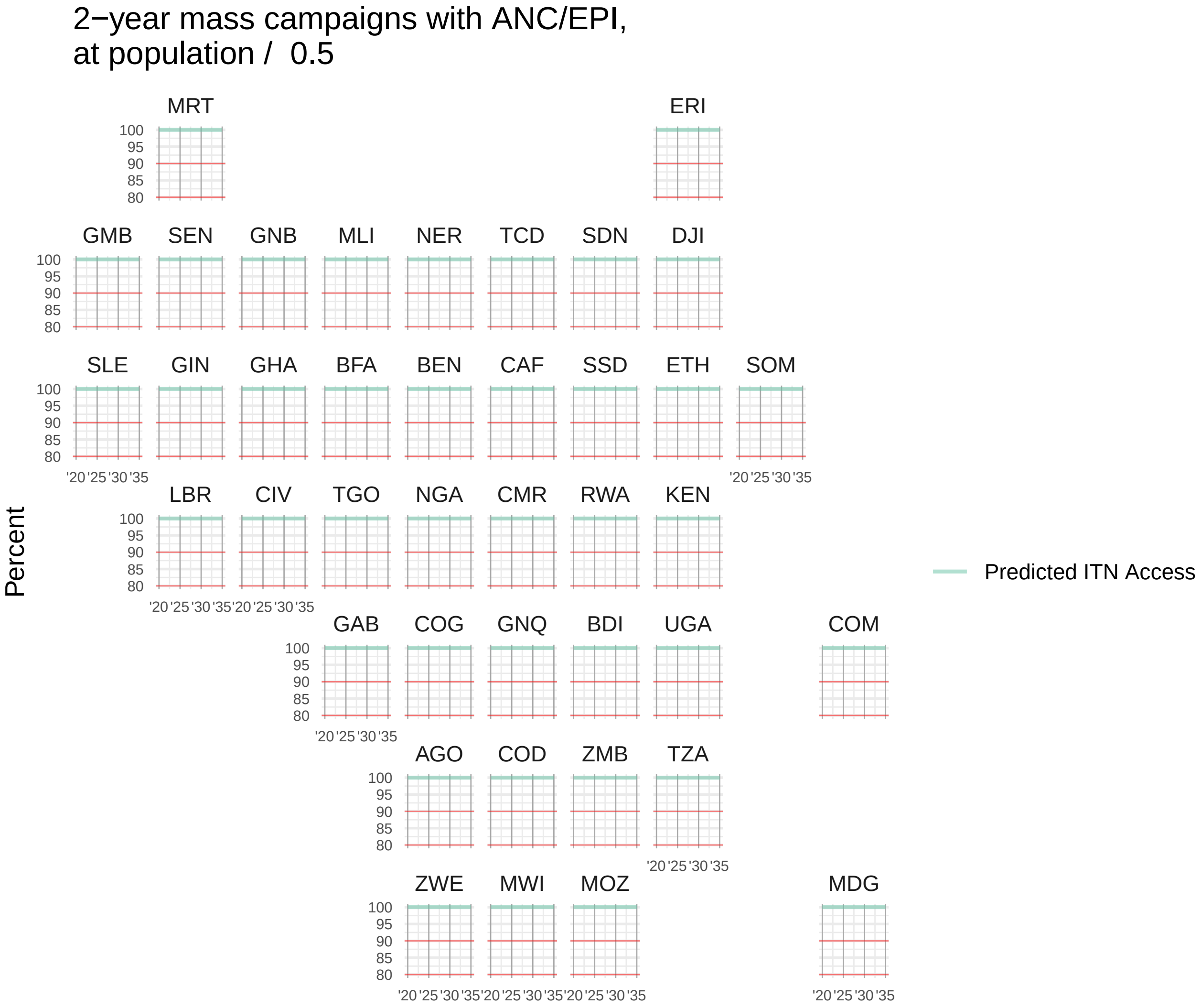

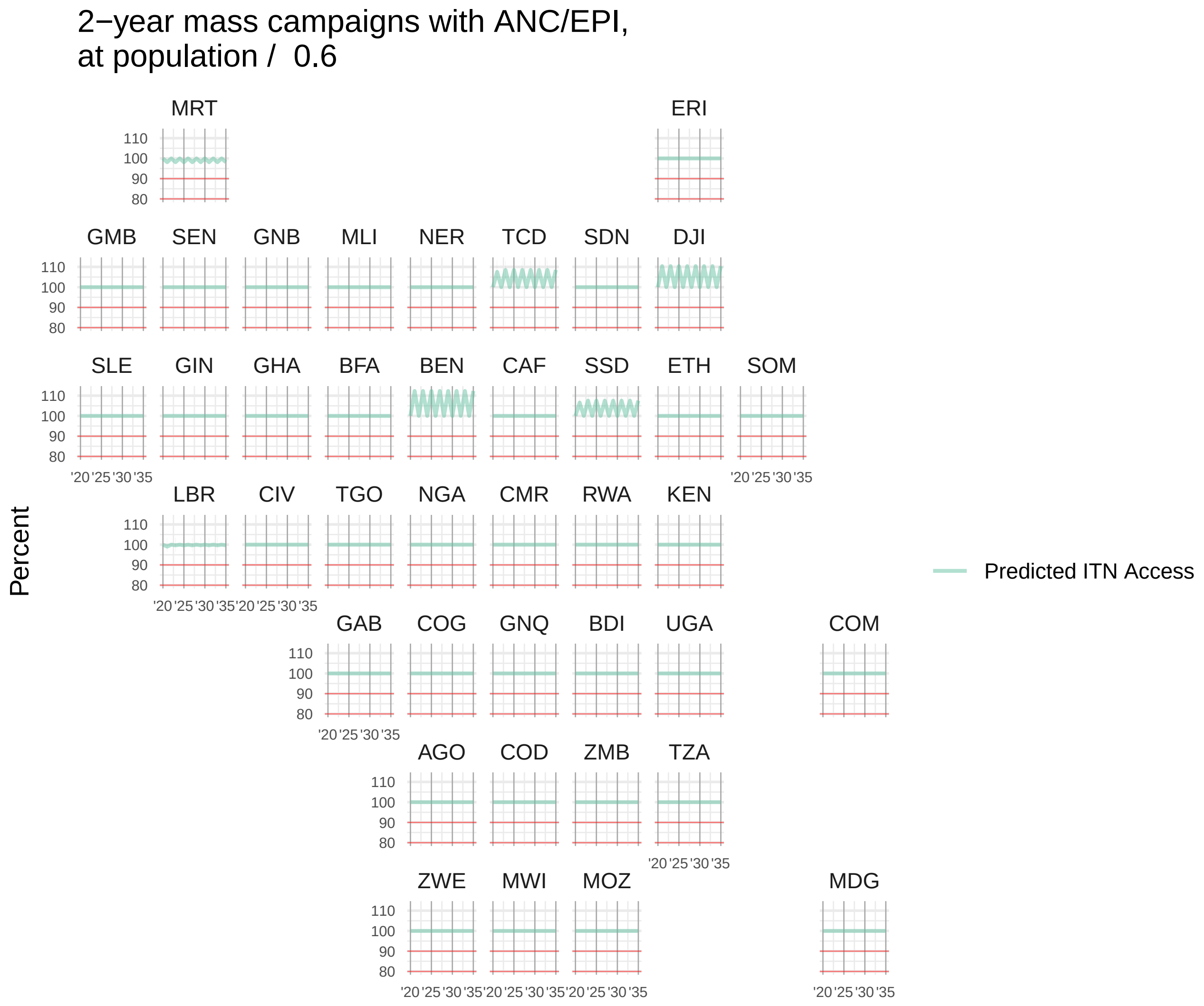
2−year mass campaigns with ANC/EPI,
at population / 0.6
MRT
ERI
GMB
SEN
GNB
MLI
NER
TCD
SDN
DJI
SLE
GIN
GHA
BFA
BEN
CAF
SSD
ETH
SOM
LBR
CIV
TGO
NGA
CMR
RWA
KEN
GAB
COG
GNQ
BDI
UGA
COM
AGO
COD
ZMB
TZA
ZWE
MWI
MOZ
MDG
Percent
110
100
90
80
'20 '25 '30 '35
Predicted ITN Access

2−year mass campaigns with ANC/EPI, at population / 0.7

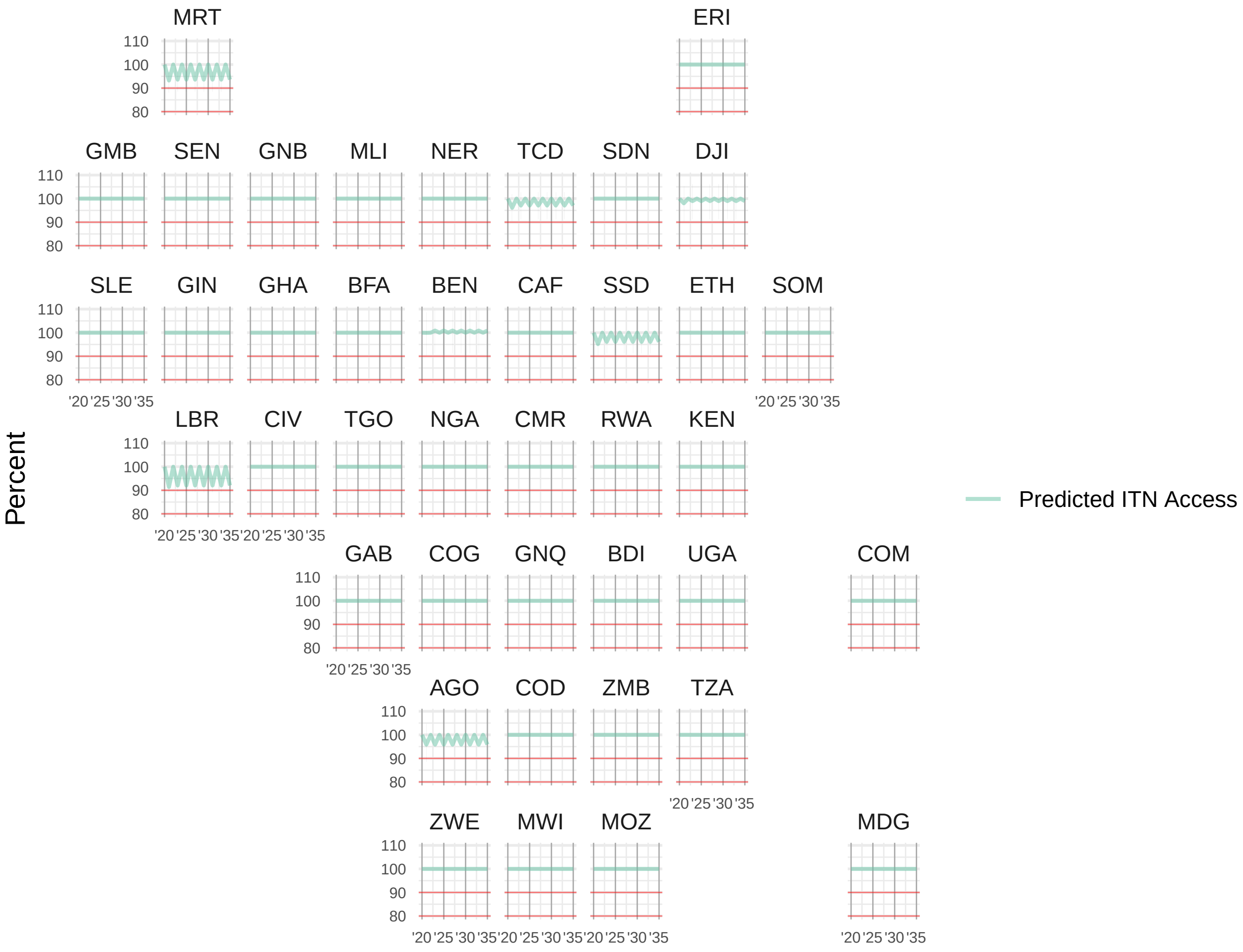

2−year mass campaigns with ANC/EPI,
at population / 0.8

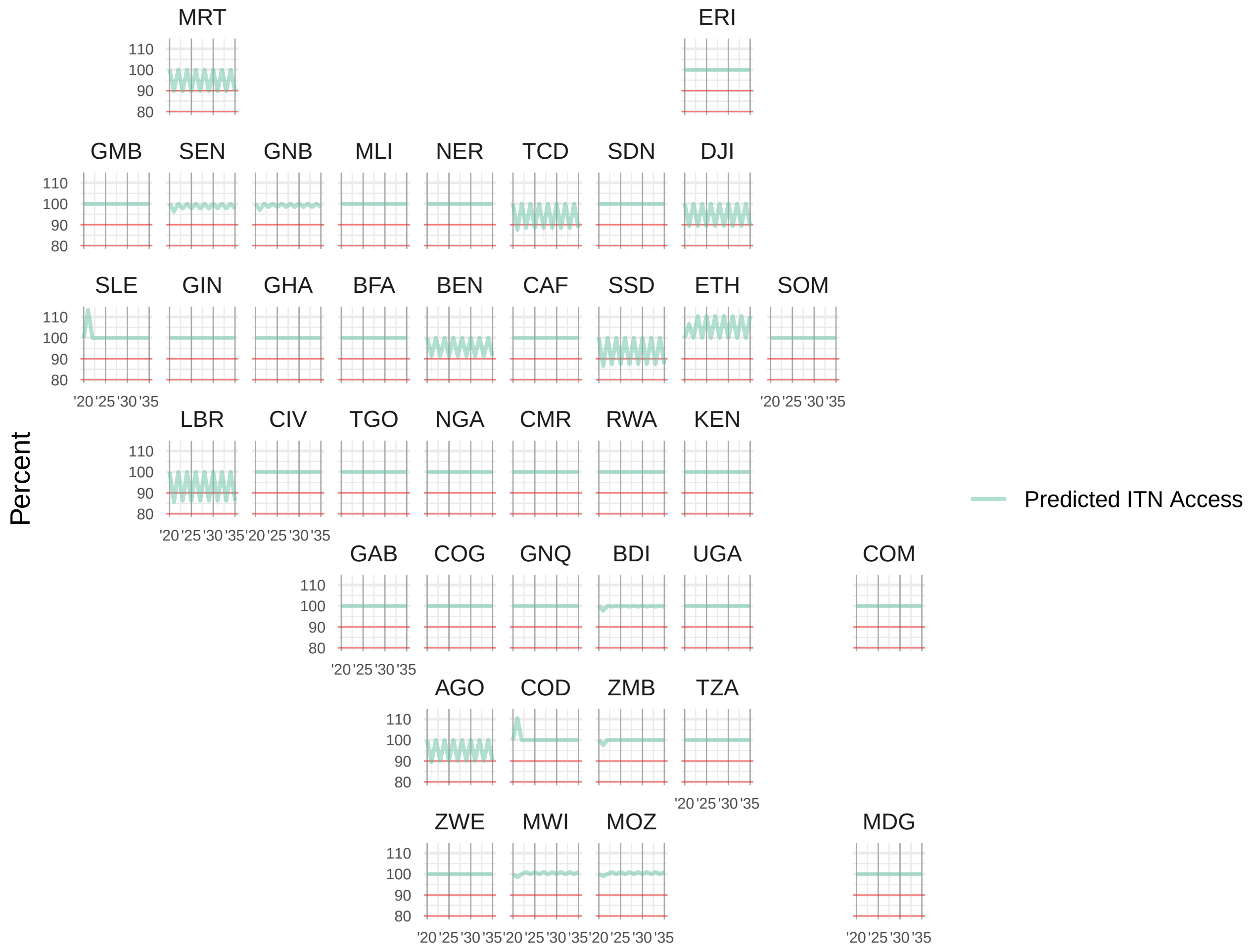

2−year mass campaigns with ANC/EPI,
at population / 0.9

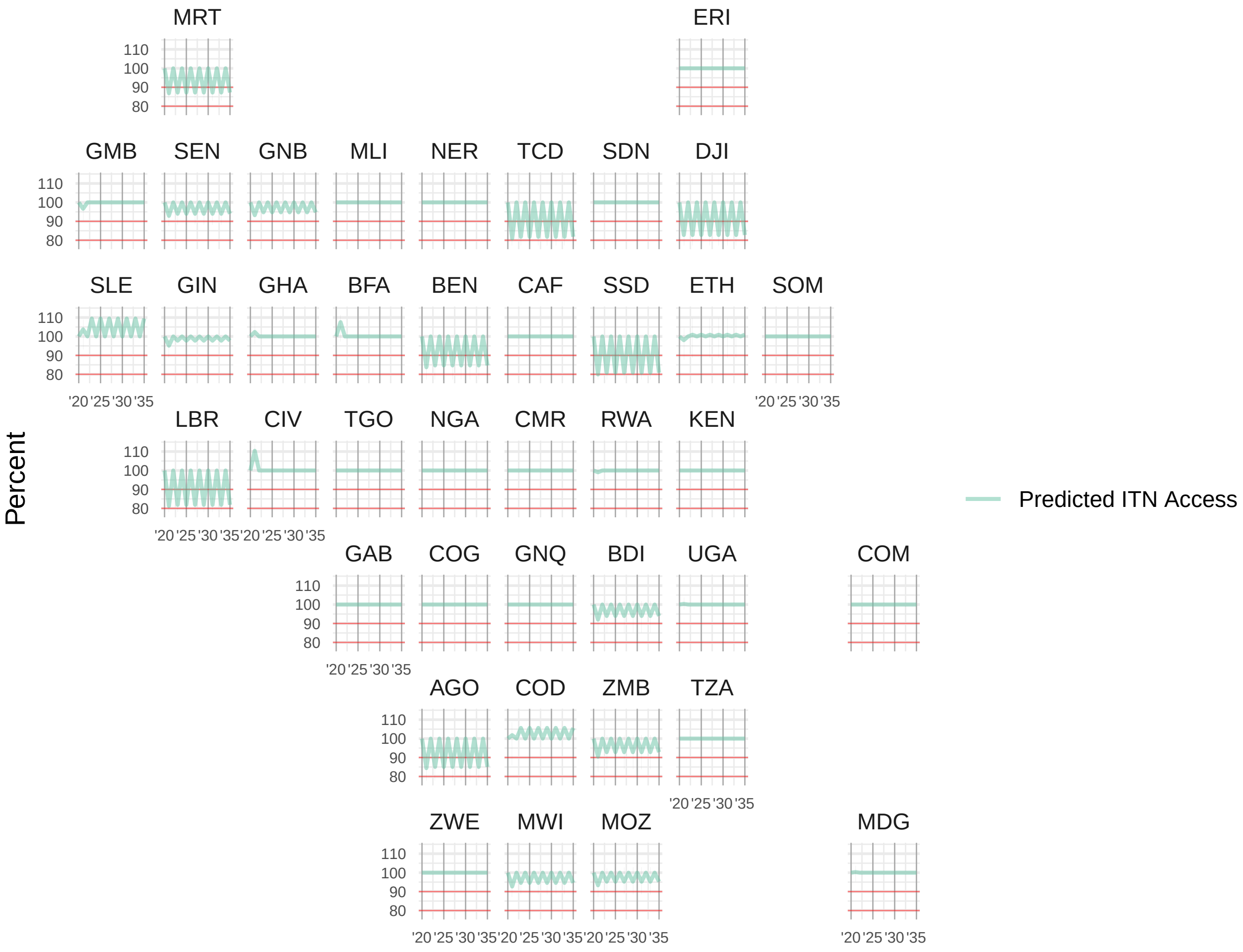

2−year mass campaigns with ANC/EPI,
at population / 1

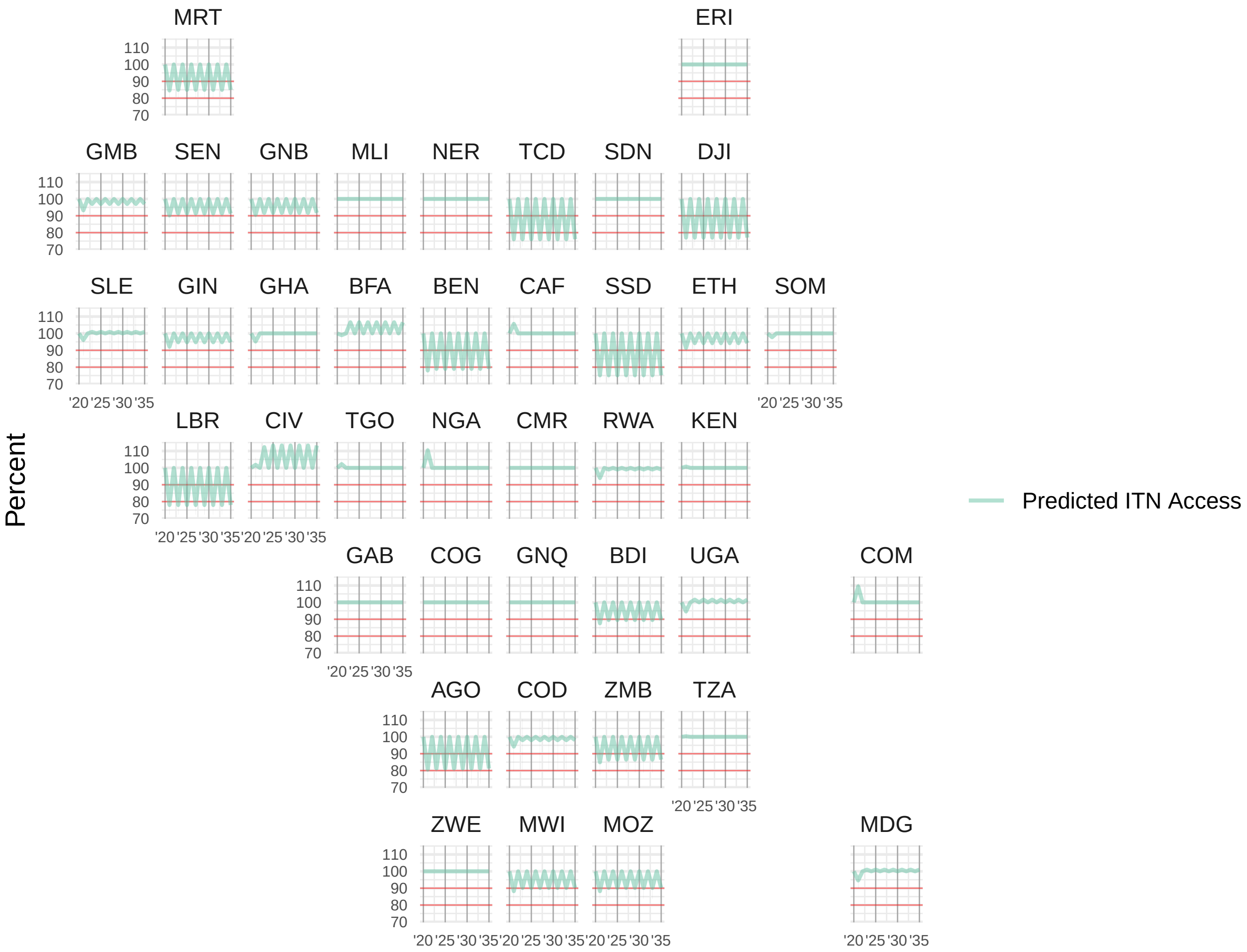

2−year mass campaigns with ANC/EPI,
at population / 1.1

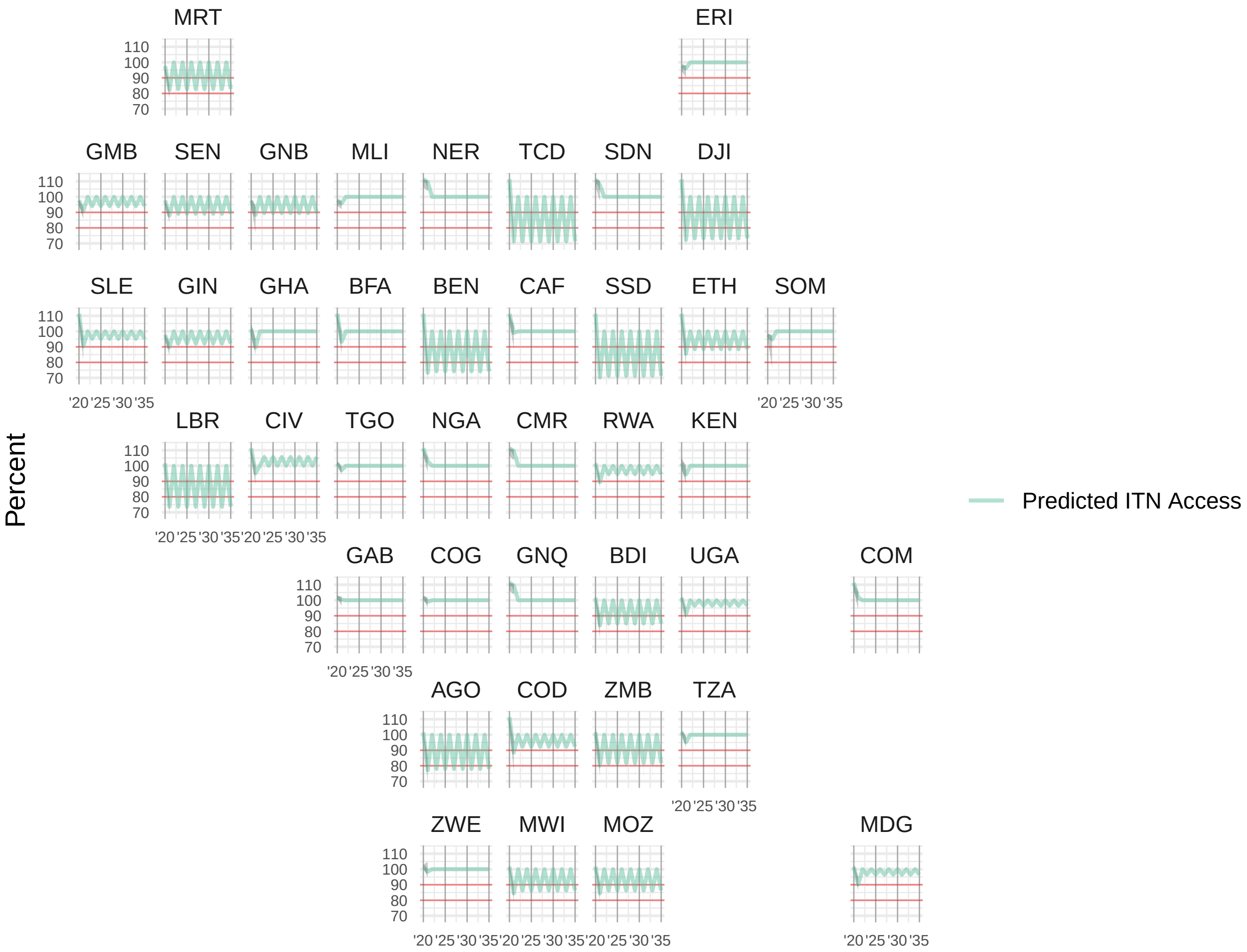

2−year mass campaigns with ANC/EPI,
at population / 1.2

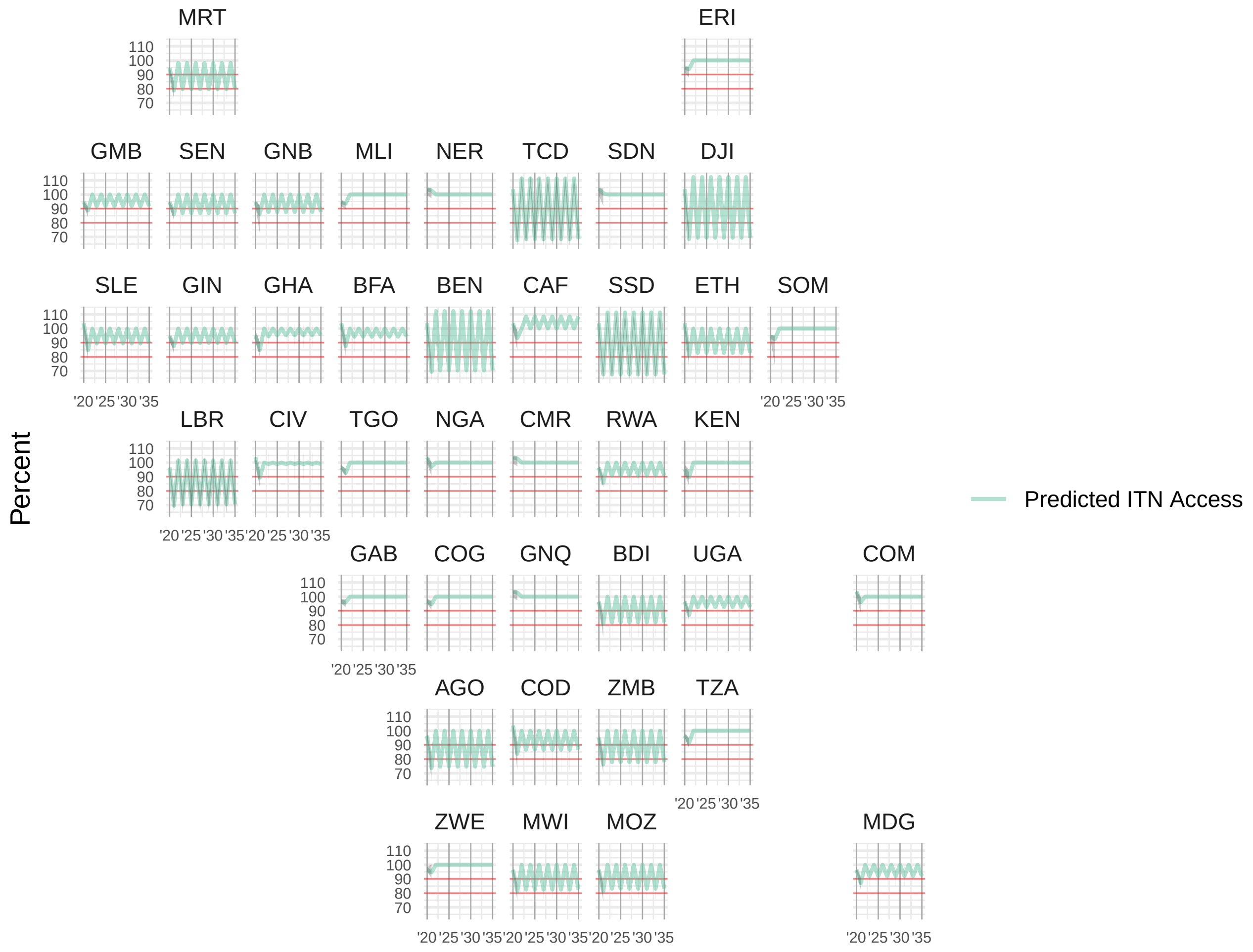

2−year mass campaigns with ANC/EPI,
at population / 1.3

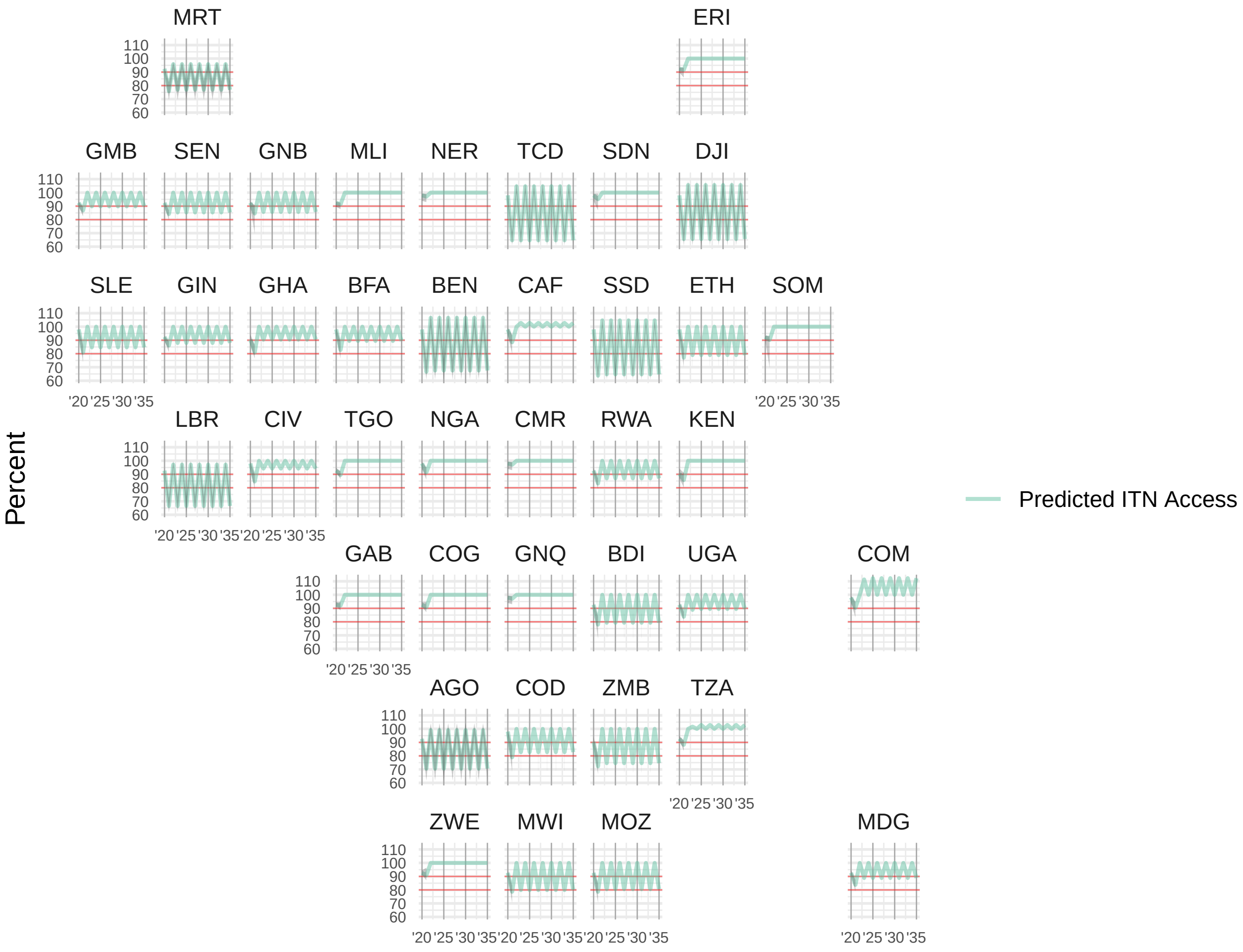

2−year mass campaigns with ANC/EPI,
at population / 1.4

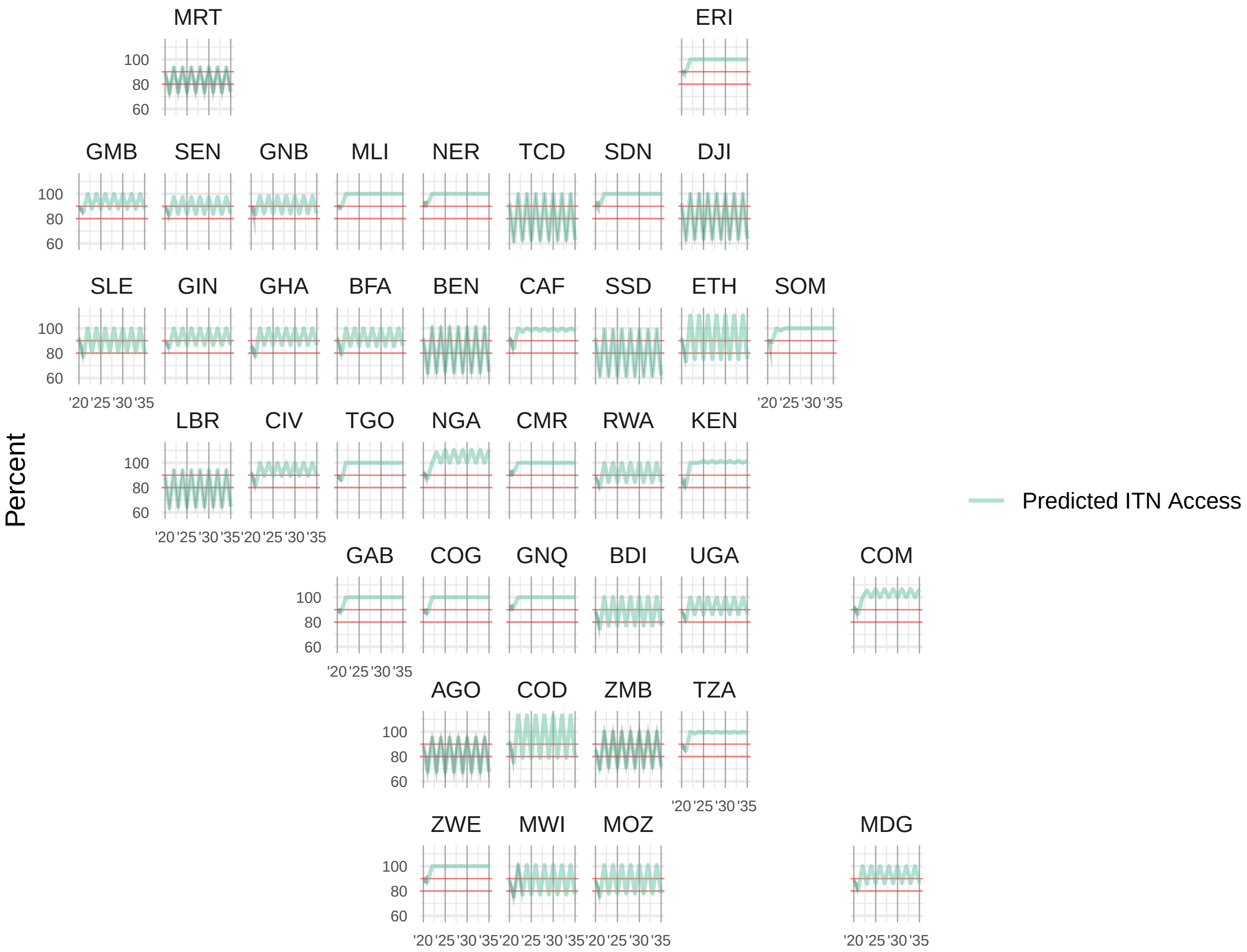

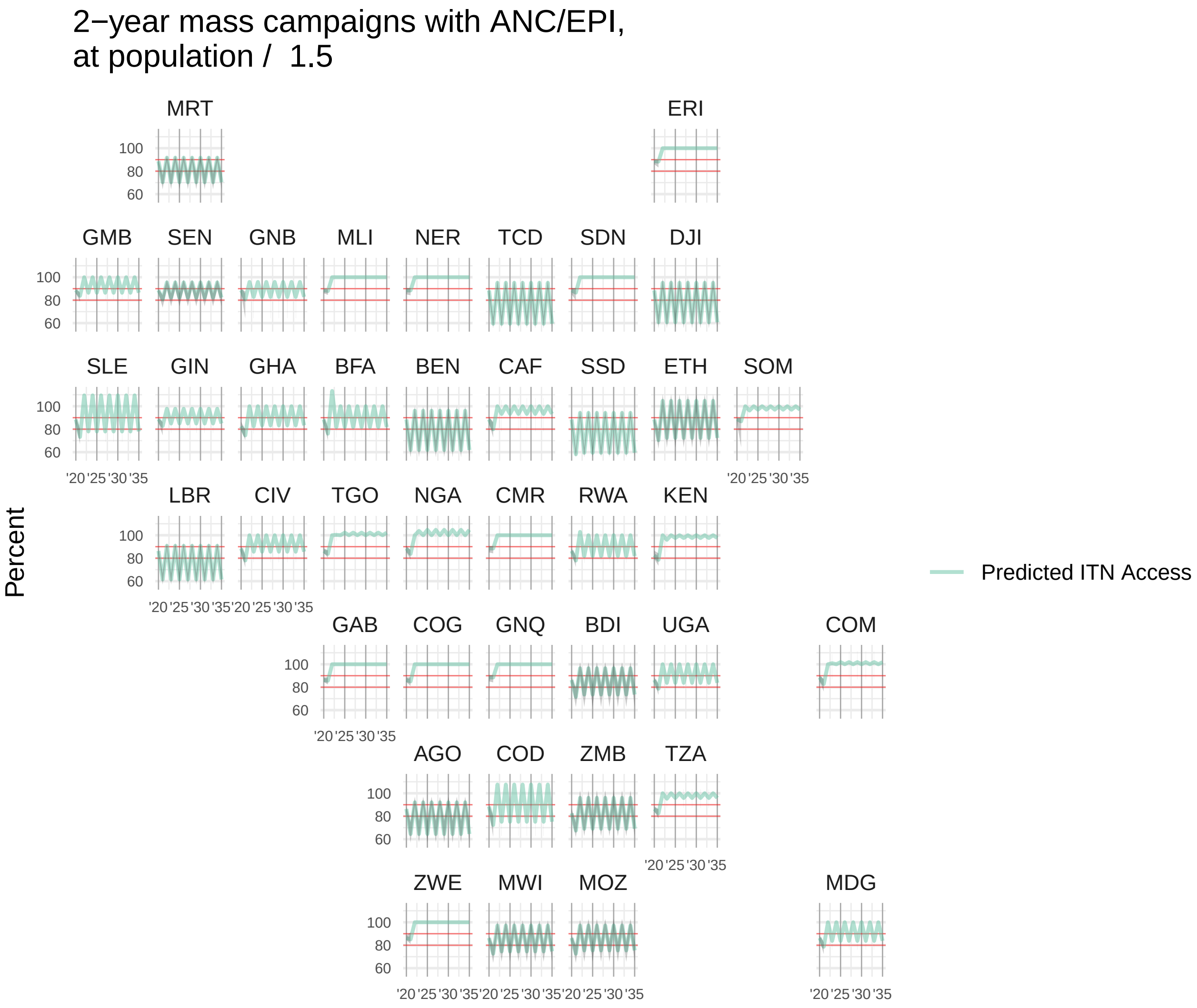
2−year mass campaigns with ANC/EPI,
at population / 1.5
MRT
ERI
GMB
SEN
GNB
MLI
NER
TCD
SDN
DJI
SLE
GIN
GHA
BFA
BEN
CAF
SSD
ETH
SOM
LBR
CIV
TGO
NGA
CMR
RWA
KEN
GAB
COG
GNQ
BDI
UGA
COM
AGO
COD
ZMB
TZA
ZWE
MWI
MOZ
MDG
100
80
60
'20 '25 '30 '35
Percent
Predicted ITN Access

2−year mass campaigns with ANC/EPI, at population / 1.6

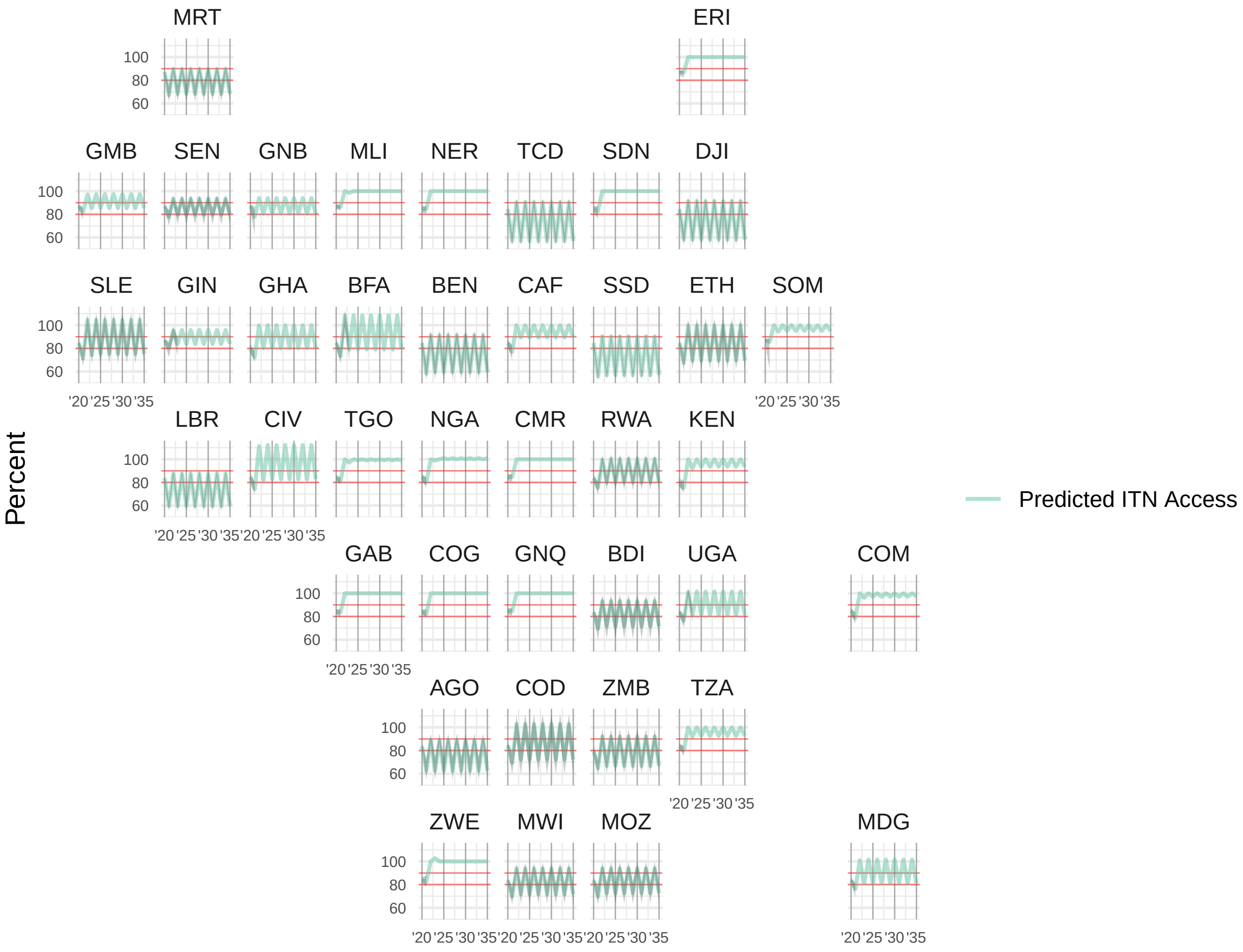

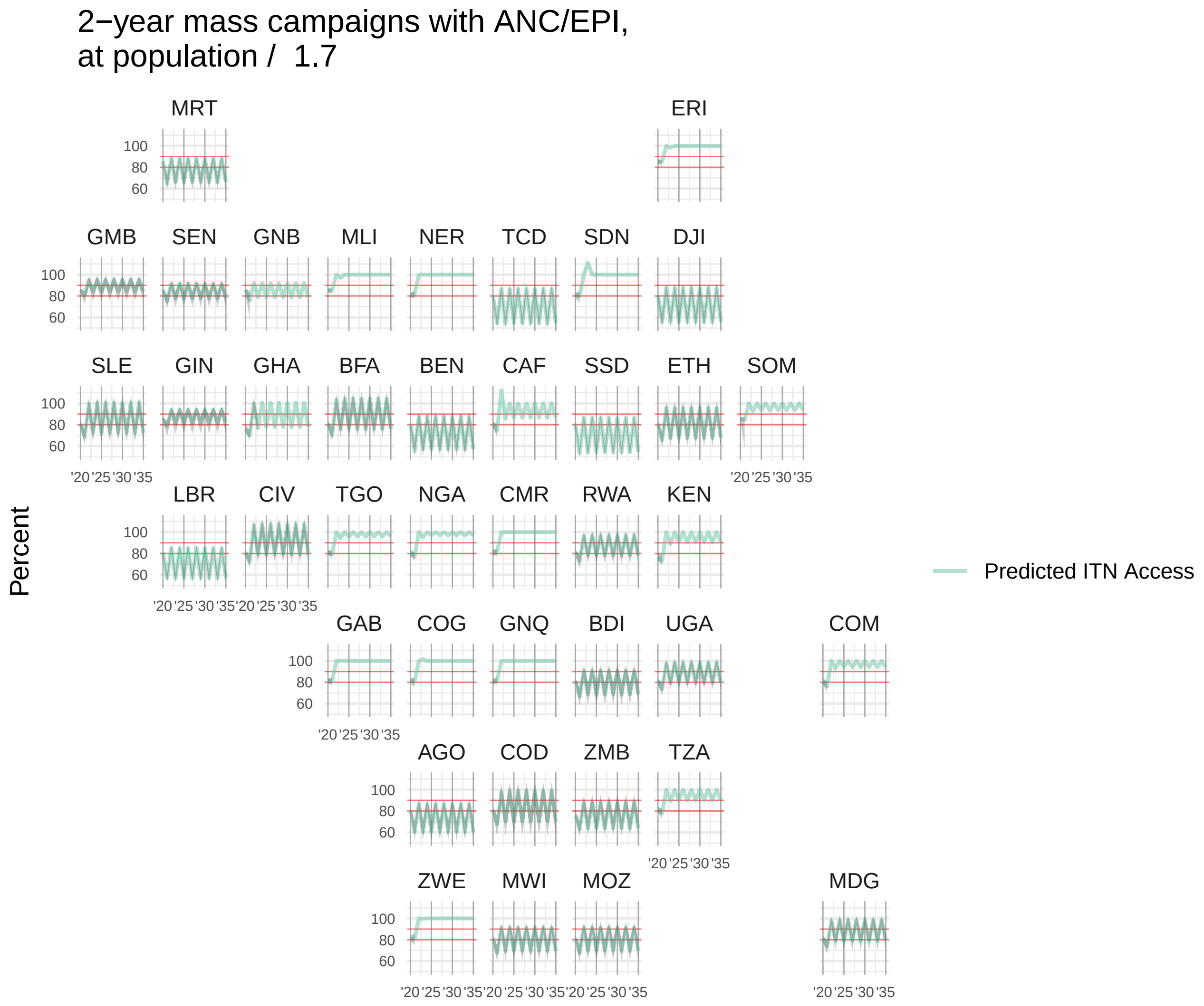

2−year mass campaigns with ANC/EPI,
at population / 1.7
Percent
MRT
ERI
GMB
SEN
GNB
MLI
NER
TCD
SDN
DJI
SLE
GIN
GHA
BFA
BEN
CAF
SSD
ETH
SOM
LBR
CIV
TGO
NGA
CMR
RWA
KEN
GAB
COG
GNQ
BDI
UGA
COM
AGO
COD
ZMB
TZA
ZWE
MWI
MOZ
MDG
100
80
60
'20 '25 '30 '35
Predicted ITN Access

2−year mass campaigns with ANC/EPI, at population / 1.8

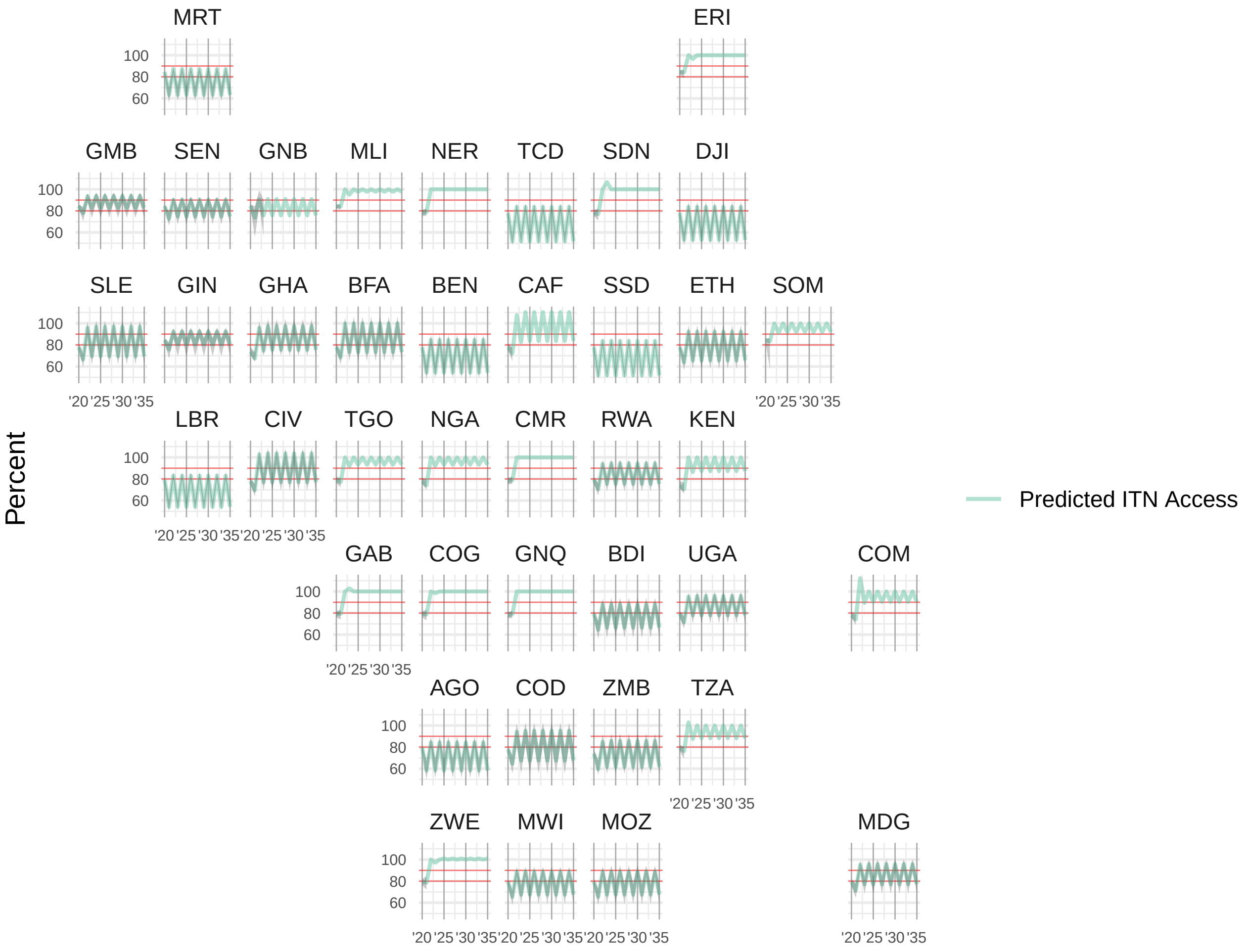

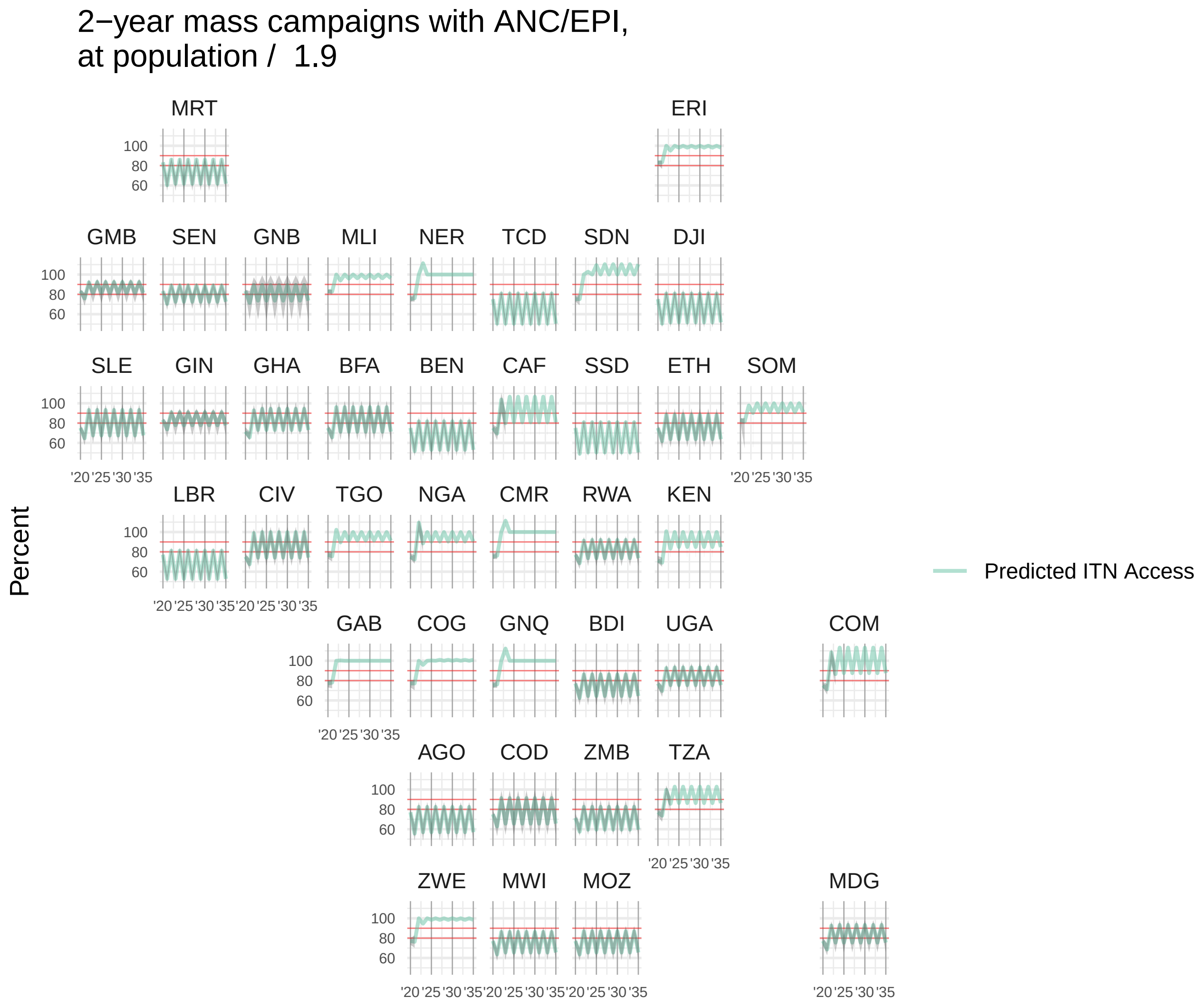
2−year mass campaigns with ANC/EPI,
at population / 1.9
Percent
Predicted ITN Access
MRT
ERI
GMB
SEN
GNB
MLI
NER
TCD
SDN
DJI
SLE
GIN
GHA
BFA
BEN
CAF
SSD
ETH
SOM
LBR
CIV
TGO
NGA
CMR
RWA
KEN
GAB
COG
GNQ
BDI
UGA
COM
AGO
COD
ZMB
TZA
ZWE
MWI
MOZ
MDG
100
80
60
'20 '25 '30 '35

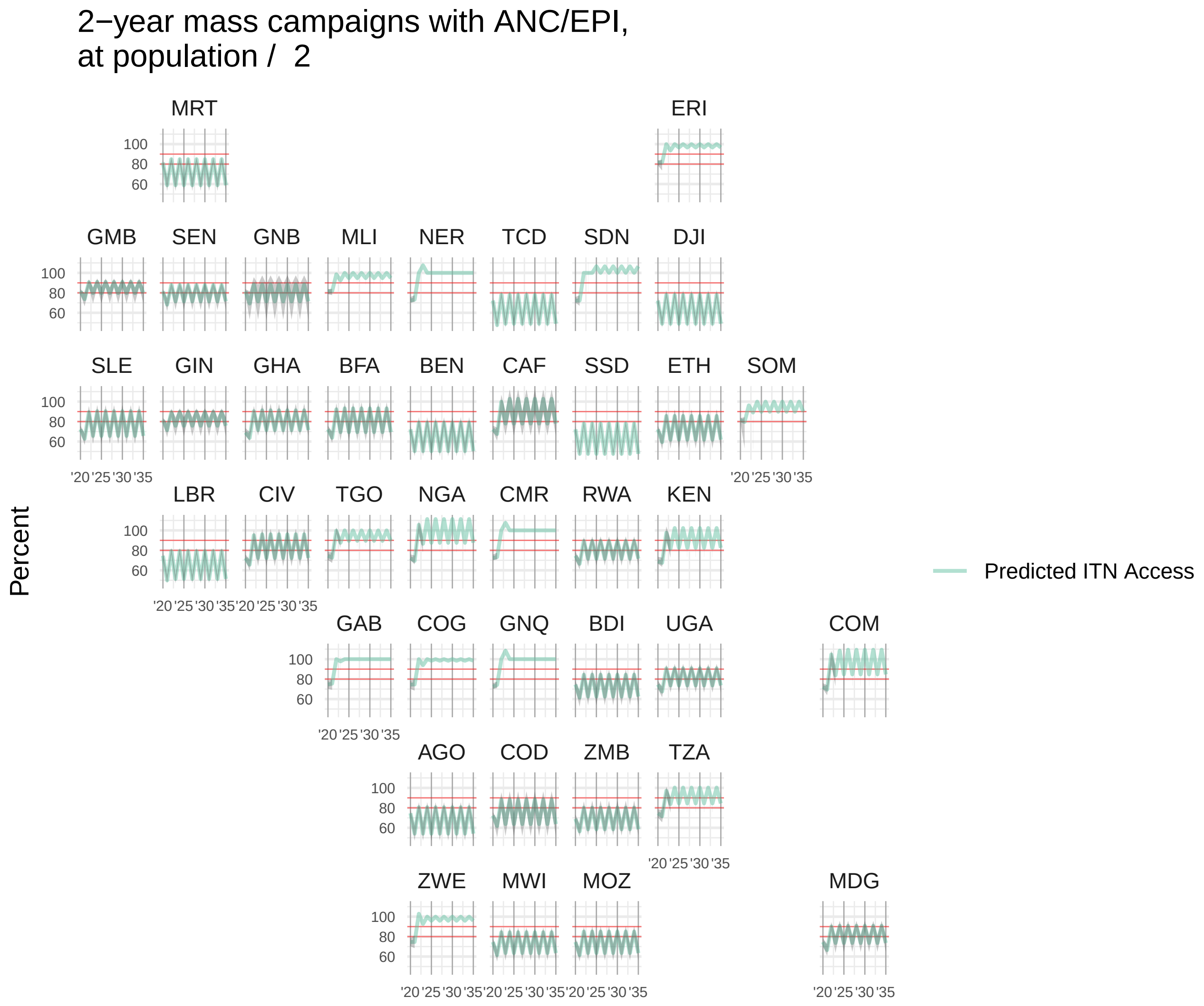
2−year mass campaigns with ANC/EPI,
at population / 2
MRT
ERI
GMB
SEN
GNB
MLI
NER
TCD
SDN
DJI
SLE
GIN
GHA
BFA
BEN
CAF
SSD
ETH
SOM
LBR
CIV
TGO
NGA
CMR
RWA
KEN
GAB
COG
GNQ
BDI
UGA
COM
AGO
COD
ZMB
TZA
ZWE
MWI
MOZ
MDG
100
80
60
'20 '25 '30 '35
Percent
Predicted ITN Access

6 Scenario 6 - Two-year mass campaigns with varying ANC/EPI distribution

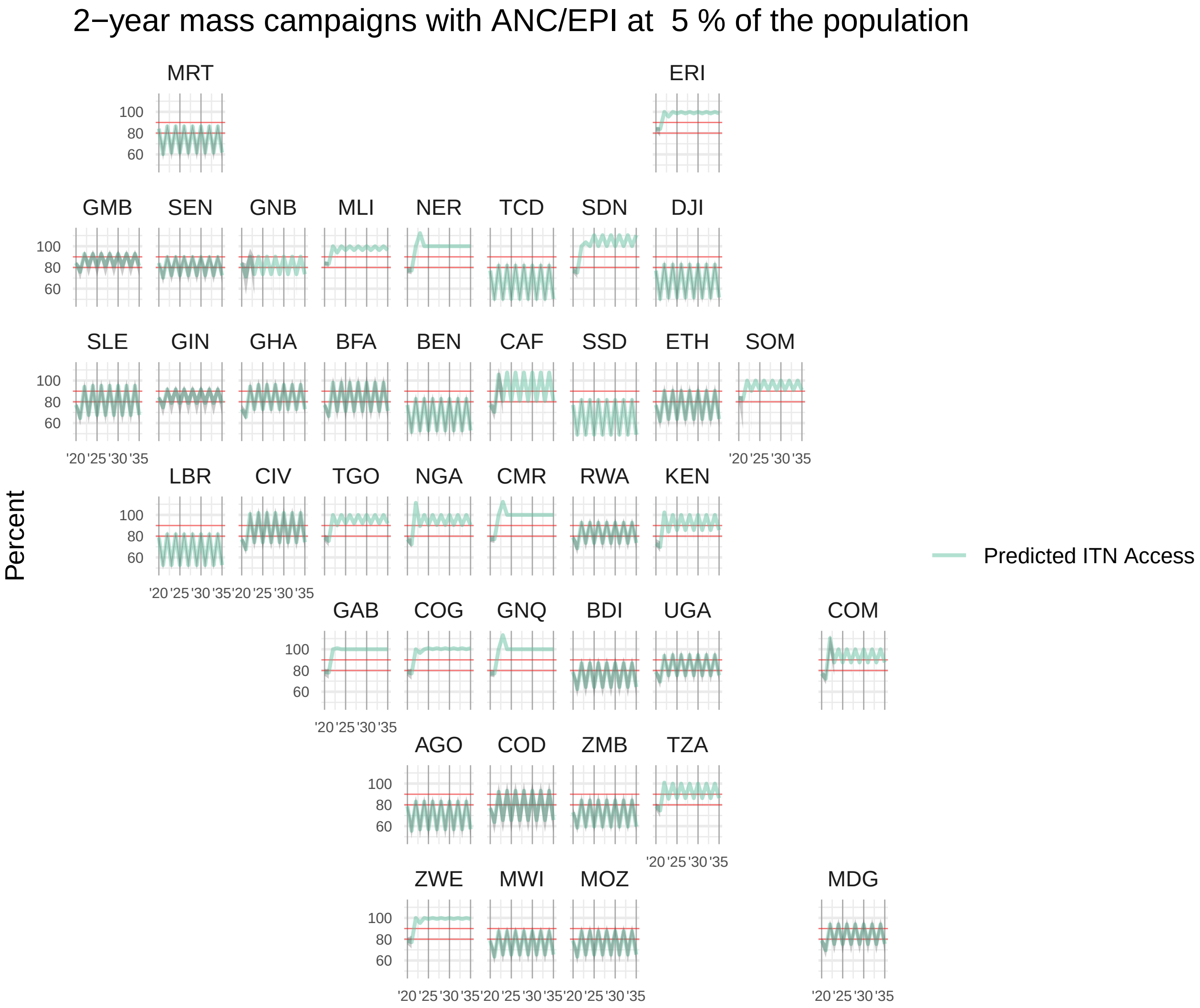

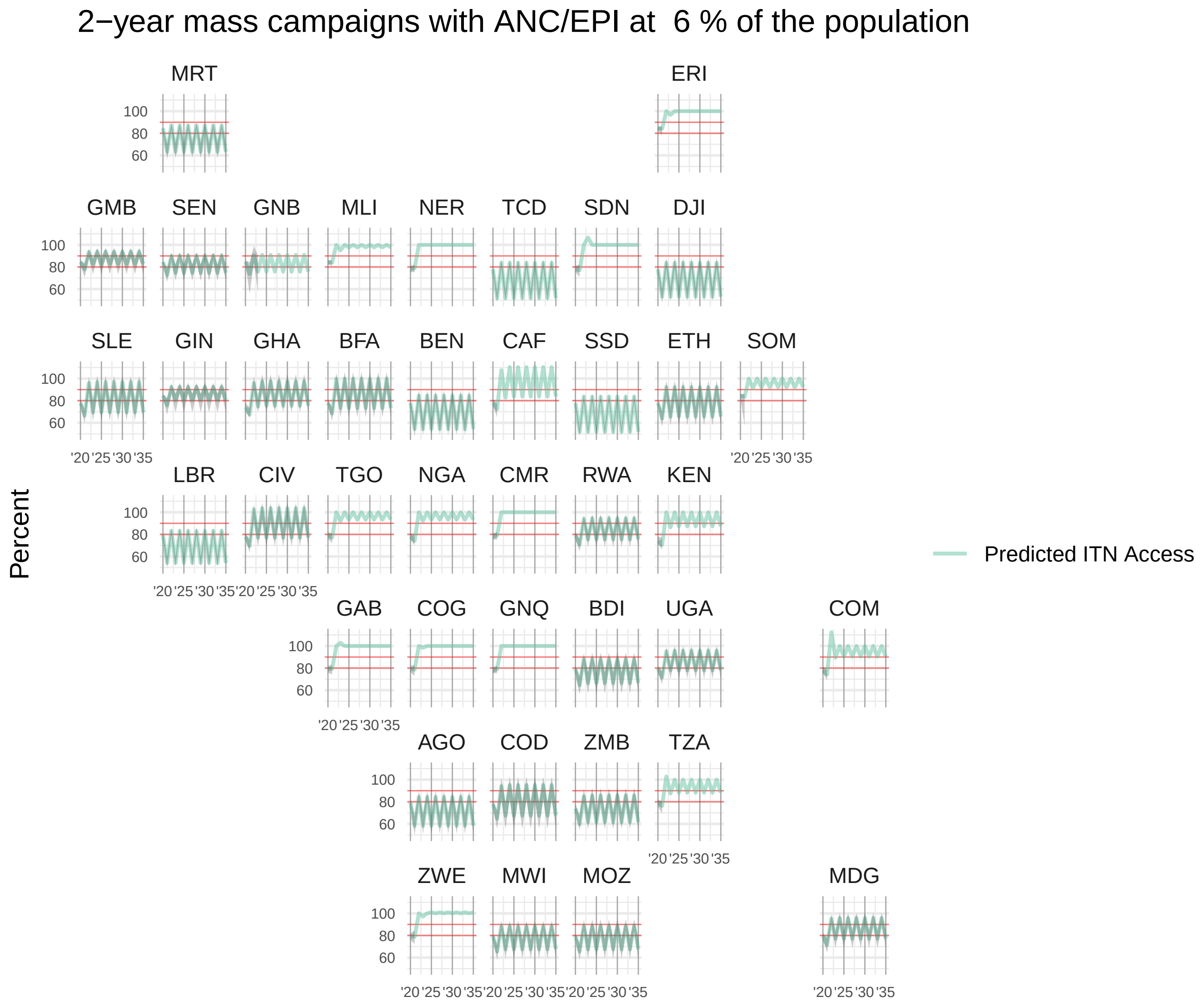

2−year mass campaigns with ANC/EPI at 6 % of the population
MRT
ERI
GMB
SEN
GNB
MLI
NER
TCD
SDN
DJI
SLE
GIN
GHA
BFA
BEN
CAF
SSD
ETH
SOM
LBR
CIV
TGO
NGA
CMR
RWA
KEN
GAB
COG
GNQ
BDI
UGA
COM
AGO
COD
ZMB
TZA
ZWE
MWI
MOZ
MDG
100
80
60
'20 '25 '30 '35
Percent
Predicted ITN Access

2−year mass campaigns with ANC/EPI at 7 % of the population

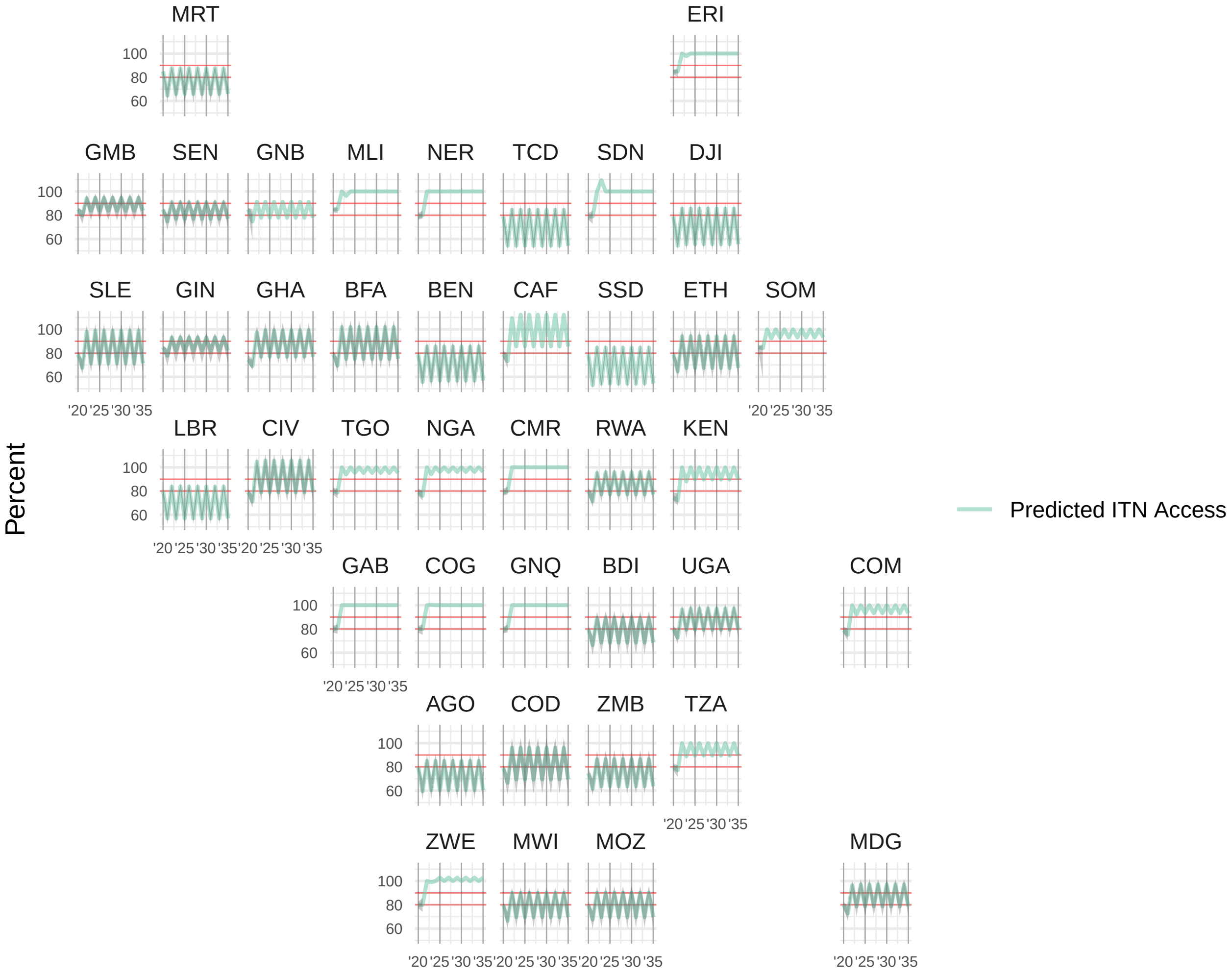

Supplement: Supplementary file 1 — Additional file 1: Estimated ITN access under five ITN distributions scenarios and at varying quantification approaches. [file 12936_2023_4609_MOESM1_ESM.pdf]
