# Supplementary material for: How many mosquito nets are needed to maintain universal coverage: an update
Source: Malar J. 2023 Jun 30;22:200. doi: 10.1186/s12936-023-04609-z (PMC10314435; doi:10.1186/s12936-023-04609-z)
Supplement: Supplementary file 2 — Additional file 2: Table S1. Recommended annual quantifiers for continuous distribution channels. All scenarios assume that ANC and EPI delivery of ITNs is ongoing and provides nets to 6% of the population. However, quantifiers listed in the table represent only the continuous distribution channel, e.g. Angola would require both ANC/EPI distribution as well as continuous distribution quantified using population x 27% to maintain ITN access at levels of 70%. Table S2. Lowest level of ITN access between three-year campaigns at different population quantifiers for all countries. Green color reflects higher ITN access while red indicates lower ITN access reached between campaigns. Routine ITN delivery to pregnant women and infants is assumed. Table S3. Lowest level of ITN access between two-year campaigns at different population quantifiers for all countries. Green color reflects higher ITN access while red indicates lower ITN access reached between campaigns. Values of 100 indicate excess nets in the system. Routine ITN delivery to pregnant women and infants is assumed. Table S4. Summary of recommended quantifiers for all countries and scenarios, to maintain ITN access at or above 80%. The quantifiers are for only the continuous distribution channel or mass campaign; annual ANC/EPI distribution equivalent to 6% of the population is assumed in each scenario, but is not part of the listed quantifiers. Countries are listed by ISO3 code. [file 12936_2023_4609_MOESM2_ESM.docx]

Additional file

Table S1: Recommended annual quantifiers for continuous distribution channels. All scenarios assume that ANC and EPI delivery of ITNs is ongoing and provides nets to 6% of the population. However, quantifiers listed in the table represent only the continuous distribution channel, e.g. Angola would require both ANC/EPI distribution as well as continuous distribution quantified using population x 27% to maintain ITN access at levels of 70%.

| **Minimum quantifier (population x quantifier, annually) to sustain ITN access at or above specified target level** | | | | | | | |
| --- | --- | --- | --- | --- | --- | --- | --- |
|  | | **Scenario 2 (full continuous distribution strategy)** | | | **Scenario 3 (continuous distribution between mass campaigns)** | | |
|  | | **Targeted ITN access level:** | | | | | |
| **Country (ISO3 code)** | **Retention time (years)** | **70%** | **80%** | **90%** | **70%** | **80%** | **90%** |
| DJI | 1.0 | 29% | 37% | 43% | 27% | 35% | 37% |
| LBR | 1.0 | 28% | 36% | 46% | 27% | 35% |  |
| SSD | 1.0 | 30% | 37% | 44% | 28% | 36% | 38% |
| TCD | 1.0 | 30% | 37% | 44% | 28% | 35% | 38% |
| AGO | 1.1 | 27% | 35% | 45% | 25% | 32% | 39% |
| BEN | 1.1 | 29% | 36% | 43% | 27% | 34% | 40% |
| MRT | 1.1 | 23% | 30% | 44% | 21% | 27% | 38% |
| BDI | 1.3 | 25% | 32% | 41% | 19% | 26% | 36% |
| ETH | 1.3 | 26% | 32% | 38% | 20% | 27% | 27% |
| MWI | 1.3 | 24% | 32% | 41% | 19% | 26% | 35% |
| MOZ | 1.3 | 24% | 31% | 40% | 18% | 26% | 35% |
| ZMB | 1.3 | 28% | 35% | 43% | 23% | 30% | 38% |
| COD | 1.4 | 24% | 31% | 37% | 18% | 24% | 25% |
| GNB | 1.4 | 22% | 32% | 39% | 13% | 19% | 32% |
| SEN | 1.4 | 23% | 33% | 39% | 14% | 20% | 33% |
| GIN | 1.5 | 18% | 28% | 36% | 11% | 16% | 29% |
| SLE | 1.5 | 24% | 30% | 36% | 17% | 23% | 24% |
| BFA | 1.6 | 22% | 28% | 34% | 14% | 21% | 39% |
| GMB | 1.6 | 16% | 25% | 35% | 8% | 14% | 27% |
| MDG | 1.6 | 21% | 27% | 35% | 12% | 19% | 27% |
| RWA | 1.6 | 23% | 28% | 36% | 13% | 20% | 28% |
| CIV | 1.7 | 22% | 27% | 32% | 12% | 18% | 38% |
| UGA | 1.7 | 21% | 27% | 35% | 12% | 18% | 27% |
| GHA | 1.8 | 23% | 28% | 32% | 13% | 20% | 28% |
| CAF | 1.9 | 18% | 24% | 29% | 9% | 15% | 34% |
| COM | 2.1 | 15% | 22% | 26% | 6% | 10% | 38% |
| TZA | 2.1 | 14% | 21% | 28% | 4% | 11% | 20% |
| NGA | 2.2 | 14% | 21% | 25% | 4% | 9% | 37% |
| KEN | 2.3 | 16% | 22% | 26% | 6% | 13% | 20% |
| SOM | 2.4 | 10% | 14% | 24% | 0% | 4% | 15% |
| TGO | 2.4 | 12% | 17% | 24% | 1% | 8% | 16% |
| MLI | 2.8 | 8% | 11% | 19% | 0% | 0% | 11% |
| ZWE | 2.8 | 10% | 14% | 21% | 0% | 5% | 12% |
| COG | 2.9 | 10% | 13% | 20% | 0% | 4% | 15% |
| SDN | 2.9 | 10% | 14% | 17% | 0% | 1% | 32% |
| ERI | 3.0 | 7% | 10% | 18% | 0% | 0% | 10% |
| GAB | 3.3 | 8% | 12% | 16% | 0% | 1% | 10% |
| CMR | 3.5 | 8% | 11% | 14% | 0% | 1% | 9% |
| NER | 3.5 | 8% | 11% | 14% | 0% | 1% | 9% |
| GNQ | 3.6 | 8% | 11% | 14% | 0% | 1% | 8% |

Table S2: Lowest level of ITN access between three-year campaigns at different population quantifiers for all countries. Green color reflects higher ITN access while red indicates lower ITN access reached between campaigns. Routine ITN delivery to pregnant women and infants is assumed.

| **Lowest ITN access between three-year campaigns for each quantifier** | | | | | | | | | | | | |
| --- | --- | --- | --- | --- | --- | --- | --- | --- | --- | --- | --- | --- |
|  | | **Population divided by:** | | | | | | | | | | |
| **Cty** | **Retention time (years)** | **1.0** | **1.1** | **1.2** | **1.3** | **1.4** | **1.5** | **1.6** | **1.7** | **1.8** | **1.9** | **2.0** |
| AGO | 1.1 | 27 | 26 | 24 | 24 | 22 | 22 | 22 | 21 | 21 | 21 | 21 |
| BFA | 1.6 | 59 | 56 | 53 | 50 | 48 | 46 | 45 | 42 | 41 | 41 | 39 |
| BDI | 1.3 | 41 | 40 | 37 | 36 | 34 | 34 | 33 | 32 | 32 | 30 | 30 |
| BEN | 1.1 | 23 | 23 | 22 | 22 | 20 | 20 | 20 | 20 | 19 | 19 | 19 |
| COD | 1.4 | 48 | 45 | 42 | 41 | 38 | 37 | 37 | 35 | 34 | 33 | 33 |
| CAF | 1.9 | 74 | 70 | 68 | 65 | 63 | 61 | 58 | 56 | 54 | 52 | 50 |
| COG | 2.9 | 96 | 92 | 88 | 86 | 83 | 81 | 79 | 78 | 75 | 74 | 73 |
| CIV | 1.7 | 65 | 62 | 59 | 56 | 53 | 52 | 49 | 48 | 46 | 45 | 44 |
| CMR | 3.5 | 100 | 100 | 99 | 95 | 90 | 88 | 85 | 82 | 79 | 77 | 75 |
| DJI | 1.0 | 22 | 22 | 20 | 20 | 20 | 19 | 19 | 19 | 19 | 19 | 17 |
| ERI | 3.0 | 95 | 92 | 91 | 89 | 88 | 86 | 85 | 84 | 83 | 82 | 81 |
| ETH | 1.3 | 41 | 39 | 37 | 35 | 34 | 33 | 33 | 31 | 30 | 30 | 28 |
| GAB | 3.3 | 100 | 96 | 93 | 90 | 87 | 84 | 82 | 81 | 79 | 78 | 76 |
| GHA | 1.8 | 67 | 63 | 60 | 57 | 55 | 53 | 51 | 50 | 48 | 46 | 45 |
| GMB | 1.6 | 70 | 67 | 65 | 62 | 59 | 56 | 54 | 53 | 51 | 50 | 48 |
| GIN | 1.5 | 63 | 60 | 57 | 54 | 53 | 50 | 48 | 47 | 45 | 44 | 44 |
| GNQ | 3.6 | 100 | 100 | 100 | 96 | 91 | 88 | 86 | 83 | 80 | 78 | 76 |
| GNB | 1.4 | 53 | 50 | 48 | 45 | 44 | 42 | 41 | 41 | 39 | 38 | 38 |
| KEN | 2.3 | 81 | 77 | 74 | 71 | 69 | 67 | 65 | 63 | 61 | 58 | 57 |
| COM | 2.1 | 83 | 78 | 75 | 71 | 68 | 67 | 65 | 63 | 61 | 59 | 57 |
| LBR | 1.0 | 22 | 21 | 21 | 19 | 19 | 19 | 19 | 18 | 18 | 18 | 18 |
| MDG | 1.6 | 65 | 61 | 59 | 57 | 54 | 51 | 50 | 49 | 47 | 46 | 44 |
| MLI | 2.8 | 94 | 91 | 89 | 88 | 86 | 85 | 84 | 83 | 82 | 80 | 78 |
| MRT | 1.1 | 29 | 29 | 27 | 27 | 26 | 26 | 26 | 26 | 24 | 24 | 24 |
| MWI | 1.3 | 43 | 41 | 39 | 37 | 36 | 34 | 34 | 33 | 32 | 32 | 30 |
| MOZ | 1.3 | 44 | 41 | 40 | 39 | 37 | 36 | 34 | 33 | 33 | 32 | 32 |
| NER | 3.5 | 100 | 100 | 99 | 95 | 90 | 88 | 85 | 82 | 79 | 77 | 75 |
| NGA | 2.2 | 86 | 81 | 77 | 74 | 71 | 68 | 67 | 65 | 63 | 62 | 59 |
| RWA | 1.6 | 61 | 59 | 56 | 53 | 51 | 49 | 47 | 46 | 44 | 43 | 41 |
| SDN | 2.9 | 100 | 97 | 91 | 88 | 84 | 81 | 78 | 76 | 73 | 71 | 70 |
| SLE | 1.5 | 52 | 49 | 46 | 44 | 42 | 41 | 39 | 38 | 37 | 35 | 35 |
| SEN | 1.4 | 51 | 48 | 45 | 44 | 42 | 41 | 39 | 39 | 38 | 37 | 37 |
| SOM | 2.4 | 89 | 87 | 86 | 84 | 83 | 81 | 79 | 77 | 75 | 73 | 71 |
| SSD | 1.0 | 20 | 20 | 19 | 19 | 19 | 19 | 17 | 17 | 17 | 17 | 17 |
| TCD | 1.0 | 22 | 20 | 20 | 19 | 19 | 19 | 19 | 17 | 17 | 17 | 17 |
| TGO | 2.4 | 88 | 85 | 82 | 80 | 77 | 75 | 73 | 71 | 69 | 67 | 65 |
| TZA | 2.1 | 83 | 80 | 77 | 75 | 72 | 69 | 67 | 64 | 63 | 61 | 60 |
| UGA | 1.7 | 65 | 62 | 59 | 57 | 54 | 53 | 50 | 49 | 47 | 46 | 44 |
| ZMB | 1.3 | 40 | 37 | 36 | 34 | 33 | 32 | 30 | 30 | 29 | 28 | 28 |
| ZWE | 2.8 | 94 | 90 | 87 | 84 | 82 | 80 | 78 | 76 | 75 | 73 | 71 |

Table S3: Lowest level of ITN access between two-year campaigns at different population quantifiers for all countries. Green color reflects higher ITN access while red indicates lower ITN access reached between campaigns. Values of 100 indicate excess nets in the system (for example Cameroon). Routine ITN delivery to pregnant women and infants is assumed.

| **Lowest ITN access between two-year campaigns for each quantifier** | | | | | | | | | | | | |
| --- | --- | --- | --- | --- | --- | --- | --- | --- | --- | --- | --- | --- |
|  | | **Population divided by:** | | | | | | | | | | |
| **Cty** | **Retention time (years)** | **1.0** | **1.1** | **1.2** | **1.3** | **1.4** | **1.5** | **1.6** | **1.7** | **1.8** | **1.9** | **2.0** |
| AGO | 1.1 | 81 | 78 | 75 | 71 | 68 | 64 | 62 | 60 | 58 | 57 | 54 |
| BFA | 1.6 | 100 | 100 | 94 | 89 | 86 | 82 | 79 | 76 | 73 | 71 | 69 |
| BDI | 1.3 | 90 | 85 | 82 | 79 | 77 | 74 | 72 | 69 | 67 | 64 | 62 |
| BEN | 1.1 | 79 | 74 | 70 | 68 | 65 | 62 | 59 | 57 | 54 | 53 | 50 |
| COD | 1.4 | 98 | 92 | 87 | 83 | 79 | 75 | 72 | 70 | 68 | 66 | 64 |
| CAF | 1.9 | 100 | 100 | 100 | 100 | 97 | 93 | 89 | 86 | 83 | 80 | 78 |
| COG | 2.9 | 100 | 100 | 100 | 100 | 100 | 100 | 100 | 100 | 98 | 96 | 94 |
| CIV | 1.7 | 100 | 100 | 99 | 94 | 89 | 86 | 82 | 79 | 77 | 74 | 72 |
| CMR | 3.5 | 100 | 100 | 100 | 100 | 100 | 100 | 100 | 100 | 100 | 100 | 100 |
| DJI | 1.0 | 77 | 73 | 69 | 66 | 64 | 61 | 58 | 56 | 53 | 52 | 49 |
| ERI | 3.0 | 100 | 100 | 100 | 100 | 100 | 100 | 100 | 98 | 97 | 95 | 94 |
| ETH | 1.3 | 94 | 88 | 83 | 79 | 75 | 72 | 69 | 68 | 66 | 64 | 62 |
| GAB | 3.3 | 100 | 100 | 100 | 100 | 100 | 100 | 100 | 100 | 100 | 100 | 98 |
| GHA | 1.8 | 100 | 100 | 94 | 90 | 86 | 83 | 80 | 77 | 75 | 73 | 71 |
| GMB | 1.6 | 97 | 94 | 92 | 90 | 88 | 86 | 85 | 84 | 83 | 82 | 80 |
| GIN | 1.5 | 95 | 92 | 90 | 88 | 86 | 85 | 84 | 82 | 81 | 78 | 76 |
| GNQ | 3.6 | 100 | 100 | 100 | 100 | 100 | 100 | 100 | 100 | 100 | 100 | 100 |
| GNB | 1.4 | 92 | 89 | 88 | 86 | 84 | 83 | 81 | 79 | 76 | 74 | 71 |
| KEN | 2.3 | 100 | 100 | 100 | 100 | 100 | 96 | 92 | 89 | 86 | 83 | 82 |
| COM | 2.1 | 100 | 100 | 100 | 100 | 100 | 100 | 96 | 93 | 89 | 87 | 84 |
| LBR | 1.0 | 78 | 74 | 71 | 67 | 64 | 61 | 59 | 57 | 54 | 53 | 51 |
| MDG | 1.6 | 100 | 97 | 92 | 89 | 86 | 84 | 81 | 79 | 77 | 75 | 74 |
| MLI | 2.8 | 100 | 100 | 100 | 100 | 100 | 100 | 99 | 97 | 95 | 94 | 92 |
| MRT | 1.1 | 85 | 83 | 80 | 77 | 74 | 70 | 68 | 66 | 63 | 62 | 59 |
| MWI | 1.3 | 90 | 86 | 82 | 80 | 77 | 75 | 72 | 70 | 68 | 65 | 63 |
| MOZ | 1.3 | 90 | 86 | 83 | 81 | 78 | 75 | 73 | 70 | 68 | 65 | 63 |
| NER | 3.5 | 100 | 100 | 100 | 100 | 100 | 100 | 100 | 100 | 100 | 100 | 100 |
| NGA | 2.2 | 100 | 100 | 100 | 100 | 100 | 100 | 99 | 95 | 92 | 88 | 87 |
| RWA | 1.6 | 99 | 95 | 91 | 87 | 84 | 82 | 80 | 78 | 75 | 74 | 72 |
| SDN | 2.9 | 100 | 100 | 100 | 100 | 100 | 100 | 100 | 100 | 100 | 100 | 100 |
| SLE | 1.5 | 100 | 95 | 89 | 85 | 81 | 78 | 74 | 72 | 69 | 68 | 66 |
| SEN | 1.4 | 91 | 89 | 87 | 85 | 84 | 82 | 80 | 78 | 75 | 73 | 71 |
| SOM | 2.4 | 100 | 100 | 100 | 100 | 98 | 96 | 94 | 93 | 91 | 90 | 89 |
| SSD | 1.0 | 75 | 71 | 68 | 65 | 62 | 59 | 57 | 54 | 52 | 50 | 48 |
| TCD | 1.0 | 76 | 71 | 68 | 65 | 63 | 59 | 57 | 54 | 52 | 50 | 49 |
| TGO | 2.4 | 100 | 100 | 100 | 100 | 100 | 100 | 97 | 95 | 92 | 90 | 88 |
| TZA | 2.1 | 100 | 100 | 100 | 100 | 98 | 95 | 92 | 90 | 88 | 86 | 84 |
| UGA | 1.7 | 100 | 97 | 93 | 89 | 86 | 84 | 81 | 79 | 78 | 75 | 74 |
| ZMB | 1.3 | 86 | 82 | 78 | 75 | 71 | 69 | 67 | 64 | 62 | 60 | 58 |
| ZWE | 2.8 | 100 | 100 | 100 | 100 | 100 | 100 | 100 | 100 | 97 | 95 | 93 |

Table S4: Summary of recommended quantifiers for all countries and scenarios, to maintain ITN access at or above 80%. The quantifiers are for only the continuous distribution channel or mass campaign; annual ANC/EPI distribution equivalent to 6% of the population is assumed in each scenario, but is not part of the listed quantifiers. Countries are listed by ISO3 code.

|  | | **Continuous Distribution ITNs = Population x X, annually** | | **Mass Campaign ITNs = Population / X** | |
| --- | --- | --- | --- | --- | --- |
| **Country** | **Retention time (years)** | **Scenario 2: Full-scale continuous + routine** | **Scenario 3: Campaign + routine + continuous between campaigns** | **Scenario 4: Three-yearly campaigns** | **Scenario 5: Two-yearly campaigns** |
| AGO | 1.1 | 35% | 32% | 0.1 | 1.0 |
| BDI | 1.3 | 32% | 26% | 0.3 | 1.2 |
| BEN | 1.1 | 36% | 34% | 0.1 | 0.9 |
| BFA | 1.6 | 28% | 21% | 0.5 | 1.5 |
| CAF | 1.9 | 24% | 15% | 0.8 | 1.8 |
| CIV | 1.7 | 27% | 18% | 0.6 | 1.6 |
| CMR | 3.5 | 11% | 1% | 1.7 | 2.0 |
| COD | 1.4 | 31% | 24% | 0.4 | 1.3 |
| COG | 2.9 | 13% | 4% | 1.5 | 2.0 |
| COM | 2.1 | 22% | 10% | 1.0 | 2.0 |
| DJI | 1.0 | 37% | 35% | 0.1 | 0.9 |
| ERI | 3.0 | 10% | 0% | 2.0 | 2.0 |
| ETH | 1.3 | 32% | 27% | 0.3 | 1.2 |
| GAB | 3.3 | 12% | 1% | 1.7 | 2.0 |
| GHA | 1.8 | 28% | 20% | 0.6 | 1.6 |
| GIN | 1.5 | 28% | 16% | 0.6 | 1.8 |
| GMB | 1.6 | 25% | 14% | 0.7 | 1.9 |
| GNB | 1.4 | 32% | 19% | 0.4 | 1.6 |
| GNQ | 3.6 | 11% | 1% | 1.7 | 2.0 |
| KEN | 2.3 | 22% | 13% | 1.0 | 2.0 |
| LBR | 1.0 | 36% | 35% |  | 0.9 |
| MDG | 1.6 | 27% | 19% | 0.6 | 1.6 |
| MLI | 2.8 | 11% | 0% | 1.8 | 2.0 |
| MOZ | 1.3 | 31% | 26% | 0.3 | 1.3 |
| MRT | 1.1 | 30% | 27% | 0.1 | 1.1 |
| MWI | 1.3 | 32% | 26% | 0.3 | 1.2 |
| NER | 3.5 | 11% | 1% | 1.7 | 2.0 |
| NGA | 2.2 | 21% | 9% | 1.1 | 2.0 |
| RWA | 1.6 | 28% | 20% | 0.6 | 1.5 |
| SDN | 2.9 | 14% | 1% | 1.5 | 2.0 |
| SEN | 1.4 | 33% | 20% | 0.4 | 1.5 |
| SLE | 1.5 | 30% | 23% | 0.4 | 1.4 |
| SOM | 2.4 | 14% | 4% | 1.5 | 2.0 |
| SSD | 1.0 | 37% | 36% |  | 0.9 |
| TCD | 1.0 | 37% | 35% |  | 0.9 |
| TGO | 2.4 | 17% | 8% | 1.2 | 2.0 |
| TZA | 2.1 | 21% | 11% | 1.0 | 2.0 |
| UGA | 1.7 | 27% | 18% | 0.6 | 1.6 |
| ZMB | 1.3 | 35% | 30% | 0.3 | 1.1 |
| ZWE | 2.8 | 14% | 5% | 1.4 | 2.0 |
