# Supplementary material for: How many mosquito nets are needed to maintain universal coverage: an update
Source: Malar J. 2023 Jun 30;22:200. doi: 10.1186/s12936-023-04609-z (PMC10314435; doi:10.1186/s12936-023-04609-z)
Supplement: Supplementary file 3 — Additional file 3: Frontier plots of Person-years of ITN access vs Total nets delivered. [file 12936_2023_4609_MOESM3_ESM.pdf]

Additional File 3: Frontier plots for each country showing person-years of ITN access vs total nets required

Hannah Koenker

13 June, 2023

Contents

Frontier Plots (alpha order by ISO3 country code) 1

Frontier Plots (alpha order by ISO3 country code)

Note: all countries use an illustrative population of 10,000,000 people.

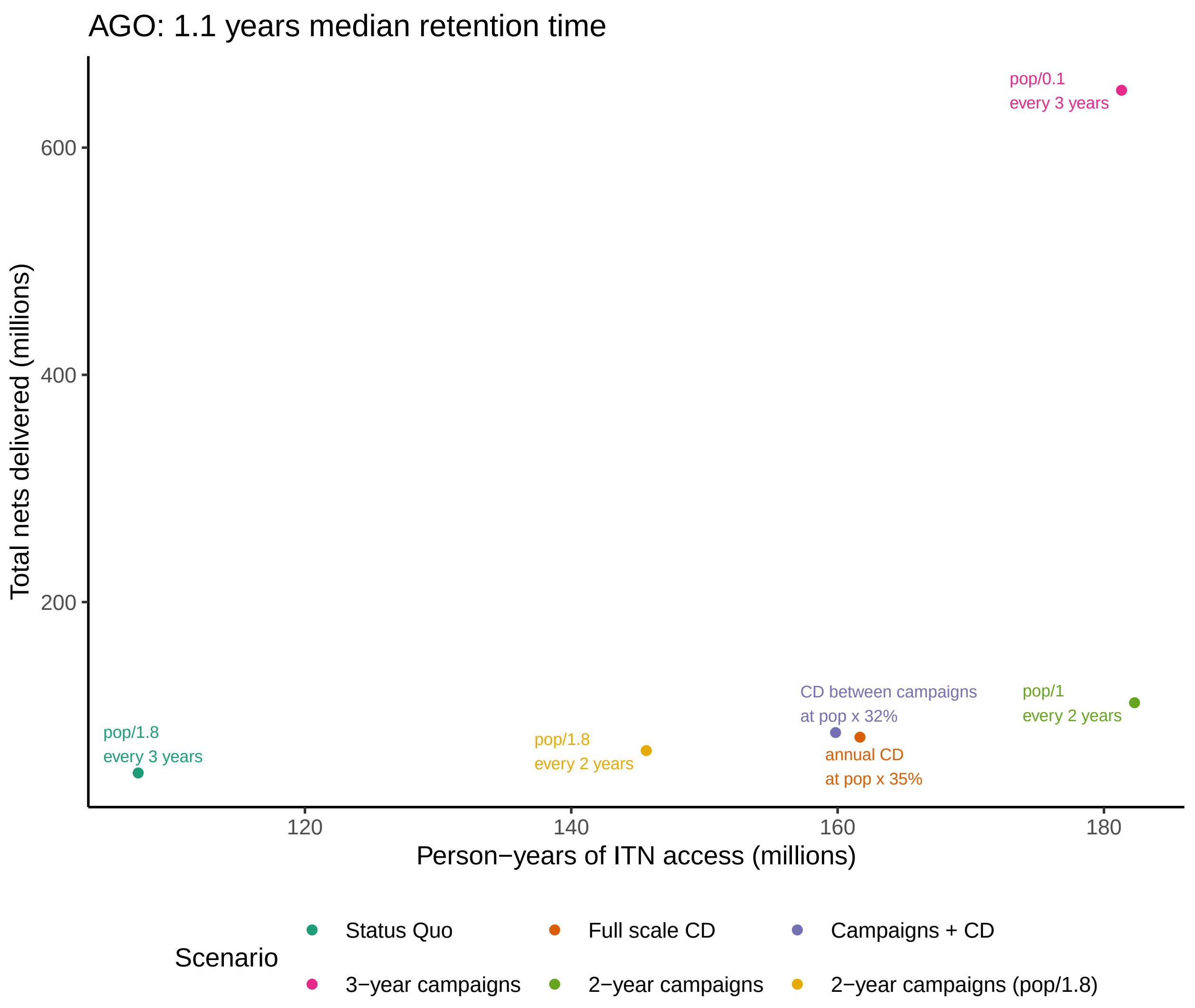
AGO: 1.1 years median retention time
Total nets delivered (millions)
600
400
200
pop/0.1
every 3 years
CD between campaigns
at pop x 32%
pop/1
every 2 years
pop/1.8
every 3 years
pop/1.8
every 2 years
annual CD
at pop x 35%
120
140
160
180
Person−years of ITN access (millions)
Scenario
Status Quo
Full scale CD
Campaigns + CD
3−year campaigns
2−year campaigns
2−year campaigns (pop/1.8)

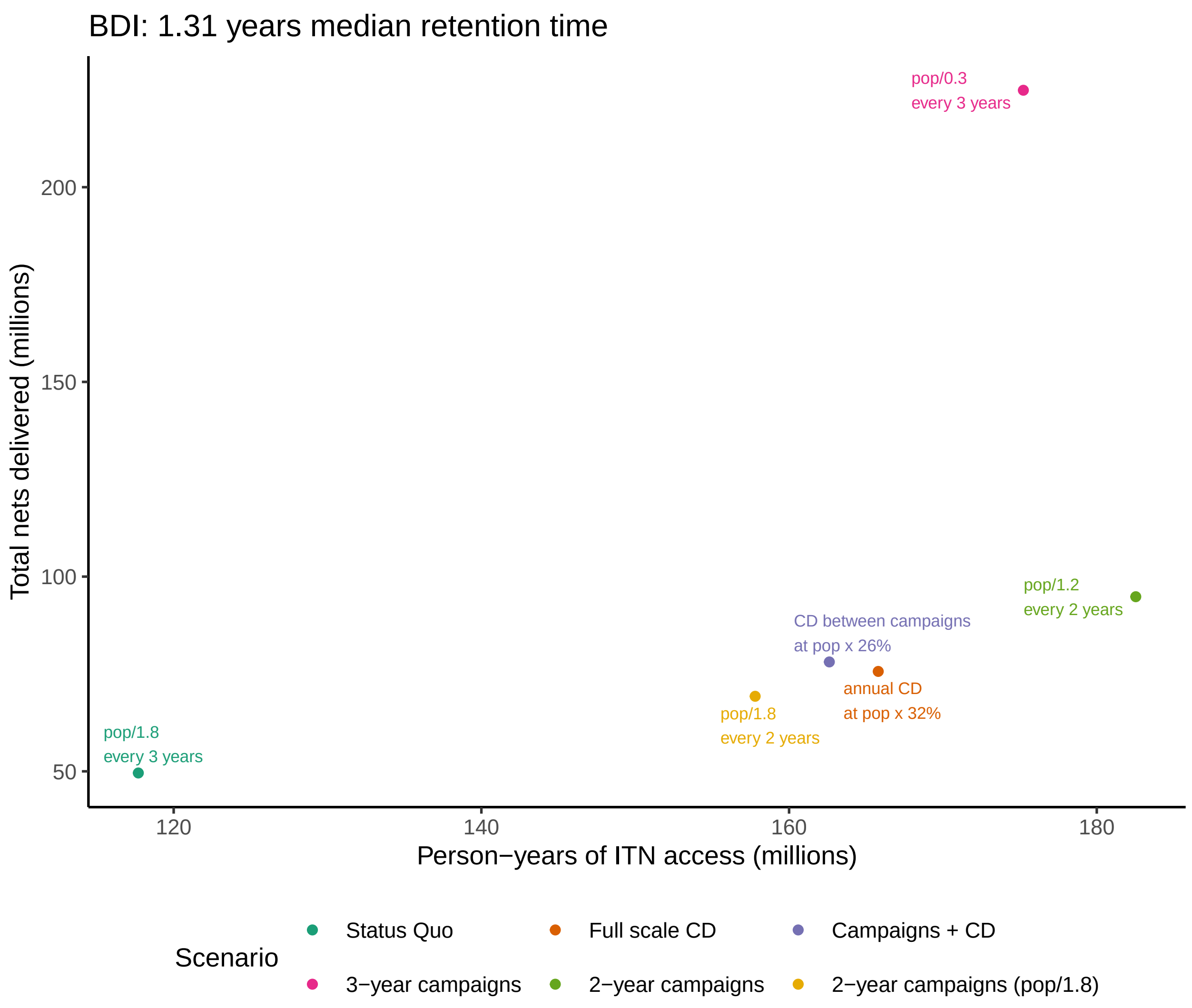

BDI: 1.31 years median retention time
Total nets delivered (millions)
200
150
100
50
120
140
160
180
Person−years of ITN access (millions)
pop/0.3
every 3 years
pop/1.2
every 2 years
CD between campaigns
at pop x 26%
annual CD
at pop x 32%
pop/1.8
every 2 years
pop/1.8
every 3 years
Scenario
Status Quo
Full scale CD
Campaigns + CD
3−year campaigns
2−year campaigns
2−year campaigns (pop/1.8)

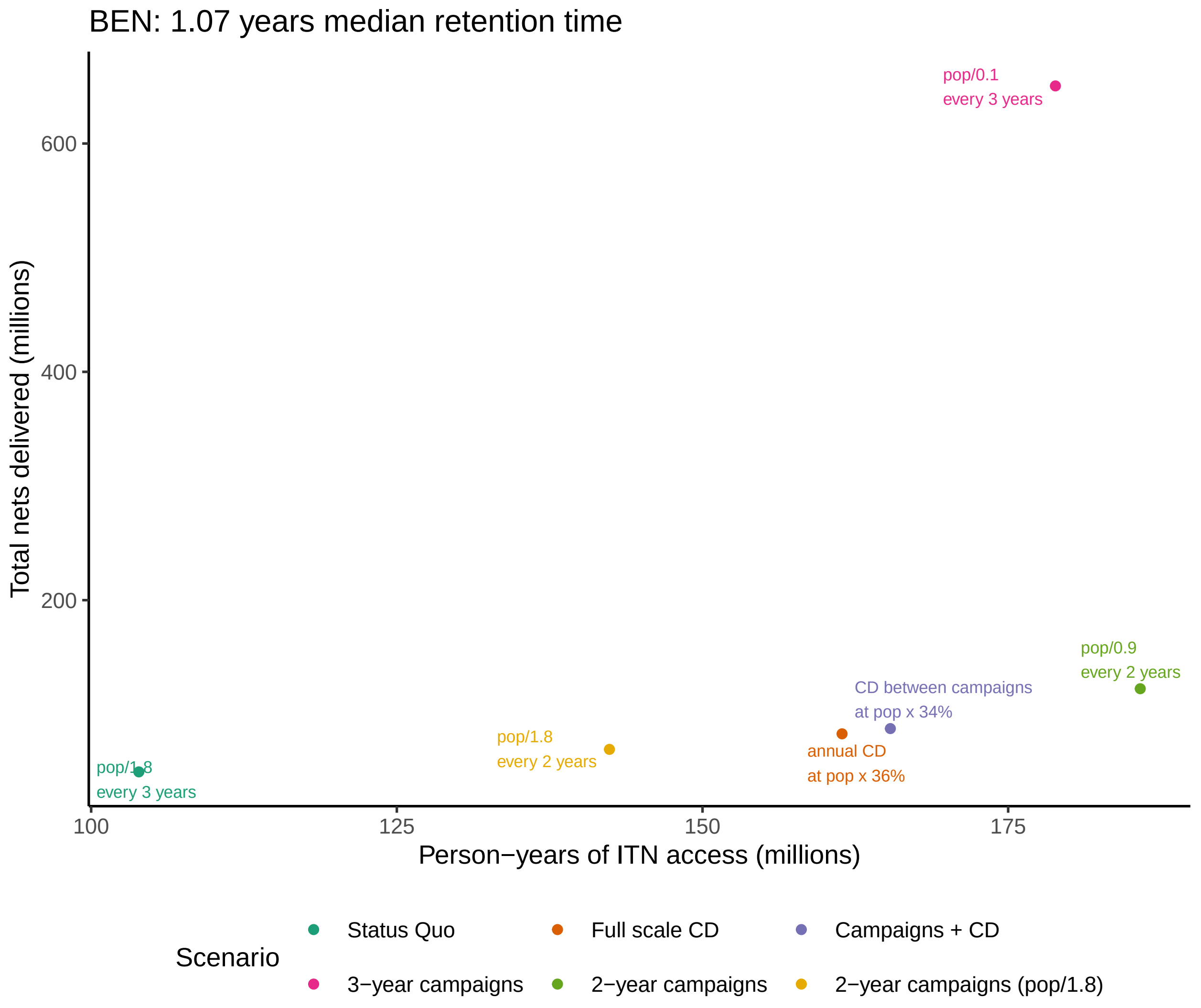
BEN: 1.07 years median retention time
Total nets delivered (millions)
600
400
200
100
125
150
175
Person−years of ITN access (millions)
pop/0.1
every 3 years
pop/0.9
every 2 years
CD between campaigns
at pop x 34%
annual CD
at pop x 36%
pop/1.8
every 2 years
pop/1.8
every 3 years
Scenario
Status Quo
Full scale CD
Campaigns + CD
3−year campaigns
2−year campaigns
2−year campaigns (pop/1.8)

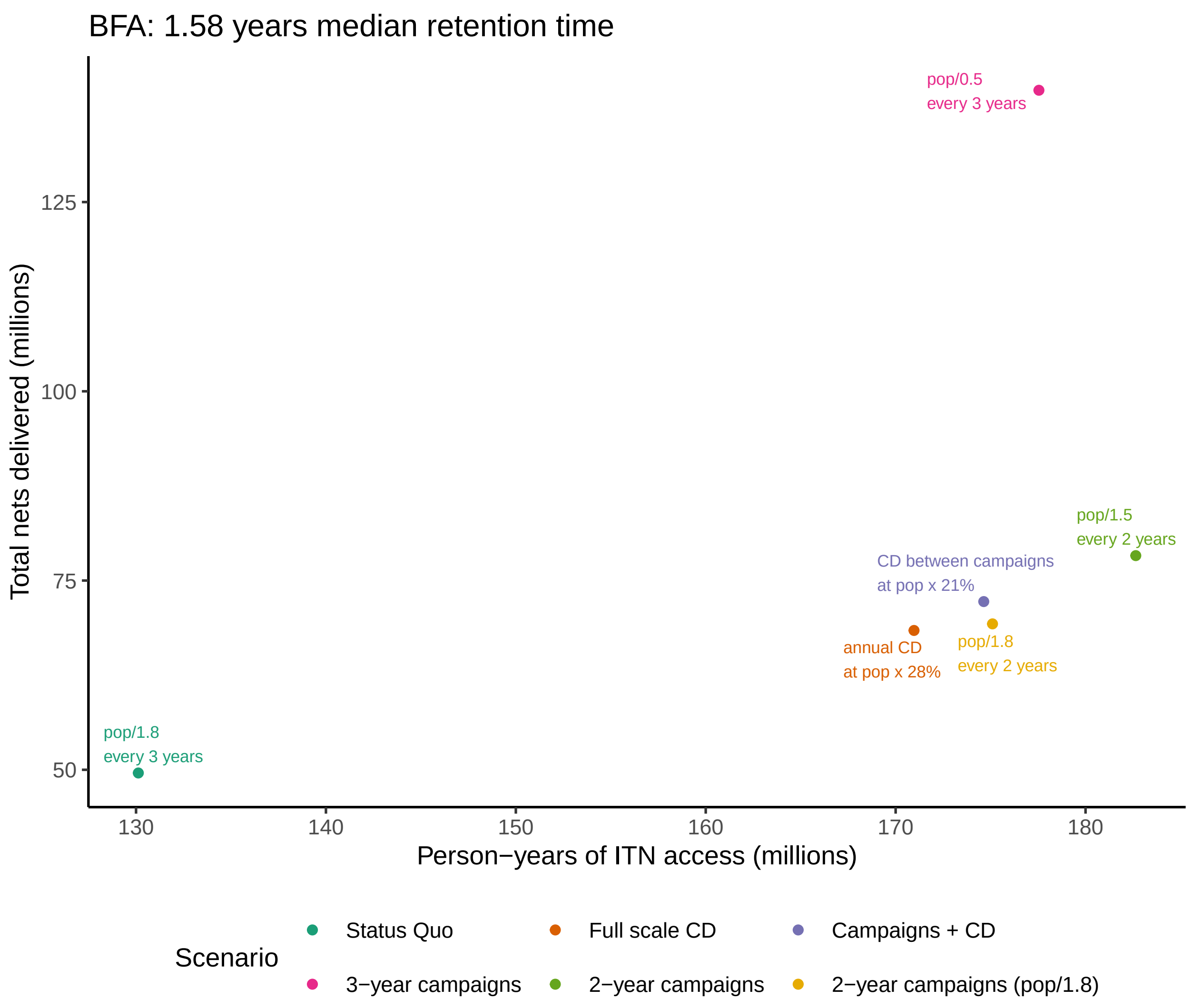

BFA: 1.58 years median retention time
pop/0.5
every 3 years
pop/1.5
every 2 years
CD between campaigns
at pop x 21%
annual CD
at pop x 28%
pop/1.8
every 2 years
pop/1.8
every 3 years
125
100
75
50
Total nets delivered (millions)
130
140
150
160
170
180
Person−years of ITN access (millions)
Scenario
Status Quo
Full scale CD
Campaigns + CD
3−year campaigns
2−year campaigns
2−year campaigns (pop/1.8)

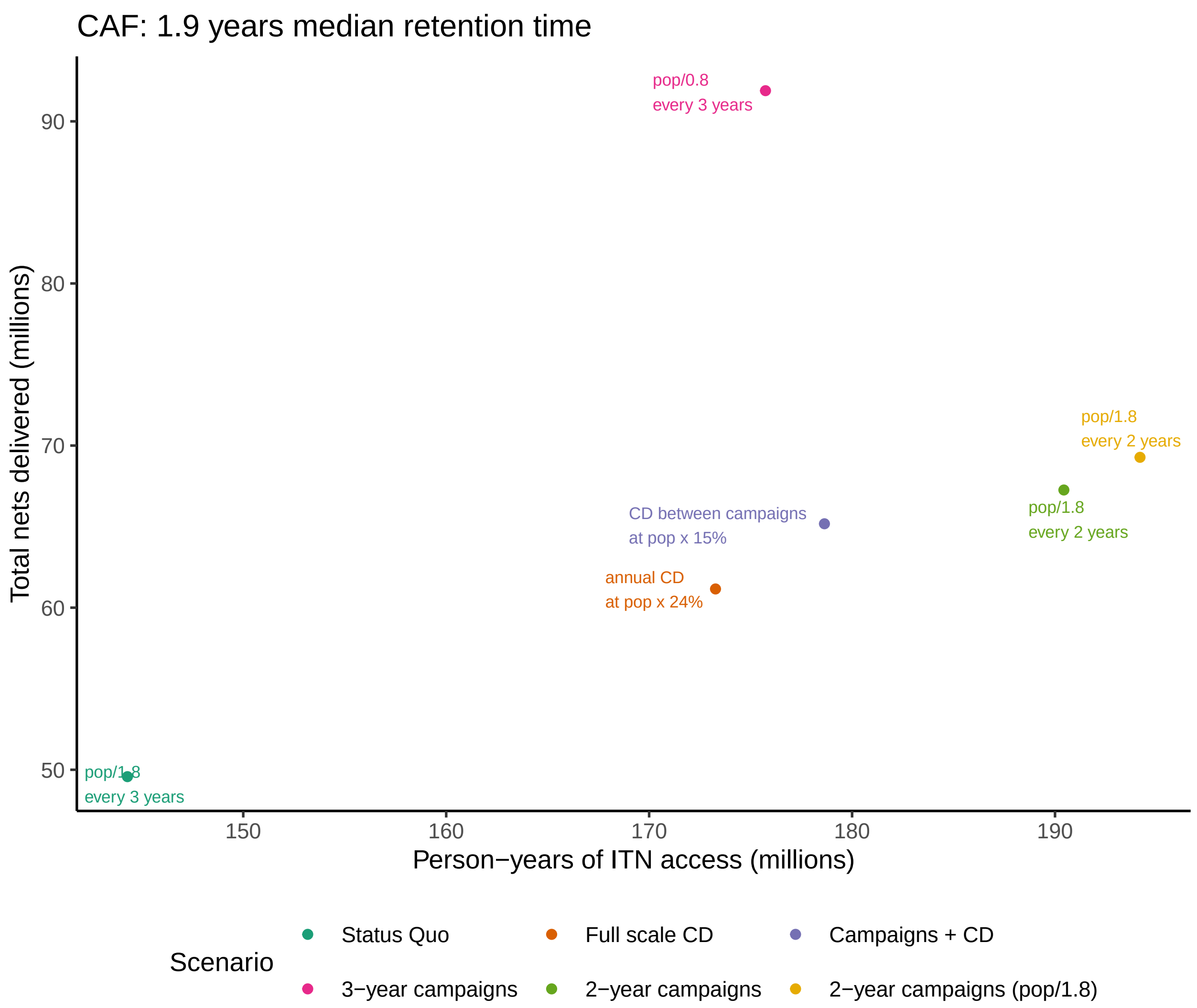

CAF: 1.9 years median retention time
Total nets delivered (millions)
Person−years of ITN access (millions)
90
80
70
60
50
150
160
170
180
190
pop/0.8
every 3 years
pop/1.8
every 2 years
CD between campaigns
at pop x 15%
pop/1.8
every 2 years
annual CD
at pop x 24%
pop/1.8
every 3 years
Scenario
Status Quo
Full scale CD
Campaigns + CD
3−year campaigns
2−year campaigns
2−year campaigns (pop/1.8)

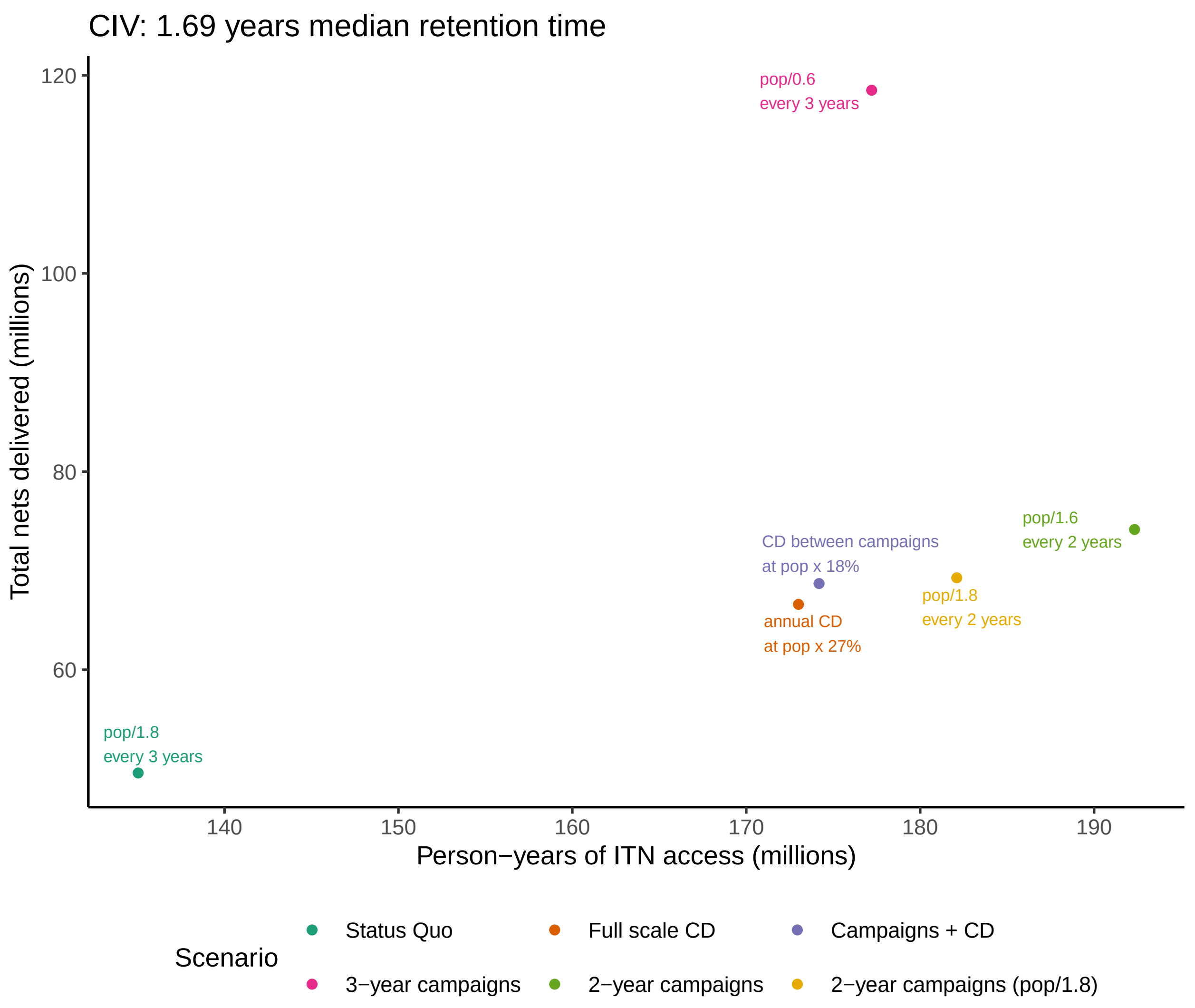
CIV: 1.69 years median retention time
Total nets delivered (millions)
120
100
80
60
pop/0.6
every 3 years
CD between campaigns
at pop x 18%
annual CD
at pop x 27%
pop/1.6
every 2 years
pop/1.8
every 2 years
pop/1.8
every 3 years
140
150
160
170
180
190
Person−years of ITN access (millions)
Scenario
Status Quo
Full scale CD
Campaigns + CD
3−year campaigns
2−year campaigns
2−year campaigns (pop/1.8)

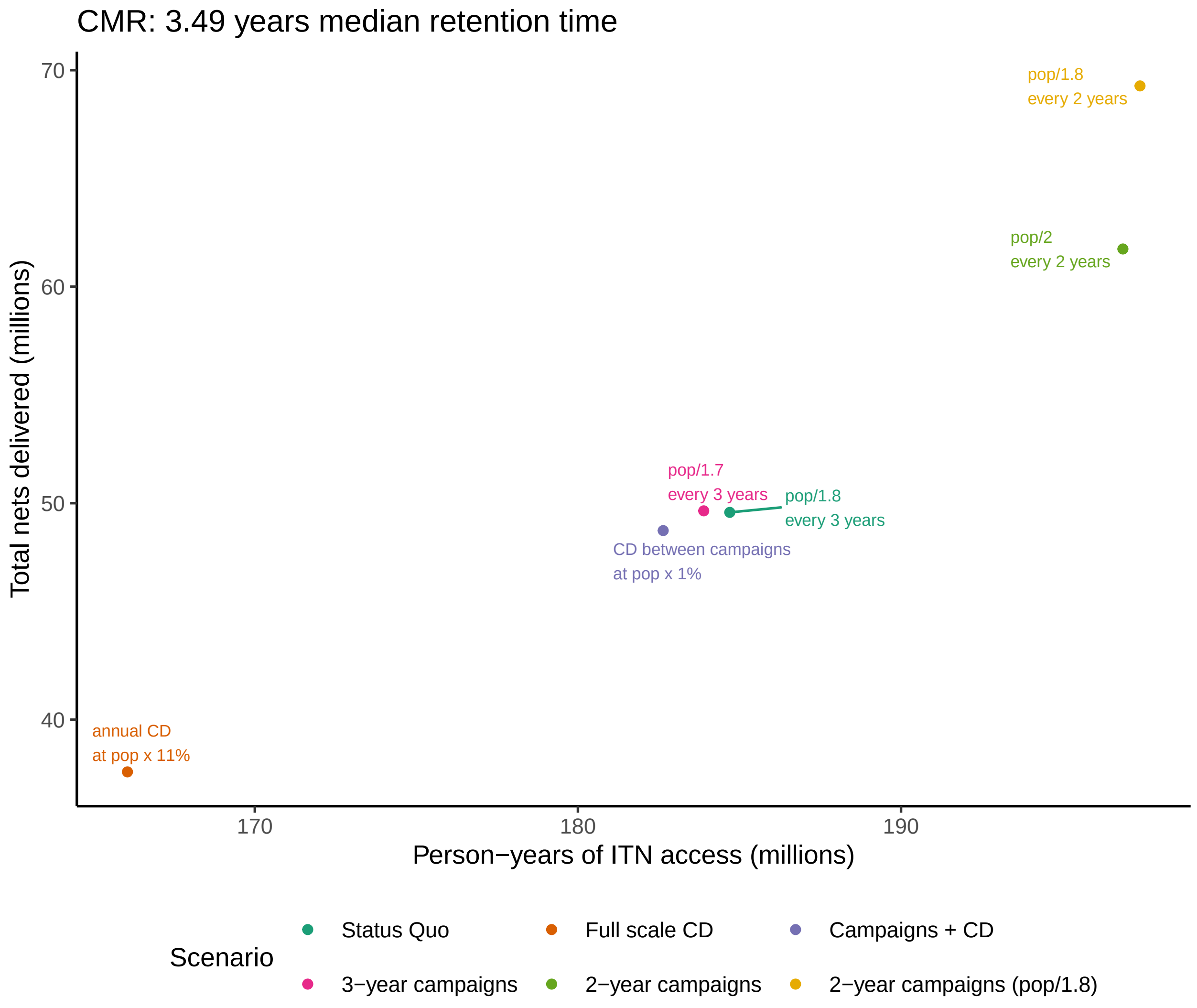

CMR: 3.49 years median retention time
Total nets delivered (millions)
70
60
50
40
pop/1.8
every 2 years
pop/2
every 2 years
pop/1.7
every 3 years
pop/1.8
every 3 years
CD between campaigns
at pop x 1%
annual CD
at pop x 11%
170
180
190
Person−years of ITN access (millions)
Scenario
Status Quo
Full scale CD
Campaigns + CD
3−year campaigns
2−year campaigns
2−year campaigns (pop/1.8)

COD: 1.41 years median retention time

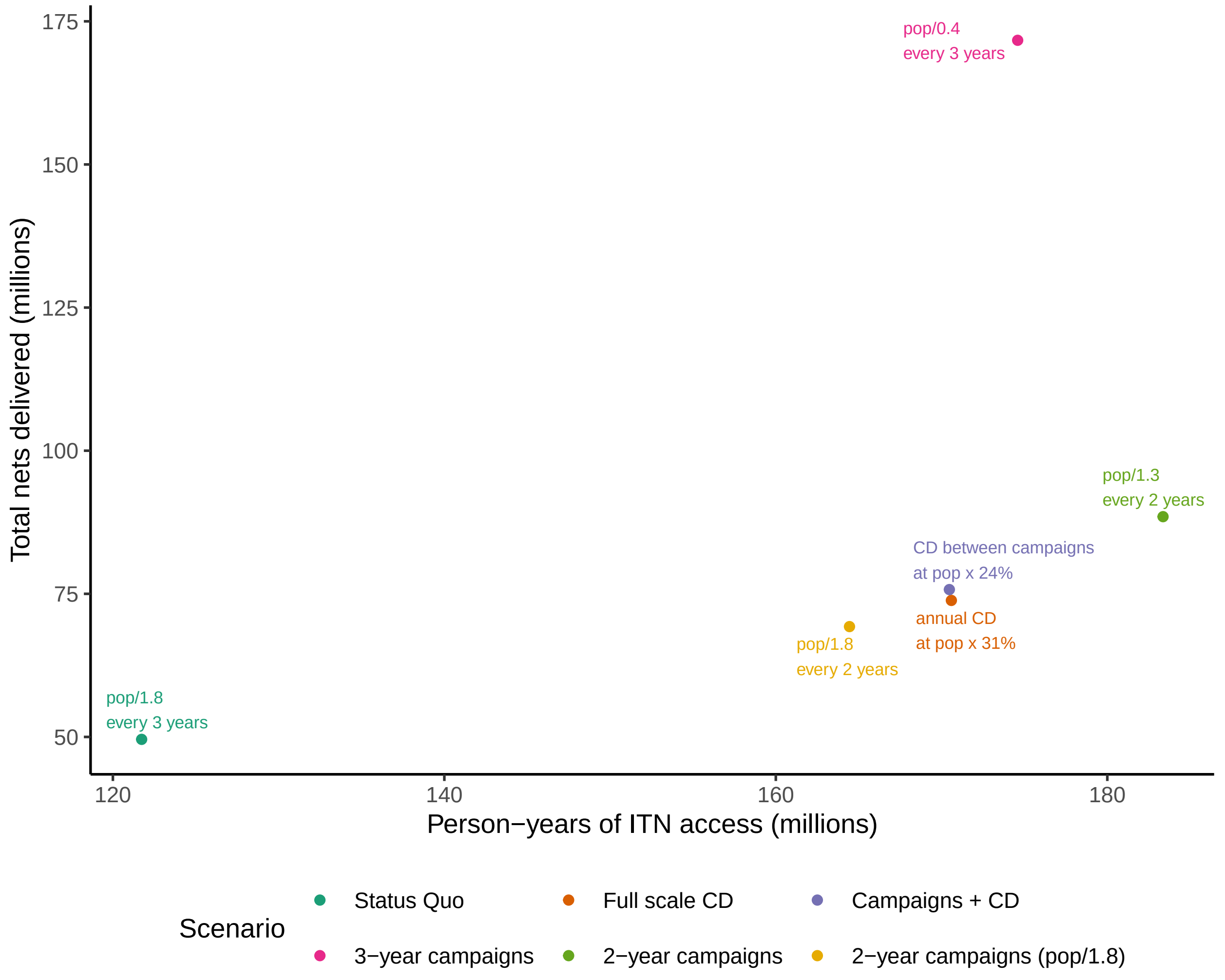

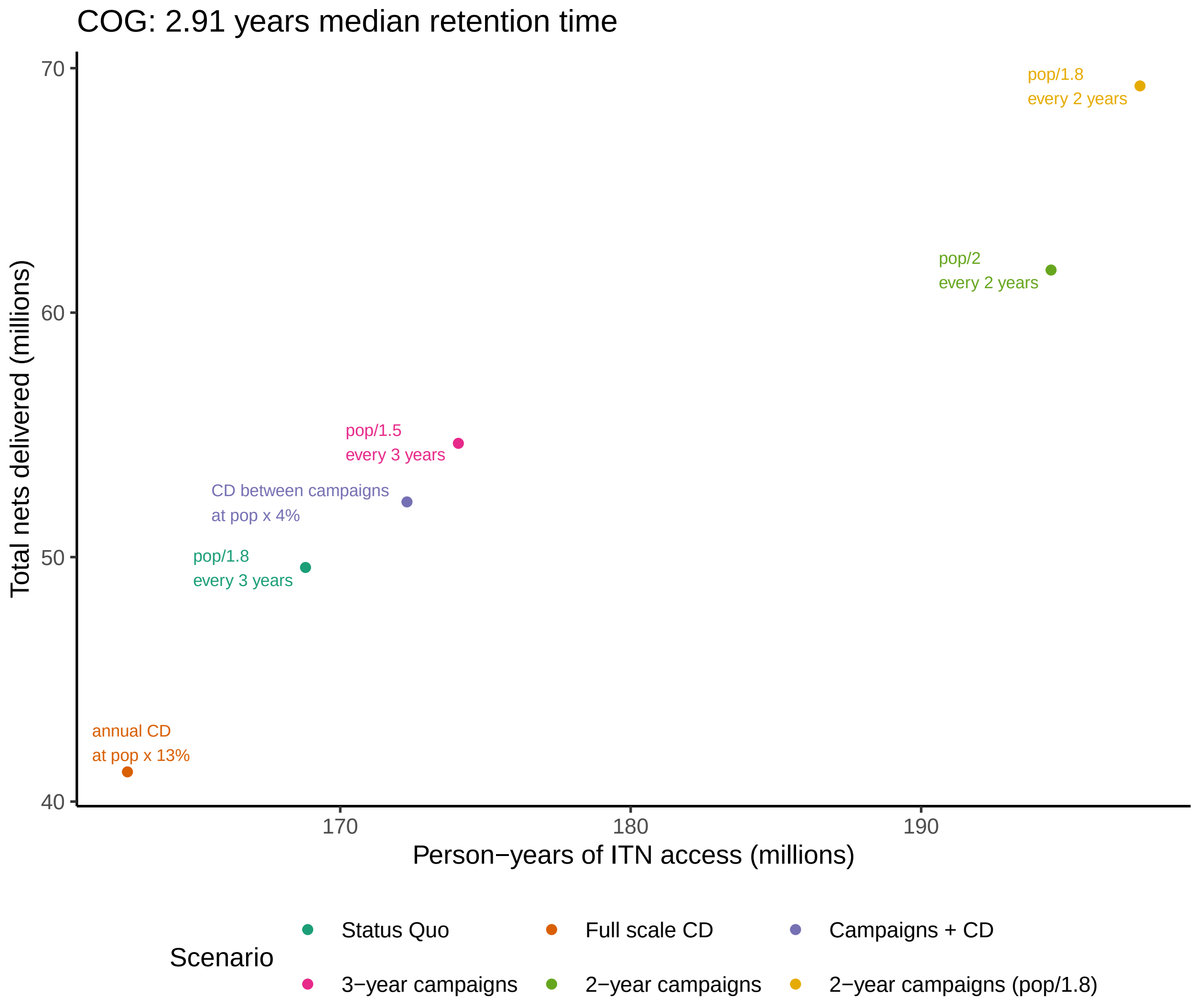
COG: 2.91 years median retention time
Total nets delivered (millions)
70
60
50
40
pop/1.8
every 2 years
pop/2
every 2 years
pop/1.5
every 3 years
CD between campaigns
at pop x 4%
pop/1.8
every 3 years
annual CD
at pop x 13%
170
180
190
Person−years of ITN access (millions)
Scenario
Status Quo
Full scale CD
Campaigns + CD
3−year campaigns
2−year campaigns
2−year campaigns (pop/1.8)

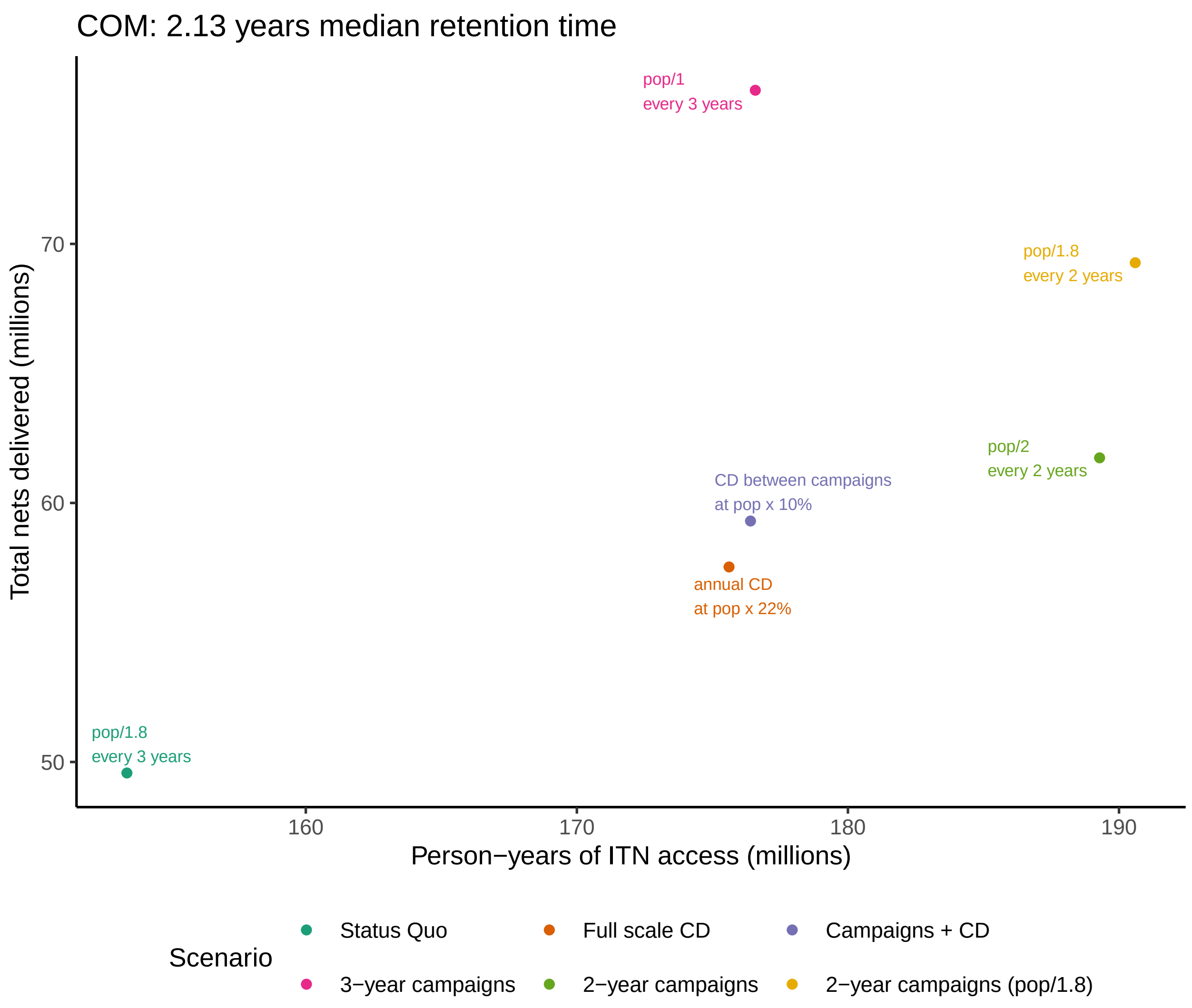

COM: 2.13 years median retention time
Total nets delivered (millions)
Person−years of ITN access (millions)
70
60
50
160
170
180
190
pop/1
every 3 years
pop/1.8
every 2 years
pop/2
every 2 years
CD between campaigns
at pop x 10%
annual CD
at pop x 22%
pop/1.8
every 3 years
Scenario
Status Quo
Full scale CD
Campaigns + CD
3−year campaigns
2−year campaigns
2−year campaigns (pop/1.8)

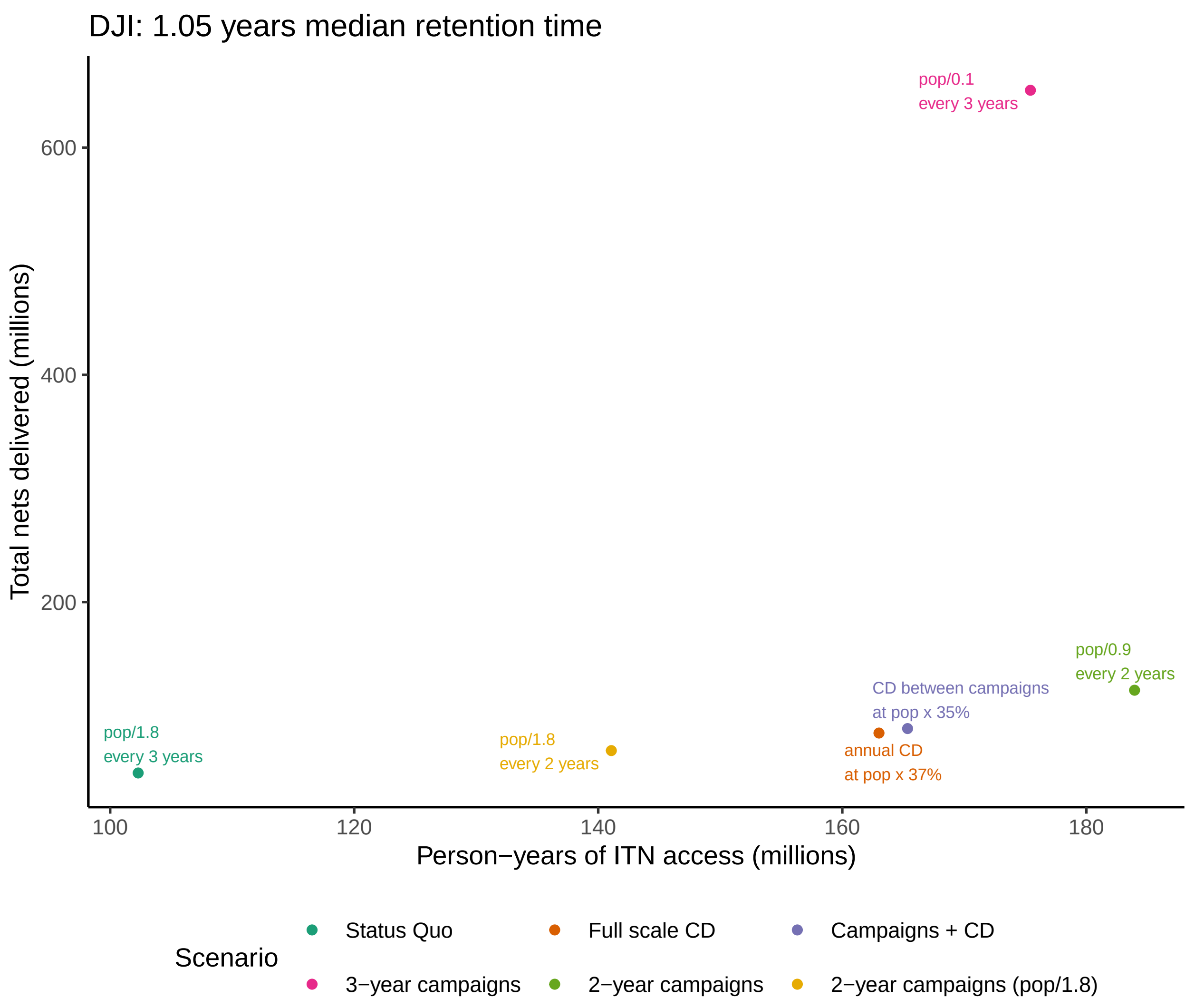
DJI: 1.05 years median retention time
Total nets delivered (millions)
600
400
200
100
120
140
160
180
Person−years of ITN access (millions)
pop/0.1
every 3 years
pop/0.9
every 2 years
CD between campaigns
at pop x 35%
annual CD
at pop x 37%
pop/1.8
every 3 years
pop/1.8
every 2 years
Scenario
Status Quo
Full scale CD
Campaigns + CD
3−year campaigns
2−year campaigns
2−year campaigns (pop/1.8)

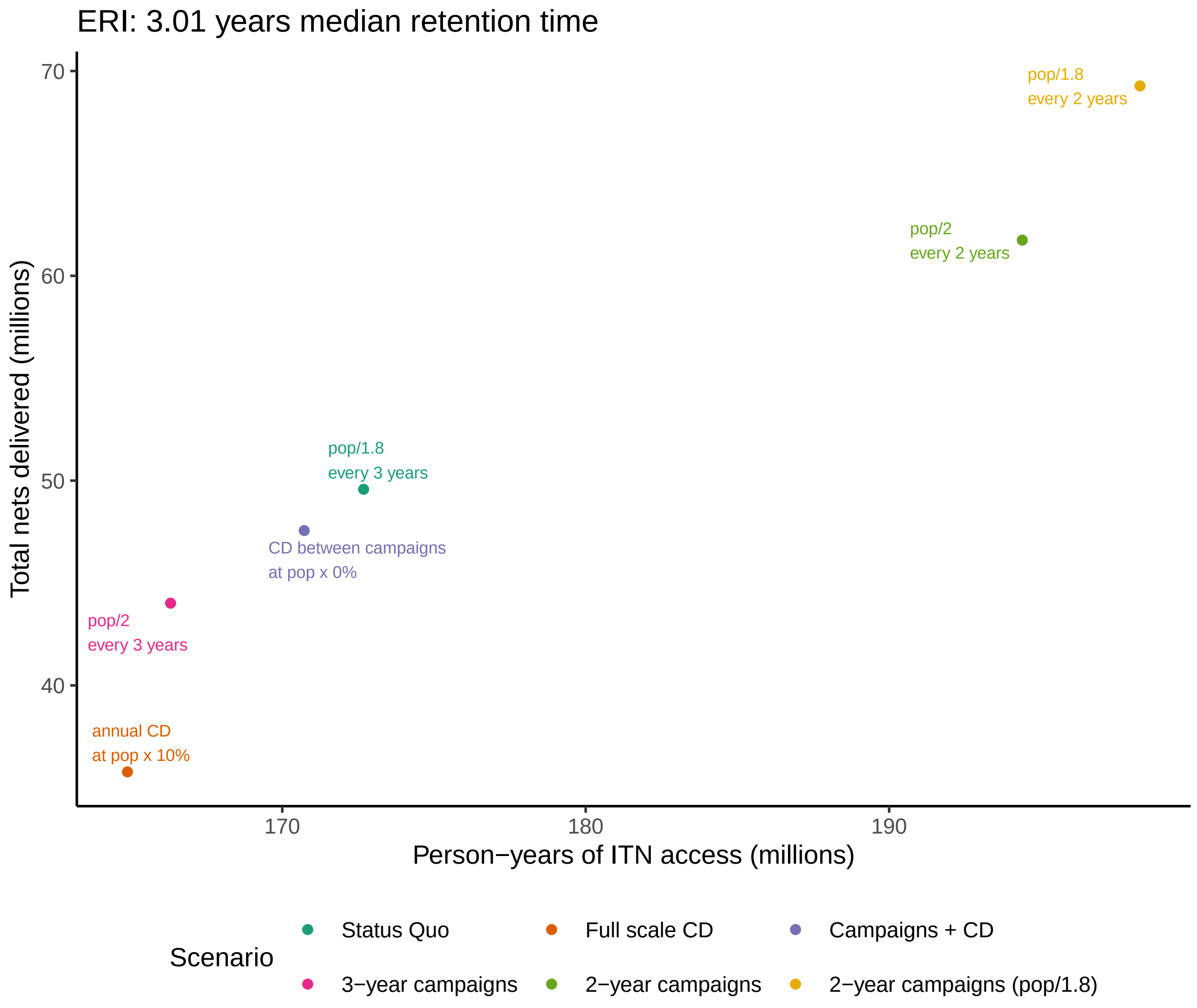
ERI: 3.01 years median retention time
Total nets delivered (millions)
70
60
50
40
pop/1.8
every 2 years
pop/2
every 2 years
pop/1.8
every 3 years
CD between campaigns
at pop x 0%
pop/2
every 3 years
annual CD
at pop x 10%
170
180
190
Person−years of ITN access (millions)
Scenario
Status Quo
Full scale CD
Campaigns + CD
3−year campaigns
2−year campaigns
2−year campaigns (pop/1.8)

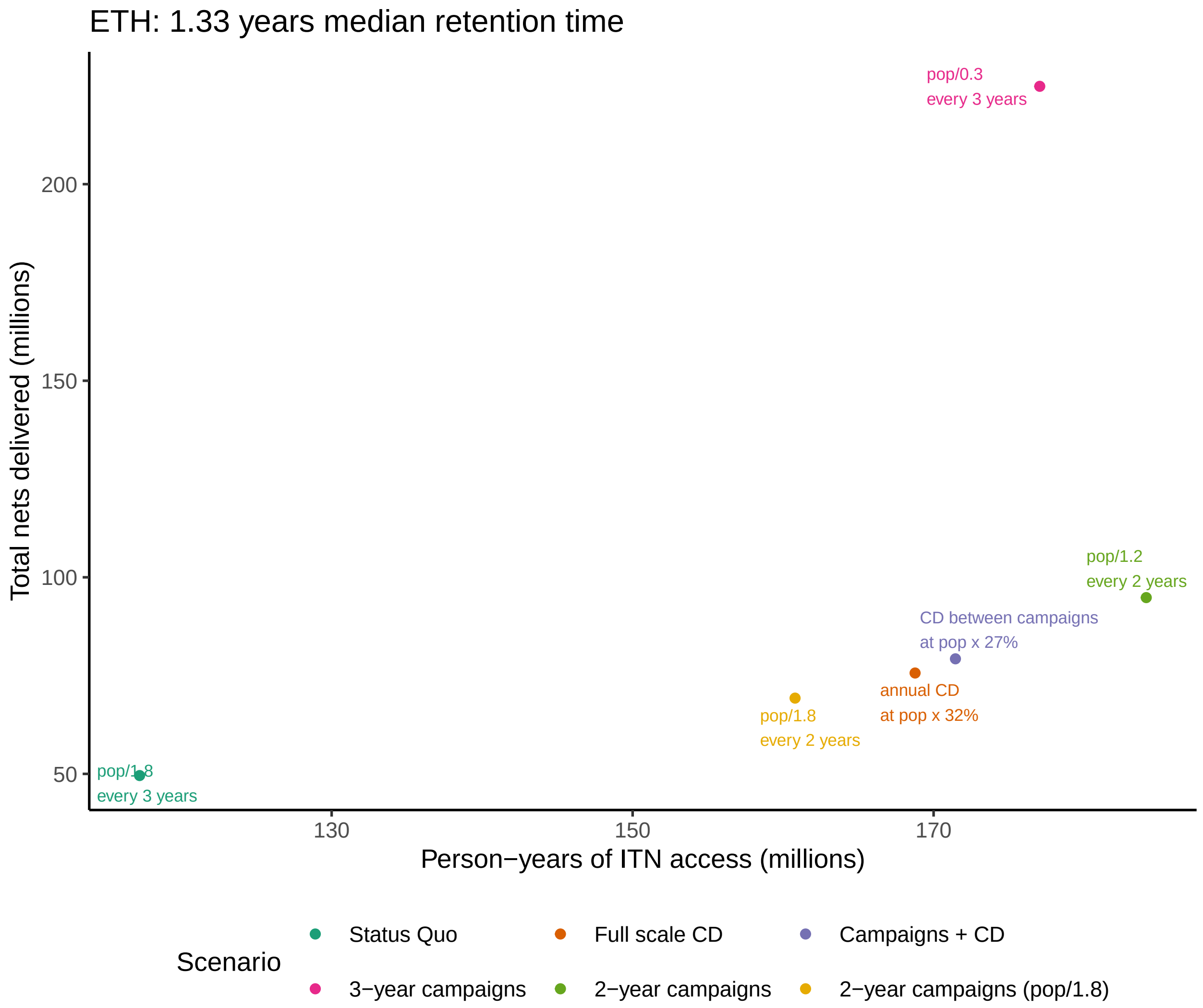
ETH: 1.33 years median retention time
Total nets delivered (millions)
200
150
100
50
130
150
170
Person−years of ITN access (millions)
pop/0.3
every 3 years
pop/1.2
every 2 years
CD between campaigns
at pop x 27%
annual CD
at pop x 32%
pop/1.8
every 2 years
pop/1.8
every 3 years
Scenario
Status Quo
Full scale CD
Campaigns + CD
3−year campaigns
2−year campaigns
2−year campaigns (pop/1.8)

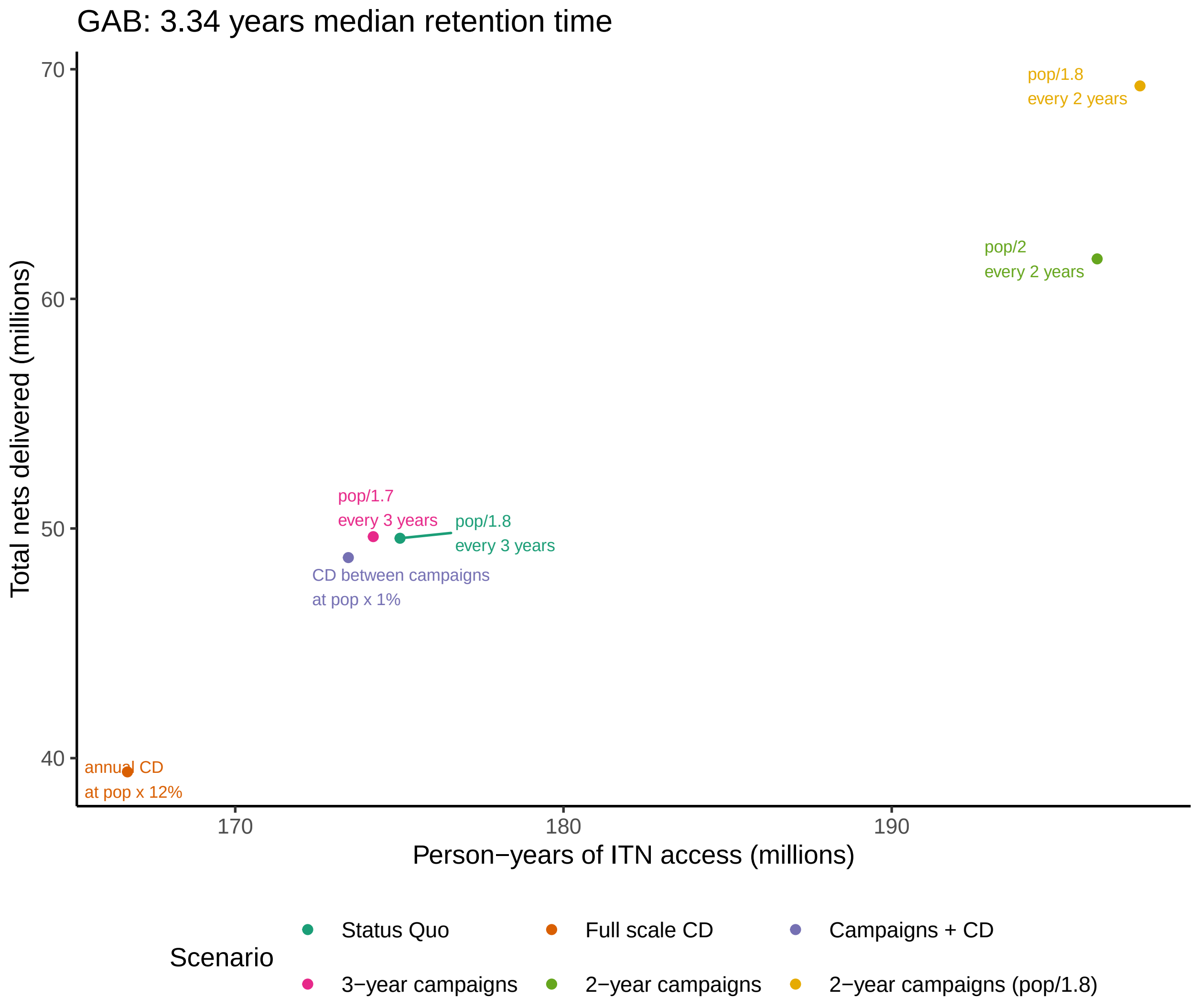

GAB: 3.34 years median retention time
Total nets delivered (millions)
Person−years of ITN access (millions)
70
60
50
40
170
180
190
pop/1.8
every 2 years
pop/2
every 2 years
pop/1.7
every 3 years
pop/1.8
every 3 years
CD between campaigns
at pop x 1%
annual CD
at pop x 12%
Scenario
Status Quo
Full scale CD
Campaigns + CD
3−year campaigns
2−year campaigns
2−year campaigns (pop/1.8)

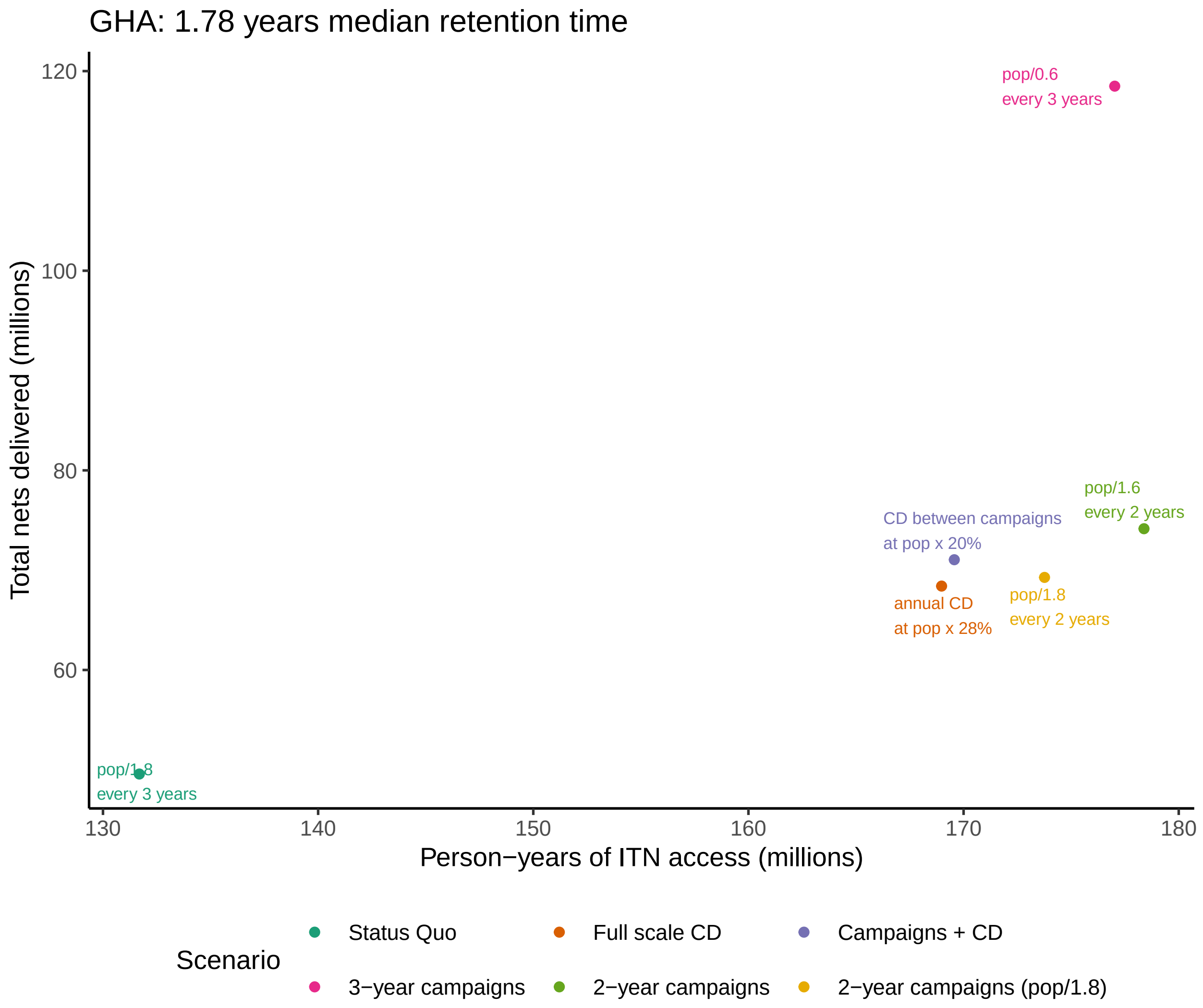
GHA: 1.78 years median retention time
Total nets delivered (millions)
120
100
80
60
pop/0.6
every 3 years
pop/1.6
every 2 years
CD between campaigns
at pop x 20%
annual CD
at pop x 28%
pop/1.8
every 2 years
pop/1.8
every 3 years
130
140
150
160
170
180
Person−years of ITN access (millions)
Scenario
Status Quo
Full scale CD
Campaigns + CD
3−year campaigns
2−year campaigns
2−year campaigns (pop/1.8)

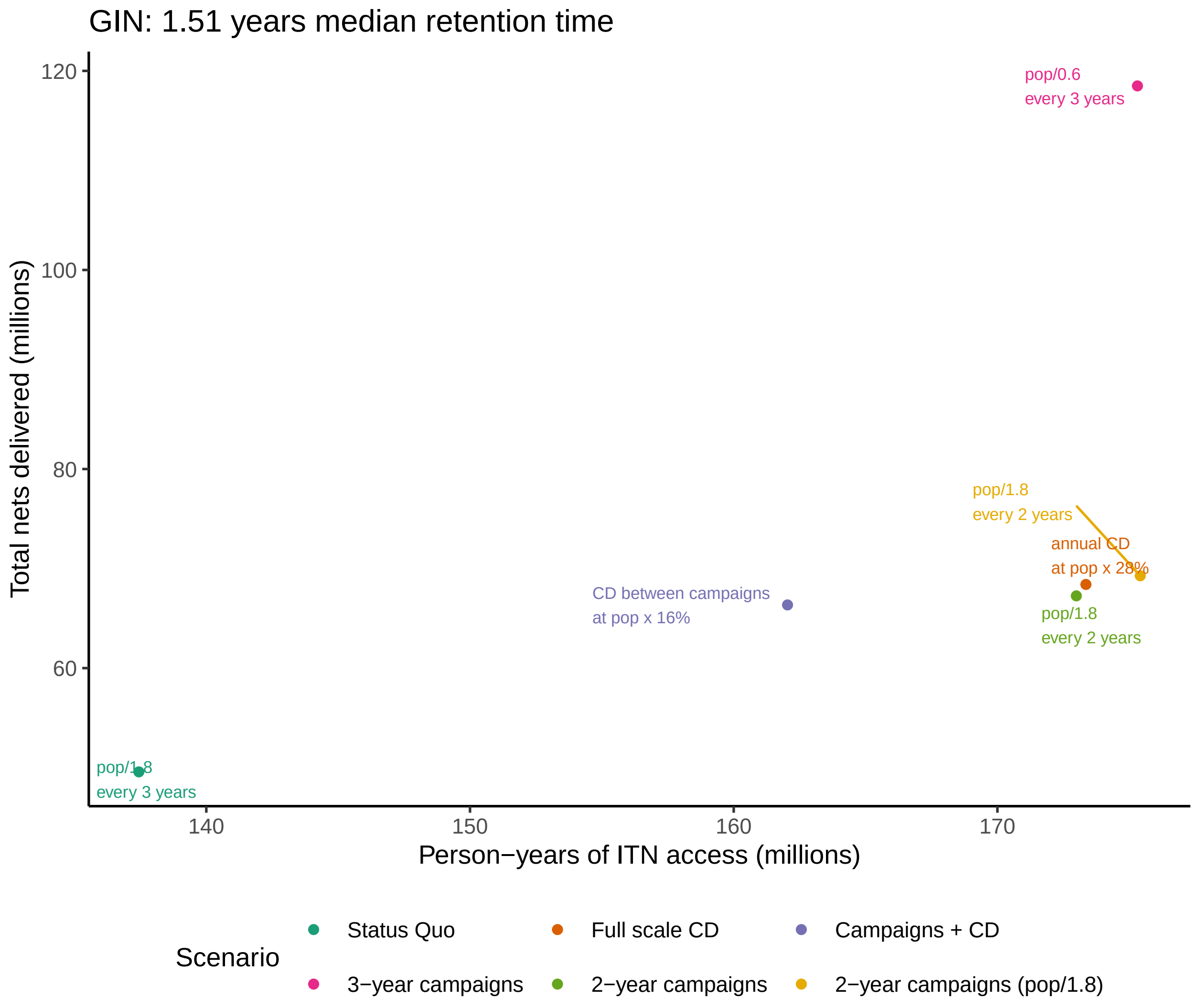
GIN: 1.51 years median retention time
Total nets delivered (millions)
120
100
80
60
pop/0.6
every 3 years
pop/1.8
every 2 years
annual CD
at pop x 28%
CD between campaigns
at pop x 16%
pop/1.8
every 2 years
pop/1.8
every 3 years
140
150
160
170
Person−years of ITN access (millions)
Scenario
Status Quo
Full scale CD
Campaigns + CD
3−year campaigns
2−year campaigns
2−year campaigns (pop/1.8)

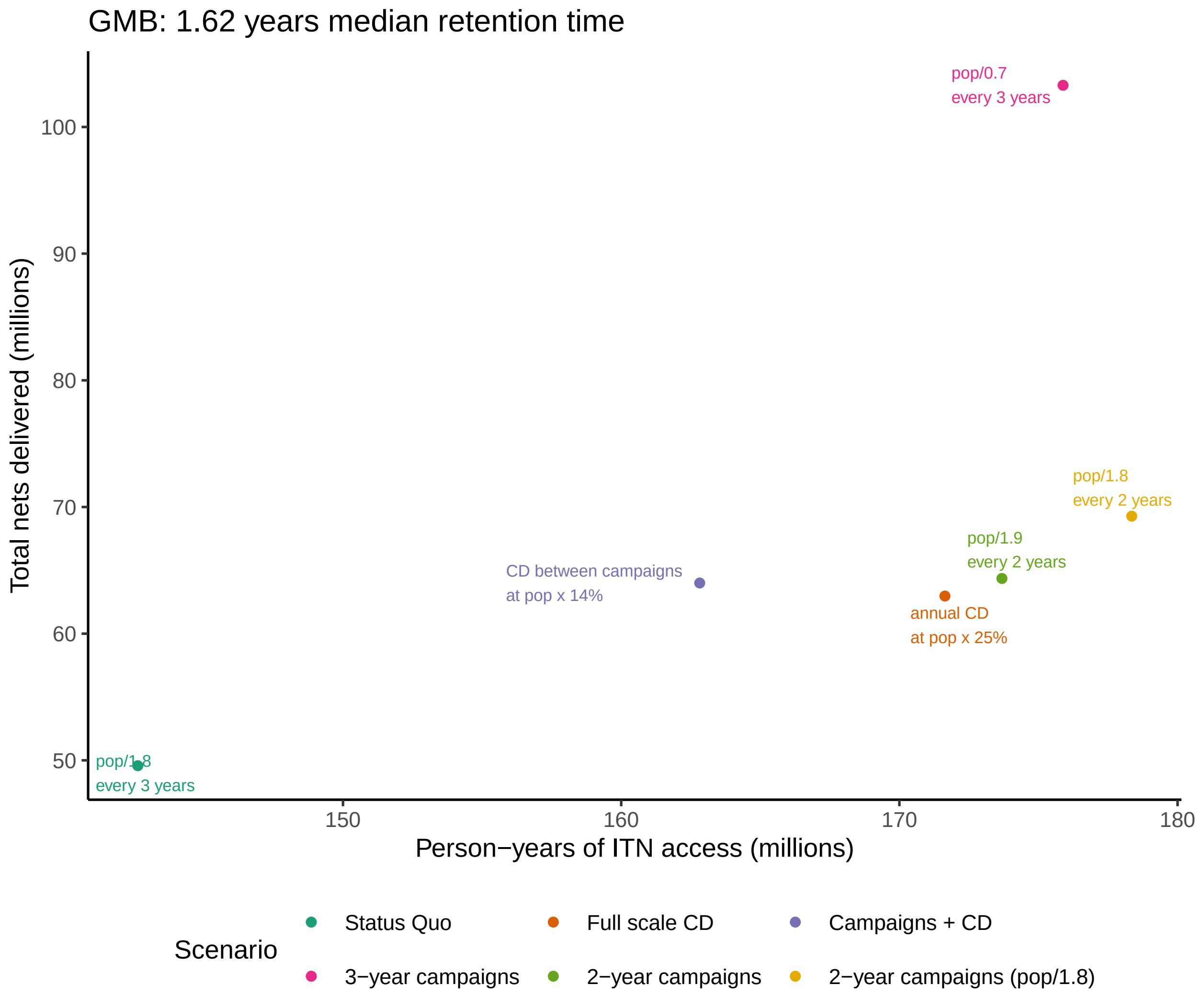
GMB: 1.62 years median retention time
Total nets delivered (millions)
100
90
80
70
60
50
pop/0.7
every 3 years
pop/1.8
every 2 years
pop/1.9
every 2 years
CD between campaigns
at pop x 14%
annual CD
at pop x 25%
pop/1.8
every 3 years
150
160
170
180
Person−years of ITN access (millions)
Scenario
Status Quo
Full scale CD
Campaigns + CD
3−year campaigns
2−year campaigns
2−year campaigns (pop/1.8)

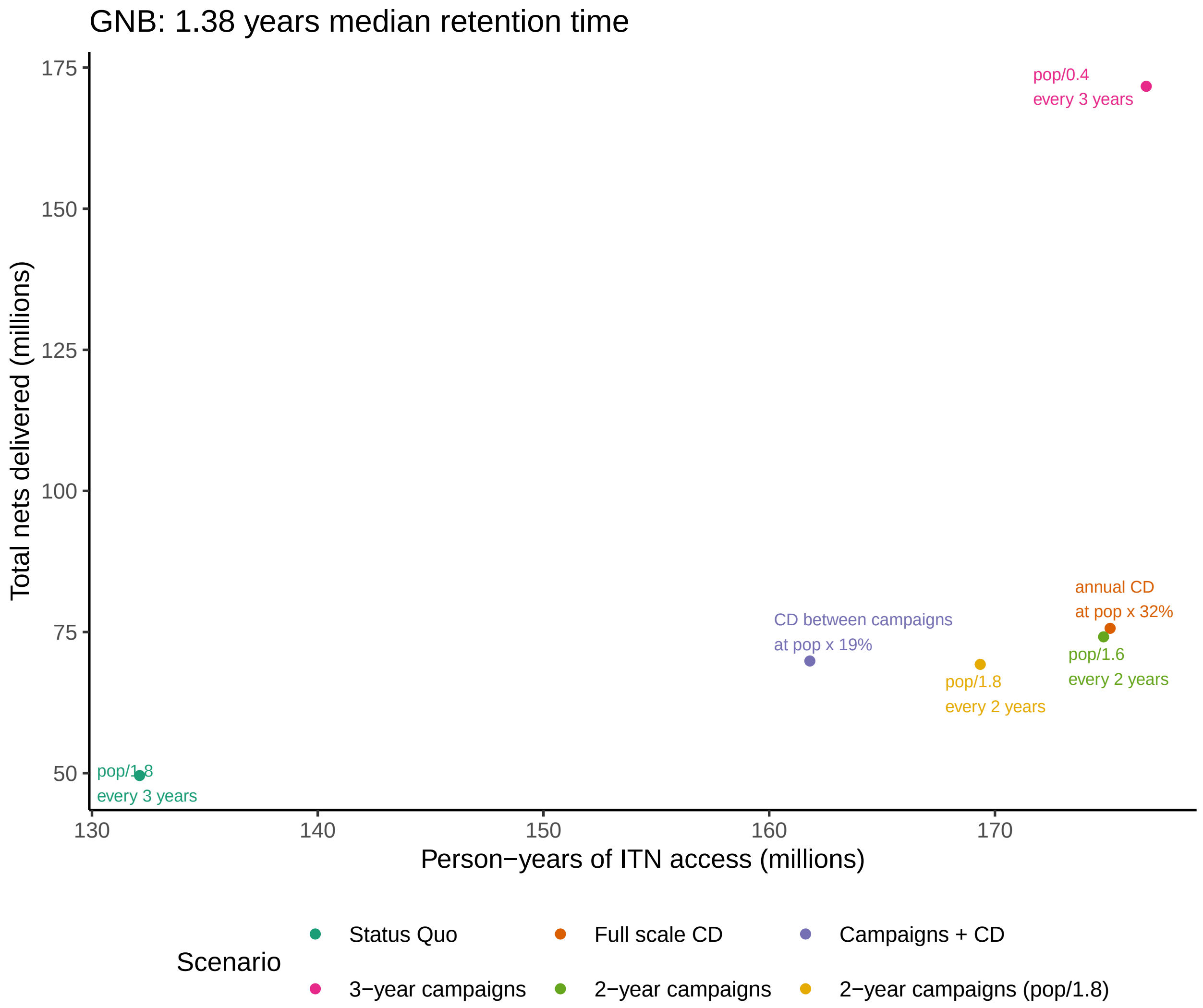

GNB: 1.38 years median retention time
Total nets delivered (millions)
175
150
125
100
75
50
130
140
150
160
170
Person−years of ITN access (millions)
pop/0.4
every 3 years
annual CD
at pop x 32%
CD between campaigns
at pop x 19%
pop/1.6
every 2 years
pop/1.8
every 2 years
pop/1.8
every 3 years
Scenario
Status Quo
Full scale CD
Campaigns + CD
3−year campaigns
2−year campaigns
2−year campaigns (pop/1.8)

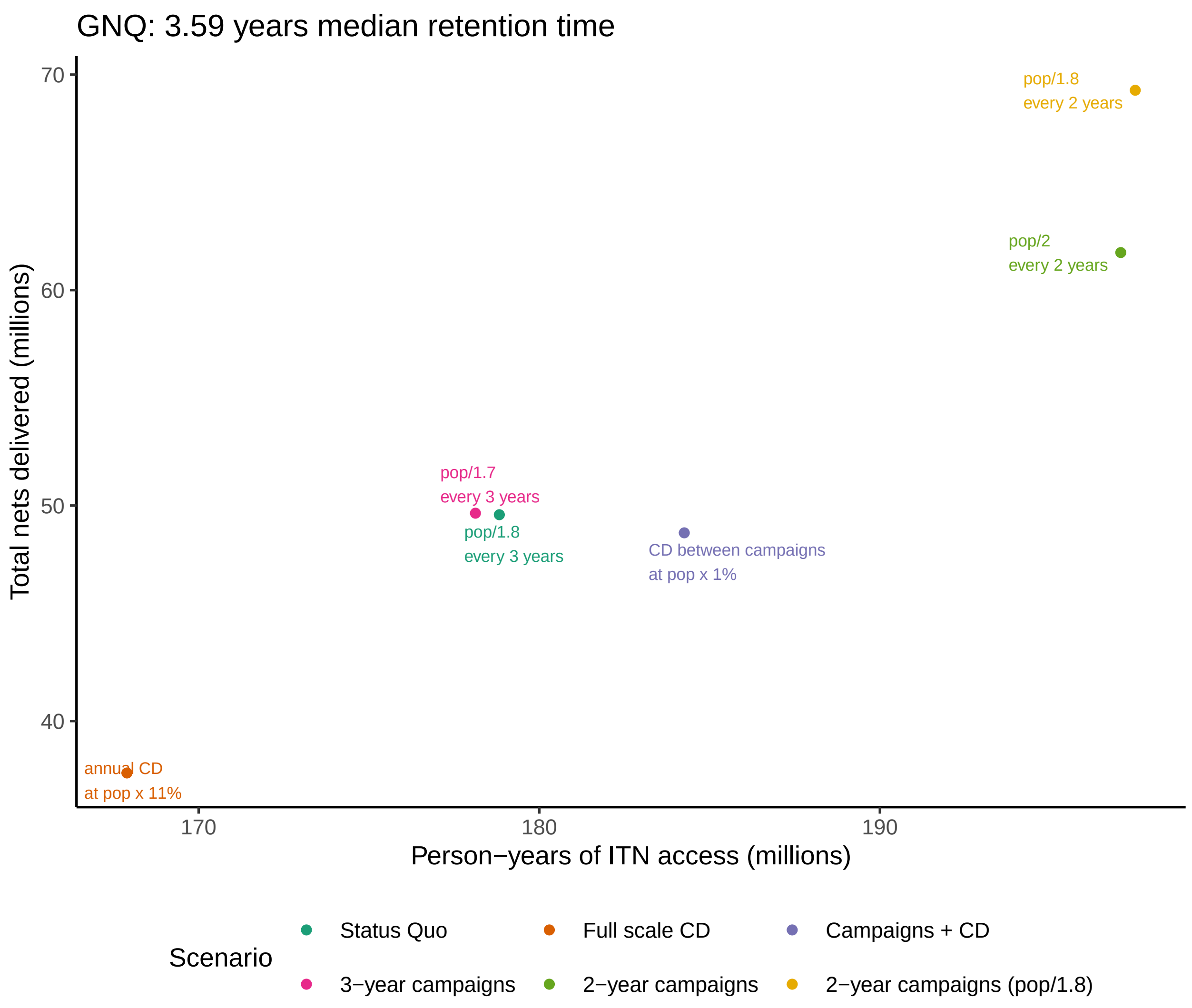
GNQ: 3.59 years median retention time
Total nets delivered (millions)
70
60
50
40
Person−years of ITN access (millions)
170
180
190
pop/1.8
every 2 years
pop/2
every 2 years
pop/1.7
every 3 years
pop/1.8
every 3 years
CD between campaigns
at pop x 1%
annual CD
at pop x 11%
Scenario
Status Quo
Full scale CD
Campaigns + CD
3−year campaigns
2−year campaigns
2−year campaigns (pop/1.8)

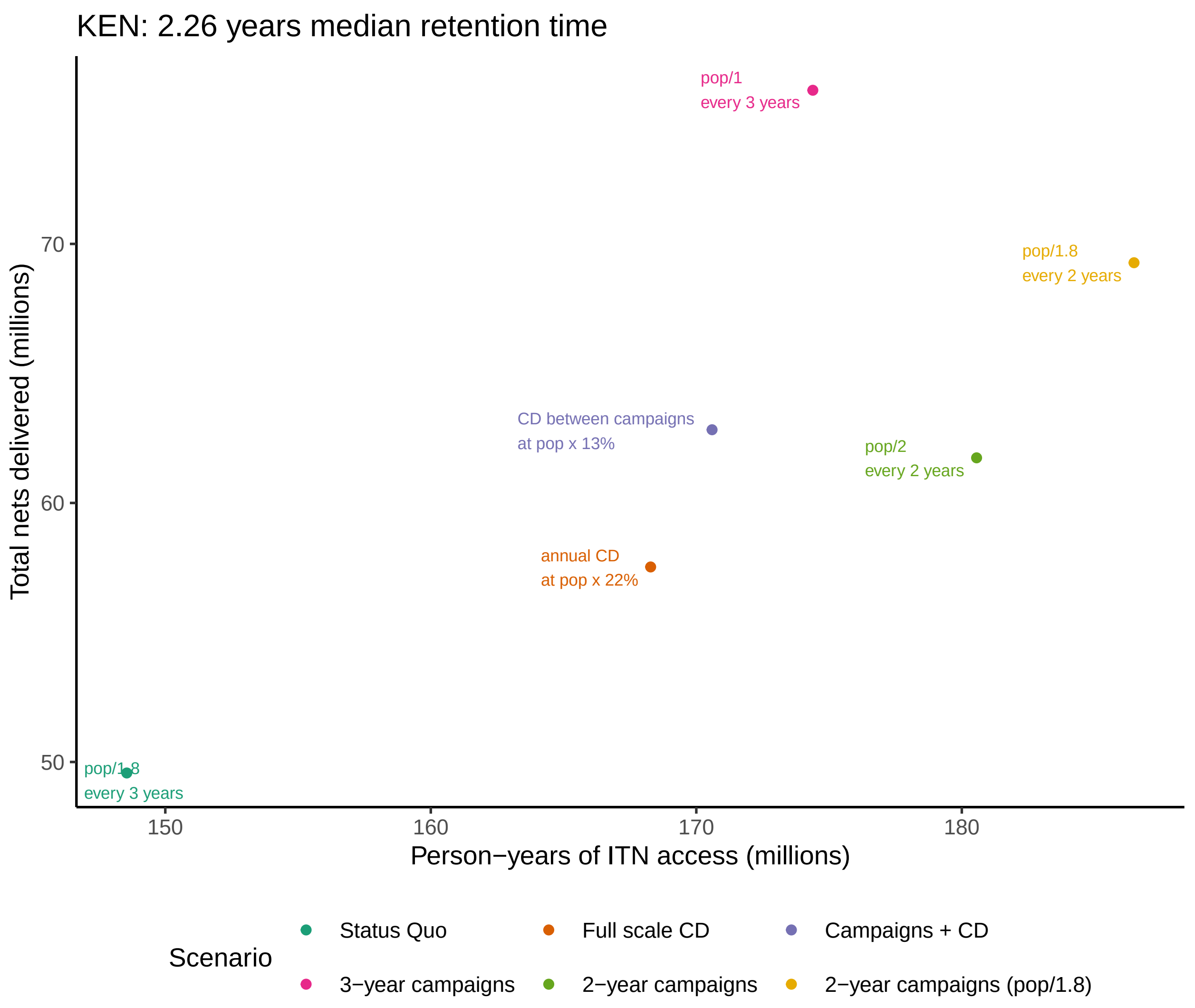
KEN: 2.26 years median retention time
pop/1
every 3 years
pop/1.8
every 2 years
CD between campaigns
at pop x 13%
pop/2
every 2 years
annual CD
at pop x 22%
pop/1.8
every 3 years
Total nets delivered (millions)
70
60
50
150
160
170
180
Person−years of ITN access (millions)
Scenario
Status Quo
Full scale CD
Campaigns + CD
3−year campaigns
2−year campaigns
2−year campaigns (pop/1.8)

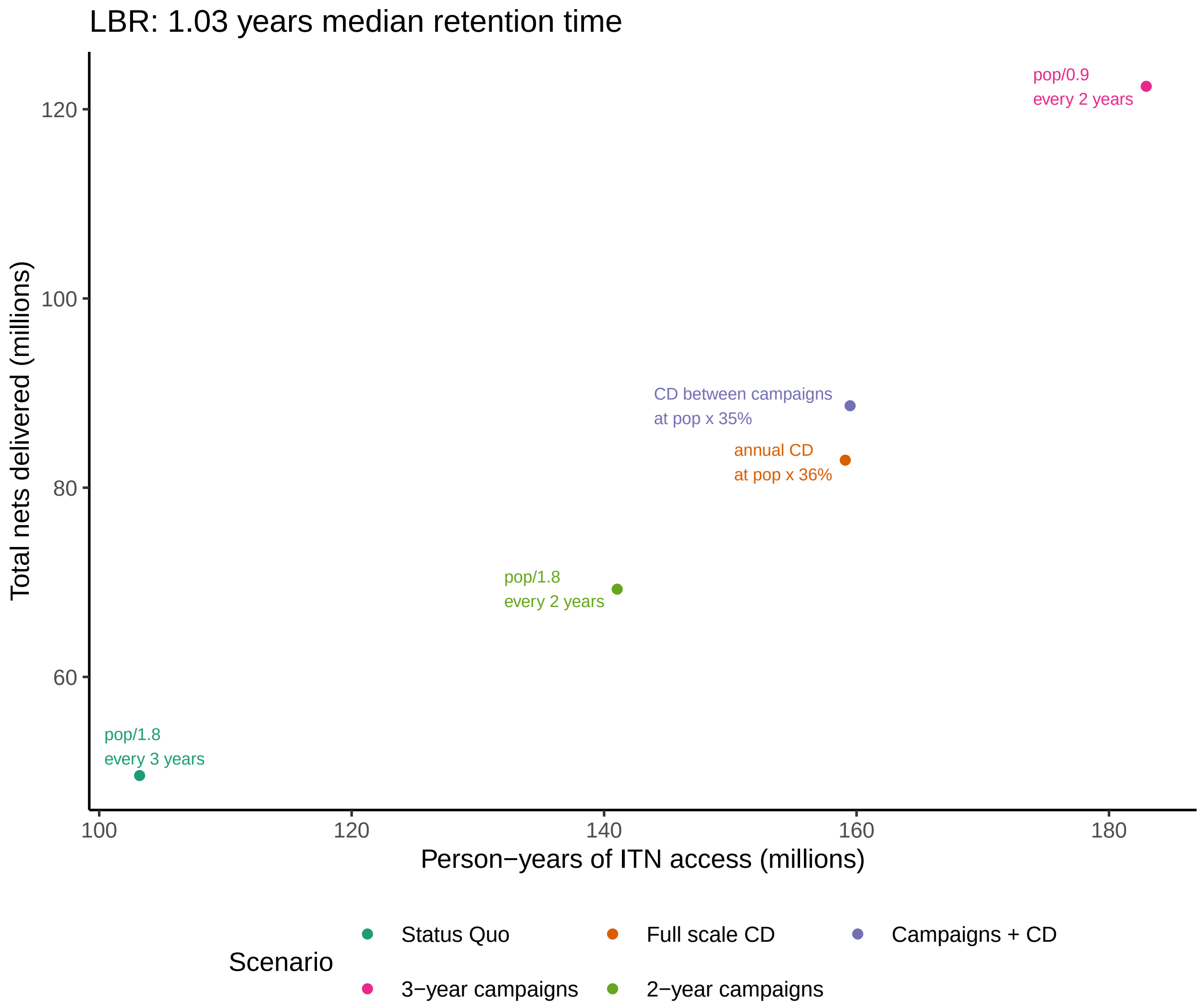

LBR: 1.03 years median retention time
Total nets delivered (millions)
Person−years of ITN access (millions)
120
100
80
60
100
120
140
160
180
pop/0.9
every 2 years
CD between campaigns
at pop x 35%
annual CD
at pop x 36%
pop/1.8
every 2 years
pop/1.8
every 3 years
Scenario
Status Quo
Full scale CD
Campaigns + CD
3−year campaigns
2−year campaigns

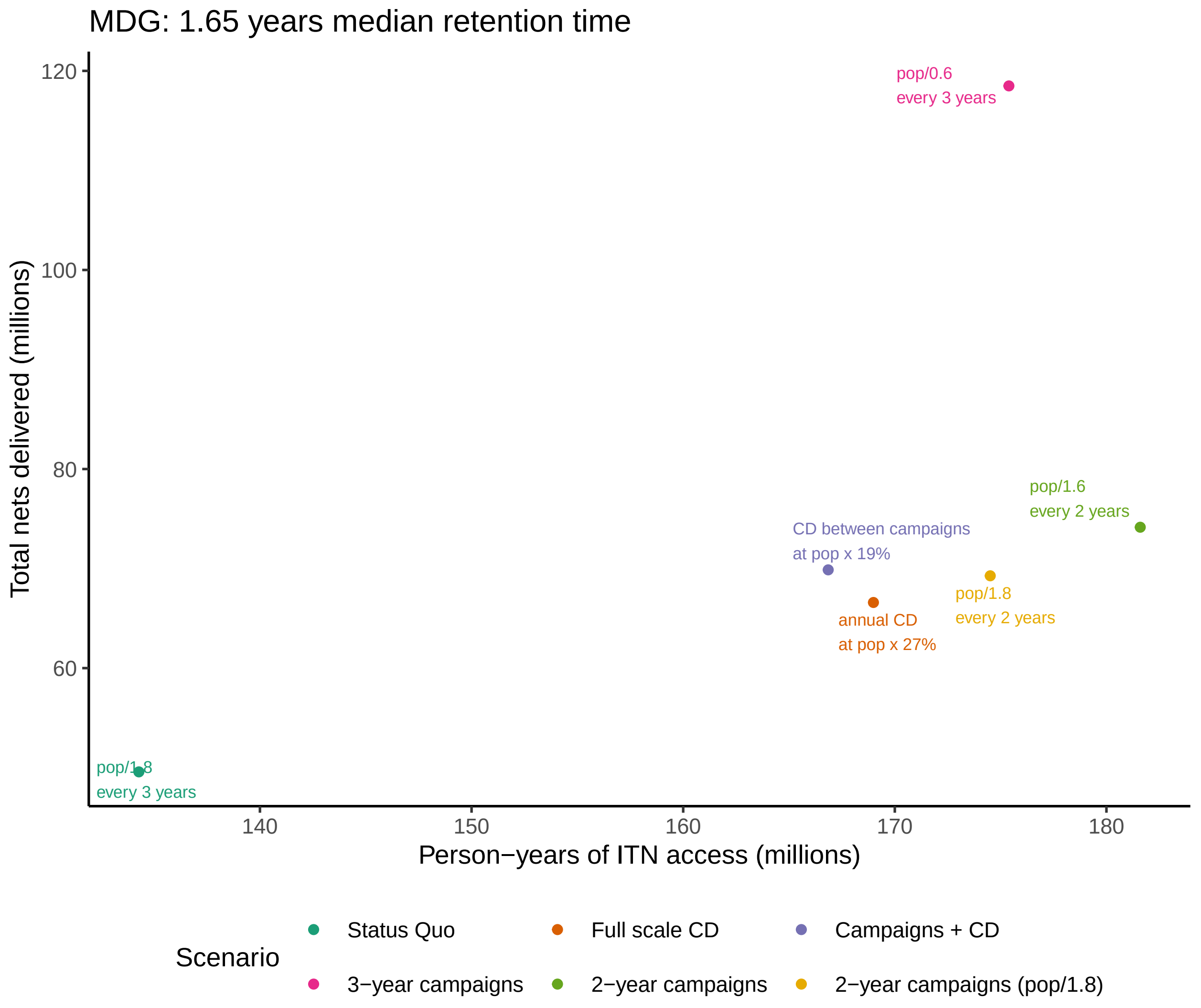

MDG: 1.65 years median retention time
Total nets delivered (millions)
120
100
80
60
pop/0.6
every 3 years
pop/1.6
every 2 years
CD between campaigns
at pop x 19%
pop/1.8
every 2 years
annual CD
at pop x 27%
pop/1.8
every 3 years
140
150
160
170
180
Person−years of ITN access (millions)
Scenario
Status Quo
Full scale CD
Campaigns + CD
3−year campaigns
2−year campaigns
2−year campaigns (pop/1.8)

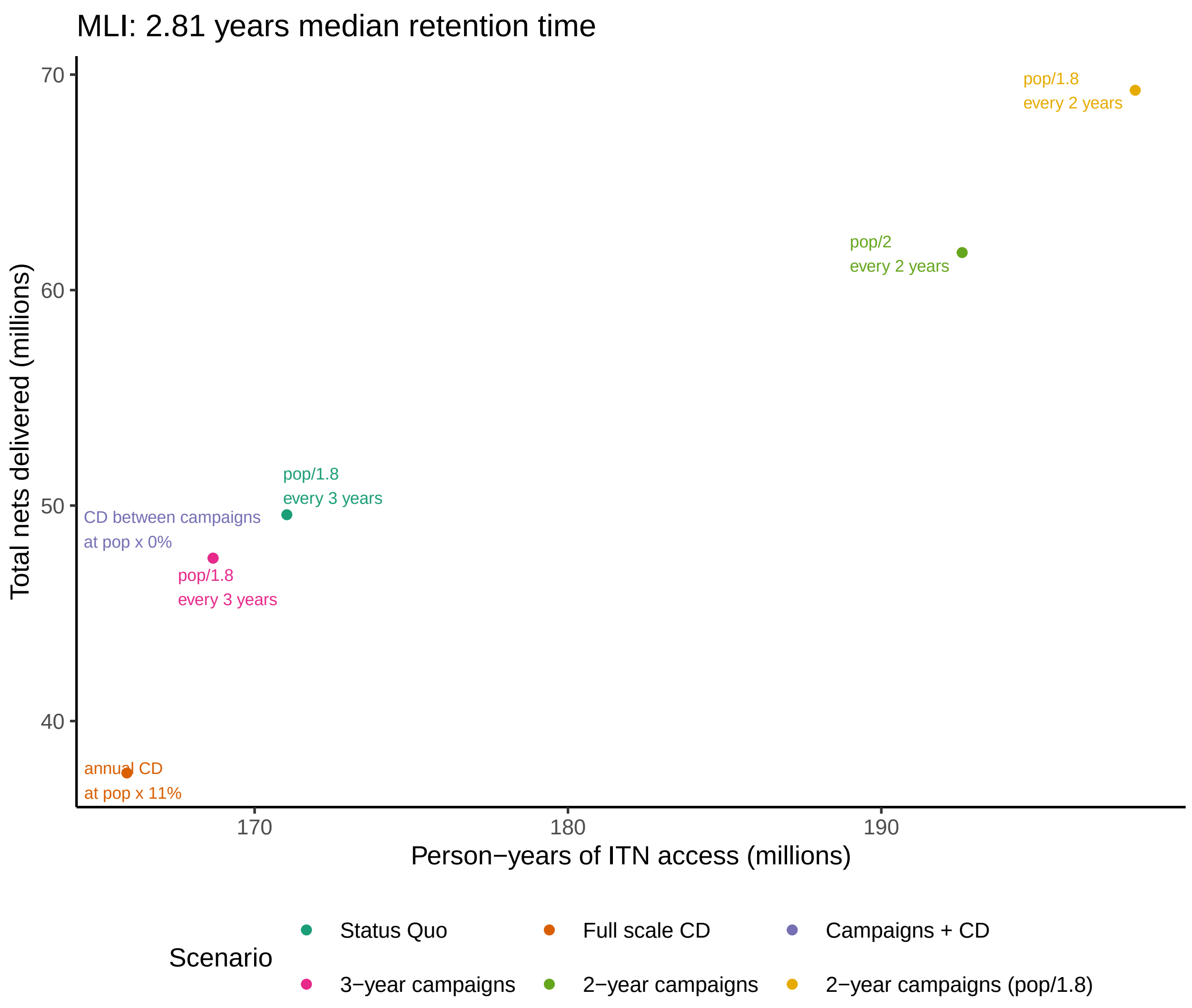

MLI: 2.81 years median retention time
Total nets delivered (millions)
70
60
50
40
pop/1.8
every 2 years
pop/2
every 2 years
pop/1.8
every 3 years
CD between campaigns
at pop x 0%
pop/1.8
every 3 years
annual CD
at pop x 11%
170
180
190
Person−years of ITN access (millions)
Scenario
Status Quo
Full scale CD
Campaigns + CD
3−year campaigns
2−year campaigns
2−year campaigns (pop/1.8)

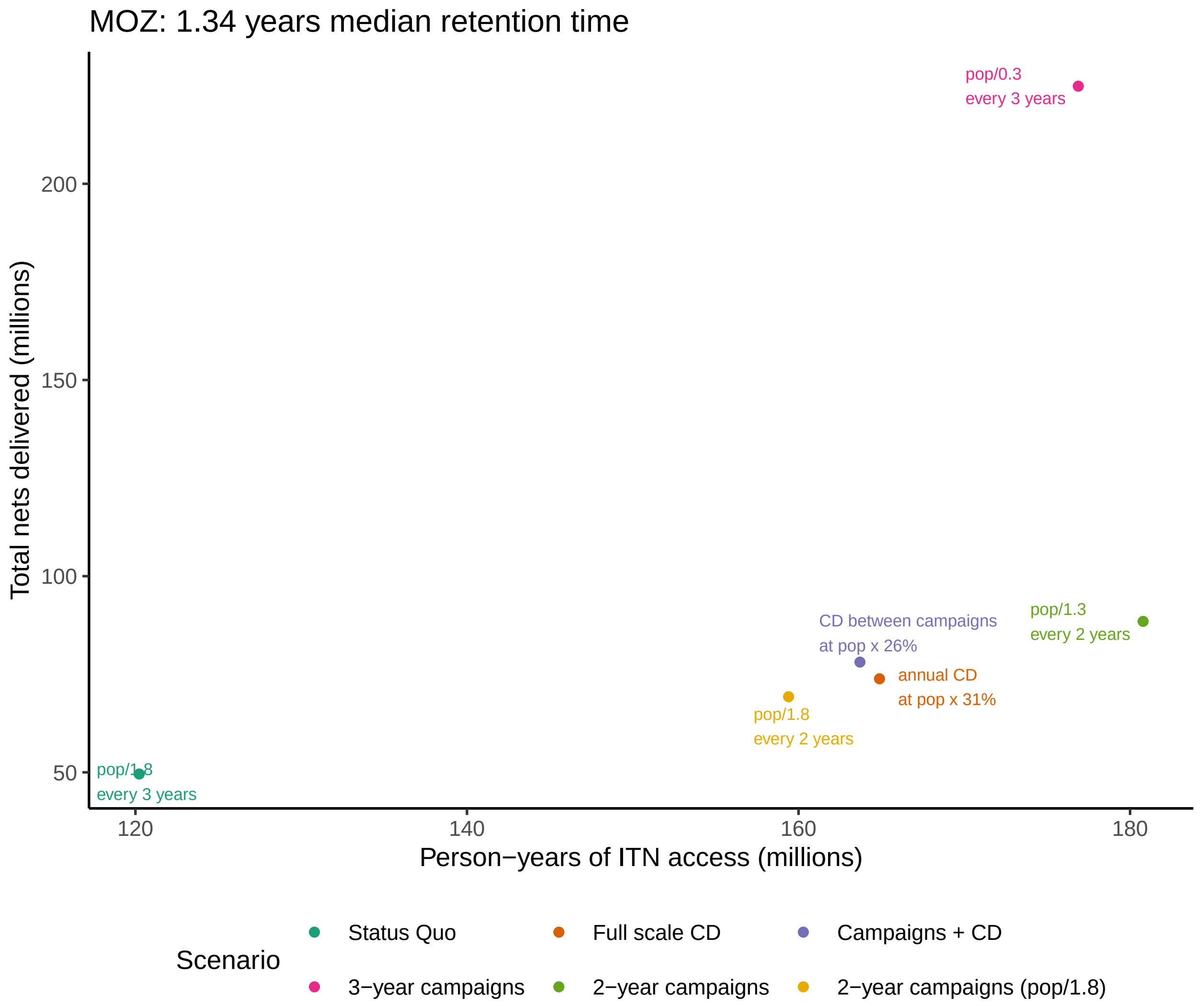
MOZ: 1.34 years median retention time
pop/0.3
every 3 years
CD between campaigns
at pop x 26%
pop/1.3
every 2 years
annual CD
at pop x 31%
pop/1.8
every 2 years
pop/1.8
every 3 years
Total nets delivered (millions)
200
150
100
50
120
140
160
180
Person−years of ITN access (millions)
Scenario
Status Quo
Full scale CD
Campaigns + CD
3−year campaigns
2−year campaigns
2−year campaigns (pop/1.8)

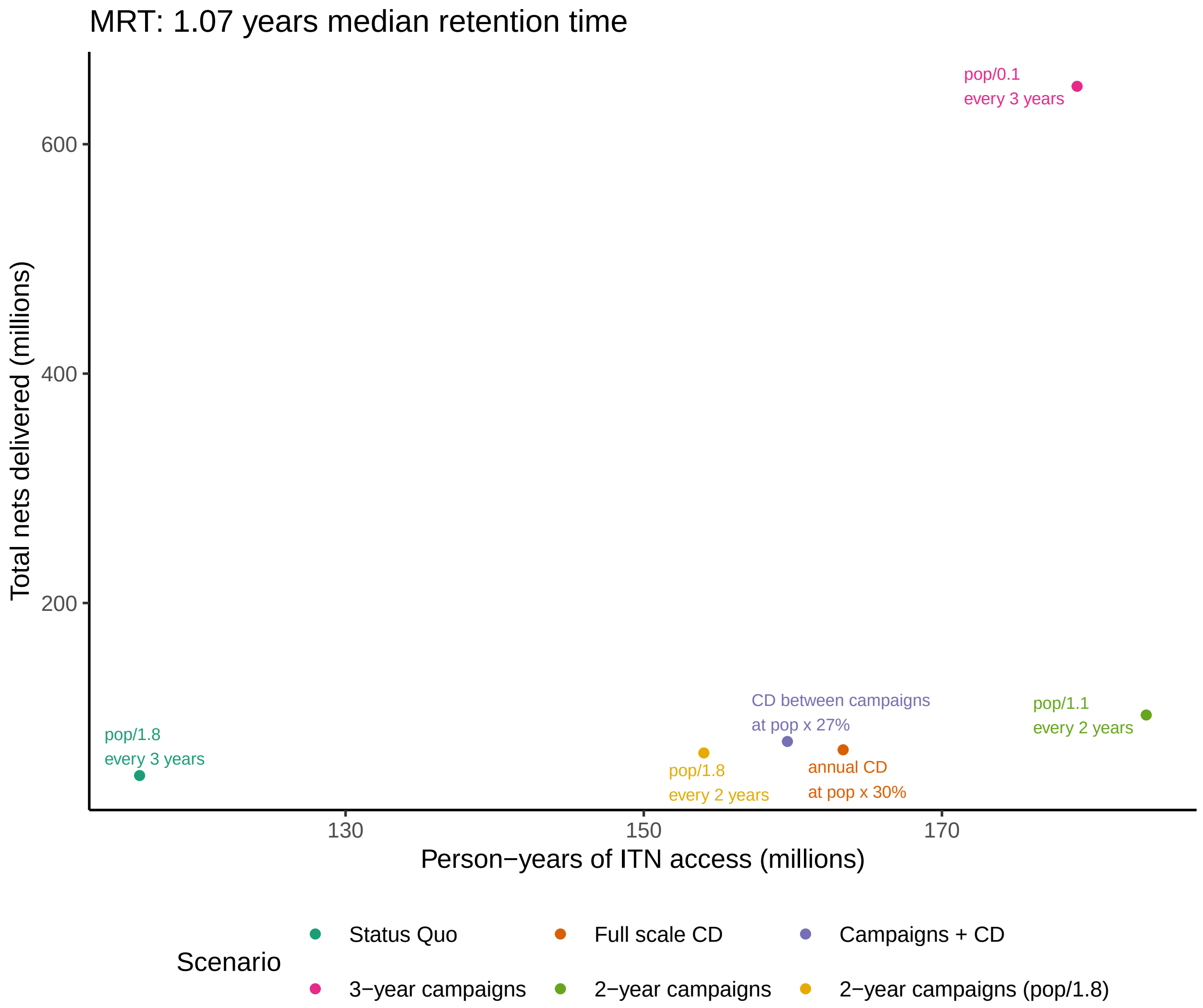

MRT: 1.07 years median retention time
Total nets delivered (millions)
600
400
200
pop/0.1
every 3 years
CD between campaigns
at pop x 27%
pop/1.1
every 2 years
pop/1.8
every 3 years
pop/1.8
every 2 years
annual CD
at pop x 30%
130
150
170
Person−years of ITN access (millions)
Scenario
Status Quo
Full scale CD
Campaigns + CD
3−year campaigns
2−year campaigns
2−year campaigns (pop/1.8)

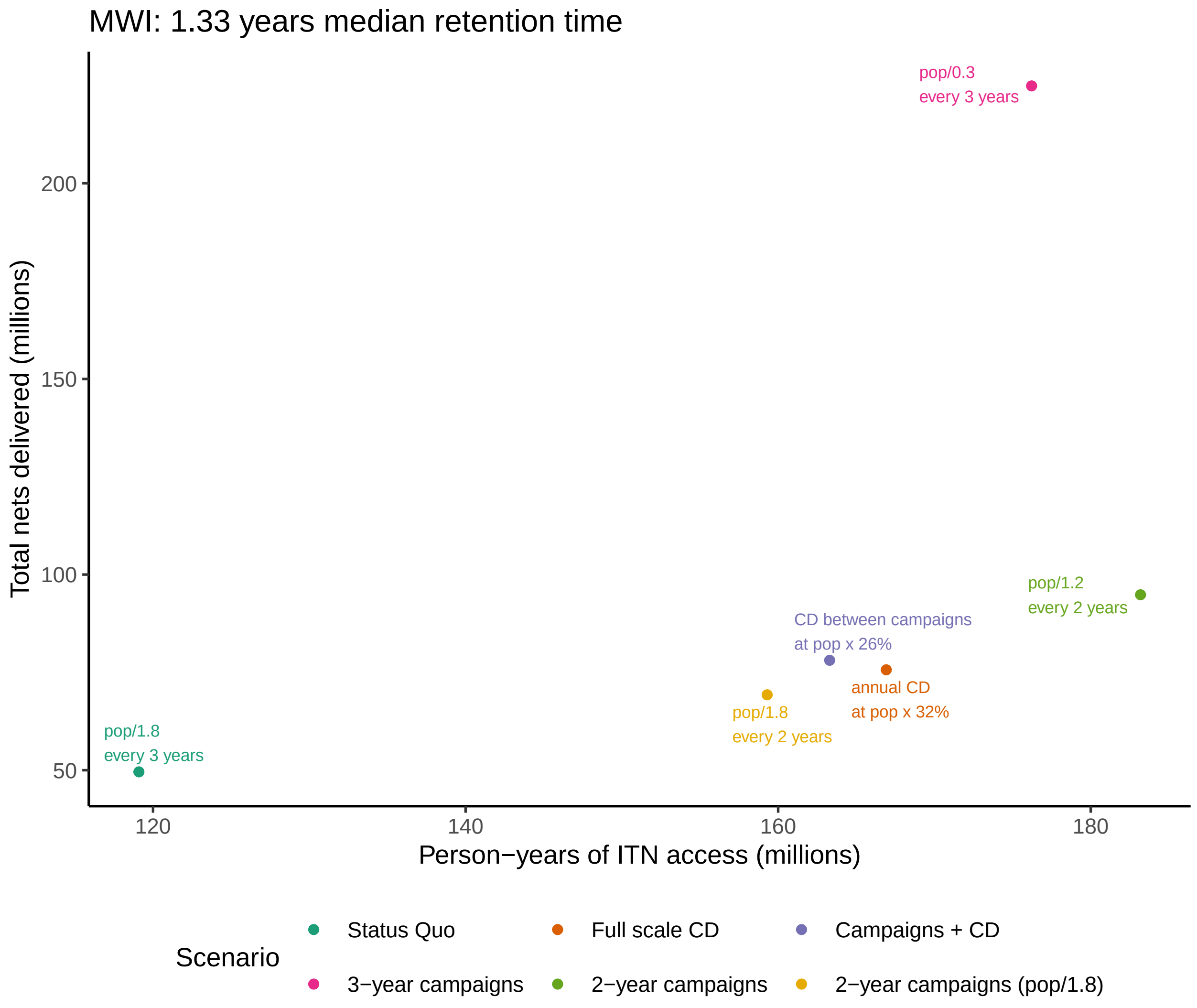
MWI: 1.33 years median retention time
Total nets delivered (millions)
Person−years of ITN access (millions)
200
150
100
50
120
140
160
180
pop/0.3
every 3 years
pop/1.2
every 2 years
CD between campaigns
at pop x 26%
annual CD
at pop x 32%
pop/1.8
every 2 years
pop/1.8
every 3 years
Scenario
Status Quo
Full scale CD
Campaigns + CD
3−year campaigns
2−year campaigns
2−year campaigns (pop/1.8)

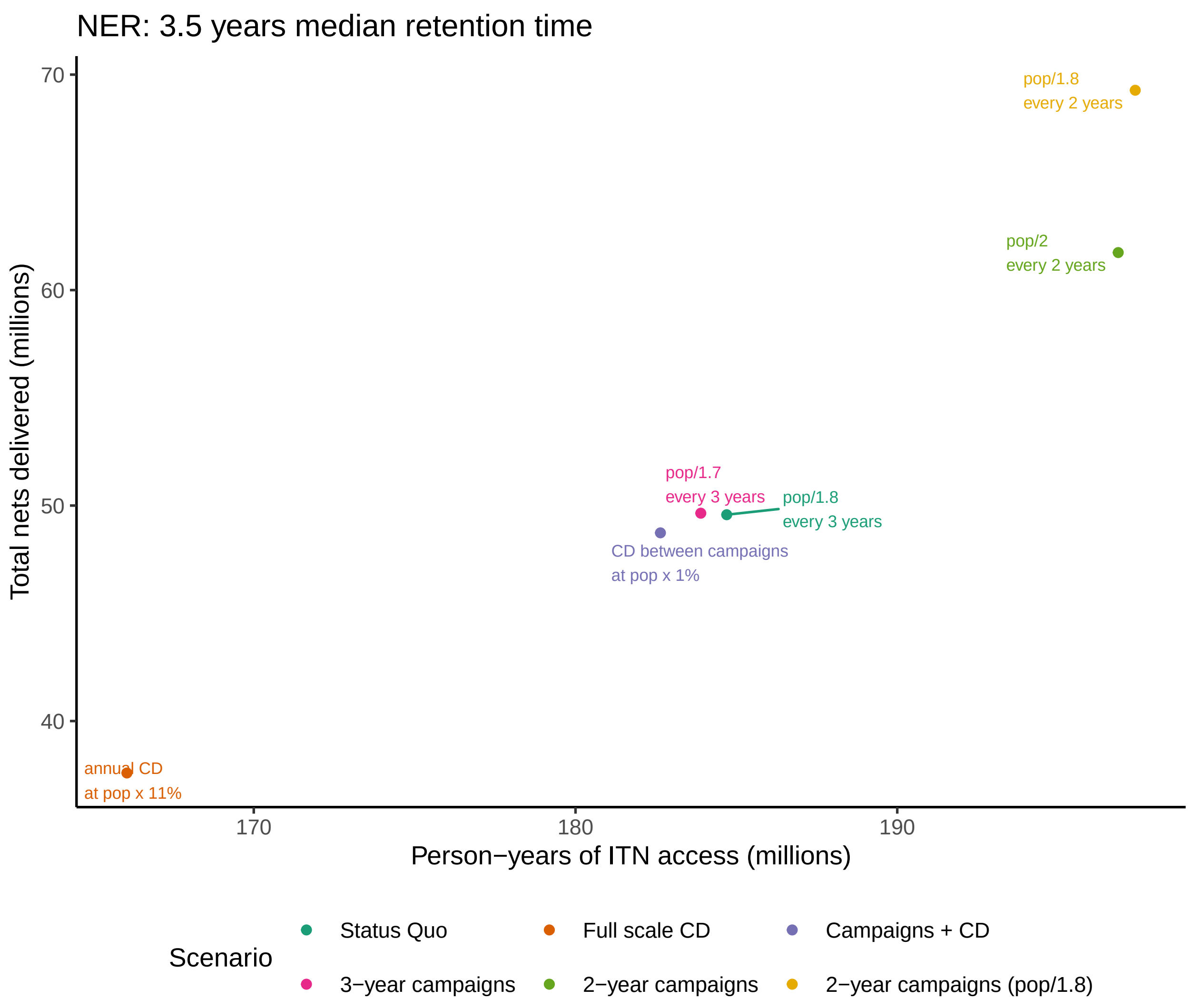
NER: 3.5 years median retention time
Total nets delivered (millions)
70
60
50
40
pop/1.8
every 2 years
pop/2
every 2 years
pop/1.7
every 3 years
pop/1.8
every 3 years
CD between campaigns
at pop x 1%
annual CD
at pop x 11%
170
180
190
Person−years of ITN access (millions)
Scenario
Status Quo
Full scale CD
Campaigns + CD
3−year campaigns
2−year campaigns
2−year campaigns (pop/1.8)

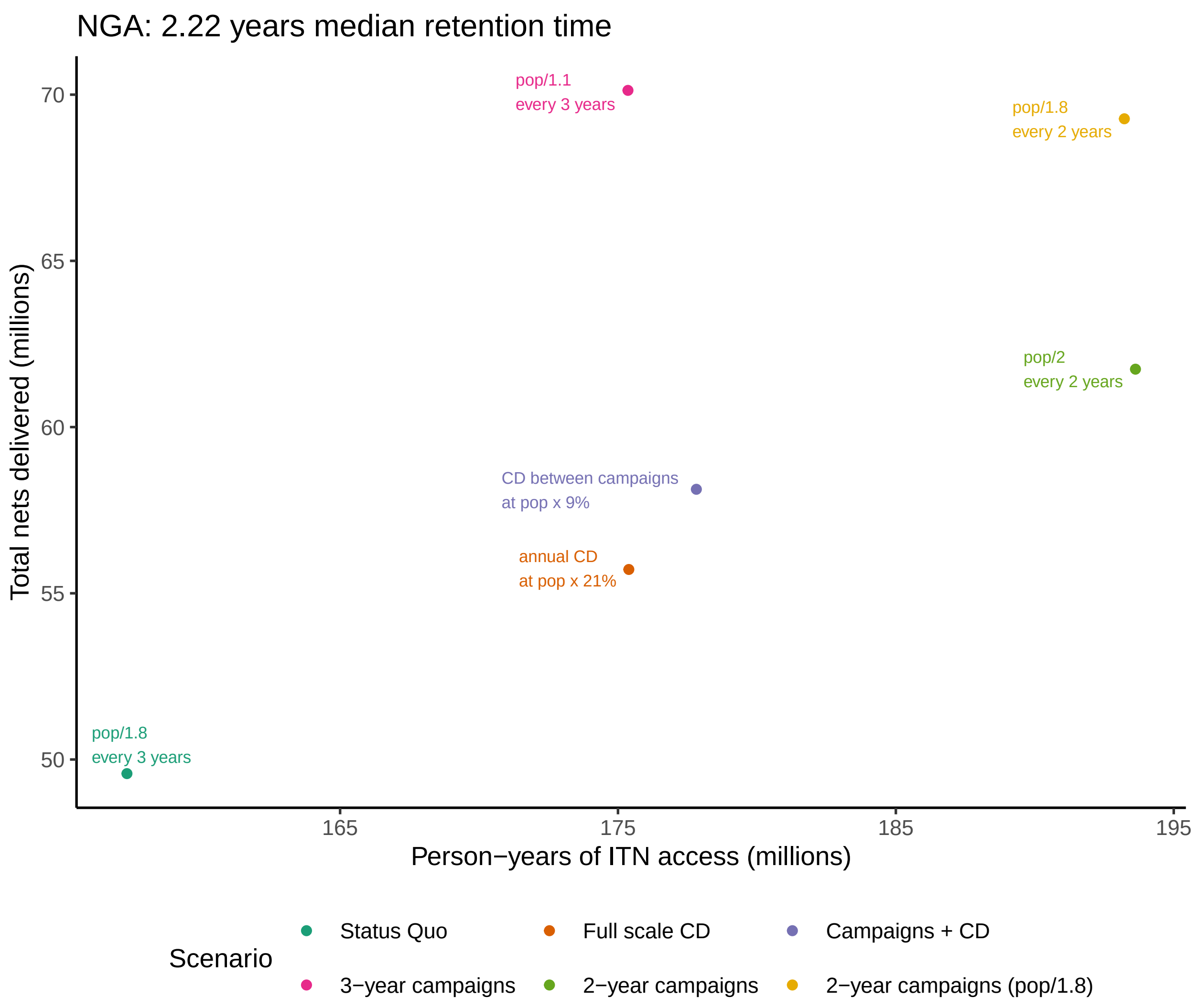
NGA: 2.22 years median retention time
Total nets delivered (millions)
70
65
60
55
50
pop/1.1
every 3 years
pop/1.8
every 2 years
pop/2
every 2 years
CD between campaigns
at pop x 9%
annual CD
at pop x 21%
pop/1.8
every 3 years
165
175
185
195
Person−years of ITN access (millions)
Scenario
Status Quo
Full scale CD
Campaigns + CD
3−year campaigns
2−year campaigns
2−year campaigns (pop/1.8)

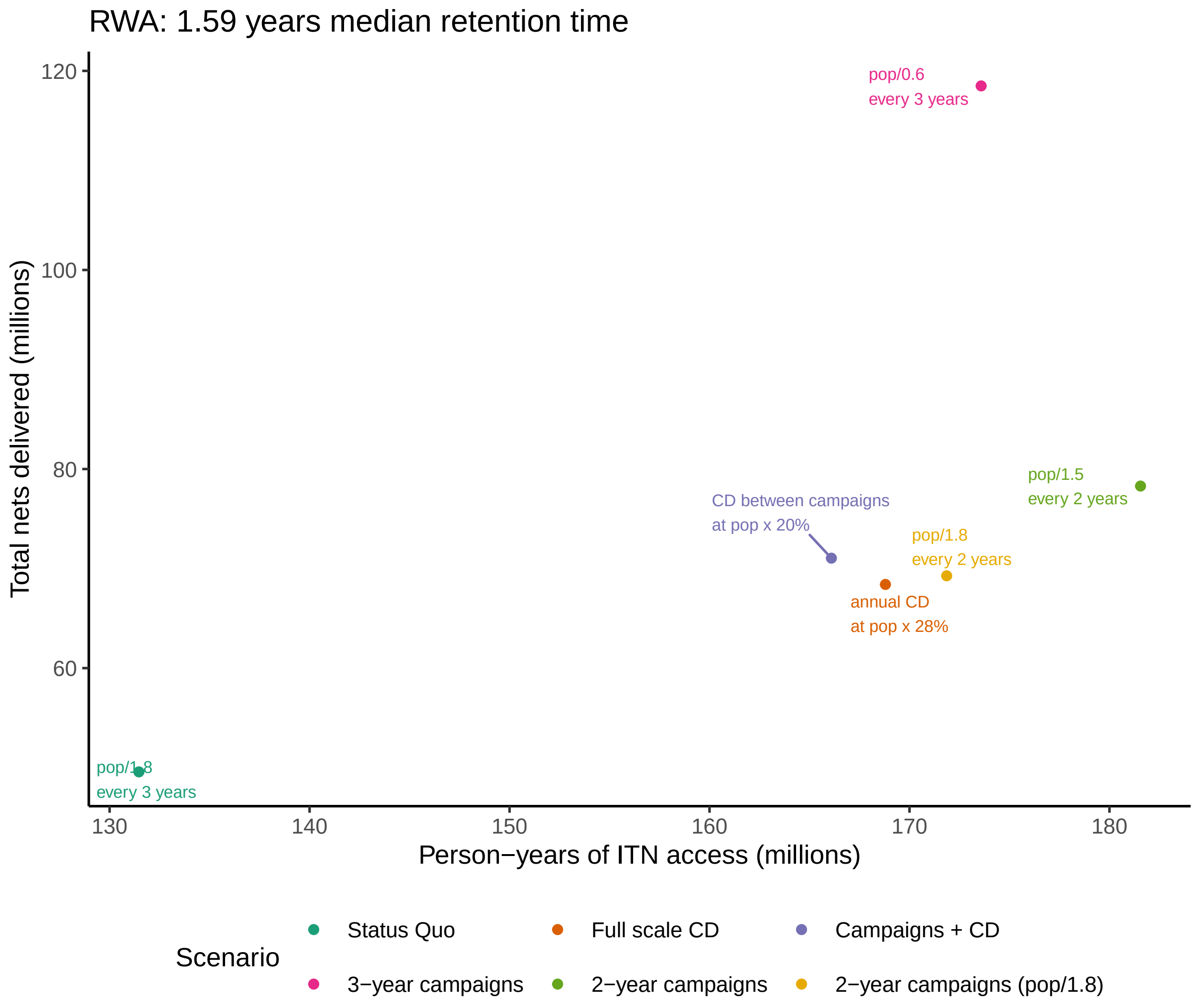
RWA: 1.59 years median retention time
Total nets delivered (millions)
Person−years of ITN access (millions)
120
100
80
60
130
140
150
160
170
180
pop/0.6
every 3 years
pop/1.5
every 2 years
CD between campaigns
at pop x 20%
pop/1.8
every 2 years
annual CD
at pop x 28%
pop/1.8
every 3 years
Scenario
Status Quo
Full scale CD
Campaigns + CD
3−year campaigns
2−year campaigns
2−year campaigns (pop/1.8)

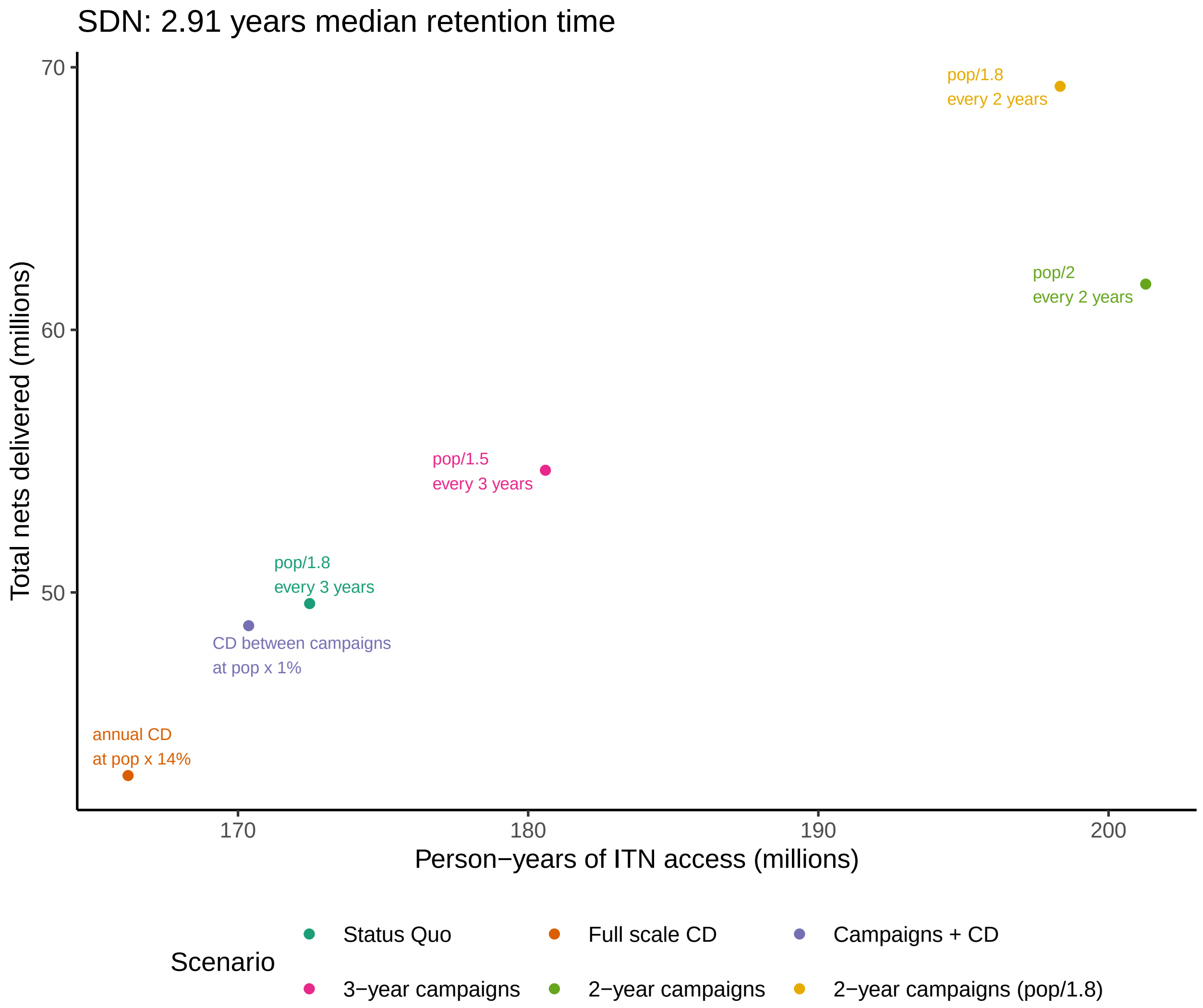

SDN: 2.91 years median retention time
Total nets delivered (millions)
70
60
50
pop/1.8
every 2 years
pop/2
every 2 years
pop/1.5
every 3 years
pop/1.8
every 3 years
CD between campaigns
at pop x 1%
annual CD
at pop x 14%
170
180
190
200
Person−years of ITN access (millions)
Scenario
Status Quo
Full scale CD
Campaigns + CD
3−year campaigns
2−year campaigns
2−year campaigns (pop/1.8)

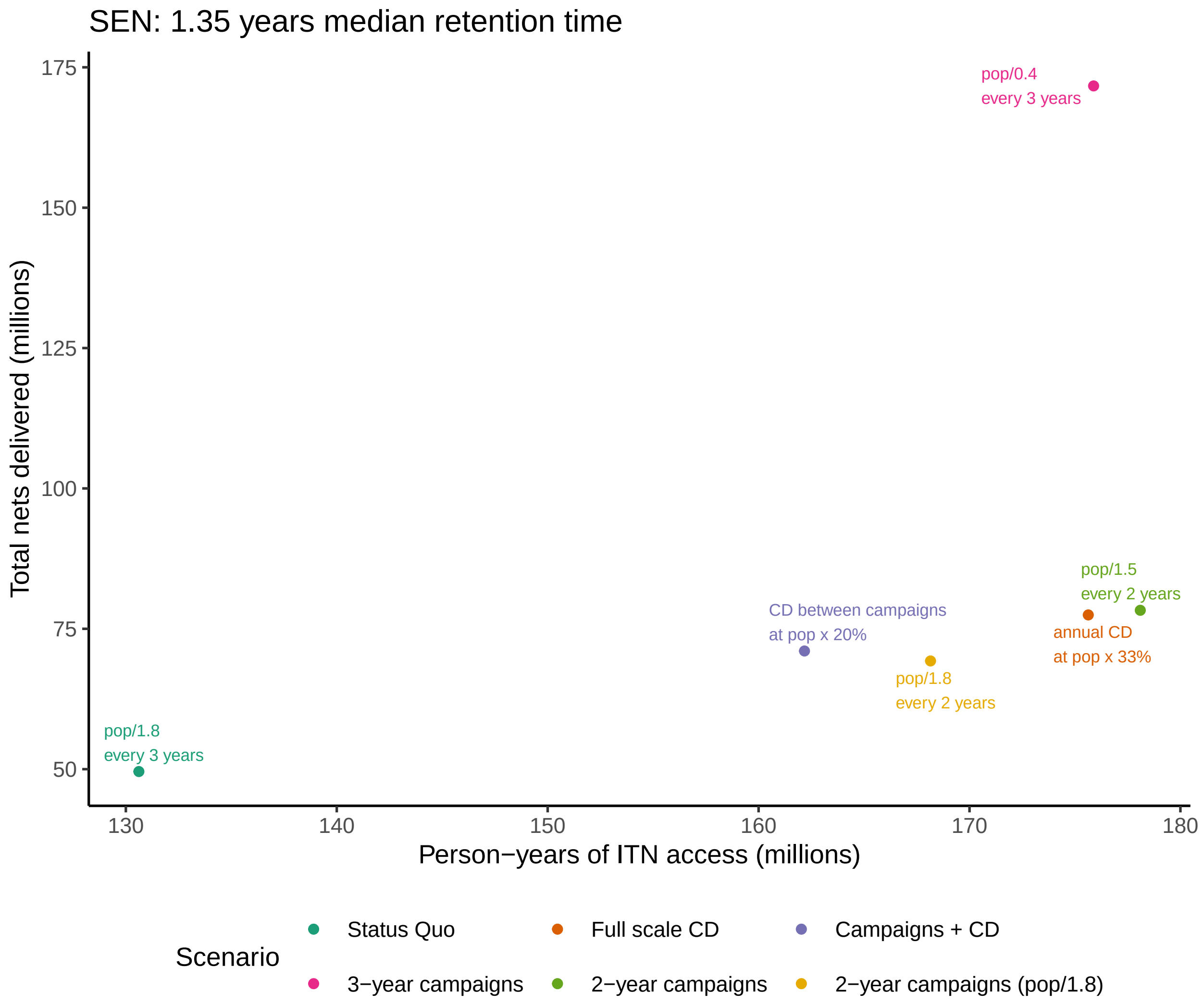
SEN: 1.35 years median retention time
Total nets delivered (millions)
175
150
125
100
75
50
130
140
150
160
170
180
Person−years of ITN access (millions)
pop/0.4
every 3 years
pop/1.5
every 2 years
CD between campaigns
at pop x 20%
annual CD
at pop x 33%
pop/1.8
every 2 years
pop/1.8
every 3 years
Scenario
Status Quo
Full scale CD
Campaigns + CD
3−year campaigns
2−year campaigns
2−year campaigns (pop/1.8)

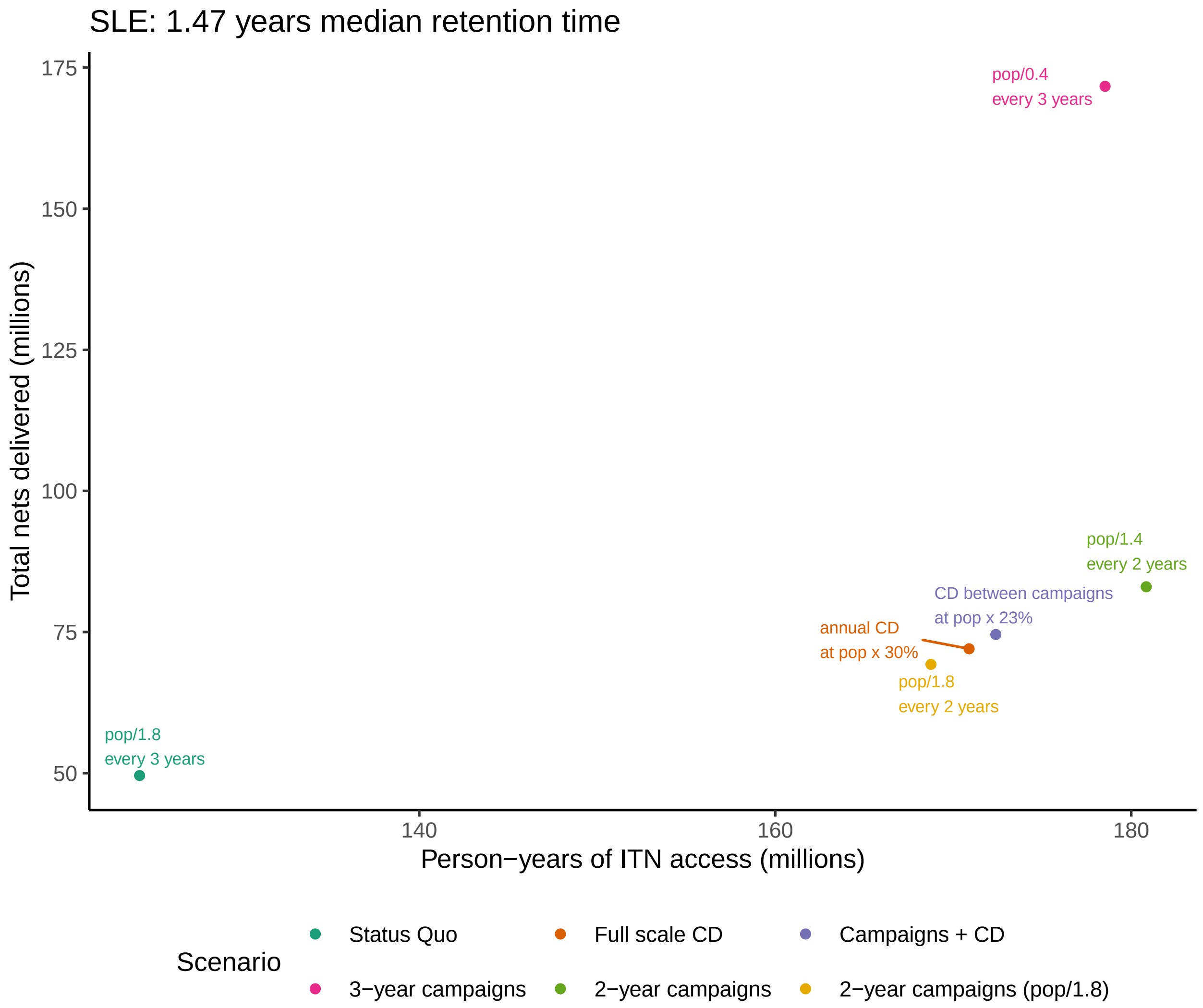

SLE: 1.47 years median retention time
Total nets delivered (millions)
175
150
125
100
75
50
140
160
180
Person−years of ITN access (millions)
pop/0.4
every 3 years
pop/1.4
every 2 years
CD between campaigns
at pop x 23%
annual CD
at pop x 30%
pop/1.8
every 2 years
pop/1.8
every 3 years
Scenario
Status Quo
Full scale CD
Campaigns + CD
3−year campaigns
2−year campaigns
2−year campaigns (pop/1.8)

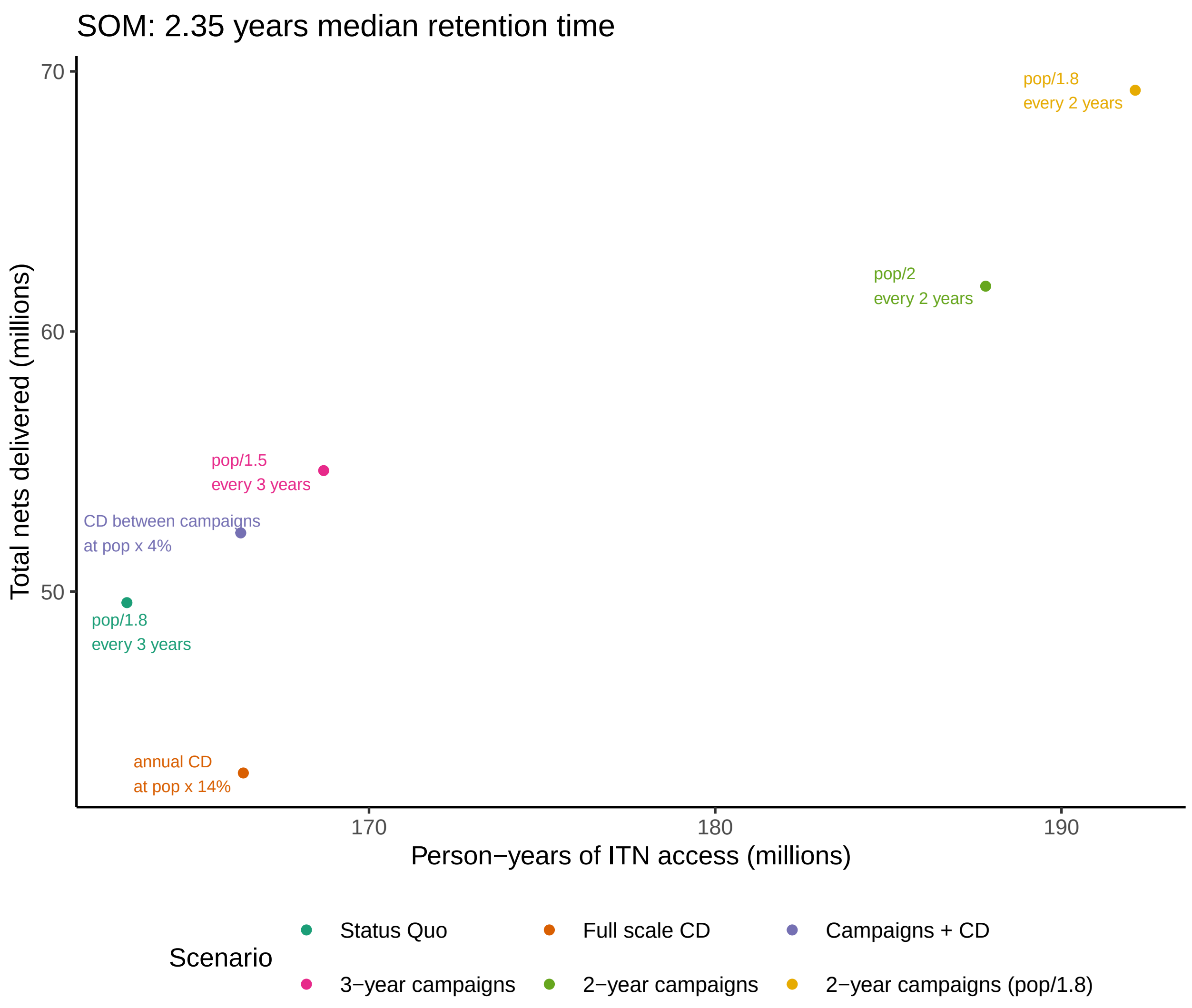
SOM: 2.35 years median retention time
Total nets delivered (millions)
70
60
50
170
180
190
Person−years of ITN access (millions)
pop/1.8
every 2 years
pop/2
every 2 years
pop/1.5
every 3 years
CD between campaigns
at pop x 4%
pop/1.8
every 3 years
annual CD
at pop x 14%
Scenario
Status Quo
Full scale CD
Campaigns + CD
3−year campaigns
2−year campaigns
2−year campaigns (pop/1.8)

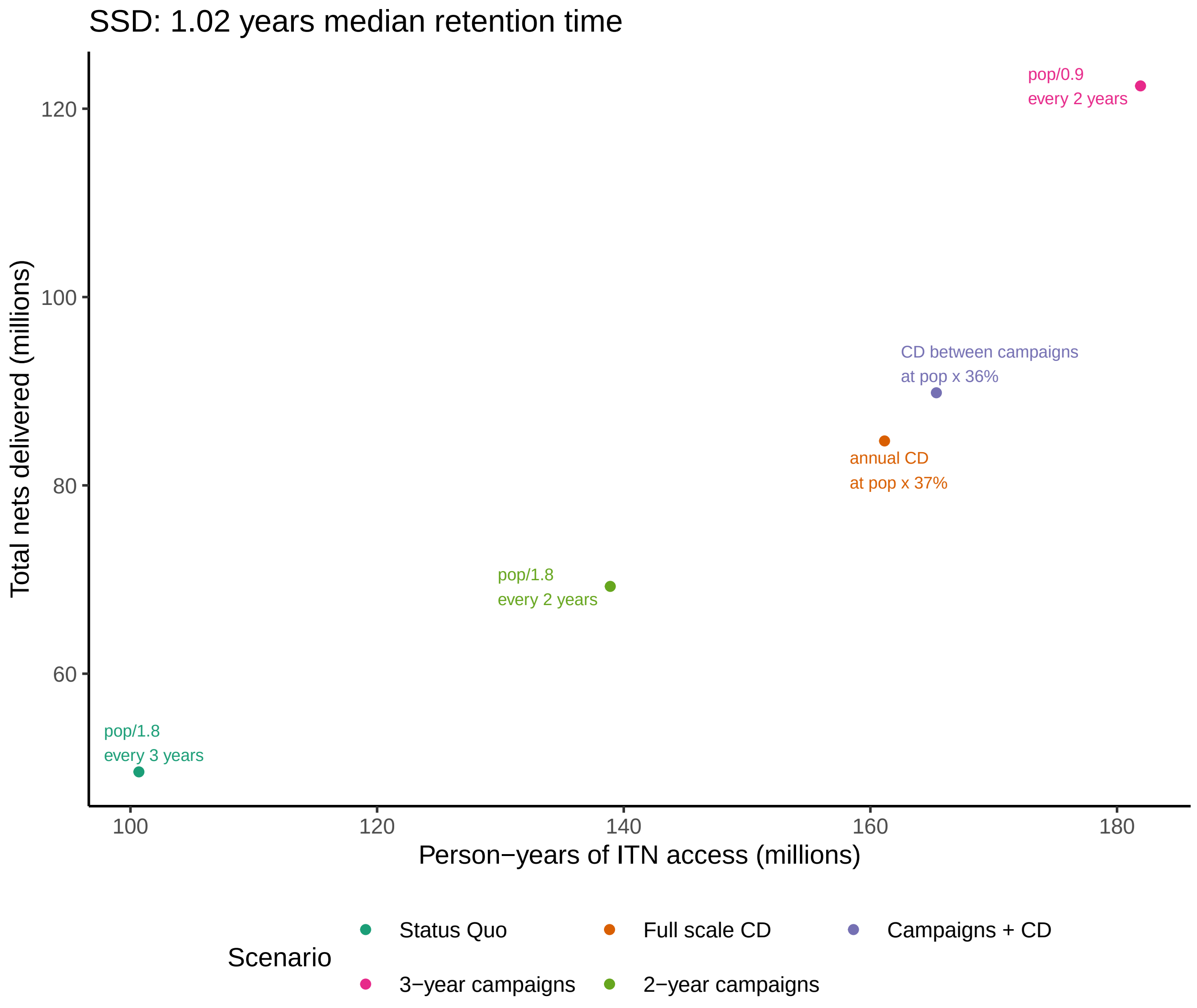
SSD: 1.02 years median retention time
Total nets delivered (millions)
60
80
100
120
Person−years of ITN access (millions)
100
120
140
160
180
pop/0.9
every 2 years
CD between campaigns
at pop x 36%
annual CD
at pop x 37%
pop/1.8
every 2 years
pop/1.8
every 3 years
Scenario
Status Quo
Full scale CD
Campaigns + CD
3−year campaigns
2−year campaigns

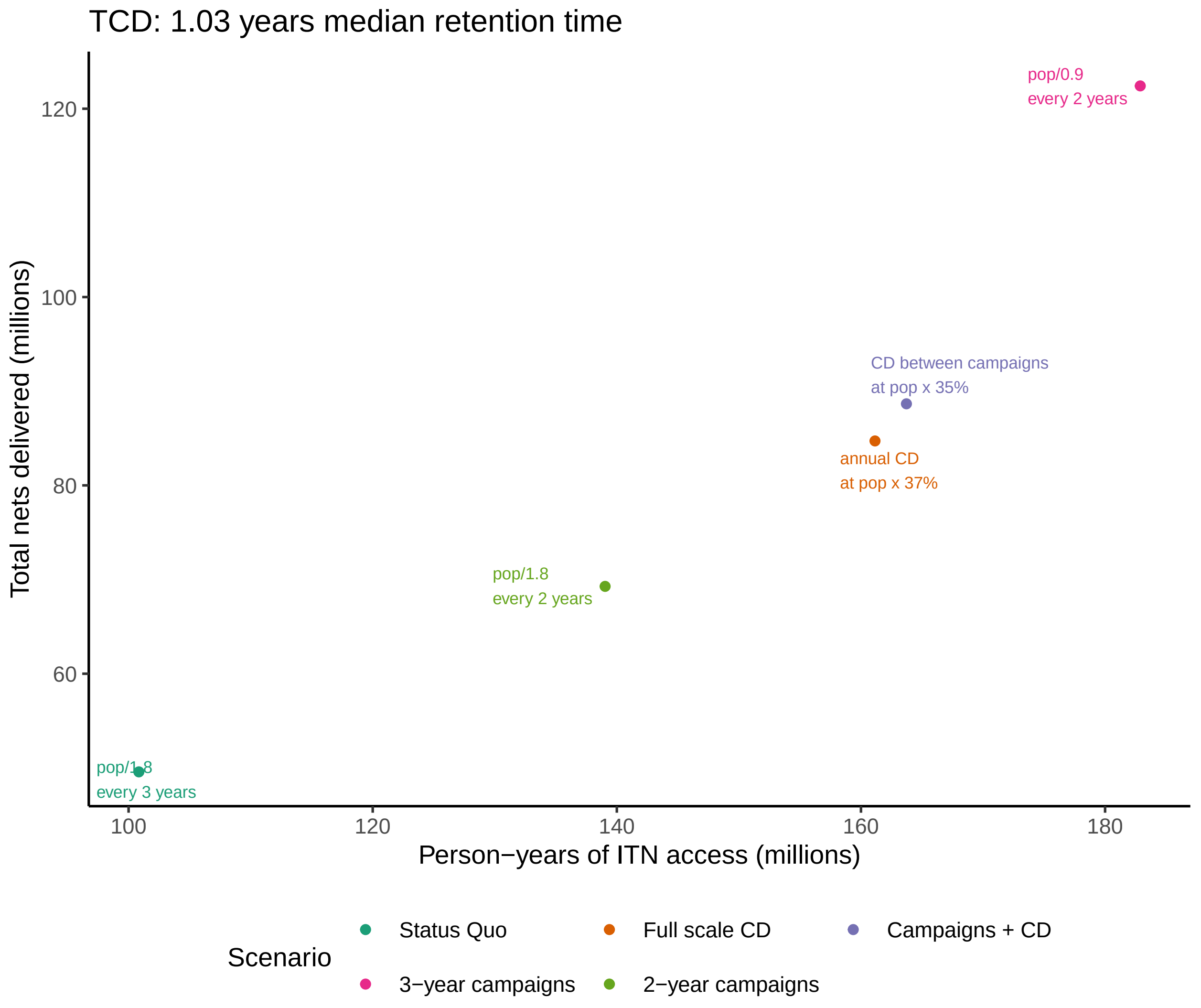

TCD: 1.03 years median retention time
Total nets delivered (millions)
Person−years of ITN access (millions)
120
100
80
60
100
120
140
160
180
pop/0.9
every 2 years
CD between campaigns
at pop x 35%
annual CD
at pop x 37%
pop/1.8
every 2 years
pop/1.8
every 3 years
Scenario
Status Quo
Full scale CD
Campaigns + CD
3−year campaigns
2−year campaigns

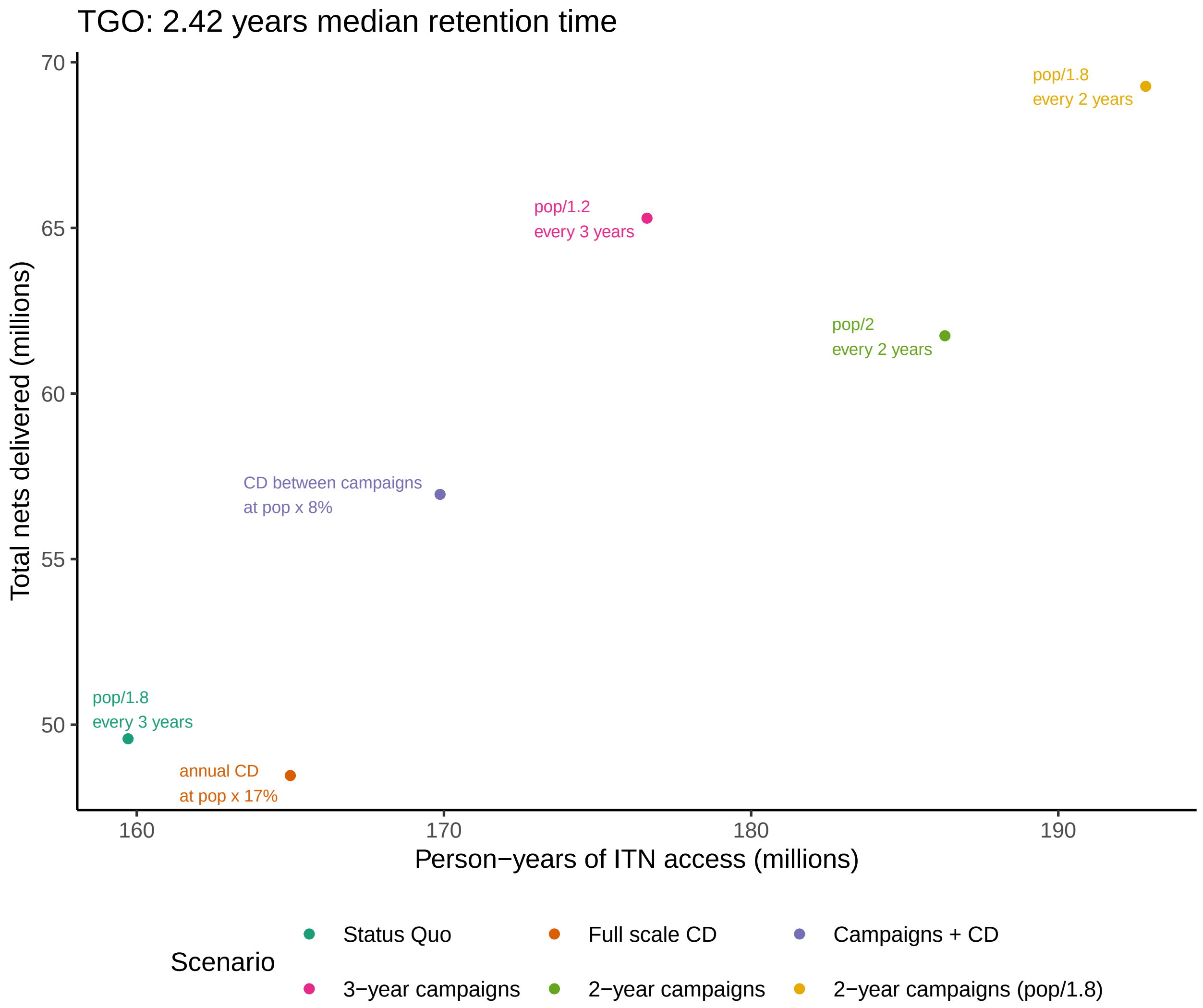
TGO: 2.42 years median retention time
Total nets delivered (millions)
70
65
60
55
50
pop/1.8
every 2 years
pop/1.2
every 3 years
pop/2
every 2 years
CD between campaigns
at pop x 8%
pop/1.8
every 3 years
annual CD
at pop x 17%
160
170
180
190
Person−years of ITN access (millions)
Scenario
Status Quo
Full scale CD
Campaigns + CD
3−year campaigns
2−year campaigns
2−year campaigns (pop/1.8)

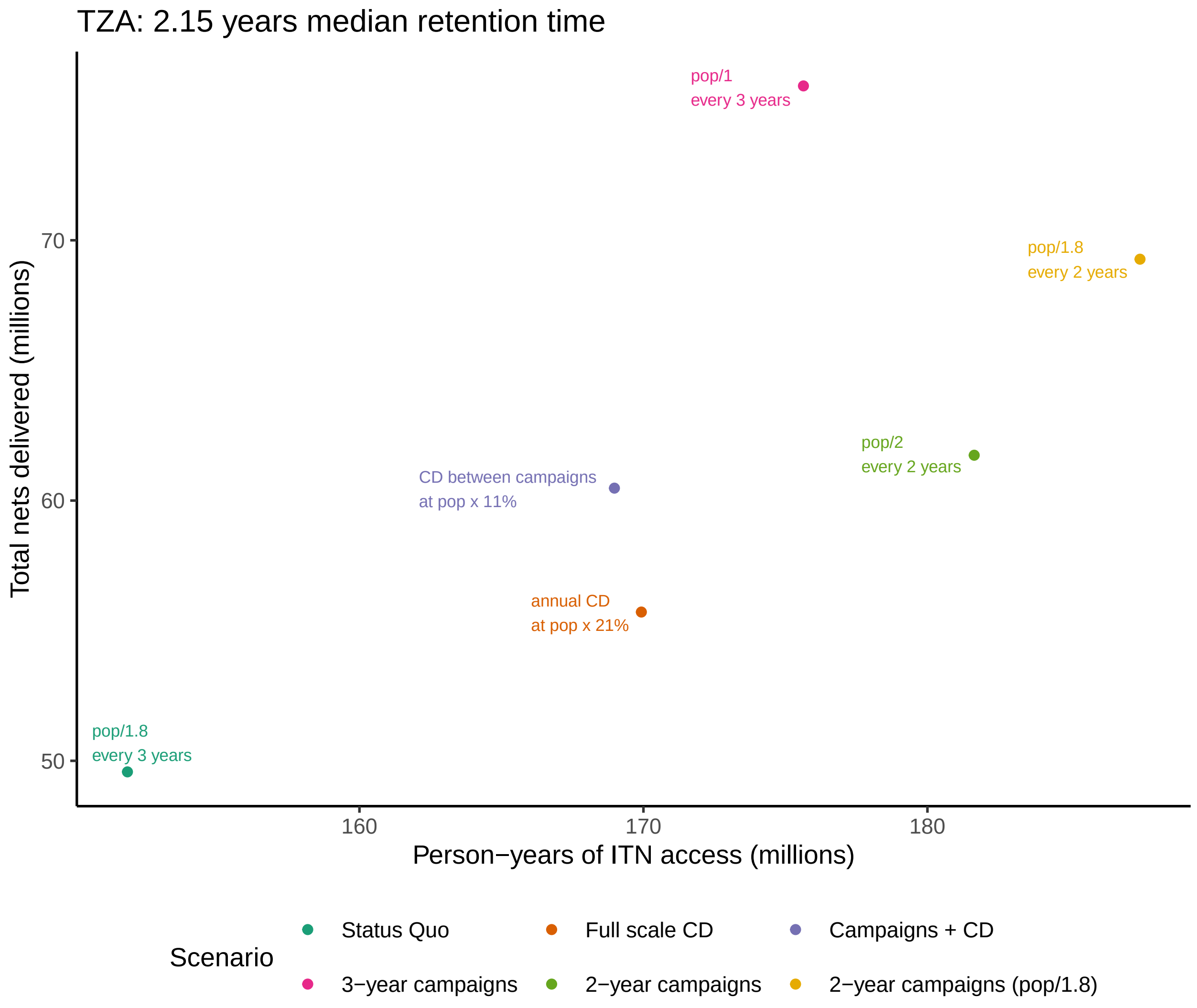
TZA: 2.15 years median retention time
Total nets delivered (millions)
Person−years of ITN access (millions)
70
60
50
160
170
180
pop/1
every 3 years
pop/1.8
every 2 years
pop/2
every 2 years
CD between campaigns
at pop x 11%
annual CD
at pop x 21%
pop/1.8
every 3 years
Scenario
Status Quo
Full scale CD
Campaigns + CD
3−year campaigns
2−year campaigns
2−year campaigns (pop/1.8)

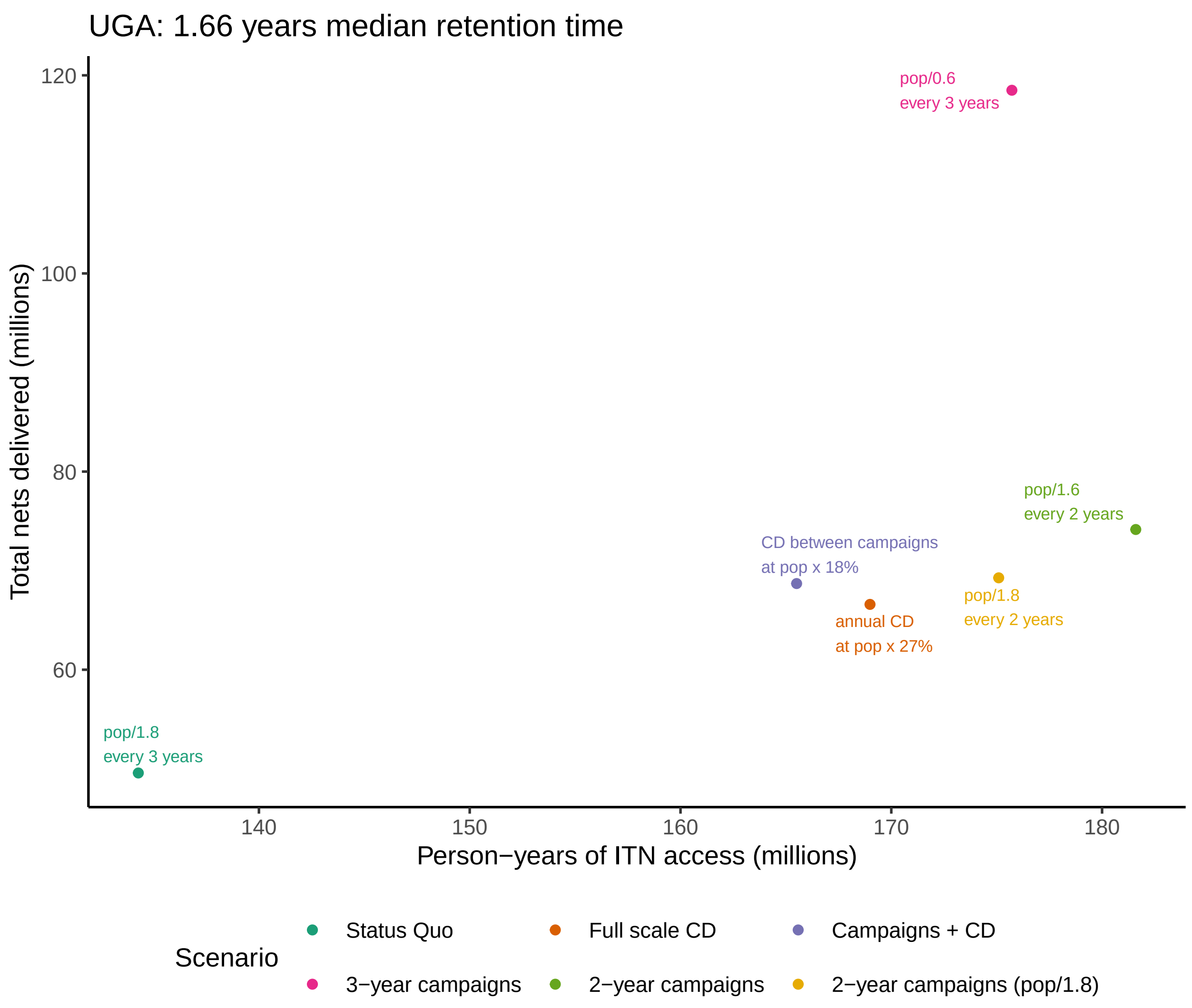
UGA: 1.66 years median retention time
Total nets delivered (millions)
120
100
80
60
140
150
160
170
180
Person−years of ITN access (millions)
pop/0.6
every 3 years
pop/1.6
every 2 years
CD between campaigns
at pop x 18%
pop/1.8
every 2 years
annual CD
at pop x 27%
pop/1.8
every 3 years
Scenario
Status Quo
Full scale CD
Campaigns + CD
3−year campaigns
2−year campaigns
2−year campaigns (pop/1.8)

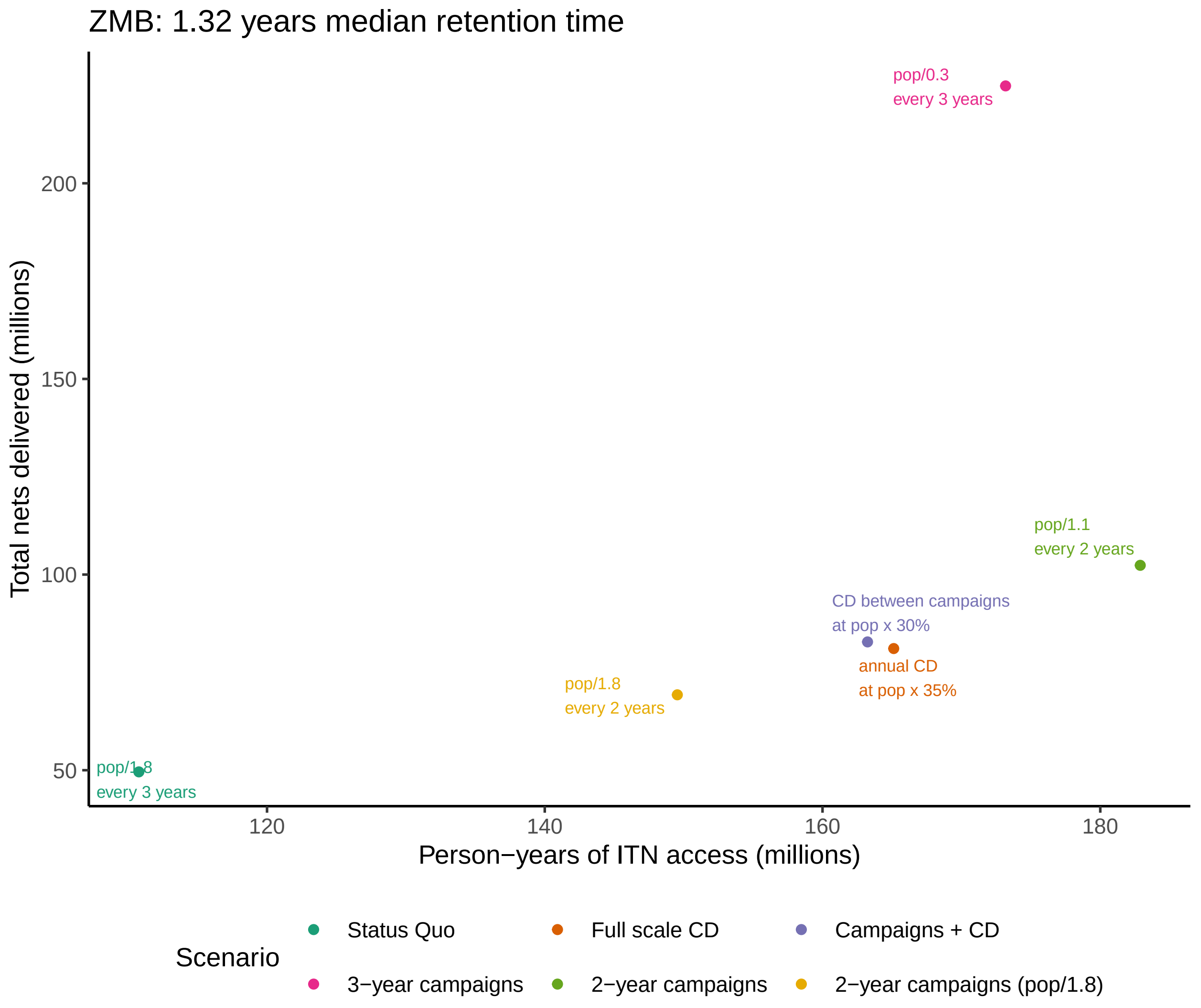
ZMB: 1.32 years median retention time
pop/0.3
every 3 years
pop/1.1
every 2 years
CD between campaigns
at pop x 30%
annual CD
at pop x 35%
pop/1.8
every 2 years
pop/1.8
every 3 years
Total nets delivered (millions)
200
150
100
50
120
140
160
180
Person−years of ITN access (millions)
Scenario
Status Quo
Full scale CD
Campaigns + CD
3−year campaigns
2−year campaigns
2−year campaigns (pop/1.8)

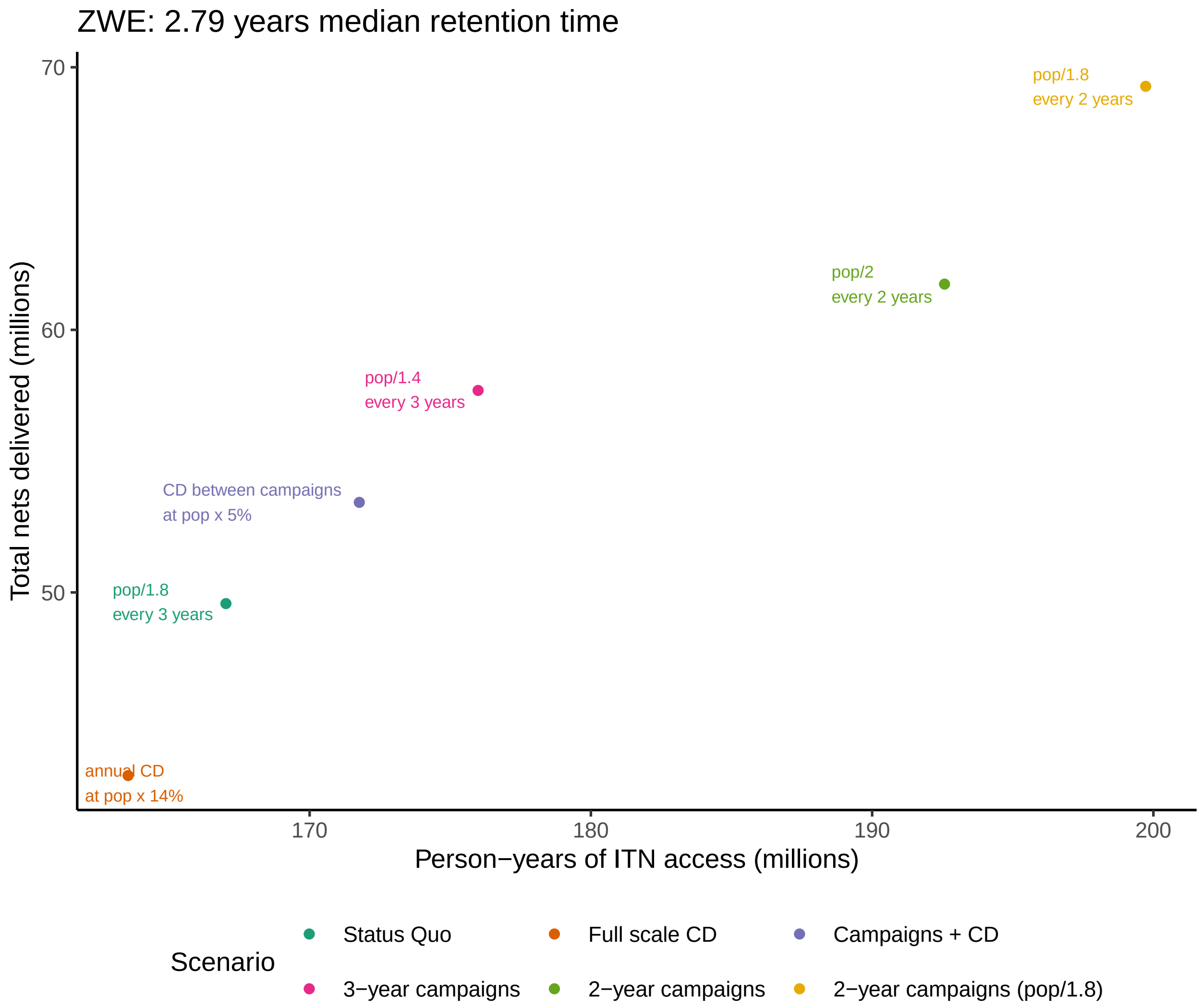
ZWE: 2.79 years median retention time
Total nets delivered (millions)
70
60
50
Person−years of ITN access (millions)
170
180
190
200
pop/1.8
every 2 years
pop/2
every 2 years
pop/1.4
every 3 years
CD between campaigns
at pop x 5%
pop/1.8
every 3 years
annual CD
at pop x 14%
Scenario
Status Quo
Full scale CD
Campaigns + CD
3−year campaigns
2−year campaigns
2−year campaigns (pop/1.8)
